# Gold-catalyzed [4+3]- and [4+2]-annulations of 3-en-1-ynamides with isoxazoles *via* novel 6π-electrocyclizations of 3-azahepta trienyl cations†
†Electronic supplementary information (ESI) available. CCDC 1589549, 1589562, 1589561, 1589558, 1589559 and 1589560. For ESI and crystallographic data in CIF or other electronic format see DOI: 10.1039/c8sc00232k


**DOI:** 10.1039/c8sc00232k

**Published:** 2018-02-19

**Authors:** Sovan Sundar Giri, Rai-Shung Liu

**Affiliations:** a Department of Chemistry , National Tsing-Hua University , Hsinchu , Taiwan , Republic of China . Email: rsliu@mx.nthu.edu.tw

## Abstract

New gold-catalyzed [4+3]-annulations of 3-en-1-ynamides with isoxazoles afford 4*H*-azepines efficiently; this process involves 6π electrocyclizations of gold-stabilized 3-azaheptatrienyl cations. In the presence of Zn(OTf)_2_, the resulting 4*H*-azepines undergo skeletal rearrangement to furnish substituted pyridine derivatives. We subsequently develop new catalytic [4+2]-annulations between the same 3-en-1-ynamides and isoxazoles to deliver substituted pyridine products using Au(i)/Zn(ii) catalysts. This work reports the first success of the 6π electrocyclizations of heptatrienyl cations that are unprecedented in literature reports.

## Abstract 2

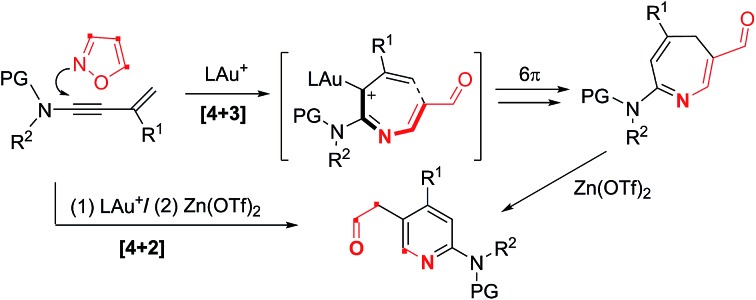
Gold-catalysed [4+3]- and [4+2]-annulations of 3-substituted 3-en-1-ynamides with isoxazoles were achieved with Au(i) and Au(i)/Zn(ii) catalysts respectively.

## Introduction

Electrocyclizations of acyclic conjugated π-motifs are powerful tools to access five-, six- and seven-membered carbocycles;^1^ prominent examples include Nazarov cyclizations of pentadienyl cations^2^ and 6π electrocyclizations of trienes,^3^ which have found widespread applications in organic synthesis.

**Scheme 1:**
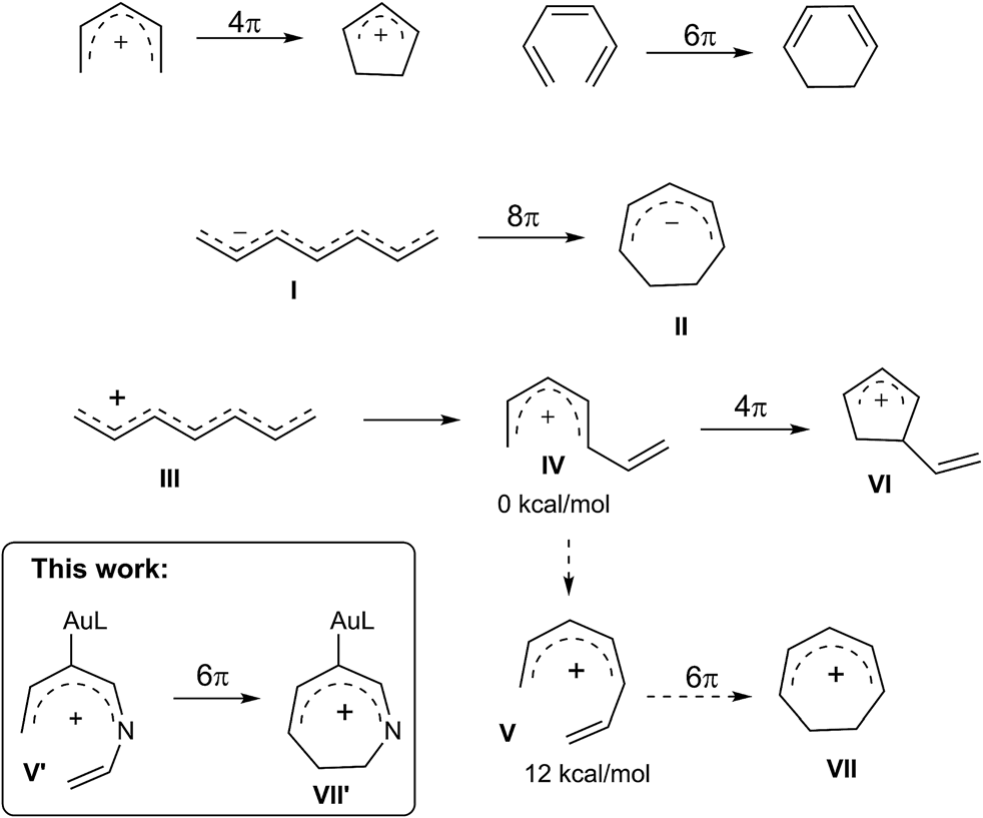
Electrocyclizations of conjugated π-motifs.

In the context of seven-carbon π-motifs, heptatrienyl anions **I** undergo facile 8π electrocyclizations *via* rapid interconversions among various anion configurations (Scheme 1).^4^ In contrast, heptatrienyl cations **III**^5^ exclusively undergo Nazarov reactions because of the difficulties of forming all σ-*cis* configured cations **V** that have a high energy state.5*b* 1-Aza- and 1-oxaheptatrienyl cations^6^ were also reported to follow Nazarov cyclizations. The realization of a 6π electrocyclization of conjugated seven-membered cations is formidable but challenging. This work reveals the first success of such seven-membered cyclizations of gold-stabilized 3-azaheptatrienyl cations **V′** to form azacyclic products **3–4***via* a new C–C bond formation.
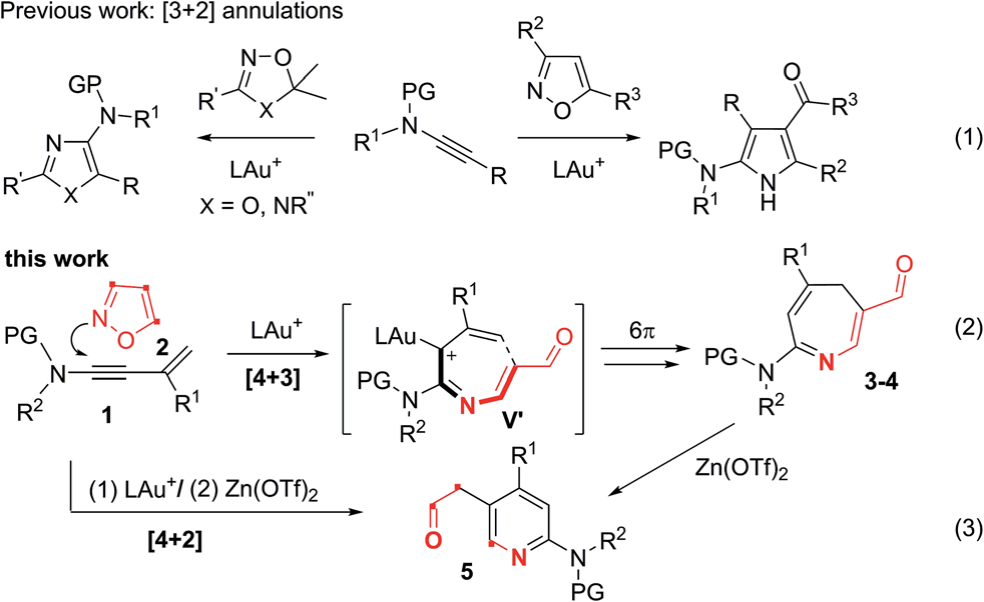


The advent of gold catalysis has inspired new annulations between alkynes and poor nucleophiles.^7^ N–O containing nucleophiles serve as useful building blocks to construct valuable azacyclic frameworks.^7^ Ye and Hashmi reported interesting [3+2]-annulations of isoxazoles or benzisoxazoles with electron-rich ynamides, yielding substituted pyrrole derivatives through aza-Nazarov cyclizations of the key intermediate [eqn (1)].^7^,^8^ These [3+2]-annulations were extensively expanded to other N–O heterocycles including benzisoxazoles, 1,2,4-oxadiazoles, 1,4,2-dioxazoles and 4,5-dihydro-1,2,4-oxadiazoles, yielding additional five-membered azacycles as depicted in [eqn (1)].^9^ Here, we report two distinct [4+3]- and [4+2]-annulations between 3-en-1-ynamides and isoxazoles using varied catalysts. An Au(i) catalyst alone delivers 4*H*-azepines **3–4** through 6π electrocyclizations of intermediates **V′** [eqn (2)] whereas a combined action of Au(i)/Zn(ii) on the same reactants furnishes highly functionalized pyridines **5** [eqn (3)]. With our convenient synthesis, the synthetic utility of new 4*H*-azepines **3–4** is also reported.^10^

## Results and discussion

We examined the reactions of 3-methyl-3-en-1-ynamide **1a** with 3,5-dimethylisoxazole **2a** using various gold catalysts. Heating this mixture (**1a**/**2a** = 1 : 2 ratio) in hot DCE with 5 mol% LAuCl/AgNTf_2_ [L = *p*(*t*-Bu)_2_(*o*-biphenyl) and IPr] afforded a [4+3]-annulation product, 4*H*-azepine **3a**, in 64% and 75% yields respectively (Table 1, entries 1–2). Under these conditions, a low loading (1.2 equiv.) of 3,5-dimethylisoxazole **2a** gave **3a** in a decreased yield, *ca.* 62% (entry 3). With a 10 mol% catalyst, IPrAuCl/AgNTf_2_ gave a clean reaction, yielding desired **3a** up to 91% (entry 4). We tested other phosphine ligands such as PPh_3_ and P(OPh)_3_, yielding desired **3a** in satisfactory yields (78–81%, entries 5–6). Other counter anions such as OTf^–^ and SbF_6_^–^ were also effective in producing **3a** in 85–88% yields (entries 7–8). AgNTf_2_ alone was not active at all (entry 9).

**Table 1:** [4+3]-Annulations over various gold catalysts

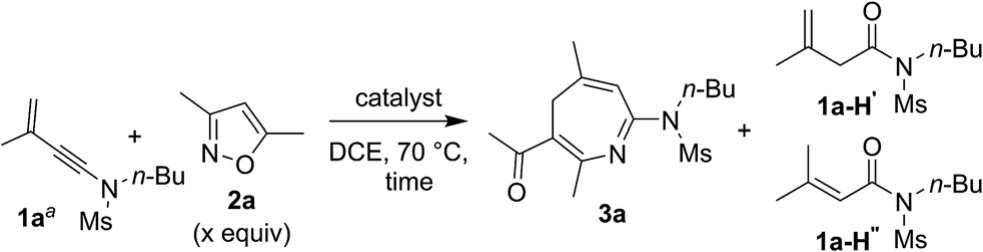						
Entry	Catalyst [mol%]	x	Time [h]	Yield^*b*^ [%]		
				**1a**	**3a**	**1a–H′**/**1a–H′′**
1^*c*^	LAuCl/AgNTf_2_ [5]	2	3	20	64	—
2^*d*^	IPrAuCl/AgNTf_2_ [5]	2	7	12	75	7 [2.5 : 1]
3	IPrAuCl/AgNTf_2_ [5]	1.2	7	23	62	5 [1 : 1]
**4**	**IPrAuCl/AgNTf**_**2**_ **[10]**	**2**	**3**	**—**	**91**	**Trace**
5	PPh_3_AuCl/AgNTf_2_ [10]	2	3.5	—	81	5 [1.25 : 1]
6	[PhO]_3_PAuCl/AgNTf_2_ [10]	2	3.5	—	78	13 [1.1 : 1]
7	IPrAuCl/AgSbF_6_ [10]	2	2.5	—	85	6 [1.4 : 1]
8	IPrAuCl/AgOTf [10]	2	2	—	88	Trace
9	AgNTf_2_ [10]	2	15	33	—	11

^*a*^[**1a**] = 0.15 M.

^*b*^Product yields are reported after separation from a silica column.

^*c*^L = *p*(*t*-Bu)_2_(*o*-biphenyl).

^*d*^IPr = 1,3-bis(diisopropylphenyl)-imidazol-2-ylidene. Ms = methanesulfonyl, DCE = 1,2-dichloroethane, and Tf = trifluoromethanesulfonyl.

Suitable substituents of 3-en-1-ynamides **1** are crucial to achieve 6π cyclizations of 3-azaheptatrienyl cations **V′** [eqn (2)]. We tested the reactions on 3-en-1-ynes **1b–1m** bearing a C(3)-substituent to circumvent aza-Nazarov cyclizations as reported in Ye's work.^7^ Herein, only entries 9 and 10 showed the presence of 3-azanorcaradienes **3′**. We examined these [4+3]-annulations on 3-methyl-3-en-1-ynamides **1b–1e** bearing various sulfonamides NTsR^4^ (R^4^ = Me, cyclopropyl, benzyl and N(*n*-C_4_H_9_)(–SO_2_Bu)), affording the desired 4*H*-azepines **3b–3e** in high yields (84–90%, Table 2, entries 1–4). Nevertheless, this new annulation becomes less efficient for 3-en-1-ynamide **1f** bearing an oxazolidin-2-one to yield product **3f** in 64% yield (entry 5).

**Table 2:** [4+3]-Annulations with various 3-en-1-ynamides

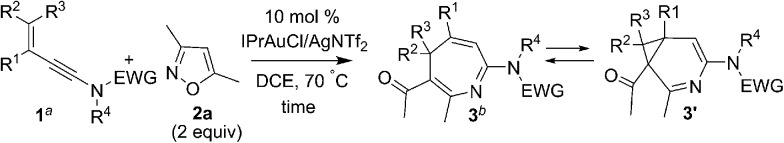
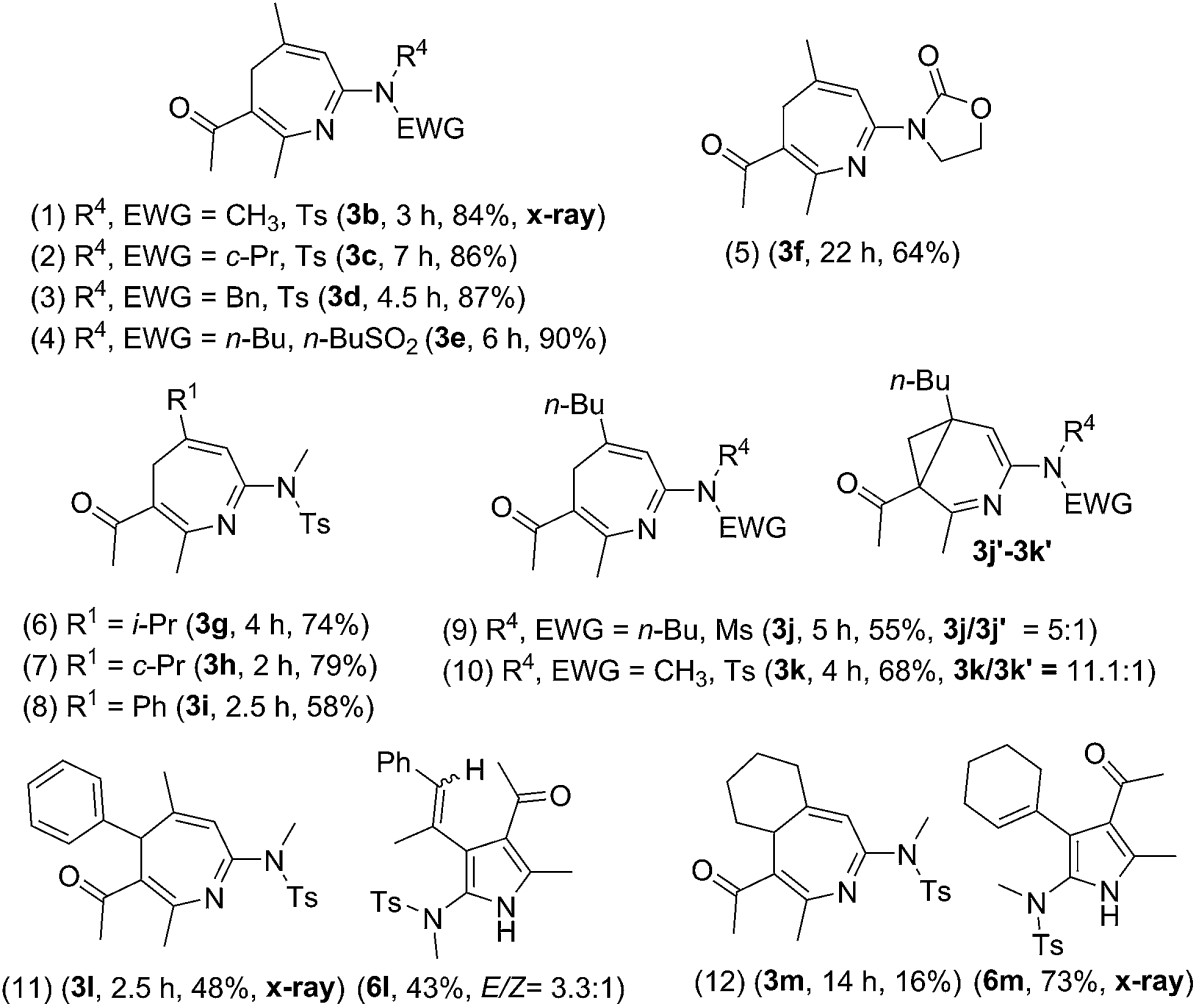

^*a*^[**1**] = 0.15 M.

^*b*^Product yields are reported after separation from a silica column. EWG = electron withdrawing group.

We altered the C(3)-substituents as in substrates **1g–1i**; their resulting products **3g–3h** (R^1^ = isopropyl and cyclopropyl) were obtained in 74–79%, and **3i** (R^1^ = Ph) with only 58% yield (entries 6–8). Notably, when a long *n*-butyl group was present as in species **1j** and **1k**, their corresponding reactions afforded compounds **3j**/**3j′** = 5/1 and **3k**/**3k′** = 11.1 : 1, respectively, in 55% and 68% yields (entries 9–10). For *E*-configured trisubstituted 3-en-1-yne **1l** (R^1^ = Me, R^2^ = Ph and R^3^ = H), 4*H*-azepine **3l** and pyrrole **6l** were obtained in equal proportions (entry 11). When a cyclohexenyl group was present for alkene as in species **1m**, pyrrole product **6m** was dominant over azepine **3m** (entry 12). Accordingly, preferable 3-en-1-ynes comprise a small R^2^ or R^3^ substituent whereas R^1^ must be substituted. Herein, the structures of 4*H*-azepines **3b** and **3l**, and pyrrole species **6m** were confirmed with X-ray diffraction.^11^

Isoxazoles of a wide scope are compatible with these [4+3]-annulations, as depicted in Table 3. The reaction of unsubstituted isoxazole **2b** with model 3-en-1-ynamide **1b** afforded the desired 4*H*-azepine **4a** in 84% yield, together with pyrrole **7a′** in only 8% yield (entry 1). Mono-substituted 3-methyl or 5-methyl isoxazoles **2c** and **2d** are also suitable for these annulations to afford compounds **4b** and **4c** in 75% and 87% yields, respectively (entries 2–3). We prepared additional 3,5-disubstituted isoxazoles **2e–2i** with R^1^ = alkyl and phenyl, and R^2^ = alkyl; their annulations proceed smoothly to produce desired **4d–4h** in 69–85% yields (entries 4–8). For di-substituted isoxazoles **2j** and **2k** bearing R^2^ = Ph, 4*H*-azepines **4i** and **4j** were obtained in 61% and 71% yields respectively, together with their rearrangement products **5i** and **5j** in 15–30% yields (entries 9–10). Compounds **4a** and **5i** were characterized by X-ray diffraction.^11^

**Table 3:** [4+3]-Annulations with various isoxazoles

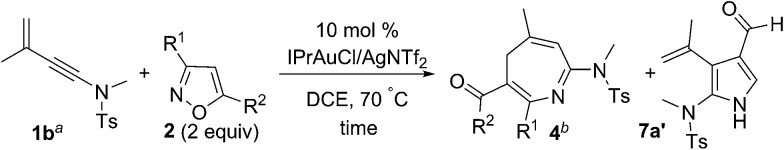					
Entry	(R^1^, R^2^)	**2**	Time [h]	Yield [%]	**4**
(1)	H, H	**2b**	4	84	**4a** (X-ray)
				8	**7a′**
(2)	H, Me	**2d**	3	75	**4b**
(3)	Me, H	**2c**	3	87	**4c**
(4)	Et, Et	**2e**	6	85	**4d**
(5)	*n*-Bu, *n*-Bu	**2f**	7	81	**4e**
(6)	Me, *n*-Bu	**2g**	3	82	**4f**
(7)	*n*-Bu, *c*-Pr	**2h**	2	77	**4g**
(8)	Ph, *n*-Bu	**2i**	4	69	**4h**
(9)	Ph, Ph	**2j**	6.5	61	**4i**
				30	**5i** (X-ray)
(10)	Me, Ph	**2k**	4	71	**4j**
	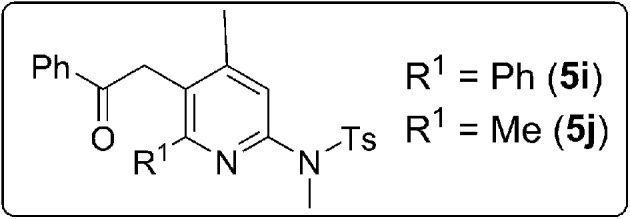			15	**5j**

^*a*^[**1b**] = 0.15 M.

^*b*^Product yields are reported after separation from a silica column.

**Scheme 2:**
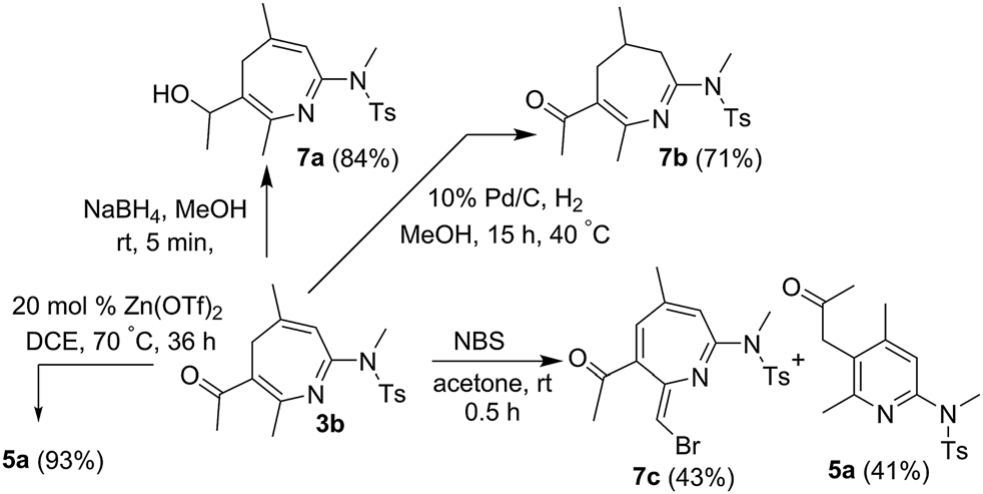
New functionalization of 4*H*-azepines.

Our convenient synthesis of 4*H*-azepines provides new synthetic utilities; several new functionalizations are depicted in Scheme 2. NaBH_4_-reduction of species **3b** delivered an alcohol derivative **7a** in 84% yield. Selective hydrogenation of the same species afforded 2-aza-1,3-dien-5-one **7b** in 71% yield. A final treatment of 4*H*-azepine **3b** with NBS in acetone afforded compound **7c**, of which the molecular structure was determined by ^1^H NOE spectra.

The Lewis-catalyzed rearrangement of 4*H*-azepines **3–4** to substituted pyridines **5** [eqn (3)] is unprecedented in 4*H*-azepine chemistry.^10^ We undertook such novel [4+2]-annulations between 3-en-1-ynamides **1** and isoxazoles **2** using Au(i)/Zn(ii) in a relay series, as depicted in Table 4. In the reactions of various 3-substituted 3-en-1-ynamides **1** (R^1^ = methyl, *n*-butyl, cyclopropyl and isopropyl) with 3,5-dimethylisoxazole **2a**, substituted pyridines **5a–5d** were obtained in satisfactory yields (51–73%, entries 1–4). In entry 1, if the reaction was performed with combined Au(i)/Zn(ii) catalysts in a non-relay operation, compounds **5a** and **3b** were isolated in 35% and 28% yields respectively. For 3-en-1-ynamide **1a** bearing a NMs(*n*-butyl), the corresponding product **5e** was obtained in 63% yield (entry 5). We tested the reactions on 3,5-disubstituted isoxazoles **2e–2f** & **2h** bearing all alkyl substituents, producing desired **5f–5h** in good yields (69–78%, entries 6–8). For such disubstituted isoxazoles bearing R^4^ = Ph, the reactions afforded the desired pyridine derivatives **5i** and **5j** in 75–80% yields (entries 9–10). The molecular structures of compounds **5a** and **5i** were characterized by X-ray diffraction.^11^

**Table 4:** [4+2]-Annulations between 3-en-1-ynamides and isoxazoles

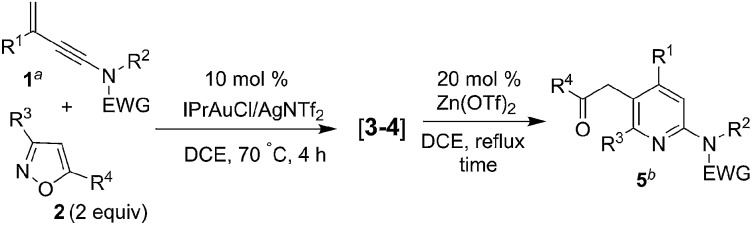							
Entry	(R^1^, R^2^, EWG)	**1**	(R^3^, R^4^)	**2**	Time [h]	Yield [%]	**5**
(1)	Me, Me, Ts	**1b**	Me, Me	**2a**	19	73 (35)^*c*^	**5a** (X-ray)
(2)	*n*-Bu, Me, Ts	**1k**	Me, Me	**2a**	33	64	**5b**
(3)	*c*-Pr, Me, Ts	**1h**	Me, Me	**2a**	20	56	**5c**
(4)	*i*-Pr, Me, Ts	**1g**	Me, Me	**2a**	15	51	**5d**
(5)	Me, *n*-Bu, Ms	**1a**	Me, Me	**2a**	28	63	**5e**
(6)	Me, Me, Ts	**1b**	*n*-Bu, *n*-Bu	**2f**	19	78	**5f**
(7)	Me, Me, Ts	**1b**	Et, Et	**2e**	16	69	**5g**
(8)	Me, Me, Ts	**1b**	*n*Bu, *c*-Pr	**2h**	20	75	**5h**
(9)	Me, Me, Ts	**1b**	Ph, Ph	**2j**	24	80	**5i** (X-ray)
(10)	Me, Me, Ts	**1b**	Me, Ph	**2k**	30	75	**5j**

^*a*^[**1**] = 0.15 M.

^*b*^Product yields are reported after separation from a silica column.

^*c*^The value in parentheses is reported using a mixture of IPrAuCl/AgNTf_2_ (10 mol%) and Zn(OTf)_2_ (20 mol%) in hot DCE (70 °C, 48 h); **3b** was also isolated in 28% yield.

**Scheme 3:**
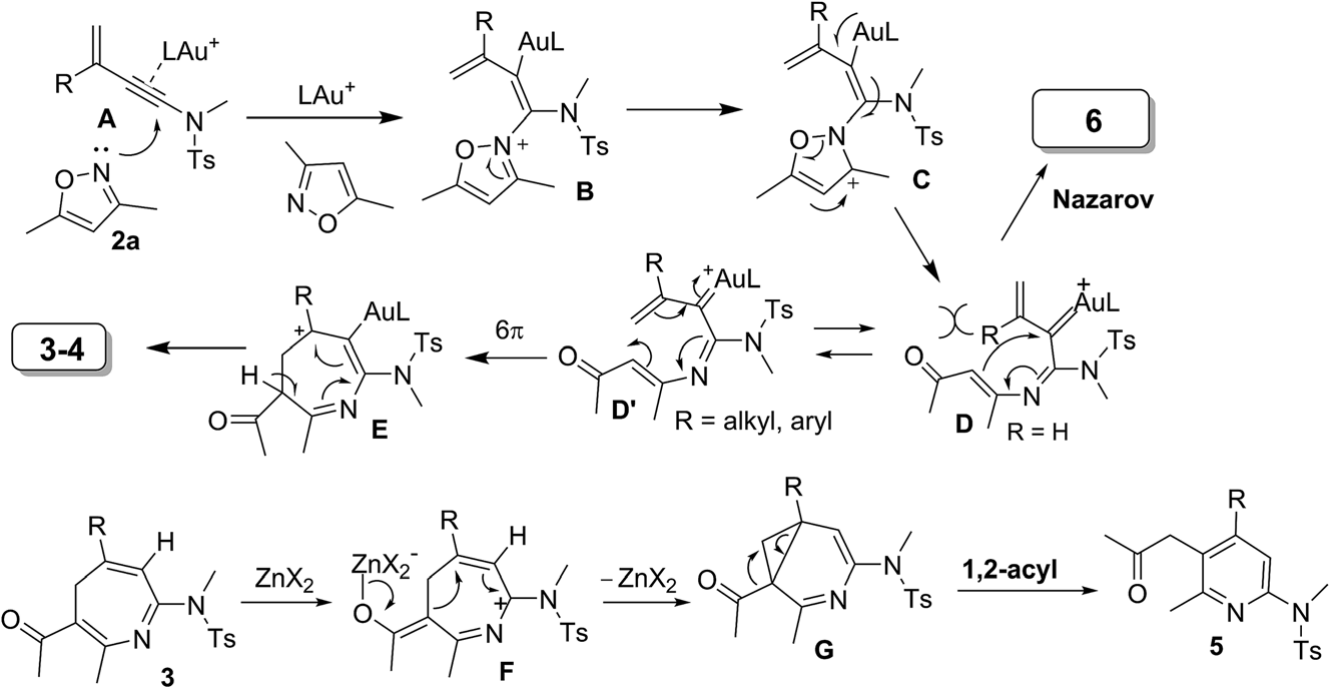
A plausible reaction mechanism.

Scheme 3 rationalizes the crucial roles of substituents of 3-en-1-ynamides in the chemoselectivity that relies on two conformational structures **D***versus***D′**. The N-attack of isoxazole at gold-π-ynamide **A** is expected to form a gold-carbene **D′**, which can be visualized as a gold-stabilized cycloheptatrienyl cation. Conformation **D** is favorable with R = H, which prefers aza-Nazarov reactions.^12^ When a C(3)-substituent is present (R = alkyl and aryl), all σ-*cis* configured species **D′** are the preferable geometry to induce novel 6π electrocyclizations. This ring closure is expected to proceed through an attack of enamide at the alkenylgold moiety that is also visualized as a gold-stabilized cation. Additional C(4)-substituents render the formation of cations **D′** difficult, thus yielding pyrrole **6** as byproducts. A loss of an acidic proton from seven-membered cations **E** is expected to yield azepines **3–4**. 4*H*-Azepines **3–4** bear an enone conjugated with a triene; this extensive conjugation is very stable to impede a 6π electrocyclization of their triene moieties unless a Lewis acid is present. Zn(OTf)_2_ likely coordinates with the carbonyl of 4*H*-azepine **3** to generate a 2-azapentadienyl cation **F** bearing a zinc enolate, further enabling an intramolecular cyclization to generate species **G**. A 1,2-acyl shift^14^ of species **G** delivers the observed product **5**.^13^

## Conclusions

In summary, this work describes new gold-catalyzed [4+3] annulations^15^ of 3-substituted 3-en-1-ynamides with isoxazoles to form 4*H*-azepines. A relay catalysis is also developed with Au(i)/Zn(ii) catalysts to achieve [4+2] annulations from the same reactants. The mechanisms of gold-catalyzed [4+3] annulations involve unprecedented 6π electrocyclizations of 3-azacycloheptatrienyl cations to form 4*H*-azepines **3–4** efficiently. Control experiments confirm that 4*H*-azepines **3–4** are catalyzed by Zn(OTf)_2_ to undergo new rearrangement reactions to form substituted pyridine derivatives.

## Conflicts of interest

The authors declare no conflict of interest.

## Supplementary Material

Supplementary information

Crystal structure data
