# Supplementary material for: Gold-catalyzed [4+3]- and [4+2]-annulations of 3-en-1-ynamides with isoxazoles via novel 6π-electrocyclizations of 3-azahepta trienyl cations
Source: Chem Sci. 2018 Feb 19;9(11):2991–5. doi: 10.1039/c8sc00232k (PMC5915799; doi:10.1039/c8sc00232k)
Supplement: Supplementary file 1 [file SC-009-C8SC00232K-s001.pdf]

Electronic Supplementary Material (ESI) for Chemical Science.
This journal is © The Royal Society of Chemistry 2018

Supporting Information

Gold-catalyzed [4+3] and [4+2]-Annulations of 3-En-1-ynamides with Isoxazoles *via* Novel 6π Electrocyclizations of 3-Azaheptatrienyl Cations

Sovan Sundar Giri and Rai-Shung Liu*

Department of Chemistry, National Tsing-Hua University, Hsinchu, Taiwan, 30013, ROC

E-mail: *rsliu@mx.nthu.edu.tw*

Contents:

(1) Representative Synthetic Procedures: --2

(2) Standard procedures for catalytic operations: --------------------------------------4

(3) Synthetic procedure for chemical functionalization: -------------------------------5

(4) References: ---6

(5) Spectral data for key compounds: ---7

(6) X-ray crystallographic data of 3b, 3l, 4a, 5a, 5i & 6m: ---------------------------26

(7) ^{1}H NOE map of compound 7c: ---65

(8) Spectral data ^{1}H, ^{13}C, NOE: --65

(1) Representative Synthetic Procedures:

(a) General procedure.

Unless otherwise noted, all reactions were performed in oven-dried glassware under nitrogen atmosphere with freshly distilled solvents. The catalytic reactions were performed under nitrogen atmosphere. Toluene and DCE were distilled from CaH_2 under nitrogen. THF were distilled from Na metal under nitrogen. All other commercial reagents were used without further purification, unless otherwise indicated. Isoxazole, **2b** (Cas no 288-14-2) and 5-methylisoxazole, **2d** (Cas no 5765-44-6) were procured from Alfa Aesar and TCI respectively. ^{1}H and ^{13}C NMR spectra were recorded using Mercury-400 MHz, Bruker 400 MHz and Varian-600 MHz spectrometers with chloroform-*d* ($CDCl_3$) solvent as internal standard. All the substrate 3-En-1-ynamides and isoxazole were prepared according to the literature procedures which are described below.

(b) Synthesis of 3-methylbut-3-en-1-ynamide (1a-1f).

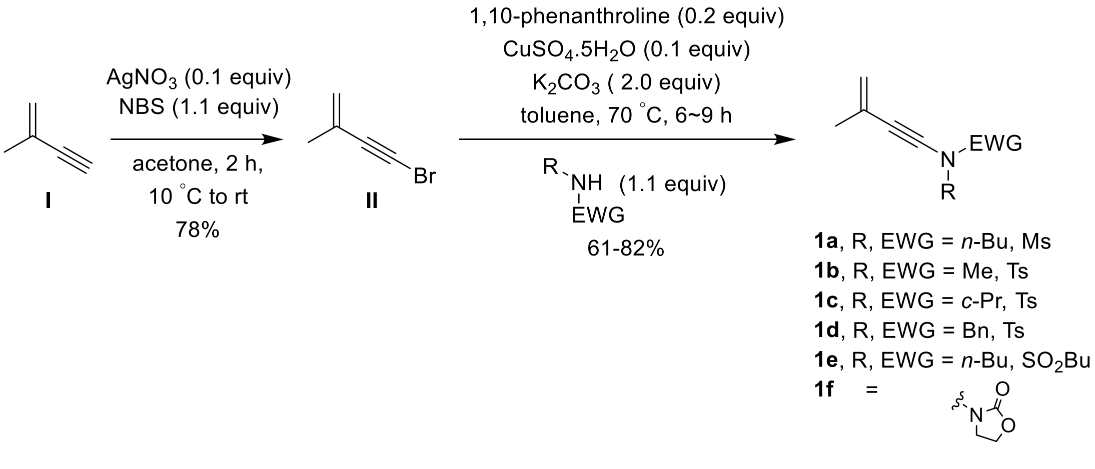

Synthesis of 4-bromo-2-methylbut-1-en-3-yne (II).

To a stirred solution of NBS (5.7 g, 32.1 mmol) and $AgNO_3$ (475 mg, 2.8 mmol) in dry acetone (30 mL), 2-methylbut-1-en-3-yne **I** (1.9 g, 29.1 mmol) was added dropwise at 10 °C under nitrogen and the resulting mixture was stirred allowing it slowly to attain room temperature over the period of 2 hrs. After completion of reaction, acetone was evaporated and pentane (40 mL) was added in to reaction mixture which was further stirred for 10 minutes at room temperature. Then it was filtered to remove brown solid and resulting reaction mixture was concentrated to afford 4-bromo-2-methylbut-1-en-3-yne, **II** (3.3 g, 22.7 mmol, 78%) as dark brown oil. It was used in the next step without further purification.

3-Methylbut-3-en-1-ynamide (1a-1f).

To a sealed tube was added *N*-alkyl sulfonamide (24.9 mmol), $CuSO_4 \cdot 5H_2O$ (570 mg, 2.3 mmol), 1,10-phenanthroline (800 mg, 4.4 mmol) and K_2CO_3 (6.2 g, 44.9 mmol), and this mixture was subsequently treated with toluene (40 mL) and 4-bromo-2-methylbut-1-en-3-yne, **II** (3.3 g, 22.7 mmol). The resulting mixture was heated at 70 °C for 6~9 h; the resulting solution was cooled to room temperature, filtered through a small celite bed, and concentrated. Purification of the crude residues was conducted with silica flash column chromatography to afford desired 3-methylbut-3-en-1-ynamide **1a-1f** with 61-82% yield. All the spectroscopic data matches with literature report.[1,2].

(c) Synthesis of *N*-butyl-*N*-(3-methylenehept-1-yn-1-yl)methanesulfonamide (1j).[1a]

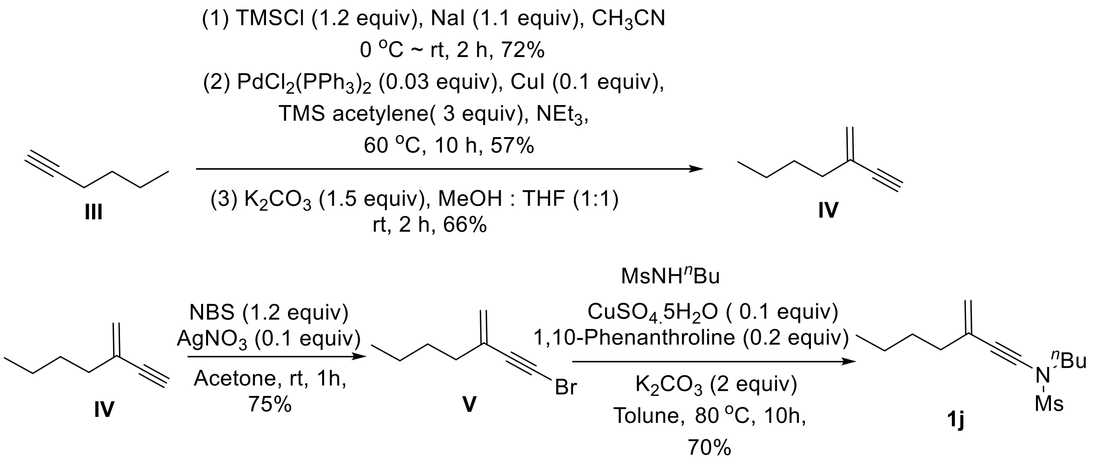

The synthesis of 3-methylenehept-1-yne **IV** intermediate followed literature procedure.[2] Bromination followed by the coupling of 3-methylenehept-1-yne **IV** with HN(*n*-Bu)Ms as described above in (b).

Other substrates **1g-1i** & **1k** were synthesized using the same reaction procedure as **1j.** Due to slow instability of **1i,** crude product of **1i** was eluted through a short silica column and used directly for the catalytic annulations.

Substrate **1l** and **1m** were synthesized according to the literature procedure.[3]

(d) Synthesis of 3,5-disubstituted isoxazoles.

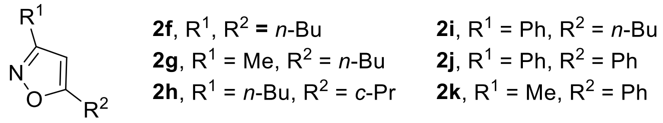

All 3,5-disubstituted isoxazoles, **2f-2k,** were synthesized according to the procedures from our recently published literatures.[4a-e] Unsubstitued isoxazole **2b** and 5-methylisoxazole **2d** were obtained commercially. 3-Methylisoxazole **2c** were prepared according to the known procedure.[5]

(2) Standard procedures for catalytic operations:

(a) Typical procedure for the synthesis of *N*-(3-acetyl-2,5-dimethyl-4*H*-azepin-7-yl)-*N*-butylmethanesulfonamide (3a).

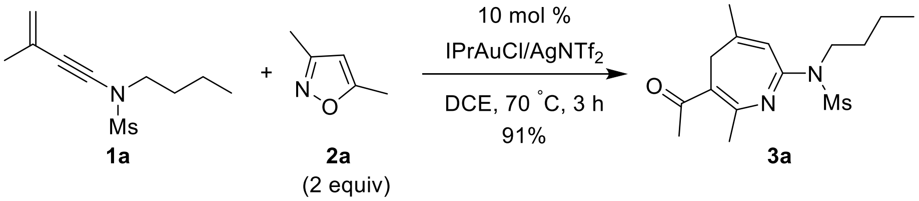

A suspension of IPrAuCl (0.029 g, 0.046 mmol) and $AgNTf_2$ (0.018 g, 0.046 mmol) in dry DCE (1 mL) was fitted with a N_2 balloon, and to this suspension was added a DCE (2 mL) solution of *N*-butyl-*N*-(3-methylbut-3-en-1-yn-1-yl)methanesulfonamide **1a** (0.1 g, 0.46 mmol) and 3,5-dimethylisoxazole **2a** (0.090 g, 0.93 mmol) at room temperature. The resulting mixture was stirred for 3 h at 70 °C before filtration over a short celite bed. The solvent was evaporated under reduced pressure, and eluted through a silica column with ethyl acetate/hexane (15:85) to afford *N*-(3-acetyl-2,5-dimethyl-4*H*-azepin-7-yl)-*N*-butylmethanesulfonamide **3a** (0.132 g, 0.42 mmol, 91%) as yellow oil.

(b) Typical procedure for the synthesis of *N*-(4,6-dimethyl-5-(2-oxopropyl)pyridin-2-yl)-*N*,4-dimethylbenzenesulfonamide (5a).

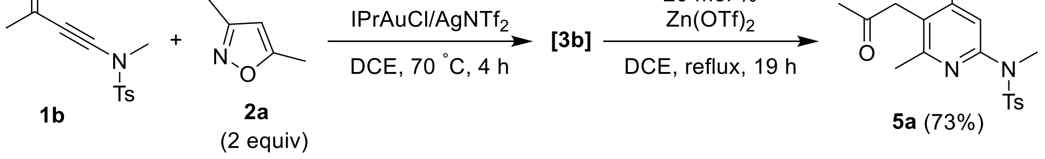

A single neck tube was charged with IPrAuCl (0.025 g, 0.040 mmol) and $AgNTf_2$ (0.015 g, 0.040 mmol), and to this mixture was added dry DCE (1 mL). The resulting mixture was stirred at room temperature for 10 min. To this mixture was added a dry DCE (2 mL) solution of *N*,4-dimethyl-*N*-(3-methylbut-3-en-1-yn-1-yl)benzenesulfonamide **1b** (0.1 g, 0.40 mmol) and 3,5-dimethylisoxazole **2a** (0.078 g, 0.0.80 mmol) dropwise. After stirring at 70 °C for 4 hours, the reaction mixture was filtered over a short celite bed. To this solution was added $Zn(OTf)_2$ (0.029 g, 0.081 mmol), and the mixture was refluxed for 19 hours. The resulting mixture was filtered through a short celite bed, concentrated, and eluted through a silica column (EA/hexane = 1/4) to afford the desired *N*-(4,6-dimethyl-5-(2-oxopropyl)pyridin-2-yl)-*N*,4-dimethylbenzenesulfonamide **5a** (0.101 g, 0.29 mmol, 73%) as white solid.

(3) Synthetic procedure for chemical functionalization:

(a) Synthesis of *N*-(3-(1-hydroxyethyl)-2, 5-dimethyl-4*H*-azepin-7-yl)-*N*, 4-dimethyl benzene sulfonamide (7a).

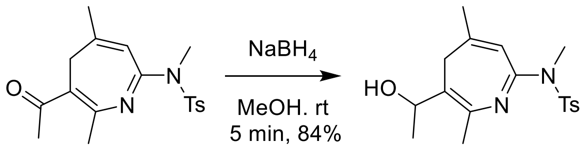

3b **7a**

To a solution of *N*-(3-acetyl-2,5-dimethyl-4*H*-azepin-7-yl)-*N*,4-dimethylbenzenesulfonamide **3b** (0.1 g, 0.29 mmol) in MeOH (2 mL) was added $NaBH_4$ (0.013 g, 0.35 mmol) slowly at room temperature. After a complete consumption of starting **3a**, the reaction mixture was treated with cold water (5 ml) and extracted with dichloromethane (5 mL x 3). The organic layer was washed with brine (10 mL), dried over $MgSO_4$, and concentrated under reduced pressure. The crude product was purified by flash chromatography on a silica column (EA/Hexane = 1:4) to afford *N*-(3-(1-hydroxyethyl)-2,5-dimethyl-4*H*-azepin-7-yl)-*N*,4-dimethylbenzenesulfonamide **7a** (0.084 g, 0.24 mmol, 84%) as a white solid.

(b) Synthesis of *N*-(6-acetyl-4,7-dimethyl-4,5-dihydro-3*H*-azepin-2-yl)-*N*,4-dimethylbenzene sulfonamide (7b).

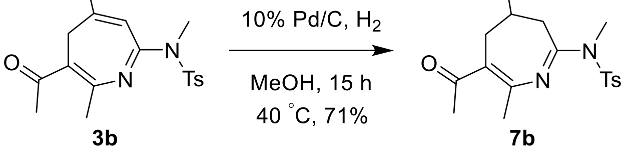

3b **7b**

To a MeOH solution (2 mL) of *N*-(3-acetyl-2,5-dimethyl-4*H*-azepin-7-yl)-*N*,4-dimethylbenzenesulfonamide **3b** (0.1 g, 0.29 mmol) was added 10% Pd/C (0.010 g); the resulting mixture was stirred at 40 °C for 15 h under a H_2 balloon. The reaction was monitored by ^{1}H NMR to ensure a complete conversion; the solution was filtered through a celite bed and evaporated under reduced pressure. The crude product was purified by flash chromatography on a silica column (EA/Hexane = 1:9) to afford *N*-(6-acetyl-4,7-dimethyl-4,5-dihydro-3*H*-azepin-2-yl)-*N*,4-dimethylbenzenesulfonamide **7b** (0.071 g, 0.20 mmol, 71%) as a viscous oil.

(c) Synthesis of (*Z*)-*N*-(3-acetyl-2-(bromomethylene)-5-methyl -2*H*-azepin-7-yl)-*N*,4-dimethylbenzenesulfonamide (7c).

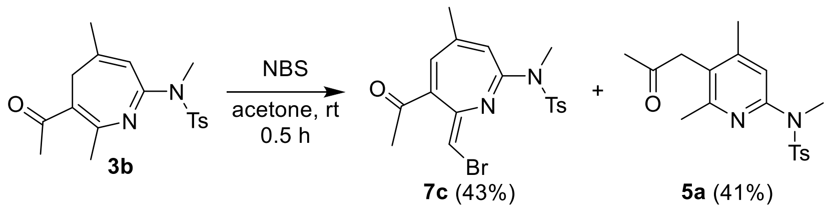

To an acetone solution (2 mL) of *N*-(3-acetyl-2,5-dimethyl-4*H*-azepin-7-yl)-*N*,4-dimethylbenzenesulfonamide **3b** (0.1 g, 0.29 mmol) was added NBS (0.062 g, 0.34 mmol); the mixture was stirred at room temperature for 30 minutes. The resulting mixture was filtered through a celite bed and evaporated under reduced pressure. The crude product was purified by flash chromatography on a silica gel column (EA/Hexane = 1.5:8.5) to afford (*Z*)-*N*-(3-acetyl-2-(bromomethylene)-5-methyl-2*H*-azepin-7-yl)-*N*,4-dimethylbenzenesulfonamide **7c** (0.052 g, 0.12 mmol, 43%) as a yellow oil.

Along with **7c,** rearrangement product **5a** was also isolated with 41% yield.

(4) References:

[1] a) S. S. Giri, L. H. Lin, P. D. Jadhav and R. S. Liu, *Adv. Synth. Catal*. 2017, **359**, 590; b) K. Jouvin, J.Heimburger and G. Evano, *Chem. Sci.*, 2012, **3**, 756.

[2] a) A. Jadhav, V. B. Pagar, D. B. Huple and R. S. Liu, *Angew. Chem. Int. Ed*. 2015, **54**, 3812; b) R. R. Singh and R. S. Liu, *Adv. Synth. Catal.* 2016, **358**, 1421.

[3] a) R. B. Dateer, K. K. Pati and R. S. Liu, *Chem. Commun.*, 2012, **48**, 7200; b) Y. Kim, R. B. Dateer and S. Chang, *Org. Lett.* 2017, **19**, 190.

[4] a) R. L. Sahani and R. S. Liu, *Angew. Chem. Int. Ed.* 2017, **56**, 1026; b) T. V. Hansen, P. Wu and V. V. fokin, *J. Org. Chem*. 2005, **70**, 7761; c) K. K.Y. Kung, V. K. Y. Lo, H. M. Ko, G. L.

Li, P. Chan, K. Leung, Z. Zhou, M. Wang, C. M. Che and M. K. Wonga, *Adv. Synth. Catal.* 2013, **355**, 2055; d) J. C. Jeyaveerana, C. Praveen, Y. Arun, A. A. Prince and P. T. Perumal, *J. Chem. Sci.* 2016, **128**, 73; e) C. Praveen, A. Kalyanasundaram and P. T. Perumal, *Synlett* 2010**,** **5**, 777.

[5] A. G. Griesbeck, M. Franke, J. Neudörfl and H. Kotaka, *Beilstein J. Org. Chem.* 2011, **7**, 127.

(5) Spectral data for key compounds:

Spectral data for *N*-cyclopropyl-4-methyl-*N*-(3-methylbut-3-en-1-yn-1-yl) benzene sulfonamide (1c).

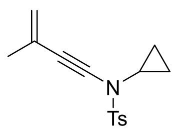

(**1c**)

Yellow oil (3.42 g, 12.4 mmol, 82%); ^{1}H NMR (400 MHz, $CDCl_3$): δ 7.80 (dt, *J* = 8.3 Hz, 1.9 Hz, 2H), 7.33 (dt, *J* = 7.9 Hz, 1.9 Hz, 2H), 5.13 ~ 5.11 (m, 1H), 5.10 ~ 5.08 (m, 1H), 2.78 ~ 2.72 (m, 1H), 2.43 (s, 3H), 1.84 (dd, *J* = 1.4 Hz, 1.1 Hz, 3H), 0.83 ~ 0.77 (m, 2H), 0.76 ~ 0.69 (m, 2H); ^{13}C NMR (100 MHz, $CDCl_3$): δ 144.7, 133.8, 129.6, 127.9, 126.0, 119.6, 81.1, 72.1, 32.7, 23.5, 21.6, 6.3; ESI-MS calcd for $C_{15}H_{18}NO_2S$[M+H]: 276.1058, found: 276.1053.

Spectral data for *N*-benzyl-4-methyl-*N*-(3-methylbut-3 -en-1-yn-1-yl) benzenesulfonamide (1d).

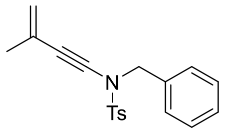

(**1d**)

Brown solid, mp: 60-61 °C (2.15 g, 6.61 mmol, 68%); ^{1}H NMR (400 MHz, $CDCl_3$): δ 7.74 (d, *J* = 8.3 Hz, 2H), 7.30 ~ 7.24 (m, 7H), 5.03 ~ 5.02 (m, 2H), 4.49 (s, 2H), 2.42 (s, 3H), 1.75 (t, *J* = 1.2 Hz, 3H); ^{13}C NMR (100 MHz, $CDCl_3$): δ 144.5, 134.5, 134.3, 129.6, 128.8, 128.4, 128.2, 127.6, 125.9, 119.1, 82.0, 72.9, 55.5, 23.2, 21.5; ESI-MS calcd for $C_{19}H_{20}NO_2S$[M+H]: 326.1215, found: 326.1219.

Spectral data for *N*-butyl-*N*-(3-methylbut-3-en-1-yn-1-yl)butane-1-sulfonamide (1e).

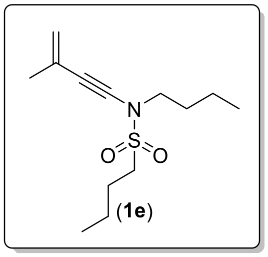

Yellow oil (0.715 g, 2.78 mmol, 61%); ^{1}H NMR (400 MHz, $CDCl_3$): δ 5.15 ~ 5.14 (m, 1H), 5.11 ~ 5.10 (m, 1H), 3.43 (t, *J* = 7.2 Hz, 2H), 3.21 ~ 3.17 (m, 2H), 1.86 (t, *J* = 1.4 Hz, 3H), 1.85 ~ 1.77 (m, 2H), 1.71 ~ 1.60 (m, 2H), 1.50 ~ 1.42 (m, 2H), 1.41 ~ 1.33 (m, 2H), 0.95 ~ 0.90 (m, 6H); ^{13}C NMR (100 MHz, $CDCl_3$): δ 126.1, 119.5, 81.4, 72.1, 51.4, 51.3, 30.5, 25.1, 23.5, 21.4, 19.4, 13.5, 13.4; ESI-MS calcd for $C_{13}H_{24}NO_2S$[M+H]: 258.1528, found: 258.1516.

Spectral data for *N*,4-dimethyl-*N*-(4-methyl-3-methylenepent-1-yn-1-yl)benzene sulfonamide (1g).

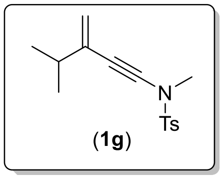

Yellow oil (2.08 g, 7.51 mmol, 52%); ^{1}H NMR (400 MHz, $CDCl_3$): δ 7.77 (d, *J* = 8.3 Hz, 2H), 7.33 (d, *J* = 8.0 Hz, 2H), 5.11 (t, *J* = 1.6 Hz, 1H), 5.09 (d, *J* = 1.7 Hz, 1H), 3.07 (s, 3H), 2.43 (s, 3H), 2.40 ~ 2.32 (m, 1H), 1.04 (d, *J* = 6.8 Hz, 6H); ^{13}C NMR (100 MHz, $CDCl_3$): δ 144.7, 137.4, 133.2, 129.7, 127.7, 116.6, 84.5, 68.6, 39.3, 35.4, 21.5; ESI-MS calcd for $C_{15}H_{20}NO_2S$[M+H]: 278.1215, found: 278.1205.

Spectral data for *N*-(3-cyclopropylbut-3-en-1-yn-1-yl)-*N*,4-dimethyl benzene sulfonamide (1h).

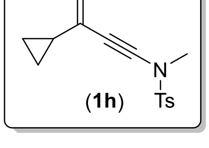

Yellow oil (2.90 g, 10.5 mmol, 64%); ^{1}H NMR (400 MHz, $CDCl_3$): δ 7.74 (d, *J* = 8.3 Hz, 2H), 7.33 (d, *J* = 8.2 Hz, 2H), 5.23 (d, *J* = 1.6 Hz, 1H), 5.11 (d, *J* = 1.7 Hz, 1H), 3.04 (s, 3H), 2.43 (s, 3H), 1.55 ~ 1.48 (m, 1H), 0.66 ~ 0.61 (m, 4H); ^{13}C NMR (100 MHz, $CDCl_3$): δ 144.8, 133.1,

129.8, 127.7, 117.2, 83.9, 66.4, 39.2, 21.6, 16.6, 5.7; ESI-MS calcd for $C_{15}H_{18}NO_2S$[M+H]: 276.1058, found: 276.1047.

Spectral data for *N*,4-dimethyl-*N*-(3-methylenehept-1-yn-1-yl)benzenesulfonamide (1k).

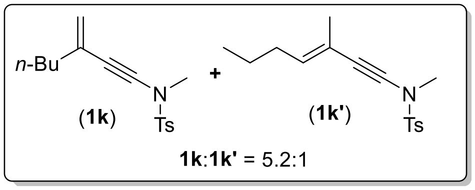

Yellow oil (5.72 g, 0.19 mmol, 75%); ^{1}H NMR for major isomer (400 MHz, $CDCl_3$): δ 7.79 ~ 7.75 (m, 2H), 7.33 (dd, *J* = 8.5 Hz, 0.7 Hz, 2H), 5.14 (t, *J* = 1.0 Hz, 1H), 5.09 ~ 5.08 (m, 1H), 3.06 (s, 3H), 2.43 (s, 3H), 2.12 ~ 2.08 (m, 2H), 1.47 ~ 1.39 (m, 2H), 1.36 ~ 1.23 (m, 2H), 0.90 ~ 0.85 (m, 3H), ^{1}H NMR for minor isomer: δ 5.57 ~ 5.52 (m, 1H), 3.07 (s, 3H), 1.78 (t, *J* = 1.1 Hz, 2H), rest of the peaks merged with others; ^{13}C NMR for major isomer (100 MHz, $CDCl_3$): δ 144.7, 133.2, 131.0, 129.7, 127.8, 119.0, 83.8, 69.8, 39.3, 37.0, 30.2, 21.9, 21.6, 13.8, ^{13}C NMR for minor isomer: δ 136.3, 117.0, 87.0, 68.6, 39.4, 32.6, 23.0, 22.4, 22.3, 13.7, rest of the peaks merged with others; ESI-MS calcd for $C_{16}H_{22}NO_2S$[M+H]: 292.1371, found: 292.1359.

Spectral data for 3-butyl-5-cyclopropylisoxazole (2h).

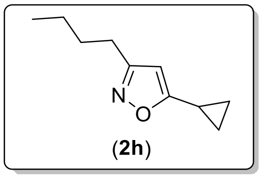

Colorless oil (2.10 g, 12.7 mmol, 44%); ^{1}H NMR (400 MHz, $CDCl_3$): δ 5.69 (s, 1H), 2.53 (t, *J* = 7.7 Hz, 2H), 1.96 ~ 1.90 (m, 1H), 1.59 ~ 1.52 (m, 2H), 1.39 ~ 1.25 (m, 2H), 0.99 ~ 0.92 (m, 2H), 0.91 ~ 0.81 (m, 5H); ^{13}C NMR (100 MHz, $CDCl_3$): δ 174.1, 164.0, 98.2, 30.3, 25.6, 22.2, 13.6, 8.0, 7.9; ESI-MS calcd for $C_{10}H_{16}NO$[M+H]: 166.1232, found: 166.1224.

Spectral data for *N*-(3-acetyl-2,5-dimethyl-4*H*-azepin-7-yl)-*N*-butyl methane sulfonamide (3a).

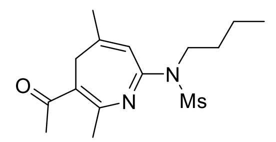

(3a)

Yellow oil (0.132 g, 0.42 mmol, 91%); ^{1}H NMR (400 MHz, $CDCl_3$): δ 5.87 (q, *J* = 1.2 Hz, 1H), 3.77 (t, *J* = 7.5 Hz, 2H), 3.19 (s, 3H), 2.55 (br, 2H), 2.37 (s, 3H), 2.19 (s, 3H), 2.06 (d, *J* = 1.2 Hz, 3H), 1.63 ~ 1.55 (m, 2H), 1.36 ~ 1.26 (m, 2H), 0.90 (t, *J* = 7.3 Hz, 3H); ^{13}C NMR (100 MHz, $CDCl_3$): δ 198.6, 153.3, 152.0, 150.4, 123.3, 114.6, 47.1, 42.7, 32.9, 31.4, 30.5, 23.4, 21.7, 19.8, 13.6; ESI-MS calcd for $C_{15}H_{25}N_2O_3S$[M+H]: 313.1586, found: 313.1593.

Spectral data for *N*-(3-acetyl-2,5-dimethyl-4*H*-azepin-7-yl)-*N*,4-dimethyl benzene sulfonamide (3b).

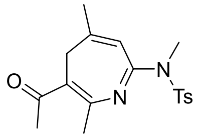

(3b)

White solid, mp: 94-95 °C (0.117 g, 0.34 mmol, 84%); ^{1}H NMR (600 MHz, $CDCl_3$): δ 7.53 (d, *J* = 8.3 Hz, 2H), 7.22 (d, *J* = 8.0 Hz, 2H), 6.09 (d, *J* = 1.3 Hz, 1H), 3.24 (s, 3H), 2.84 (br, 1H), 2.35 (s, 3H), 2.30 (s, 3H), 2.07 (s, 3H), 2.01 (d, *J* = 1.3 Hz, 3H), 1.37 (br, 1H); ^{13}C NMR (150 MHz, $CDCl_3$): δ 199.1, 152.9, 150.2, 149.9, 144.3, 134.4, 129.4, 127.0, 123.7, 115.7, 34.5, 32.2, 30.7, 23.4, 22.4, 21.6; ESI-MS calcd for $C_{18}H_{23}N_2O_3S$[M+H]: 347.1429, found: 347.1423.

Spectral data for *N*-(3-acetyl-2,5-dimethyl-4*H*-azepin-7-yl)-*N*-cyclopropyl-4-methyl benzenesulfonamide (3c).

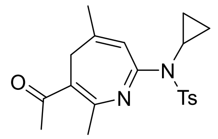

(3c)

Yellow oil (0.116 g, 0.31 mmol, 86%); ^{1}H NMR (400 MHz, $CDCl_3$): δ 7.69 (d, *J* = 8.2 Hz, 2H), 7.23 (d, *J* = 8.4 Hz, 2H), 5.90 (d, *J* = 1.1 Hz, 1H), 2.61 ~ 2.56 (m, 1H), 2.36 (s, 4H), 2.30 (s, 4H), 2.07 (s, 3H), 2.02 (s, 3H), 0.92 (d, *J* = 6.6 Hz, 2H), 0.78 (br, 2H); ^{13}C NMR (100 MHz, $CDCl_3$):

δ 198.9, 154.9, 150.5, 149.8, 144.0, 136.2, 129.3, 127.8, 123.8, 117.8, 32.9, 30.4, 29.5, 23.4, 21.8, 21.5, 9.5; ESI-MS calcd for $C_{20}H_{25}N_2O_3S$[M+H]: 373.1586, found: 373.1575.

Spectral data for *N*-(3-acetyl -2,5-dimethyl -4*H*-azepin-7-yl)-*N*-benzyl -4-methyl benzene sulfonamide (3d).

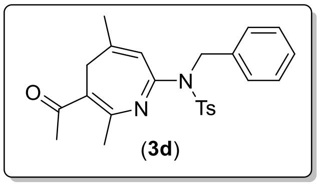

Brown solid, (0.114 g, 0.27 mmol, 87%); ^{1}H NMR (600 MHz, $CDCl_3$, -47.15 °C): δ 7.54 (d, *J* = 8.3 Hz, 2H), 7.39 (d, *J* = 7.5 Hz, 2H), 7.29 (t, *J* = 7.4 Hz, 2H), 7.24 ~ 7.21 (m, 3H), 6.03 (s, 1H), 5.10 (d, *J* = 14.5 Hz, 1H), 4.86 (d, *J* = 14.6 Hz, 1H), 2.84 (d, *J* = 12.9 Hz, 1H), 2.37 (s, 3H), 2.27 (s, 3H), 1.97 (s, 3H), 1.82 (s, 3H), 1.35 (d, *J* = 13.0 Hz, 1H); ^{13}C NMR (150 MHz, $CDCl_3$, -42.35 °C): δ 199.4, 151.1, 150.1, 144.2, 137.1, 135.4, 129.4, 128.2, 127.8, 127.1, 123.5, 116.1, 49.5, 32.2, 30.7, 23.4, 21.8, 21.6, two quaternary merged at δ 150.1 and two CH peaks merged at δ 127.1; ESI-MS calcd for $C_{24}H_{27}N_2O_3S$[M+H]: 423.1742, found: 423.1733.

Spectral data for *N*-(3-acetyl-2,5-dimethyl-4*H*-azepin-7-yl)-*N*-butylbutane-1-sulfonamide (3e).

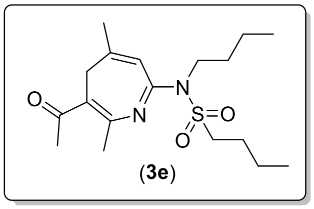

Yellow oil (0.124 g, 0.35 mmol, 90%); ^{1}H NMR (400 MHz, $CDCl_3$): δ 5.90 (d, *J* = 1.4 Hz, 1H), 3.74 (t, *J* = 7.5 Hz, 2H), 3.27 (dd, *J* = 10.7 Hz, 7.9 Hz, 2H), 2.49 (br, 2H), 2.33 (s, 3H), 2.15 (s, 3H), 2.01 (d, *J* = 1.3 Hz, 3H), 1.73 ~ 1.65 (m, 2H), 1.62 ~ 1.54 (m, 2H), 1.42 ~ 1.33 (m, 2H), 1.32 ~ 1.24 (m, 2H), 0.89 ~ 0.84 (m, 6H); ^{13}C NMR (100 MHz, $CDCl_3$): δ 198.5, 153.0, 151.2, 150.4, 123.1, 114.4, 54.5, 46.8, 32.8, 31.7, 30.5, 25.0, 23.3, 21.9, 21.3, 19.8, 13.6, 13.4; ESI-MS calcd for $C_{18}H_{31}N_2O_3S$[M+H]: 355.2055, found: 355.2047.

Spectral data for 3-(3-acetyl-2,5-dimethyl-4*H*-azepin-7-yl)oxazolidin-2-one (3f).

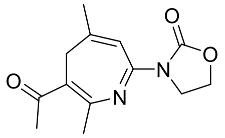

(**3f**)

Viscous oil (0.074 g, 0.30 mmol, 64%); ^{1}H NMR (400 MHz, $CDCl_3$): δ 6.38 (s, 1H), 4.38 (t, *J* = 8.0 Hz, 2H), 4.06 (t, *J* = 8.2 Hz, 2H), 2.51 (s, 2H), 2.32 (s, 3H), 2.13 (s, 3H), 2.04 (d, *J* = 1.0 Hz, 3H); ^{13}C NMR (100 MHz, $CDCl_3$): δ 198.7, 154.3, 151.6, 150.4, 150.1, 123.1, 113.7, 62.0, 43.7, 32.9, 30.4, 23.5, 22.6; ESI-MS calcd for $C_{13}H_{17}N_2O_3$[M+H]: 249.1239, found:249.1234.

Spectral data for *N*-(3-acetyl-5-isopropyl-2-methyl-4*H*-azepin-7-yl)-*N*,4-dimethyl benzene sulfonamide (3g).

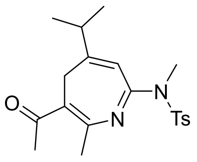

(**3g**)

Yellow oil (0.100 g, 0.27 mmol, 74%); ^{1}H NMR (600 MHz, $CDCl_3$, -14.05 $^{\circ}$C): δ 7.51 (d, *J* = 8.3 Hz, 2H), 7.20 (d, *J* = 8.2 Hz, 2H), 6.08 (s, 1H), 3.28 (s, 3H), 2.99 (d, *J* = 13.1 Hz, 1H), 2.45 ~ 2.39 (m, 1H), 2.34 (s, 3H), 2.30 (s, 3H), 209 (s, 3H), 1.21 (d, *J* = 13.2 Hz, 1H), 1.09 (d, *J* = 6.8 Hz, 3H), 1.05 (d, *J* = 6.7 Hz, 3H); ^{13}C NMR (150 MHz, $CDCl_3$, -14.85 $^{\circ}$C): δ 198.8, 159.8, 153.9, 150.6, 144.3, 134.4, 129.5, 126.9, 124.1, 112.8, 34.6, 34.2, 31.0, 29.5, 22.7, 21.6, 21.3, 20.4; EI-MS calcd for $C_{20}H_{26}N_2O_3S$[M^+]: 374.1664, found: 374.1665.

Spectral data for *N*-(3-acetyl-5-cyclopropyl-2-methyl-4*H*-azepin-7-yl)-*N*,4-dimethyl benzenesulfonamide (3h).

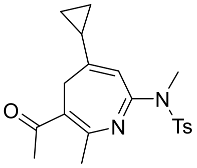

(**3h**)

White solid, mp: 96-97 $^{\circ}$C (0.107 g, 0.28 mmol, 79%); ^{1}H NMR (600 MHz, $CDCl_3$, -18.55 $^{\circ}$C): δ 7.53 (d, *J* = 8.3 Hz, 2H), 7.20 (d, *J* = 8.2 Hz, 2H), 6.14 (s, 1H), 3.26 (s, 3H), 2.69 (d, *J* = 13.4 Hz,

1H), 2.34 (s, 3H), 2.31 (s, 3H), 2.10 (s, 3H), 1.59 ~ 1.55 (m, 1H), 1.09 ~ 1.05 (m, 1H), 0.98 (d, *J* = 13.3 Hz, 1H), 0.89 ~ 0.85 (m, 1H), 0.81 ~ 0.72 (m, 1H), 0.63 ~ 0.59 (m, 1H); ^{13}C NMR (150 MHz, $CDCl_3$, -11.15 $^{\circ}$C): δ 198.6, 157.3, 153.1, 151.6, 144.3, 134.1, 129.4, 127.0, 124.0, 113.3, 34.6, 31.3, 26.5, 23.0, 21.6, 16.8, 7.9, 6.2; ESI-MS calcd for $C_{20}H_{25}N_2O_3S$[M+H]: 373.1586, found: 373.1574.

Spectral data for *N*-(3-acetyl-2-methyl-5-phenyl-4*H*-azepin-7-yl)-*N*,4-dimethyl benzene sulfonamide (3i).

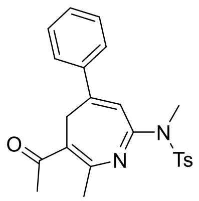

(**3i**)

Viscous oil (0.076 g, 0.19 mmol, 58%); ^{1}H NMR (600 MHz, $CDCl_3$): δ 7.71 (d, *J* = 8.4 Hz, 2H), 7.54 (d, *J* = 8.3 Hz, 2H), 7.43 ~ 7.37 (m, 3H), 7.21 (d, *J* = 8.2 Hz, 2H), 6.66 (s, 1H), 3.64 (d, *J* = 13.7 Hz, 1H), 3.33 (s, 3H), 2.36 (s, 3H), 2.19 (s, 3H), 2.17 (s, 3H), 1.53 (d, *J* = 13.7 Hz, 1H); ^{13}C NMR (150 MHz, $CDCl_3$): δ 198.7, 153.3, 151.4, 148.6, 144.5, 137.5, 134.3, 129.6, 129.4, 128.8, 127.1, 127.0, 124.3, 115.4, 34.7, 30.9, 29.5, 22.8, 21.7; EI-MS calcd for $C_{23}H_{24}N_2O_3S$[M^+]: 408.1508, found: 408.1510.

Spectral data for *N*-(3-acetyl-5-butyl-2-methyl-4*H*-azepin-7-yl)-*N*-butyl methane sulfonamide (3j).

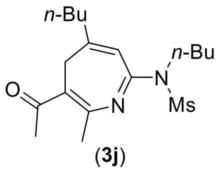

(**3j**)
isomer ratio= 5:1

Yellow oil (0.076 g, 0.21mmol, 55%); ^{1}H NMR for major isomer (400 MHz, $CDCl_3$): δ 5.83 (s, 1H), 3.78 ~ 3.73 (m, 2H), 3.19 (s, 3H), 2.56 (br, 1H), 2.35 (s, 3H), 2.29 (t, *J* = 7.3 Hz, 2H), 2.19 (s, 3H), 2.07 (d, *J* = 6.0 Hz, 1H), 1.66 ~ 1.56 (m, 2H), 1.54 ~ 1.45 (m, 2H), 1.40 ~ 1.24 (m, 4H), 0.90 ~ 0.83 (m, 6H), ^{1}H NMR for minor isomer: δ 5.96 (s, 1H), 3.17 (s, 3H), 2.32 (s, 3H), 1.26 ~ 1.18 (m, 4H), 0.80 ~ 0.76 (m, 6H), rest of the peaks merged with others; ^{13}C NMR for major

isomer (100 MHz, $CDCl_3$): δ 198.4, 156.6, 153.7, 150.7, 123.3, 113.6, 47.2, 42.8, 36.4, 31.5, 31.4, 30.6, 29.3, 22.2, 21.8, 19.9, 13.8, 13.6, ^{13}C NMR for minor isomer: δ 202.3, 156.3, 151.9, 146.3, 127.3, 114.3, 43.7, 42.7, 30.1, 26.3, 22.6, 21.0, 20.0, 13.9, rest of the peaks merged with others; ESI-MS calcd for $C_{18}H_{31}N_2O_3S$[M+H]: 355.2055, found: 355.2045.

Spectral data for *N*-(3-acetyl-5-butyl-2-methyl-4*H*-azepin-7-yl)-*N*,4-dimethyl benzene sulfonamide (3k).

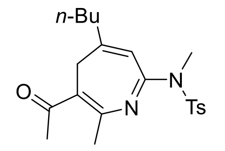

(**3k**)
isomeric ratio = 11.1:1

viscous oil (0.090 g, 0.23 mmol, 68%); ^{1}H NMR for major isomer (600 MHz, $CDCl_3$, -16.25 °C): δ 7.52 (d, *J* = 8.3 Hz, 2H), 7.21 (d, *J* = 8.4 Hz, 2H), 6.06 (s, 1H), 3.24 (s, 3H), 2.92 (d, *J* = 14.0 Hz, 1H), 2.35 (s, 3H), 2.27 (s, 3H), 2.23 (t, *J* = 7.9 Hz, 2H), 2.08 (s, 3H), 1.52 ~ 1.49 (m, 1H), 1.43 ~ 1.40 (m, 1H), 1.36 ~ 1.28 (m, 2H), 0.88 (t, *J* = 7.3 Hz, 3H), ^{1}H NMR for minor isomer: δ 7.62 (d, *J* = 8.3 Hz, 2H), 6.24 (d, *J* = 1.2 Hz, 1H), 3.29 (s, 3H), 2.24 (s, 3H), 2.02 (s, 3H), 1.95 (s, 3H), 0.59 (t, *J* = 7.2 Hz, 3H), rest of the peaks merged with others; ^{13}C NMR for major isomer (150 MHz, $CDCl_3$, -13.65 °C): δ 198.9, 154.1, 153.3, 150.4, 144.3, 134.3, 129.5, 126.9, 123.8, 114.4, 36.2, 34.6, 31.0, 30.8, 29.1, 22.6, 22.4, 21.6, 14.0, ^{13}C NMR for minor isomer: δ 203.4, 155.0, 152.1, 151.4, 146.1, 135.5, 129.3, 128.9, 128.8, 127.4, 43.6, 30.1, 28.0, 26.1, 23.3, 21.1, rest of the peaks merged with others; EI-MS calcd for $C_{21}H_{28}N_2O_3S[M^+]$: 388.1821, found: 388.1824.

Spectral data for *N*-(3-acetyl-2,5-dimethyl-4-phenyl-4*H*-azepin-7-yl)-*N*,4-dimethyl benzene sulfonamide (3l).

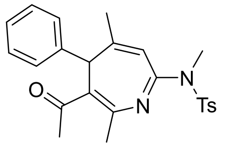

(**3l**)

White solid, mp: 156-157 °C (0.061 g, 0.15 mmol, 48%); ^{1}H NMR (400 MHz, $CDCl_3$): δ 7.14 ~ 7.09 (m, 4H), 7.08 ~ 7.00 (m, 3H), 6.89 (dd, *J* = 6.9 Hz, 1.2 Hz, 2H), 6.27 (t, *J* = 1.4 Hz, 1H),

4.63 (s, 1H), 2.76 (s, 3H), 2.42 (s, 3H), 2.38 (s, 3H), 2.31 (s, 3H), 2.18 (s, 3H); ^{13}C NMR (100 MHz, $CDCl_3$): δ 200.5, 154.2, 152.1, 149.2, 143.4, 138.3, 135.9, 129.3, 127.7, 127.4, 126.3, 126.2, 126.1, 116.0, 46.8, 34.3, 30.3, 26.4, 22.9, 21.5; ESI-MS calcd for $C_{24}H_{27}N_2O_3S$[M+H]: 423.1742, found: 423.1734.

Spectral data for *N*-(5-acetyl-4-methyl-5a,7,8,9-tetrahydro-6*H*-benzo[*d*]azepin-2-yl)-*N*,4-dimethylbenzenesulfonamide (3m).

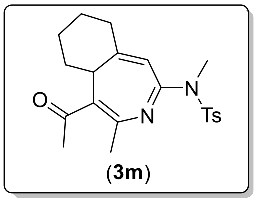

Viscous oil (0.021 g, 0.06 mmol, 16%); ^{1}H NMR (400 MHz, $CDCl_3$): δ 7.56 (d, *J* = 8.3 Hz, 2H), 7.21 (d, *J* = 8.0 Hz, 2H), 6.05 (s, 1H), 3.30 (s, 3H), 2.51 ~ 2.40 (m, 1H), 2.37 (s, 3H), 2.31 (s, 4H), 1.87 ~ 1.80 (m, 4H), 1.77 ~ 1.69 (m, 1H), 1.61 ~ 1.48 (m, 3H), 1.46 ~ 1.37 (m, 1H), 1.35 ~ 1.28 (m, 1H); ^{13}C NMR (100 MHz, $CDCl_3$): δ 204.6, 153.2, 151.8, 144.0, 138.9, 135.7, 129.4, 128.4, 127.3, 113.3, 37.9, 34.3, 32.6, 28.5, 23.7, 23.1, 21.5, 21.2, 20.5; ESI-MS calcd for $C_{21}H_{27}N_2O_3S$[M+H]: 387.1742, found: 387.1744.

Spectral data for *N*-(3-formyl-5-methyl-4*H*-azepin-7-yl)-*N*,4-dimethyl benzene sulfonamide (4a).

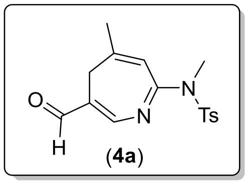

White solid, mp: 107-108 $^{\circ}$C (0.106 g, 0.33 mmol, 84%); ^{1}H NMR (400 MHz, $CDCl_3$): δ 9.34 (s, 1H), 7.59 (d, *J* = 8.2 Hz, 2H), 7.24 (d, *J* = 8.0 Hz, 3H), 6.20 (s, 1H), 3.32 (s, 3H), 2.37 (s, 3H), 2.27 (s, 2H), 1.94 (d, *J* = 1.1 Hz, 3H); ^{13}C NMR (100 MHz, $CDCl_3$): δ 189.8, 157.8, 151.5, 149.6, 144.5, 135.4, 129.5, 127.2, 116.7, 34.5, 26.6, 24.1, 21.5, one quaternary peak merged with others; EI-MS calcd for $C_{16}H_{18}N_2O_3S[M^+]$: 318.1038, found: 318.1035.

Spectral data for *N*-(3-acetyl-5-methyl-4*H*-azepin-7-yl)-*N*,4-dimethyl benzene sulfonamide (4b).

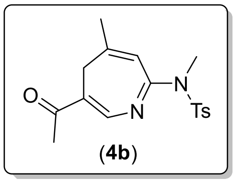

White solid, mp: 108-109 °C (0.099 g, 0.30 mmol, 75%); ^{1}H NMR (400 MHz, $CDCl_3$): δ 7.58 (d, *J* = 8.3 Hz, 2H), 7.51 (s, 1H), 7.23 (d, *J* = 8.4 Hz, 2H), 6.18 (t, *J* = 1.1 Hz, 1H), 3.29 (s, 3H), 2.36 (s, 3H), 2.27 (s, 5H), 1.94 (d, *J* = 1.1 Hz, 3H); ^{13}C NMR (100 MHz, $CDCl_3$): δ 195.7, 157.4, 149.7, 144.4, 143.7, 135.4, 129.5, 127.3, 126.2, 116.4, 34.5, 28.3, 25.7, 23.7, 21.5; EI-MS calcd for $C_{17}H_{20}N_2O_3S[M^+]$: 332.1195, found: 332.1196.

Spectral data for *N*-(3-formyl-2,5-dimethyl-4*H*-azepin-7-yl)-*N*,4-dimethyl benzene sulfonamide (4c).

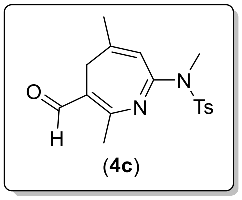

White solid, mp: 103-104 °C (0.116 g, 0.34 mmol, 87%); ^{1}H NMR (600 MHz, $CDCl_3$, -20.85 °C): δ 9.62 (s, 1H), 7.46 (d, *J* = 8.3 Hz, 2H), 7.15 (d, *J* = 8.1 Hz, 2H), 6.01 (s, 1H), 3.19 (s, 3H), 2.97 (d, *J* = 12.7 Hz, 1H), 2.27 (s, 3H), 2.06 (s, 3H), 1.83 (s, 3H), 1.06 (d, *J* = 12.4 Hz, 1H); ^{13}C NMR (150 MHz, $CDCl_3$, -15.05 °C): δ 189.0, 158.2, 154.9, 151.2, 144.5, 134.2, 129.4, 126.9, 122.7, 115.5, 34.5, 26.9, 23.7, 21.6, 18.1; ESI-MS calcd for $C_{17}H_{21}N_2O_3S[M+H]$: 333.1273, found: 333.1269.

Spectral data for *N*-(2-ethyl-5-methyl-3-propionyl-4*H*-azepin-7-yl)-*N*,4-dimethyl benzene sulfonamide (4d).

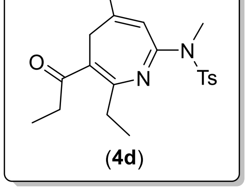

Colorless oil (0.128 g, 0.34 mmol, 85%); ^{1}H NMR (400 MHz, $CDCl_3$): δ 7.56 (d, *J* = 8.3 Hz, 2H), 7.21 (d, *J* = 8.4 Hz, 2H), 6.12 (d, *J* = 1.4 Hz, 1H), 3.26 (s, 3H), 2.56 (q, *J* = 7.4 Hz, 2H), 2.40 ~

2.33 (m, 5H), 2.12 (br, 2H), 2.02 (d, *J* = 1.2 Hz, 3H), 1.05 (t, *J* = 7.1 Hz, 3H), 0.95 (t, *J* = 7.4 Hz, 3H); ^{13}C NMR (100 MHz, $CDCl_3$): δ 202.8, 153.6, 153.2, 149.1, 144.1, 135.5, 129.4, 127.2, 122.8, 116.2, 34.9, 34.3, 32.9, 27.6, 23.3, 21.5, 12.6, 8.6; EI-MS calcd for $C_{20}H_{26}N_2O_3S[M^+]$: 374.1664, found: 374.1660.

Spectral data for *N*-(2-butyl-5-methyl-3-pentanoyl-4*H*-azepin-7-yl)-*N*,4-dimethyl benzene sulfonamide (4e).

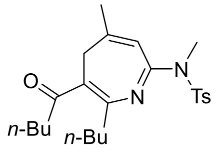

(**4e**)

White solid, mp: 65-66 °C (0.139 g, 0.32 mmol, 81%); ^{1}H NMR (400 MHz, $CDCl_3$): δ 7.55 (dd, *J* = 6.6 Hz, 1.6 Hz, 2H), 7.21 (d, *J* = 7.9 Hz, 2H), 6.11 (d, *J* = 1.3 Hz, 1H), 3.25 (s, 3H), 2.54 (t, *J* = 7.3 Hz, 2H), 2.38 ~ 2.32 (m, 5H), 2.13 (br, 2H), 2.03 (d, *J* = 1.3 Hz, 3H), 1.59 ~ 1.51 (m, 2H), 1.39 ~ 1.26 (m, 4H), 1.22 ~ 1.11 (m, 2H), 0.87 (t, *J* = 7.3 Hz, 3H), 0.80 (t, *J* = 9.0 Hz, 3H); ^{13}C NMR (100 MHz, $CDCl_3$): δ 202.8, 153.5, 151.8, 149.1, 144.0, 135.5, 129.4, 127.1, 123.7, 116.1, 41.6, 34.3, 33.8, 32.9, 30.4, 26.7, 23.2, 22.4, 22.3, 21.5, 13.9, 13.8; ESI-MS calcd for $C_{24}H_{35}N_2O_3S[M+H]$: 431.2368, found: 431.2395.

Spectral data for *N*-(2,5-dimethyl-3-pentanoyl-4*H*-azepin-7-yl)-*N*,4-dimethyl benzene sulfonamide (4f).

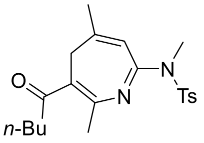

(**4f**)

Yellow oil (0.127 g, 0.32 mmol, 82%); ^{1}H NMR (400 MHz, $CDCl_3$): δ 7.55 (d, *J* = 8.3 Hz, 2H), 7.21 (d, *J* = 8.2 Hz, 2H), 6.09 (d, *J* = 1.0 Hz, 1H), 3.24 (s, 3H), 2.54 (t, *J* = 7.3 Hz, 2H), 2.35 (s, 3H), 2.21 (br, 2H), 2.03 (s, 3H), 2.00 (s, 3H), 1.58 ~ 1.50 (m, 2H), 1.36 ~ 1.15 (m, 2H), 0.86 (t, *J* = 7.3 Hz, 3H); ^{13}C NMR (100 MHz, $CDCl_3$): δ 202.1, 153.2, 149.2, 148.6, 144.1, 135.5, 129.4, 127.2, 123.4, 116.1, 41.6, 34.4, 32.6, 26.6, 23.2, 22.4, 21.9, 21.4, 13.8; ESI-MS calcd for $C_{21}H_{29}N_2O_3S[M+H]$: 389.1899, found: 389.1891.

Spectral data for *N*-(2-butyl-3-(cyclopropanecarbonyl)-5-methyl-4*H*-azepin-7-yl)-*N*,4-dimethylbenzenesulfonamide (4g).

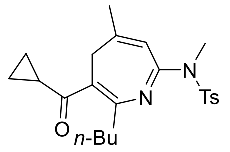

(**4g**)

White solid, mp: 87-88 °C (0.128 g, 0.31 mmol, 77%); ^{1}H NMR (400 MHz, $CDCl_3$): δ 7.56 (d, *J* = 8.4 Hz, 2H), 7.21 (d, *J* = 8.3 Hz, 2H), 6.09 (d, *J* = 1.3 Hz, 1H), 3.27 (s, 3H), 2.45 (t, *J* = 7.5 Hz, 2H), 2.36 (s, 3H), 2.20 ~ 2.08 (m, 3H), 2.03 (d, *J* = 1.2 Hz, 3H), 1.39 ~ 1.31 (m, 2H), 1.18 ~ 1.11 (m, 2H), 1.09 ~ 1.05 (m, 2H), 0.91 ~ 0.86 (m, 2H), 0.79 (t, *J* = 7.3 Hz, 3H); ^{13}C NMR (100 MHz, $CDCl_3$): δ 203.5, 153.6, 150.8, 149.7, 144.0, 135.5, 129.4, 127.2, 125.0, 115.8, 34.3, 33.6, 33.4, 30.3, 23.1, 22.4, 21.5, 21.1, 13.8, 11.8; ESI-MS calcd for $C_{23}H_{31}N_2O_3S$[M+H]: 415.2055, found: 415.2045.

Spectral data for *N*,4-dimethyl-*N*-(5-methyl-3-pentanoyl-2-phenyl-4*H*-azepin-7-yl)benzene sulfonamide (4h).

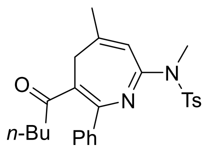

(**4h**)

Yellow oil (0.125 g, 0.27 mmol, 69%); ^{1}H NMR (400 MHz, $CDCl_3$): δ 7.59 (dd, *J* = 8.5 Hz, 1.9 Hz, 2H), 7.30 ~ 7.22 (m, 7H), 6.12 (d, *J* = 1.3 Hz, 1H), 3.27 (s, 3H), 2.37 (s, 3H), 2.26 (br, 2H), 2.10 (d, *J* = 1.3 Hz, 3H), 1.91 (t, *J* = 7.3 Hz, 2H), 1.33 ~ 1.23 (m, 2H), 1.00 ~ 0.91 (m, 2H), 0.64 (t, *J* = 7.3 Hz, 3H); ^{13}C NMR (100 MHz, $CDCl_3$): δ 206.4, 152.9, 149.8, 149.2, 144.1, 138.9, 135.5, 129.5, 128.7, 128.6, 128.3, 127.2, 126.8, 115.5, 41.8, 34.3, 34.1, 27.4, 23.1, 22.1, 21.5, 13.6; ESI-MS calcd for $C_{26}H_{31}N_2O_3S$[M+H]: 451.2055, found: 451.2048.

Spectral data for *N*-(3-benzoyl-5-methyl-2-phenyl-4*H*-azepin-7-yl)-*N*,4-dimethyl benzene sulfonamide (4i).

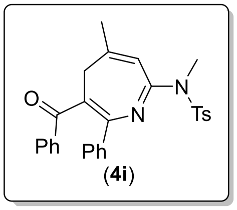

Yellowish white solid, mp: 67-68 °C (0.115 g, 0.24 mmol, 61%); ^{1}H NMR (400 MHz, $CDCl_3$): δ 7.65 (d, *J* = 8.3 Hz, 2H), 7.54 (d, *J* = 7.2 Hz, 2H), 7.27 (d, *J* = 8.0 Hz, 2H), 7.19 (t, *J* = 7.4 Hz, 1H),7.15 ~ 7.13 (m, 2H), 7.07 (t, *J* = 7.7 Hz, 2H), 6.95 (t, *J* = 3.2 Hz, 3H), 6.23 (d, *J* = 0.9 Hz, 1H), 3.35 (s, 3H), 2.45 (br, 2H), 2.41 (s, 3H), 2.18 (s, 3H); ^{13}C NMR (100 MHz, $CDCl_3$): δ 198.2, 153.5, 149.4, 149.0, 144.2, 138.2, 137.3, 135.5, 131.9, 129.5, 129.2, 128.9, 128.1, 127.7, 127.6, 127.2, 123.3, 115.8, 35.3, 34.4, 23.3, 21.5; ESI-MS calcd for $C_{28}H_{27}N_2O_3S$[M+H]: 471.1742, found: 471.1743.

Spectral data for *N*-(3-benzoyl-2,5-dimethyl-4*H*-azepin-7-yl)-*N*,4-dimethyl benzene sulfonamide (4j).

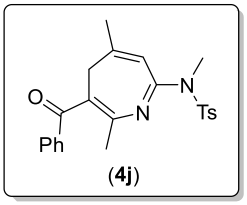

White solid, mp: 149-150 °C (0.116 g, 0.28 mmol, 71%); ^{1}H NMR (400 MHz, $CDCl_3$): δ 7.74 (dd, *J* = 7.8 Hz, 1.0 Hz, 2H), 7.60 (d, *J* = 8.3 Hz, 2H), 7.52 ~ 7.48 (m, 1H), 7.40 (t, *J* = 7.8 Hz, 2H), 7.25 (d, *J* = 7.9 Hz, 2H), 6.15 (d, *J* = 1.3 Hz, 1H), 3.28 (s, 3H), 2.38 (s, 3H), 2.25 (s, 2H), 2.05 (d, *J* = 1.2 Hz, 3H), 1.59 (s, 3H); ^{13}C NMR (100 MHz, $CDCl_3$): δ 197.8, 153.8, 149.7, 146.4, 144.1, 138.5, 135.5, 132.6, 129.5, 128.9, 128.6, 127.3, 122.1, 115.9, 34.3, 34.2, 23.3, 22.0, 21.5; ESI-MS calcd for $C_{23}H_{25}N_2O_3S$[M+H]: 409.1586, found: 409.1580.

Spectral data for *N*-(4,6-dimethyl-5-(2-oxopropyl)pyridin-2-yl)-*N*,4-dimethyl benzene sulfonamide (5a).

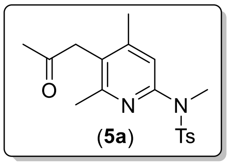

White solid, mp: 141-142 °C (0.101 g, 0.29 mmol, 73%); ^{1}H NMR (400 MHz, CDCl$_3$): δ 7.49 (d, *J* = 8.1 Hz, 2H), 7.26 (s, 1H), 7.19 (d, *J* = 8.0 Hz, 2H), 3.71 (s, 2H), 3.20 (s, 3H), 2.35 (s, 3H), 2.23 (s, 3H), 2.19 (s, 3H), 2.18 (s, 3H); ^{13}C NMR (100 MHz, CDCl$_3$): δ 204.7, 155.4, 151.4, 147.7, 143.4, 134.9, 129.2, 127.6, 124.9, 118.9, 43.8, 35.4, 29.6, 22.4, 21.4, 19.9; EI-MS calcd for $C_{18}H_{22}N_2O_3S$[M$^+$]: 346.1351, found: 346.1352.

Spectral data for *N*-(4-butyl-6-methyl-5-(2-oxopropyl)pyridin-2-yl)-*N*,4-dimethyl benzene sulfonamide (5b).

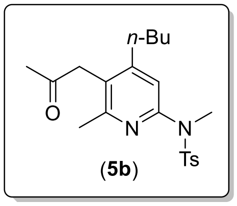

Yellow oil (0.085 g, 0.22 mmol, 64%); ^{1}H NMR (400 MHz, CDCl$_3$): δ 7.50 (d, *J* = 8.3 Hz, 2H), 7.28 (s, 1H), 7.20 (d, *J* = 8.4 Hz, 2H), 3.73 (s, 2H), 3.23 (s, 3H), 2.48 (t, *J* = 7.7 Hz, 2H), 2.37 (s, 3H), 2.23 (s, 3H), 2.19 (s, 3H), 1.53 ~ 1.46 (m, 2H), 1.40 ~ 1.30 (m, 2H), 0.92 (t, *J* = 7.2 Hz, 3H); ^{13}C NMR (100 MHz, CDCl$_3$): δ 204.9, 155.9, 151.9, 151.5, 143.4, 135.0, 129.3, 127.7, 124.3, 117.8, 43.4, 35.4, 32.8, 31.9, 29.7, 22.6, 22.5, 21.5, 13.9; ESI-MS calcd for $C_{21}H_{29}N_2O_3S$[M+H]: 389.1899, found: 389.1900.

Spectral data for *N*-(4-cyclopropyl-6-methyl-5-(2-oxopropyl)pyridin-2-yl)-*N*,4-dimethyl benzenesulfonamide (5c).

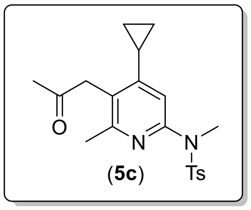

Viscous oil (0.075 g, 0.20 mmol, 56%); ^{1}H NMR (400 MHz, CDCl$_3$): δ 7.48 (d, *J* = 8.0 Hz, 2H), 7.20 (d, *J* = 7.9 Hz, 2H), 7.03 (s, 1H), 3.90 (s, 2H), 3.21 (s, 3H), 2.37 (s, 3H), 2.26 (s, 3H), 2.19 (s, 3H), 1.75 ~ 1.68 (m, 1H), 0.99 ~ 0.94 (m, 2H), 0.69 (dd, *J* = 10.4 Hz, 5.0 Hz, 2H); ^{13}C NMR (100 MHz, CDCl$_3$): δ 205.2, 155.4, 152.6, 151.9, 143.4, 134.9, 129.3, 127.7, 125.9, 114.6, 43.9, 35.4, 29.7, 22.5, 21.5, 13.4, 7.9; ESI-MS calcd for $C_{20}H_{25}N_2O_3S$[M+H]: 373.1586, found: 373.1567.

Spectral data for *N*-(4-isopropyl-6-methyl-5-(2-oxopropyl)pyridin-2-yl)-*N*,4-dimethyl benzenesulfonamide (5d).

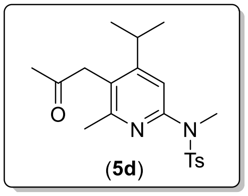

Yellow oil (0.068 g, 0.18 mmol, 51%); ^{1}H NMR (400 MHz, $CDCl_3$): δ 7.50 (d, *J* = 8.3 Hz, 2H), 7.35 (s, 1H), 7.19 (d, *J* = 8.3 Hz, 2H), 3.76 (s, 2H), 3.24 (s, 3H), 2.89 ~ 2.79 (m, 1H), 2.36 (s, 3H), 2.24 (s, 3H), 2.20 (s, 3H), 1.18 (d, *J* = 6.8 Hz, 6H); ^{13}C NMR (100 MHz, $CDCl_3$): δ 205.0, 157.8, 155.8, 151.9, 143.4, 135.0, 129.3, 127.7, 123.2, 114.3, 43.2, 35.4, 29.8, 29.7, 22.9, 22.8, 21.5; ESI-MS calcd for $C_{20}H_{27}N_2O_3S$[M+H]: 375.1742, found: 375.1747.

Spectral data for *N*-butyl-*N*-(4,6-dimethyl-5-(2-oxopropyl)pyridin-2-yl) methane sulfonamide (5e).

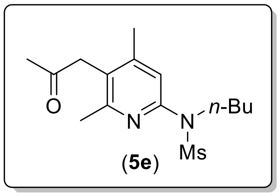

Yellow oil (0.091 g, 0.29 mmol, 63%); ^{1}H NMR (400 MHz, $CDCl_3$): δ 6.99 (s, 1H), 3.75 (t, *J* = 7.2 Hz, 2H), 3.74 (s, 2H), 2.98 (s, 3H), 2.36 (s, 3H), 2.21 (s, 3H), 2.18 (s, 3H), 1.50 ~ 1.41 (m, 2H), 1.36 ~ 1.25 (m, 2H), 0.85 (t, *J* = 7.3 Hz, 3H); ^{13}C NMR (100 MHz, $CDCl_3$): δ 204.4, 156.3, 150.5, 148.4, 126.1, 121.2, 48.4, 43.8, 38.2, 30.8, 29.8, 22.8, 19.9, 19.7, 13.5; ESI-MS calcd for $C_{15}H_{25}N_2O_3S$[M+H]: 313.1586, found: 313.1576.

Spectral data for *N*-(6-butyl-4-methyl-5-(2-oxohexyl)pyridin-2-yl)-*N*,4-dimethylbenzene sulfonamide (5f).

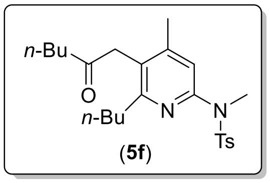

Yellow oil (0.134 g, 0.31 mmol, 78%); ^{1}H NMR (400 MHz, $CDCl_3$): δ 7.43 (d, *J* = 8.2 Hz, 2H), 7.25 (s, 1H), 7.16 (d, *J* = 8.4 Hz, 2H), 3.70 (s, 2H), 3.18 (s, 3H), 2.45 (t, *J* = 7.6 Hz, 4H), 2.34 (s,

3H), 2.17 (s, 3H), 1.60 ~ 1.52 (m, 2H), 1.40 ~ 1.33 (m, 2H), 1.32 ~ 1.24 (m, 2H), 1.22 ~ 1.11 (m, 2H), 0.87 (t, *J* = 7.4 Hz, 3H), 0.79 (t, *J* = 7.3 Hz, 3H); ^{13}C NMR (100 MHz, $CDCl_3$): δ 207.1, 158.8, 151.3, 147.9, 143.2, 134.6, 129.2, 127.6, 124.8, 119.3, 42.5, 42.2, 35.4, 34.4, 30.6, 25.9, 22.3, 22.2, 21.4, 20.1, 13.9, 13.7; ESI-MS calcd for $C_{24}H_{35}N_2O_3S$[M+H]: 431.2368, found: 431.2363.

Spectral data for *N*-(6-ethyl-4-methyl-5-(2-oxobutyl)pyridin-2-yl)-*N*,4-dimethylbenzene sulfonamide (5g).

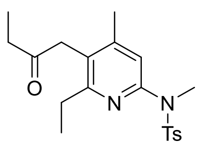

(**5g**)

White solid, mp: 95-96 °C (0.104 g, 0.27 mmol, 69%); ^{1}H NMR (400 MHz, $CDCl_3$): δ 7.45 (d, *J* = 8.2 Hz, 2H), 7.25 (s, 1H), 7.17 (d, *J* = 8.2 Hz, 2H), 3.71 (s, 2H), 3.19 (s, 3H), 2.52 ~ 2.45 (m, 4H), 2.35 (s, 3H), 2.19 (s, 3H), 1.06 (t, *J* = 7.2 Hz, 3H), 0.97 (t, *J* = 7.5 Hz, 3H); ^{13}C NMR (100 MHz, $CDCl_3$): δ 207.6, 159.7, 151.5, 147.8, 143.3, 134.6, 129.2, 127.6, 124.6, 119.4, 42.0, 35.7, 35.5, 27.9, 21.4, 20.1, 12.7, 7.8; ESI-MS calcd for $C_{20}H_{27}N_2O_3S$[M+H]: 375.1742, found: 375.1743.

Spectral data for *N*-(6-butyl-5-(2-cyclopropyl-2-oxoethyl)-4-methylpyridin-2-yl)-*N*,4-dimethylbenzenesulfonamide (5h).

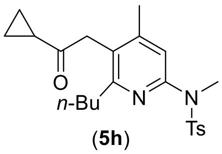

(**5h**)

White solid, mp: 72-73 °C (0.124 g, 0.30 mmol, 75%); ^{1}H NMR (400 MHz, $CDCl_3$): δ 7.44 (d, *J* = 8.1 Hz, 2H), 7.25 (s, 1H), 7.16 (d, *J* = 8.3 Hz, 2H), 3.84 (s, 2H), 3.18 (s, 3H), 2.49 (t, *J* = 7.5 Hz, 2H), 2.34 (s, 3H), 2.20 (s, 3H), 1.96 ~ 1.90 (m, 1H), 1.42 ~ 1.35 (m, 2H), 1.22 ~ 1.12 (m, 2H), 1.05 ~ 1.00 (m, 2H), 0.89 ~ 0.85 (m, 2H), 0.79 (dd, *J* = 7.6 Hz, 7.1 Hz, 3H); ^{13}C NMR (100 MHz, $CDCl_3$): δ 207.0, 158.9, 151.4, 148.0, 143.2, 134.6, 129.2, 127.6, 124.9, 119.3, 43.1, 35.5, 34.5, 30.6, 22.3, 21.4, 20.1, 13.9, 11.2; ESI-MS calcd for $C_{23}H_{31}N_2O_3S$[M+H]: 415.2055, found: 415.2047.

Spectral data for *N*,4-dimethyl-*N* -(4-methyl-5-(2-oxo-2-phenylethyl) -6-phenyl pyridin -2-yl)benzenesulfonamide (5i).

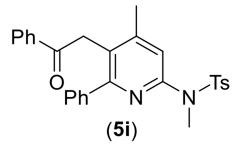

(**5i**)

White solid, mp: 135-137 °C (0.149 g, 0.32 mmol, 80%); ^{1}H NMR (400 MHz, $CDCl_3$): δ 7.91 (d, *J* = 8.3 Hz, 2H), 7.58 (t, *J* = 7.4 Hz, 1H), 7.55 (t, *J* = 8.2 Hz, 3H), 7.45 (t, *J* = 7.8 Hz, 2H), 7.27 ~ 7.23 (m, 3H), 7.22 (s, 1H), 7.20 ~ 7.17 (m, 3H), 4.29 (s, 2H), 3.25 (s, 3H), 2.38 (s, 3H), 2.25 (s, 3H); ^{13}C NMR (100 MHz, $CDCl_3$): δ 197.1, 157.7, 151.8, 149.4, 143.5, 140.1, 136.4, 134.7, 133.4, 129.3, 128.7, 128.6, 128.0, 127.7, 125.1, 120.3, 4.2, 35.6, 21.5, 20.2, two C-H peaks mixed with others; ESI-MS calcd for $C_{28}H_{27}N_2O_3S$[M+H]: 471.1742, found: 471.1741.

Spectral data for *N*-(4,6-dimethyl-5-(2-oxo-2-phenylethyl)pyridin-2-yl)-*N*,4-dimethyl benzenesulfonamide (5j).

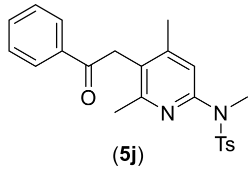

(**5j**)

Brown semi-solid (0.120 g, 0.30 mmol, 75%); ^{1}H NMR (400 MHz, $CDCl_3$): δ 8.04 (d, *J* = 8.5 Hz, 2H), 7.64 ~ 7.60 (m, 1H), 7.54 ~ 7.49 (m, 4H), 7.31 (s, 1H), 7.22 (d, *J* = 8.1 Hz, 2H), 4.32 (s, 2H), 3.23 (s, 3H), 2.38 (s, 3H), 2.24 (s, 3H), 2.20 (s, 3H); ^{13}C NMR (100 MHz, $CDCl_3$): δ 195.9, 155.7, 151.5, 148.0, 143.4, 136.7, 135.0, 133.5, 129.3, 128.8, 128.1, 127.8, 125.4, 119.1, 38.6, 35.5, 22.5, 21.5, 20.1; ESI-MS calcd for $C_{23}H_{25}N_2O_3S$[M+H]: 409.1586, found: 409.1579.

Spectral data for *N*-(4-acetyl-5-methyl-3-(1-phenylprop-1-en-2-yl)-1*H*-pyrrol-2-yl)-*N*,4-dimethylbenzenesulfonamide (6l).

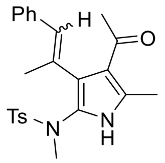

(**6l**)
(*E/Z*= 3.3:1)

White solid, mp: 171-173 °C (0.055 g, 0.13 mmol, 43%); ^{1}H NMR for major isomer (400 MHz, $CDCl_3$): δ 8.99 (s, 1H), 7.58 (d, *J* = 8.2 Hz, 2H), 7.31 ~ 7.27 (m, 2H), 7.19 ~ 7.16 (m, 1H), 7.12 (d, *J* = 8.2 Hz, 2H), 6.96 (d, *J* = 7.4 Hz, 2H), 5.55 (s, 1H), 3.22 (s, 3H), 2.46 (s, 3H), 2.27 (s, 6H), 1.82 (d, *J* = 1.3 Hz, 3H), ^{1}H NMR for minor isomer: δ 9.12 (s, 1H), 7.43 (d, *J* = 8.3 Hz, 2H), 7.22 ~ 7.19 (m, 1H), 7.04 (t, *J* = 7.7 Hz, 1H), 6.56 (d, *J* = 6.9 Hz, 1H), 2.89 (s, 3H), 2.49 (s, 3H), 2.44 (s, 3H), 1.72 (s, 3H), rest of the peaks merged with others; ^{13}C NMR for major isomer (100 MHz, $CDCl_3$): δ 195.4, 144.0, 137.3, 134.6, 133.1, 131.0, 130.8, 129.7, 128.5, 127.9, 127.5, 126.4, 124.3, 122.3, 119.8, 38.4, 29.7, 21.4, 20.6, 14.3, ^{13}C NMR for minor isomer: δ 196.1, 143.9, 134.4, 134.3, 130.0, 129.7, 127.7, 127.6, 127.0, 122.9, 120.7, 120.6, 38.6, 30.5, 21.5, 14.2, rest of the peaks merged with others; EI-MS calcd for $C_{24}H_{26}N_2O_3S[M^+]$: 422.1664, found: 422.1670.

Spectral data for *N*-(4-acetyl-3-(cyclohex-1-en-1-yl)-5-methyl-1*H*-pyrrol-2-yl)-*N*,4-dimethyl benzenesulfonamide (6m).

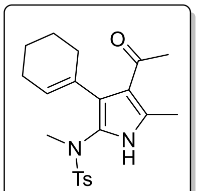

(**6m**)

White solid, mp: 175-176 °C (0.097 g, 0.25 mmol, 73%); ^{1}H NMR (400 MHz, $CDCl_3$): δ 8.65 (s, 1H), 7.58 (d, *J* = 8.3 Hz, 2H), 7.26 (d, *J* = 7.9 Hz, 2H), 4.58 (t, *J* = 1.7 Hz, 1H), 3.17 (s, 3H), 2.41 (s, 3H), 2.40 (s, 3H), 2.22 (s, 3H), 1.74 ~ 1.73 (m, 4H), 1.51 ~ 1.40 (m, 4H); ^{13}C NMR (100 MHz, $CDCl_3$): δ 195.6, 143.9, 134.6, 132.8, 131.1, 129.5, 128.3, 127.7, 123.1, 122.0, 119.9, 38.2, 30.8, 29.4, 25.4, 22.9, 21.6, 21.5, 14.4; ESI-MS calcd for $C_{21}H_{27}N_2O_3S[M+H]$: 387.1742, found: 387.1744.

Spectral data for *N*-(3-(1-hydroxyethyl)-2,5-dimethyl-4*H*-azepin-7-yl)-*N*,4-dimethylbenzene sulfonamide (7a).

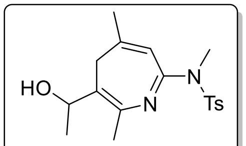

(**7a**)

White solid, mp: 165-166 °C (0.084 g, 0.24 mmol, 84%); ^{1}H NMR (600 MHz, $CDCl_3$): δ 7.59 (d, *J* = 8.1 Hz, 2H), 7.20 (d, *J* = 7.9 Hz, 2H), 6.07 (s, 1H), 4.65 (q, *J* = 6.4 Hz, 1H), 3.22 (s, 3H), 2.36 (s, 3H), 2.16 (d, *J* = 12.9 Hz, 1H), 2.07 (d, *J* = 12.7 Hz, 1H), 2.00 (s, 3H), 1.82 (s, 3H), 1.37 (br, 1H), 1.23 (d, *J* = 6.4 Hz, 3H); ^{13}C NMR (150 MHz, $CDCl_3$): δ 153.5, 147.8, 143.6, 137.5, 136.2, 129.4, 127.5, 124.7, 117.0, 66.9, 34.4, 30.1, 22.8, 21.6, 21.4, 18.5; ESI-MS calcd for $C_{18}H_{25}N_2O_3S$[M+H]: 349.1586, found: 349.1573.

Spectral data for *N*-(4-formyl-3-(prop-1-en-2-yl)-1*H*-pyrrol-2-yl)-*N*,4-dimethyl benzene sulfonamide (7a').

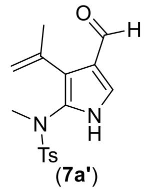

(**7a'**)

Viscous oil (0.010 g, 0.03 mmol, 8%); ^{1}H NMR (600 MHz, $CDCl_3$): δ 9.64 (s, 1H), 9.14 (s, 1H), 7.53 (d, *J* = 8.2 Hz, 2H), 7.26 (s, 1H), 7.25 (d, *J* = 7.8 Hz, 2H), 4.81 (d, *J* = 1.4 Hz, 1H), 3.88 (d, *J* = 0.8 Hz, 1H), 3.20 (s, 3H), 2.39 (s, 3H), 1.72 (s, 3H); ^{13}C NMR (150 MHz, $CDCl_3$): δ 185.5, 144.4, 136.5, 134.0, 129.7, 127.6, 125.9, 124.3, 123.5, 120.7, 117.1, 37.7, 23.9, 21.5; EI-MS calcd for $C_{16}H_{18}N_2O_3S[M^+]$: 318.1038, found: 318.1036.

Spectral data for *N*-(6-acetyl-4,7-dimethyl-4,5-dihydro-3*H*-azepin-2-yl)-*N*,4-dimethyl benzenesulfonamide (7b).

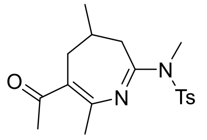

(**7b**)

Viscous oil (0.071 g, 0.20 mmol, 71%); ^{1}H NMR (400 MHz, $CDCl_3$): δ 7.68 (d, *J* = 8.3 Hz, 2H), 7.31 (d, *J* = 8.2 Hz, 2H), 3.25 (s, 3H), 2.82 (dd, *J* = 12.8 Hz, 6.5 Hz, 1H), 2.71 ~ 2.57 (m, 1H), 2.41 (s, 3H), 2.26 (s, 3H), 2.16 ~ 2.05 (m, 5H), 1.98 (dd, *J* = 14.0 Hz, 5.0 Hz, 1H), 0.92 (d, *J* = 6.8 Hz, 3H); ^{13}C NMR (100 MHz, $CDCl_3$): δ 200.5, 160.6, 155.3, 144.4, 136.0, 129.8, 127.2, 121.5, 43.9, 37.9, 35.3, 33.1, 30.5, 21.6, 21.5, 20.1; ESI-MS calcd for $C_{18}H_{25}N_2O_3S$[M+H]: 349.1586, found: 349.1572.

Spectral data for (*Z*)-*N*-(3-acetyl-2-(bromomethylene)-5-methyl-2*H*-azepin-7-yl)-*N*,4-dimethylbenzenesulfonamide (7c).

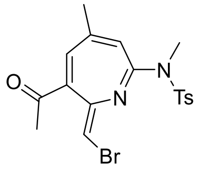

(**7c**)

Yellow oil (0.052 g, 0.12 mmol, 43%); ^{1}H NMR (400 MHz, $CDCl_3$): δ 7.44 (d, *J* = 8.3 Hz, 2H), 7.20 (d, *J* = 8.2 Hz, 2H), 6.89 (d, *J* = 1.2 Hz, 1H), 6.87 (t, *J* = 1.5 Hz, 1H), 5.45 (s, 1H), 3.21 (s, 3H), 2.36 (s, 3H), 2.31 (s, 3H), 2.13 (d, *J* = 1.3 Hz, 3H); ^{13}C NMR (100 MHz, $CDCl_3$): δ 195.7, 154.4, 146.0, 144.4, 139.8, 138.2, 135.8, 134.1, 129.6, 128.5, 127.3, 96.9, 36.7, 27.3, 24.1, 21.5; ESI-MS calcd for $C_{18}H_{19}BrN_2O_3SNa$[M+Na]: 445.0197, found: 445.0195.

(6) X-ray:

X-ray crystallographic data of compound (3b).

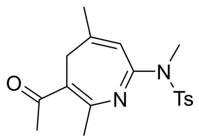

(**3b**)

CCDC 1589549

d19102

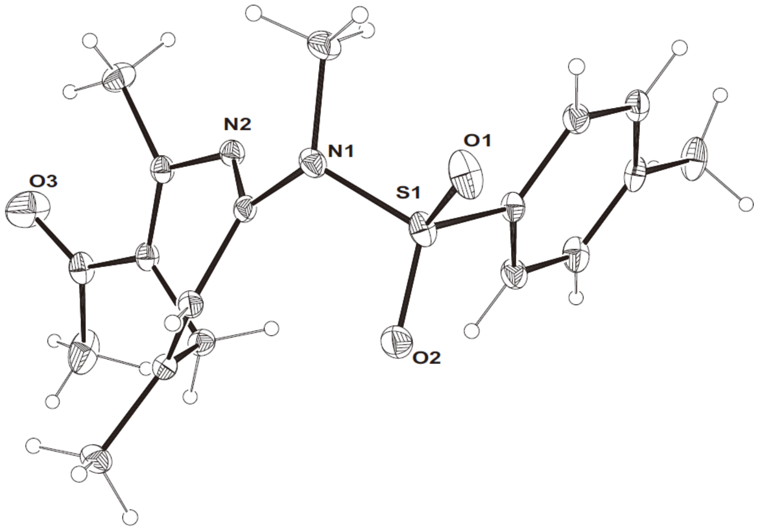

Table 1. Crystal data and structure refinement for d19102.

Identification code	d19102	
Empirical formula	C18 H22 N2 O3 S	
Formula weight	346.44	
Temperature	200(2) K	
Wavelength	0.71073 Å	
Crystal system	Monoclinic	
Space group	P 21/c	
Unit cell dimensions	a = 17.4170(10) Å	□= 90°.
	b = 7.3299(4) Å	□= 95.370(2)°.
	c = 13.5997(7) Å	□ = 90°.
Volume	1728.58(16) Å^3	
Z	4	
Density (calculated)	1.331 Mg/m^3	
Absorption coefficient	0.206 mm^{-1}	
F(000)	736	
Crystal size	0.42 x 0.29 x 0.06 mm^3	
Theta range for data collection	3.01 to 25.06°.	
Index ranges	-20<=h<=20, -8<=k<=8, -16<=l<=15	
Reflections collected	24394	
Independent reflections	3043 [R(int) = 0.0551]	
Completeness to theta = 25.06°	99.1 %	
Absorption correction	multi-scan	
Max. and min. transmission	0.9878 and 0.9185	
Refinement method	Full-matrix least-squares on F^2	
Data / restraints / parameters	3043 / 0 / 223	
Goodness-of-fit on F^2	1.061	
Final R indices [I>2sigma(I)]	R1 = 0.0752, wR2 = 0.1895	

R indices (all data)	R1 = 0.0866, wR2 = 0.1966
Extinction coefficient	0.016(2)
Largest diff. peak and hole	0.700 and -0.373 e.Å^{-3}

Table 2. Atomic coordinates (x 10^4) and equivalent isotropic displacement parameters (Å^{2}x 10^3)
for d19102. U(eq) is defined as one third of the trace of the orthogonalized U^{ij} tensor.

	x	y	z	U(eq)
C(1)	6108(2)	3097(6)	3229(3)	29(1)
C(2)	5433(3)	3936(7)	2821(3)	35(1)
C(3)	4788(3)	3895(7)	3339(4)	36(1)
C(4)	4802(3)	3049(7)	4256(4)	37(1)
C(5)	4101(3)	3019(9)	4826(4)	55(2)
C(6)	5479(3)	2236(8)	4632(4)	43(1)
C(7)	6135(3)	2261(7)	4136(4)	38(1)
C(8)	6962(3)	6784(7)	2599(4)	43(1)
C(9)	7852(2)	5245(6)	3859(3)	28(1)
C(10)	8533(2)	4112(6)	3984(3)	31(1)
C(11)	8828(3)	3582(6)	4886(4)	32(1)
C(12)	9595(3)	2631(7)	5064(4)	45(1)
C(13)	8392(3)	4002(7)	5758(3)	35(1)
C(14)	8460(3)	6031(7)	5943(3)	32(1)
C(15)	8911(3)	6728(8)	6852(4)	40(1)
C(16)	9147(4)	5452(11)	7667(5)	78(2)
C(17)	8107(2)	7179(6)	5260(3)	30(1)
C(18)	8081(3)	9214(7)	5309(4)	49(1)
N(1)	7386(2)	5116(6)	2927(3)	32(1)
N(2)	7660(2)	6540(5)	4420(3)	30(1)
O(1)	6710(2)	3350(6)	1561(2)	50(1)
O(2)	7439(2)	1723(5)	2941(3)	42(1)
O(3)	9097(3)	8320(7)	6944(4)	86(2)
S(1)	6948(1)	3177(2)	2595(1)	34(1)

Table 3. Bond lengths [Å] and angles [°] for d19102.

C(1)-C(7)	1.374(6)
C(1)-C(2)	1.395(6)
C(1)-S(1)	1.766(4)
C(2)-C(3)	1.381(7)
C(2)-H(2)	0.9500
C(3)-C(4)	1.390(7)
C(3)-H(3)	0.9500
C(4)-C(6)	1.376(7)
C(4)-C(5)	1.506(7)
C(5)-H(5A)	0.9800

C(5)-H(5B)	0.9800
C(5)-H(5C)	0.9800
C(6)-C(7)	1.381(7)
C(6)-H(6)	0.9500
C(7)-H(7)	0.9500
C(8)-N(1)	1.475(6)
C(8)-H(8A)	0.9800
C(8)-H(8B)	0.9800
C(8)-H(8C)	0.9800
C(9)-N(2)	1.282(6)
C(9)-N(1)	1.443(6)
C(9)-C(10)	1.445(6)
C(10)-C(11)	1.341(7)
C(10)-H(10)	0.9500
C(11)-C(13)	1.500(7)
C(11)-C(12)	1.506(6)
C(12)-H(12A)	0.9800
C(12)-H(12B)	0.9800
C(12)-H(12C)	0.9800
C(13)-C(14)	1.511(7)
C(13)-H(13A)	0.9900
C(13)-H(13B)	0.9900
C(14)-C(17)	1.357(7)
C(14)-C(15)	1.491(7)
C(15)-O(3)	1.215(7)
C(15)-C(16)	1.479(8)
C(16)-H(16A)	0.9800
C(16)-H(16B)	0.9800
C(16)-H(16C)	0.9800
C(17)-N(2)	1.402(6)
C(17)-C(18)	1.494(7)
C(18)-H(18A)	0.9800
C(18)-H(18B)	0.9800
C(18)-H(18C)	0.9800
N(1)-S(1)	1.656(4)
O(1)-S(1)	1.433(3)
O(2)-S(1)	1.420(4)
C(7)-C(1)-C(2)	120.6(4)
C(7)-C(1)-S(1)	119.5(4)
C(2)-C(1)-S(1)	119.8(3)
C(3)-C(2)-C(1)	118.9(4)
C(3)-C(2)-H(2)	120.5
C(1)-C(2)-H(2)	120.5
C(2)-C(3)-C(4)	121.4(5)
C(2)-C(3)-H(3)	119.3
C(4)-C(3)-H(3)	119.3
C(6)-C(4)-C(3)	118.0(4)
C(6)-C(4)-C(5)	120.6(5)
C(3)-C(4)-C(5)	121.4(5)
C(4)-C(5)-H(5A)	109.5

C(4)-C(5)-H(5B)	109.5
H(5A)-C(5)-H(5B)	109.5
C(4)-C(5)-H(5C)	109.5
H(5A)-C(5)-H(5C)	109.5
H(5B)-C(5)-H(5C)	109.5
C(7)-C(6)-C(4)	122.1(5)
C(7)-C(6)-H(6)	118.9
C(4)-C(6)-H(6)	118.9
C(1)-C(7)-C(6)	119.0(5)
C(1)-C(7)-H(7)	120.5
C(6)-C(7)-H(7)	120.5
N(1)-C(8)-H(8A)	109.5
N(1)-C(8)-H(8B)	109.5
H(8A)-C(8)-H(8B)	109.5
N(1)-C(8)-H(8C)	109.5
H(8A)-C(8)-H(8C)	109.5
H(8B)-C(8)-H(8C)	109.5
N(2)-C(9)-N(1)	114.5(4)
N(2)-C(9)-C(10)	127.7(4)
N(1)-C(9)-C(10)	117.0(4)
C(11)-C(10)-C(9)	121.0(4)
C(11)-C(10)-H(10)	119.5
C(9)-C(10)-H(10)	119.5
C(10)-C(11)-C(13)	119.0(4)
C(10)-C(11)-C(12)	122.6(4)
C(13)-C(11)-C(12)	118.4(4)
C(11)-C(12)-H(12A)	109.5
C(11)-C(12)-H(12B)	109.5
H(12A)-C(12)-H(12B)	109.5
C(11)-C(12)-H(12C)	109.5
H(12A)-C(12)-H(12C)	109.5
H(12B)-C(12)-H(12C)	109.5
C(11)-C(13)-C(14)	107.2(4)
C(11)-C(13)-H(13A)	110.3
C(14)-C(13)-H(13A)	110.3
C(11)-C(13)-H(13B)	110.3
C(14)-C(13)-H(13B)	110.3
H(13A)-C(13)-H(13B)	108.5
C(17)-C(14)-C(15)	121.6(5)
C(17)-C(14)-C(13)	118.2(4)
C(15)-C(14)-C(13)	120.2(4)
O(3)-C(15)-C(16)	118.5(5)
O(3)-C(15)-C(14)	122.0(5)
C(16)-C(15)-C(14)	119.5(5)
C(15)-C(16)-H(16A)	109.5
C(15)-C(16)-H(16B)	109.5
H(16A)-C(16)-H(16B)	109.5
C(15)-C(16)-H(16C)	109.5
H(16A)-C(16)-H(16C)	109.5
H(16B)-C(16)-H(16C)	109.5

C(14)-C(17)-N(2)	122.2(4)
C(14)-C(17)-C(18)	127.0(5)
N(2)-C(17)-C(18)	110.7(4)
C(17)-C(18)-H(18A)	109.5
C(17)-C(18)-H(18B)	109.5
H(18A)-C(18)-H(18B)	109.5
C(17)-C(18)-H(18C)	109.5
H(18A)-C(18)-H(18C)	109.5
H(18B)-C(18)-H(18C)	109.5
C(9)-N(1)-C(8)	115.8(4)
C(9)-N(1)-S(1)	120.2(3)
C(8)-N(1)-S(1)	115.1(3)
C(9)-N(2)-C(17)	125.2(4)
O(2)-S(1)-O(1)	120.1(2)
O(2)-S(1)-N(1)	107.80(19)
O(1)-S(1)-N(1)	106.0(2)
O(2)-S(1)-C(1)	108.3(2)
O(1)-S(1)-C(1)	107.8(2)
N(1)-S(1)-C(1)	106.1(2)

Symmetry transformations used to generate equivalent atoms:

Table 4. Anisotropic displacement parameters ($Å^2$ x 10^3)for d19102. The anisotropic displacement factor exponent takes the form: $-2\pi^2[h^2a^{*2}U^{11} + ... + 2 h k a^* b^* U^{12}]$

	U^{11}	U^{22}	U^{33}	U^{23}	U^{13}	U^{12}
C(1)	27(2)	35(3)	26(2)	-3(2)	0(2)	-5(2)
C(2)	34(2)	38(3)	32(2)	2(2)	-2(2)	-5(2)
C(3)	28(2)	39(3)	43(3)	-5(2)	4(2)	-6(2)
C(4)	35(3)	43(3)	35(3)	-13(2)	7(2)	-16(2)
C(5)	42(3)	73(4)	53(3)	-20(3)	22(3)	-23(3)
C(6)	43(3)	59(4)	26(2)	5(2)	-2(2)	-15(3)
C(7)	34(2)	44(3)	33(3)	6(2)	-5(2)	-5(2)
C(8)	40(3)	47(3)	39(3)	13(2)	-5(2)	4(2)
C(9)	24(2)	30(2)	29(2)	6(2)	4(2)	-5(2)
C(10)	25(2)	28(2)	39(3)	-5(2)	2(2)	0(2)
C(11)	29(2)	26(2)	41(3)	-2(2)	0(2)	-2(2)
C(12)	37(3)	42(3)	52(3)	1(3)	-14(2)	6(2)
C(13)	38(3)	34(3)	33(2)	5(2)	1(2)	-2(2)
C(14)	29(2)	37(3)	32(2)	-4(2)	6(2)	-3(2)
C(15)	35(3)	50(3)	36(3)	-11(2)	5(2)	-5(2)
C(16)	84(5)	97(6)	46(4)	16(4)	-31(3)	-44(4)
C(17)	27(2)	34(3)	31(2)	-4(2)	6(2)	-2(2)
C(18)	61(4)	31(3)	56(3)	-4(3)	5(3)	-1(3)
N(1)	27(2)	41(2)	28(2)	7(2)	-1(2)	0(2)
N(2)	27(2)	32(2)	31(2)	1(2)	5(2)	0(2)
O(1)	38(2)	85(3)	26(2)	-10(2)	4(1)	-7(2)
O(2)	35(2)	38(2)	52(2)	-10(2)	2(2)	4(2)

O(3)	117(4)	56(3)	78(3)	-22(3)	-37(3)	-5(3)
S(1)	27(1)	46(1)	27(1)	-8(1)	2(1)	-1(1)

Table 5. Hydrogen coordinates (x 10^4) and isotropic displacement parameters ($Å^2$x 10^3) for d19102.

	x	y	z	U(eq)
H(2)	5417	4525	2198	42
H(3)	4326	4457	3064	44
H(5A)	3711	3858	4519	82
H(5B)	4247	3399	5509	82
H(5C)	3889	1780	4822	82
H(6)	5495	1638	5253	51
H(7)	6598	1708	4417	45
H(8A)	7308	7838	2680	64
H(8B)	6527	6961	2998	64
H(8C)	6768	6662	1902	64
H(10)	8776	3738	3421	37
H(12A)	9790	2341	4428	67
H(12B)	9532	1502	5434	67
H(12C)	9963	3432	5444	67
H(13A)	8613	3318	6345	42
H(13B)	7844	3652	5619	42
H(16A)	8714	4647	7779	117
H(16B)	9299	6145	8270	117
H(16C)	9584	4717	7491	117
H(18A)	8237	9727	4693	74
H(18B)	8435	9640	5865	74
H(18C)	7556	9610	5403	74

X-ray crystallographic data of compound (3l).

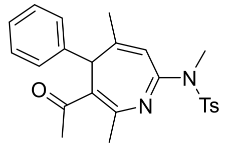

(3l)

CCDC 1589562

d19423

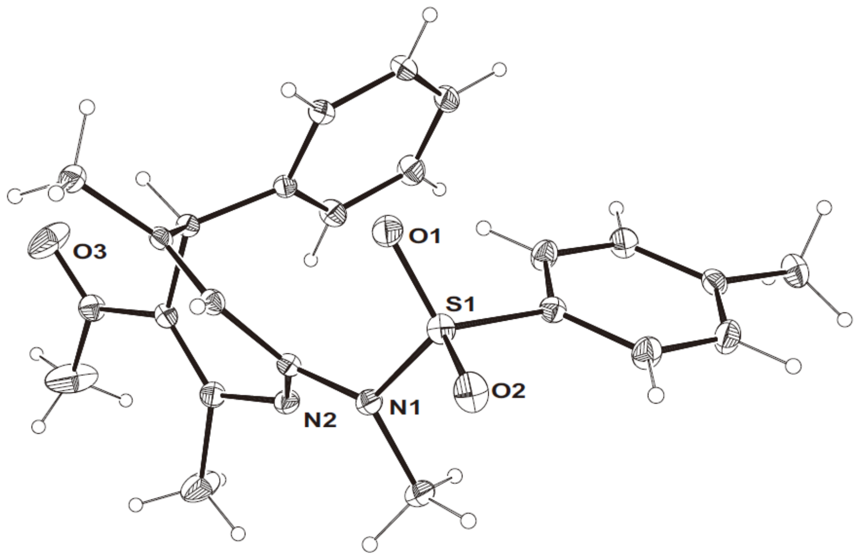

Table 1. Crystal data and structure refinement for d19423.

Identification code	d19423	
Empirical formula	C24 H26 N2 O3 S	
Formula weight	422.53	
Temperature	200(2) K	
Wavelength	0.71073 Å	
Crystal system	Monoclinic	
Space group	P 21/c	
Unit cell dimensions	a = 9.8261(4) Å	a= 90°.
	b = 13.6201(5) Å	b= 96.8680(10)°.
	c = 16.2063(5) Å	g = 90°.
Volume	2153.37(14) Å^3	
Z	4	
Density (calculated)	1.303 Mg/m^3	
Absorption coefficient	0.178 mm^{-1}	
F(000)	896	
Crystal size	0.56 x 0.49 x 0.13 mm^3	
Theta range for data collection	2.57 to 25.04°.	
Index ranges	-11<=h<=11, -16<=k<=16, -18<=l<=19	
Reflections collected	31582	
Independent reflections	3775 [R(int) = 0.0333]	
Completeness to theta = 25.04°	99.3 %	
Absorption correction	multi-scan	
Max. and min. transmission	0.9772 and 0.9066	
Refinement method	Full-matrix least-squares on F^2	

Data / restraints / parameters	3775 / 0 / 276
Goodness-of-fit on F^2	1.020
Final R indices [I>2sigma(I)]	R1 = 0.0377, wR2 = 0.0905
R indices (all data)	R1 = 0.0461, wR2 = 0.0989
Largest diff. peak and hole	0.287 and -0.367 e.$Å^{-3}$

Table 2. Atomic coordinates (x 10^4) and equivalent isotropic displacement parameters ($Å^2$x 10^3)
for d19423. U(eq) is defined as one third of the trace of the orthogonalized U^{ij} tensor.

	x	y	z	U(eq)
C(1)	3416(2)	7705(1)	6821(1)	29(1)
C(2)	4340(2)	7025(1)	7190(1)	38(1)
C(3)	3936(2)	6376(1)	7766(1)	39(1)
C(4)	2610(2)	6385(1)	7981(1)	34(1)
C(5)	2162(2)	5654(2)	8590(1)	47(1)
C(6)	1706(2)	7074(2)	7604(1)	44(1)
C(7)	2091(2)	7731(2)	7025(1)	41(1)
C(8)	2978(2)	7207(2)	4972(1)	38(1)
C(9)	5475(2)	7536(1)	5072(1)	28(1)
C(10)	6530(2)	8278(1)	5056(1)	28(1)
C(11)	7868(2)	8061(1)	5177(1)	27(1)
C(12)	8961(2)	8803(1)	5073(1)	35(1)
C(13)	8351(2)	7033(1)	5418(1)	27(1)
C(14)	7968(2)	6674(1)	6253(1)	28(1)
C(15)	8060(2)	7318(1)	6923(1)	35(1)
C(16)	7883(2)	6987(2)	7712(1)	42(1)
C(17)	7603(2)	6014(2)	7844(1)	43(1)
C(18)	7483(2)	5375(2)	7184(1)	45(1)
C(19)	7668(2)	5701(1)	6392(1)	38(1)
C(20)	7916(2)	6373(1)	4676(1)	29(1)
C(21)	9071(2)	5960(1)	4270(1)	38(1)
C(22)	8887(3)	5201(3)	3610(2)	103(1)
C(23)	6565(2)	6240(1)	4417(1)	34(1)
C(24)	5931(2)	5623(2)	3696(2)	74(1)
N(1)	4166(1)	7831(1)	5272(1)	31(1)
N(2)	5515(2)	6644(1)	4813(1)	32(1)
O(1)	5225(1)	8952(1)	6386(1)	42(1)
O(2)	2792(1)	9156(1)	5827(1)	45(1)
O(3)	10220(2)	6231(2)	4485(1)	90(1)
S(1)	3934(1)	8528(1)	6080(1)	32(1)

Table 3. Bond lengths [Å] and angles [°] for d19423.

C(1)-C(7)	1.381(3)
C(1)-C(2)	1.382(2)

C(1)-S(1)	1.7617(18)
C(2)-C(3)	1.379(3)
C(2)-H(2)	0.9500
C(3)-C(4)	1.389(3)
C(3)-H(3)	0.9500
C(4)-C(6)	1.382(3)
C(4)-C(5)	1.504(3)
C(5)-H(5A)	0.9800
C(5)-H(5B)	0.9800
C(5)-H(5C)	0.9800
C(6)-C(7)	1.382(3)
C(6)-H(6)	0.9500
C(7)-H(7)	0.9500
C(8)-N(1)	1.478(2)
C(8)-H(8A)	0.9800
C(8)-H(8B)	0.9800
C(8)-H(8C)	0.9800
C(9)-N(2)	1.288(2)
C(9)-N(1)	1.421(2)
C(9)-C(10)	1.451(2)
C(10)-C(11)	1.340(2)
C(10)-H(10)	0.9500
C(11)-C(12)	1.498(2)
C(11)-C(13)	1.515(2)
C(12)-H(12A)	0.9800
C(12)-H(12B)	0.9800
C(12)-H(12C)	0.9800
C(13)-C(20)	1.520(2)
C(13)-C(14)	1.527(2)
C(13)-H(13)	1.0000
C(14)-C(19)	1.382(3)
C(14)-C(15)	1.390(2)
C(15)-C(16)	1.386(3)
C(15)-H(15)	0.9500
C(16)-C(17)	1.375(3)
C(16)-H(16)	0.9500
C(17)-C(18)	1.373(3)
C(17)-H(17)	0.9500
C(18)-C(19)	1.391(3)
C(18)-H(18)	0.9500
C(19)-H(19)	0.9500
C(20)-C(23)	1.356(2)
C(20)-C(21)	1.489(2)
C(21)-O(3)	1.199(2)
C(21)-C(22)	1.483(3)
C(22)-H(22A)	0.9800
C(22)-H(22B)	0.9800
C(22)-H(22C)	0.9800
C(23)-N(2)	1.392(2)
C(23)-C(24)	1.511(3)

C(24)-H(24A)	0.9800
C(24)-H(24B)	0.9800
C(24)-H(24C)	0.9800
N(1)-S(1)	1.6542(15)
O(1)-S(1)	1.4278(14)
O(2)-S(1)	1.4313(14)
C(7)-C(1)-C(2)	120.18(17)
C(7)-C(1)-S(1)	120.29(14)
C(2)-C(1)-S(1)	119.52(13)
C(3)-C(2)-C(1)	119.70(17)
C(3)-C(2)-H(2)	120.2
C(1)-C(2)-H(2)	120.2
C(2)-C(3)-C(4)	121.25(17)
C(2)-C(3)-H(3)	119.4
C(4)-C(3)-H(3)	119.4
C(6)-C(4)-C(3)	117.91(17)
C(6)-C(4)-C(5)	121.07(17)
C(3)-C(4)-C(5)	121.01(17)
C(4)-C(5)-H(5A)	109.5
C(4)-C(5)-H(5B)	109.5
H(5A)-C(5)-H(5B)	109.5
C(4)-C(5)-H(5C)	109.5
H(5A)-C(5)-H(5C)	109.5
H(5B)-C(5)-H(5C)	109.5
C(7)-C(6)-C(4)	121.72(18)
C(7)-C(6)-H(6)	119.1
C(4)-C(6)-H(6)	119.1
C(1)-C(7)-C(6)	119.24(17)
C(1)-C(7)-H(7)	120.4
C(6)-C(7)-H(7)	120.4
N(1)-C(8)-H(8A)	109.5
N(1)-C(8)-H(8B)	109.5
H(8A)-C(8)-H(8B)	109.5
N(1)-C(8)-H(8C)	109.5
H(8A)-C(8)-H(8C)	109.5
H(8B)-C(8)-H(8C)	109.5
N(2)-C(9)-N(1)	113.89(15)
N(2)-C(9)-C(10)	126.98(16)
N(1)-C(9)-C(10)	118.40(15)
C(11)-C(10)-C(9)	122.34(16)
C(11)-C(10)-H(10)	118.8
C(9)-C(10)-H(10)	118.8
C(10)-C(11)-C(12)	122.60(16)
C(10)-C(11)-C(13)	120.90(15)
C(12)-C(11)-C(13)	116.49(15)
C(11)-C(12)-H(12A)	109.5
C(11)-C(12)-H(12B)	109.5
H(12A)-C(12)-H(12B)	109.5
C(11)-C(12)-H(12C)	109.5
H(12A)-C(12)-H(12C)	109.5

H(12B)-C(12)-H(12C)	109.5
C(11)-C(13)-C(20)	107.01(13)
C(11)-C(13)-C(14)	114.90(14)
C(20)-C(13)-C(14)	115.87(14)
C(11)-C(13)-H(13)	106.1
C(20)-C(13)-H(13)	106.1
C(14)-C(13)-H(13)	106.1
C(19)-C(14)-C(15)	118.25(16)
C(19)-C(14)-C(13)	122.02(16)
C(15)-C(14)-C(13)	119.39(15)
C(16)-C(15)-C(14)	120.79(18)
C(16)-C(15)-H(15)	119.6
C(14)-C(15)-H(15)	119.6
C(17)-C(16)-C(15)	120.39(19)
C(17)-C(16)-H(16)	119.8
C(15)-C(16)-H(16)	119.8
C(18)-C(17)-C(16)	119.34(18)
C(18)-C(17)-H(17)	120.3
C(16)-C(17)-H(17)	120.3
C(17)-C(18)-C(19)	120.56(19)
C(17)-C(18)-H(18)	119.7
C(19)-C(18)-H(18)	119.7
C(14)-C(19)-C(18)	120.66(18)
C(14)-C(19)-H(19)	119.7
C(18)-C(19)-H(19)	119.7
C(23)-C(20)-C(21)	125.62(16)
C(23)-C(20)-C(13)	119.74(15)
C(21)-C(20)-C(13)	114.57(15)
O(3)-C(21)-C(22)	116.98(19)
O(3)-C(21)-C(20)	119.75(17)
C(22)-C(21)-C(20)	123.27(18)
C(21)-C(22)-H(22A)	109.5
C(21)-C(22)-H(22B)	109.5
H(22A)-C(22)-H(22B)	109.5
C(21)-C(22)-H(22C)	109.5
H(22A)-C(22)-H(22C)	109.5
H(22B)-C(22)-H(22C)	109.5
C(20)-C(23)-N(2)	123.82(16)
C(20)-C(23)-C(24)	127.69(17)
N(2)-C(23)-C(24)	108.46(16)
C(23)-C(24)-H(24A)	109.5
C(23)-C(24)-H(24B)	109.5
H(24A)-C(24)-H(24B)	109.5
C(23)-C(24)-H(24C)	109.5
H(24A)-C(24)-H(24C)	109.5
H(24B)-C(24)-H(24C)	109.5
C(9)-N(1)-C(8)	117.59(14)
C(9)-N(1)-S(1)	123.84(11)
C(8)-N(1)-S(1)	114.31(12)
C(9)-N(2)-C(23)	125.26(15)

O(1)-S(1)-O(2)	119.36(9)
O(1)-S(1)-N(1)	107.83(8)
O(2)-S(1)-N(1)	107.13(8)
O(1)-S(1)-C(1)	109.72(8)
O(2)-S(1)-C(1)	107.17(8)
N(1)-S(1)-C(1)	104.65(8)

Symmetry transformations used to generate equivalent atoms:

Table 4. Anisotropic displacement parameters (Å^{2}x 10^3)for d19423. The anisotropic displacement factor exponent takes the form: -2□^{2}[$h^2a^{*2}U^{11}$ + ... + 2 h k a* b* U^{12}]

	U^{11}	U^{22}	U^{33}	U^{23}	U^{13}	U^{12}
C(1)	31(1)	28(1)	29(1)	-2(1)	4(1)	2(1)
C(2)	29(1)	42(1)	44(1)	6(1)	7(1)	5(1)
C(3)	38(1)	39(1)	40(1)	7(1)	5(1)	9(1)
C(4)	39(1)	35(1)	27(1)	-4(1)	7(1)	-2(1)
C(5)	57(1)	47(1)	39(1)	4(1)	16(1)	-2(1)
C(6)	32(1)	55(1)	47(1)	7(1)	13(1)	5(1)
C(7)	31(1)	46(1)	45(1)	8(1)	6(1)	10(1)
C(8)	30(1)	43(1)	41(1)	-3(1)	0(1)	-6(1)
C(9)	28(1)	31(1)	23(1)	2(1)	1(1)	-1(1)
C(10)	34(1)	26(1)	26(1)	1(1)	4(1)	-2(1)
C(11)	33(1)	27(1)	21(1)	-2(1)	4(1)	-3(1)
C(12)	34(1)	34(1)	38(1)	1(1)	4(1)	-6(1)
C(13)	28(1)	29(1)	26(1)	-2(1)	3(1)	-1(1)
C(14)	24(1)	32(1)	26(1)	3(1)	1(1)	2(1)
C(15)	36(1)	38(1)	29(1)	-2(1)	2(1)	-3(1)
C(16)	41(1)	59(1)	26(1)	-3(1)	2(1)	-3(1)
C(17)	37(1)	63(1)	28(1)	13(1)	1(1)	0(1)
C(18)	50(1)	41(1)	44(1)	15(1)	5(1)	0(1)
C(19)	47(1)	33(1)	35(1)	2(1)	5(1)	2(1)
C(20)	36(1)	24(1)	26(1)	0(1)	6(1)	0(1)
C(21)	39(1)	39(1)	37(1)	-5(1)	7(1)	3(1)
C(22)	50(2)	153(3)	104(2)	-91(2)	-7(2)	31(2)
C(23)	38(1)	31(1)	33(1)	-7(1)	7(1)	-5(1)
C(24)	46(1)	97(2)	81(2)	-61(2)	11(1)	-16(1)
N(1)	28(1)	33(1)	31(1)	-2(1)	3(1)	-2(1)
N(2)	30(1)	32(1)	32(1)	-5(1)	4(1)	-4(1)
O(1)	46(1)	38(1)	42(1)	-10(1)	10(1)	-12(1)
O(2)	50(1)	37(1)	51(1)	10(1)	12(1)	16(1)
O(3)	41(1)	113(2)	121(2)	-77(1)	33(1)	-20(1)
S(1)	36(1)	27(1)	35(1)	1(1)	7(1)	2(1)

Table 5. Hydrogen coordinates (x 10^4) and isotropic displacement parameters (Å^{2}x 10^3) for d19423.

	x	y	z	U(eq)
H(2)	5250	7006	7047	45
H(3)	4577	5913	8021	47
H(5A)	1159	5612	8521	70
H(5B)	2487	5866	9157	70
H(5C)	2549	5009	8488	70
H(6)	797	7095	7747	53
H(7)	1452	8195	6770	49
H(8A)	2975	7081	4377	57
H(8B)	2128	7544	5066	57
H(8C)	3041	6583	5275	57
H(10)	6262	8942	4956	34
H(12A)	9516	8576	4647	53
H(12B)	9547	8885	5602	53
H(12C)	8533	9433	4903	53
H(13)	9375	7055	5477	33
H(15)	8246	7993	6839	42
H(16)	7956	7435	8165	50
H(17)	7494	5786	8386	52
H(18)	7271	4705	7269	54
H(19)	7588	5250	5941	46
H(22A)	8493	5503	3087	155
H(22B)	8268	4688	3769	155
H(22C)	9778	4910	3539	155
H(24A)	6324	5812	3191	111
H(24B)	4938	5730	3615	111
H(24C)	6121	4928	3816	111

X-ray crystallographic data of compound (4a).

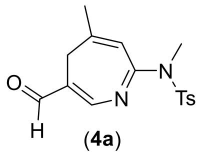

(**4a**)

CCDC 1589561

19381

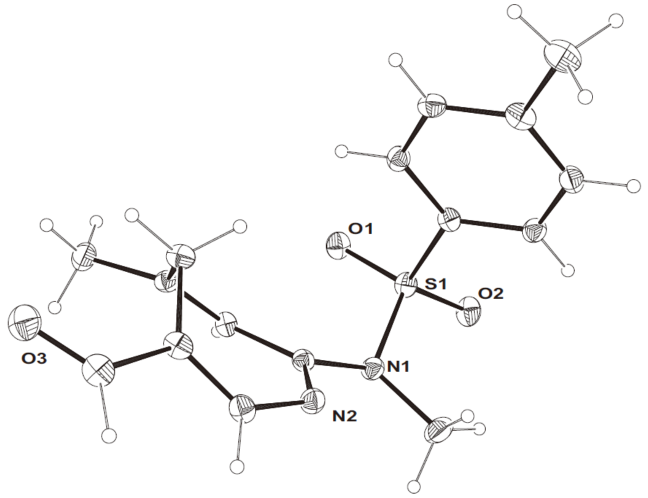

Table 1. Crystal data and structure refinement for ch19381.

Identification code	ch19381	
Empirical formula	C16 H18 N2 O3 S	
Formula weight	318.38	
Temperature	200(2) K	
Wavelength	0.71073 Å	
Crystal system	Monoclinic	
Space group	C 2/c	
Unit cell dimensions	a = 10.9900(11) Å	□= 90°.
	b = 9.6096(9) Å	□= 94.047(7)°.
	c = 29.660(3) Å	□ = 90°.
Volume	3124.5(5) Å^3	
Z	8	
Density (calculated)	1.354 Mg/m^3	
Absorption coefficient	0.221 mm^{-1}	
F(000)	1344	
Crystal size	0.10 x 0.09 x 0.04 mm^3	
Theta range for data collection	1.38 to 25.24°.	
Index ranges	-12<=h<=13, -7<=k<=11, -35<=l<=27	
Reflections collected	8336	
Independent reflections	2790 [R(int) = 0.0562]	
Completeness to theta = 25.24°	98.2 %	
Absorption correction	multi-scan	
Max. and min. transmission	0.9912 and 0.9782	
Refinement method	Full-matrix least-squares on F^2	
Data / restraints / parameters	2790 / 0 / 199	
Goodness-of-fit on F^2	1.011	
Final R indices [I>2sigma(I)]	R1 = 0.0592, wR2 = 0.1393	
R indices (all data)	R1 = 0.1146, wR2 = 0.1623	
Largest diff. peak and hole	0.355 and -0.395 e.Å^{-3}	

Table 2. Atomic coordinates (x 10^4) and equivalent isotropic displacement parameters ($Å^2$x 10^3)

for ch19381. U(eq) is defined as one third of the trace of the orthogonalized U^{ij} tensor.

	x	y	z	U(eq)
C(1)	5071(3)	4504(4)	5728(1)	31(1)
C(2)	5405(3)	5786(4)	5566(1)	36(1)
C(3)	6510(4)	5926(4)	5374(1)	39(1)
C(4)	7279(3)	4792(5)	5346(1)	40(1)
C(5)	8525(3)	4973(5)	5158(2)	55(1)
C(6)	6922(3)	3509(4)	5497(1)	41(1)
C(7)	5821(3)	3352(4)	5690(1)	37(1)
C(8)	4779(4)	3409(4)	6786(2)	55(1)
C(9)	4660(3)	5961(4)	6686(1)	31(1)
C(10)	3873(3)	7159(4)	6594(1)	31(1)
C(11)	4284(3)	8439(4)	6524(1)	33(1)
C(12)	3467(4)	9692(4)	6497(1)	46(1)
C(13)	5630(3)	8682(4)	6490(1)	37(1)
C(14)	6215(3)	8404(4)	6952(1)	36(1)
C(15)	6735(3)	9536(5)	7232(2)	45(1)
C(16)	6191(3)	7132(4)	7132(1)	38(1)
N(1)	4197(3)	4619(3)	6558(1)	35(1)
N(2)	5677(3)	5941(3)	6939(1)	37(1)
O(1)	2931(2)	5463(3)	5876(1)	42(1)
O(2)	3316(2)	2949(3)	5987(1)	47(1)
O(3)	6628(3)	10767(3)	7135(1)	56(1)
S(1)	3736(1)	4357(1)	6018(1)	35(1)

Table 3. Bond lengths [Å] and angles [°] for ch19381.

C(1)-C(2)	1.381(5)
C(1)-C(7)	1.390(5)
C(1)-S(1)	1.757(4)
C(2)-C(3)	1.384(5)
C(2)-H(2)	0.9500
C(3)-C(4)	1.386(6)
C(3)-H(3)	0.9500
C(4)-C(6)	1.379(5)
C(4)-C(5)	1.524(5)
C(5)-H(5A)	0.9800
C(5)-H(5B)	0.9800
C(5)-H(5C)	0.9800
C(6)-C(7)	1.384(5)
C(6)-H(6)	0.9500
C(7)-H(7)	0.9500

C(8)-N(1)	1.468(5)
C(8)-H(8A)	0.9800
C(8)-H(8B)	0.9800
C(8)-H(8C)	0.9800
C(9)-N(2)	1.301(4)
C(9)-N(1)	1.428(5)
C(9)-C(10)	1.454(5)
C(10)-C(11)	1.331(5)
C(10)-H(10)	0.9500
C(11)-C(12)	1.501(5)
C(11)-C(13)	1.507(5)
C(12)-H(12A)	0.9800
C(12)-H(12B)	0.9800
C(12)-H(12C)	0.9800
C(13)-C(14)	1.496(5)
C(13)-H(13A)	0.9900
C(13)-H(13B)	0.9900
C(14)-C(16)	1.335(5)
C(14)-C(15)	1.460(5)
C(15)-O(3)	1.221(5)
C(15)-H(15)	1.0482
C(16)-N(2)	1.383(5)
C(16)-H(16)	0.9500
N(1)-S(1)	1.667(3)
O(1)-S(1)	1.427(3)
O(2)-S(1)	1.430(3)
C(2)-C(1)-C(7)	120.6(4)
C(2)-C(1)-S(1)	119.6(3)
C(7)-C(1)-S(1)	119.7(3)
C(3)-C(2)-C(1)	119.5(4)
C(3)-C(2)-H(2)	120.2
C(1)-C(2)-H(2)	120.2
C(2)-C(3)-C(4)	120.4(4)
C(2)-C(3)-H(3)	119.8
C(4)-C(3)-H(3)	119.8
C(6)-C(4)-C(3)	119.7(4)
C(6)-C(4)-C(5)	120.3(4)
C(3)-C(4)-C(5)	120.0(4)
C(4)-C(5)-H(5A)	109.5
C(4)-C(5)-H(5B)	109.5
H(5A)-C(5)-H(5B)	109.5
C(4)-C(5)-H(5C)	109.5
H(5A)-C(5)-H(5C)	109.5
H(5B)-C(5)-H(5C)	109.5
C(4)-C(6)-C(7)	120.6(4)
C(4)-C(6)-H(6)	119.7
C(7)-C(6)-H(6)	119.7
C(6)-C(7)-C(1)	119.2(4)
C(6)-C(7)-H(7)	120.4
C(1)-C(7)-H(7)	120.4

N(1)-C(8)-H(8A)	109.5
N(1)-C(8)-H(8B)	109.5
H(8A)-C(8)-H(8B)	109.5
N(1)-C(8)-H(8C)	109.5
H(8A)-C(8)-H(8C)	109.5
H(8B)-C(8)-H(8C)	109.5
N(2)-C(9)-N(1)	114.5(3)
N(2)-C(9)-C(10)	126.6(3)
N(1)-C(9)-C(10)	118.0(3)
C(11)-C(10)-C(9)	123.8(3)
C(11)-C(10)-H(10)	118.1
C(9)-C(10)-H(10)	118.1
C(10)-C(11)-C(12)	122.7(3)
C(10)-C(11)-C(13)	119.9(3)
C(12)-C(11)-C(13)	117.3(3)
C(11)-C(12)-H(12A)	109.5
C(11)-C(12)-H(12B)	109.5
H(12A)-C(12)-H(12B)	109.5
C(11)-C(12)-H(12C)	109.5
H(12A)-C(12)-H(12C)	109.5
H(12B)-C(12)-H(12C)	109.5
C(14)-C(13)-C(11)	105.8(3)
C(14)-C(13)-H(13A)	110.6
C(11)-C(13)-H(13A)	110.6
C(14)-C(13)-H(13B)	110.6
C(11)-C(13)-H(13B)	110.6
H(13A)-C(13)-H(13B)	108.7
C(16)-C(14)-C(15)	118.3(4)
C(16)-C(14)-C(13)	120.7(4)
C(15)-C(14)-C(13)	120.8(4)
O(3)-C(15)-C(14)	124.1(4)
O(3)-C(15)-H(15)	123.4
C(14)-C(15)-H(15)	112.5
C(14)-C(16)-N(2)	127.7(4)
C(14)-C(16)-H(16)	116.2
N(2)-C(16)-H(16)	116.2
C(9)-N(1)-C(8)	117.2(3)
C(9)-N(1)-S(1)	118.1(2)
C(8)-N(1)-S(1)	114.6(3)
C(9)-N(2)-C(16)	122.4(3)
O(1)-S(1)-O(2)	119.70(16)
O(1)-S(1)-N(1)	108.12(16)
O(2)-S(1)-N(1)	106.19(17)
O(1)-S(1)-C(1)	108.52(18)
O(2)-S(1)-C(1)	108.80(17)
N(1)-S(1)-C(1)	104.47(16)

Symmetry transformations used to generate equivalent atoms:

Table 4. Anisotropic displacement parameters ($Å^2$x 10^3)for ch19381. The anisotropic displacement factor exponent takes the form: $-2\pi^2[h^2a^{*2}U^{11} + ... + 2 h k a^* b^* U^{12}]$

	U^{11}	U^{22}	U^{33}	U^{23}	U^{13}	U^{12}
C(1)	30(2)	33(2)	30(2)	-6(2)	-4(2)	-5(2)
C(2)	39(2)	32(2)	35(2)	-3(2)	-3(2)	1(2)
C(3)	45(2)	40(3)	31(2)	2(2)	0(2)	-8(2)
C(4)	32(2)	55(3)	33(2)	-4(2)	-4(2)	-4(2)
C(5)	39(2)	74(3)	51(3)	-6(3)	10(2)	-10(2)
C(6)	41(2)	43(3)	37(2)	-5(2)	1(2)	8(2)
C(7)	45(2)	30(2)	36(2)	0(2)	0(2)	-2(2)
C(8)	79(3)	35(3)	50(3)	10(2)	-5(2)	-2(2)
C(9)	31(2)	31(2)	31(2)	-1(2)	6(2)	-2(2)
C(10)	24(2)	32(2)	36(2)	-5(2)	2(2)	-2(2)
C(11)	34(2)	35(2)	28(2)	2(2)	-3(2)	-4(2)
C(12)	53(3)	34(3)	49(3)	0(2)	-5(2)	3(2)
C(13)	34(2)	42(2)	35(2)	0(2)	2(2)	-13(2)
C(14)	27(2)	45(3)	36(2)	-5(2)	0(2)	-10(2)
C(15)	35(2)	52(3)	49(3)	-4(2)	0(2)	-6(2)
C(16)	33(2)	43(3)	37(2)	-7(2)	-5(2)	3(2)
N(1)	41(2)	29(2)	35(2)	1(2)	1(2)	-4(2)
N(2)	37(2)	36(2)	36(2)	0(2)	-3(2)	3(2)
O(1)	32(1)	45(2)	49(2)	-3(2)	-5(1)	5(1)
O(2)	44(2)	31(2)	65(2)	-7(2)	7(1)	-15(1)
O(3)	62(2)	47(2)	58(2)	-7(2)	-6(2)	-16(2)
S(1)	33(1)	31(1)	41(1)	-4(1)	-1(1)	-6(1)

Table 5. Hydrogen coordinates (x 10^4) and isotropic displacement parameters ($Å^2$x 10^3) for ch19381.

	x	y	z	U(eq)
H(2)	4880	6565	5586	43
H(3)	6743	6805	5261	47
H(5A)	8630	5946	5069	82
H(5B)	8582	4370	4894	82
H(5C)	9165	4721	5391	82
H(6)	7437	2724	5468	49
H(7)	5580	2466	5795	44
H(8A)	4378	2555	6672	83
H(8B)	4704	3482	7112	83
H(8C)	5644	3382	6725	83
H(10)	3016	7020	6582	37
H(12A)	3954	10527	6450	69
H(12B)	3061	9784	6779	69
H(12C)	2852	9582	6244	69
H(13A)	5956	8044	6266	44

H(13B)	5784	9653	6398	44
H(15)	7213	9176	7527	55
H(16)	6573	7033	7428	46

X-ray crystallographic data of compound (5a).

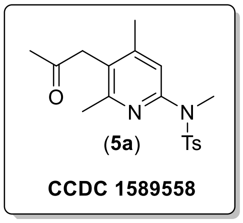

d19315

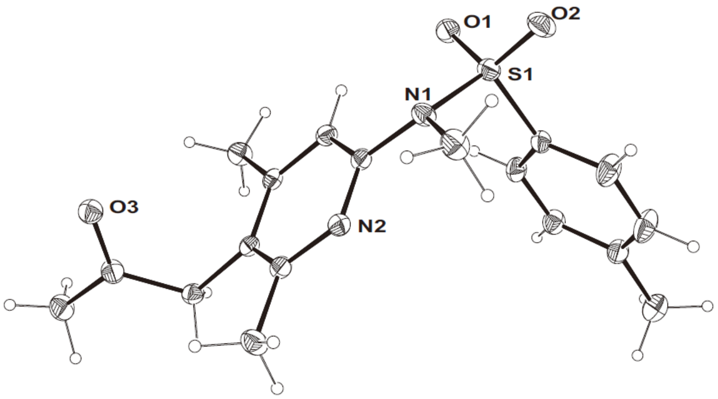

Table 1. Crystal data and structure refinement for d19315.

Identification code	d19315	
Empirical formula	C18 H22 N2 O3 S	
Formula weight	346.44	
Temperature	200(2) K	
Wavelength	0.71073 Å	
Crystal system	Monoclinic	
Space group	P 21/c	
Unit cell dimensions	a = 12.3308(12) Å	a= 90°.
	b = 16.1714(13) Å	b= 94.241(4)°.
	c = 8.8646(8) Å	g = 90°.
Volume	1762.8(3) $Å^3$	
Z	4	
Density (calculated)	1.305 Mg/m^3	
Absorption coefficient	0.202 mm^{-1}	

F(000)	736
Crystal size	0.60 x 0.03 x 0.01 mm^3
Theta range for data collection	2.52 to 25.02°.
Index ranges	-14<=h<=14, -19<=k<=19, -10<=l<=10
Reflections collected	14290
Independent reflections	3096 [R(int) = 0.1020]
Completeness to theta = 25.02°	99.5 %
Absorption correction	multi-scan
Max. and min. transmission	0.9980 and 0.8885
Refinement method	Full-matrix least-squares on F^2
Data / restraints / parameters	3096 / 0 / 222
Goodness-of-fit on F^2	1.035
Final R indices [I>2sigma(I)]	R1 = 0.0579, wR2 = 0.1300
R indices (all data)	R1 = 0.1059, wR2 = 0.1630
Largest diff. peak and hole	0.399 and -0.535 e.$Å^{-3}$

Table 2. Atomic coordinates (x 10^4) and equivalent isotropic displacement parameters ($Å^2$x 10^3)
for d19315. U(eq) is defined as one third of the trace of the orthogonalized U^{ij} tensor.

	x	y	z	U(eq)
C(1)	1885(3)	4274(2)	6765(4)	38(1)
C(2)	816(3)	4008(3)	6610(5)	61(1)
C(3)	269(3)	3866(3)	7879(5)	66(1)
C(4)	740(3)	3985(2)	9314(5)	47(1)
C(5)	146(4)	3819(3)	10706(5)	65(1)
C(6)	1813(3)	4262(2)	9454(5)	48(1)
C(7)	2378(3)	4406(2)	8197(4)	45(1)
C(8)	2453(3)	2912(2)	3996(5)	53(1)
C(9)	4053(3)	3202(2)	5781(4)	34(1)
C(10)	4864(3)	3697(2)	6487(4)	36(1)
C(11)	5671(3)	3327(2)	7427(4)	35(1)
C(12)	6562(3)	3858(2)	8180(4)	48(1)
C(13)	5663(3)	2466(2)	7599(4)	34(1)
C(14)	4857(3)	2017(2)	6770(4)	36(1)
C(15)	4817(3)	1088(2)	6783(5)	49(1)
C(16)	6521(3)	2042(2)	8640(4)	38(1)
C(17)	7599(3)	1905(2)	7999(4)	45(1)
C(18)	8508(3)	1630(3)	9069(4)	53(1)
N(1)	3178(2)	3528(2)	4790(3)	39(1)
N(2)	4050(2)	2384(2)	5904(3)	36(1)
O(1)	3438(2)	5018(1)	5500(3)	47(1)
O(2)	1829(2)	4573(2)	3883(3)	53(1)
O(3)	7826(4)	2193(4)	6752(6)	46(1)
O(3')	7613(4)	1832(3)	6601(6)	42(1)
S(1)	2597(1)	4422(1)	5138(1)	41(1)

Table 3. Bond lengths [Å] and angles [°] for d19315.

C(1)-C(7)	1.383(5)
C(1)-C(2)	1.384(5)
C(1)-S(1)	1.759(4)
C(2)-C(3)	1.373(6)
C(2)-H(2)	0.9500
C(3)-C(4)	1.372(5)
C(3)-H(3)	0.9500
C(4)-C(6)	1.394(5)
C(4)-C(5)	1.505(6)
C(5)-H(5A)	0.9800
C(5)-H(5B)	0.9800
C(5)-H(5C)	0.9800
C(6)-C(7)	1.377(5)
C(6)-H(6)	0.9500
C(7)-H(7)	0.9500
C(8)-N(1)	1.480(4)
C(8)-H(8A)	0.9800
C(8)-H(8B)	0.9800
C(8)-H(8C)	0.9800
C(9)-N(2)	1.327(4)
C(9)-C(10)	1.392(4)
C(9)-N(1)	1.441(4)
C(10)-C(11)	1.386(4)
C(10)-H(10)	0.9500
C(11)-C(13)	1.400(5)
C(11)-C(12)	1.510(5)
C(12)-H(12A)	0.9800
C(12)-H(12B)	0.9800
C(12)-H(12C)	0.9800
C(13)-C(14)	1.395(4)
C(13)-C(16)	1.515(4)
C(14)-N(2)	1.349(4)
C(14)-C(15)	1.503(5)
C(15)-H(15A)	0.9800
C(15)-H(15B)	0.9800
C(15)-H(15C)	0.9800
C(16)-C(17)	1.500(5)
C(16)-H(16A)	0.9900
C(16)-H(16B)	0.9900
C(17)-O(3')	1.246(6)
C(17)-O(3)	1.250(6)
C(17)-C(18)	1.483(5)
C(18)-H(18A)	0.9800
C(18)-H(18B)	0.9800
C(18)-H(18C)	0.9800
N(1)-S(1)	1.652(3)
O(1)-S(1)	1.435(2)

O(2)-S(1)	1.427(2)
C(7)-C(1)-C(2)	119.3(4)
C(7)-C(1)-S(1)	121.3(3)
C(2)-C(1)-S(1)	119.4(3)
C(3)-C(2)-C(1)	119.5(4)
C(3)-C(2)-H(2)	120.2
C(1)-C(2)-H(2)	120.2
C(4)-C(3)-C(2)	122.4(4)
C(4)-C(3)-H(3)	118.8
C(2)-C(3)-H(3)	118.8
C(3)-C(4)-C(6)	117.5(4)
C(3)-C(4)-C(5)	122.5(4)
C(6)-C(4)-C(5)	120.0(4)
C(4)-C(5)-H(5A)	109.5
C(4)-C(5)-H(5B)	109.5
H(5A)-C(5)-H(5B)	109.5
C(4)-C(5)-H(5C)	109.5
H(5A)-C(5)-H(5C)	109.5
H(5B)-C(5)-H(5C)	109.5
C(7)-C(6)-C(4)	121.0(4)
C(7)-C(6)-H(6)	119.5
C(4)-C(6)-H(6)	119.5
C(6)-C(7)-C(1)	120.2(3)
C(6)-C(7)-H(7)	119.9
C(1)-C(7)-H(7)	119.9
N(1)-C(8)-H(8A)	109.5
N(1)-C(8)-H(8B)	109.5
H(8A)-C(8)-H(8B)	109.5
N(1)-C(8)-H(8C)	109.5
H(8A)-C(8)-H(8C)	109.5
H(8B)-C(8)-H(8C)	109.5
N(2)-C(9)-C(10)	122.8(3)
N(2)-C(9)-N(1)	114.1(3)
C(10)-C(9)-N(1)	123.0(3)
C(11)-C(10)-C(9)	118.8(3)
C(11)-C(10)-H(10)	120.6
C(9)-C(10)-H(10)	120.6
C(10)-C(11)-C(13)	119.0(3)
C(10)-C(11)-C(12)	119.1(3)
C(13)-C(11)-C(12)	121.8(3)
C(11)-C(12)-H(12A)	109.5
C(11)-C(12)-H(12B)	109.5
H(12A)-C(12)-H(12B)	109.5
C(11)-C(12)-H(12C)	109.5
H(12A)-C(12)-H(12C)	109.5
H(12B)-C(12)-H(12C)	109.5
C(14)-C(13)-C(11)	118.0(3)
C(14)-C(13)-C(16)	121.6(3)
C(11)-C(13)-C(16)	120.4(3)
N(2)-C(14)-C(13)	122.5(3)

N(2)-C(14)-C(15)	114.9(3)
C(13)-C(14)-C(15)	122.6(3)
C(14)-C(15)-H(15A)	109.5
C(14)-C(15)-H(15B)	109.5
H(15A)-C(15)-H(15B)	109.5
C(14)-C(15)-H(15C)	109.5
H(15A)-C(15)-H(15C)	109.5
H(15B)-C(15)-H(15C)	109.5
C(17)-C(16)-C(13)	115.7(3)
C(17)-C(16)-H(16A)	108.3
C(13)-C(16)-H(16A)	108.3
C(17)-C(16)-H(16B)	108.3
C(13)-C(16)-H(16B)	108.3
H(16A)-C(16)-H(16B)	107.4
O(3')-C(17)-O(3)	30.2(3)
O(3')-C(17)-C(18)	122.8(4)
O(3)-C(17)-C(18)	118.0(4)
O(3')-C(17)-C(16)	117.9(4)
O(3)-C(17)-C(16)	122.9(4)
C(18)-C(17)-C(16)	116.8(3)
C(17)-C(18)-H(18A)	109.5
C(17)-C(18)-H(18B)	109.5
H(18A)-C(18)-H(18B)	109.5
C(17)-C(18)-H(18C)	109.5
H(18A)-C(18)-H(18C)	109.5
H(18B)-C(18)-H(18C)	109.5
C(9)-N(1)-C(8)	116.3(3)
C(9)-N(1)-S(1)	121.4(2)
C(8)-N(1)-S(1)	115.0(2)
C(9)-N(2)-C(14)	118.6(3)
O(2)-S(1)-O(1)	119.06(15)
O(2)-S(1)-N(1)	106.02(15)
O(1)-S(1)-N(1)	108.27(15)
O(2)-S(1)-C(1)	108.63(17)
O(1)-S(1)-C(1)	107.72(16)
N(1)-S(1)-C(1)	106.50(16)

Symmetry transformations used to generate equivalent atoms:

Table 4. Anisotropic displacement parameters (Å^{2}x 10^3)for d19315. The anisotropic displacement factor exponent takes the form: -2p^2[h^{2}a*^{2}U^{11} + ... + 2 h k a* b* U^{12}]

	U^{11}	U^{22}	U^{33}	U^{23}	U^{13}	U^{12}
C(1)	31(2)	37(2)	46(2)	2(2)	1(2)	0(2)
C(2)	36(2)	96(3)	51(3)	-6(2)	-7(2)	-17(2)
C(3)	36(2)	104(4)	58(3)	-7(3)	1(2)	-18(2)
C(4)	38(2)	49(2)	55(3)	0(2)	7(2)	0(2)
C(5)	54(3)	74(3)	67(3)	0(2)	17(2)	-4(2)

C(6)	42(2)	57(2)	45(2)	-1(2)	-3(2)	-2(2)
C(7)	31(2)	51(2)	51(2)	-2(2)	-3(2)	-4(2)
C(8)	49(2)	50(2)	57(3)	-5(2)	-12(2)	-4(2)
C(9)	32(2)	38(2)	33(2)	2(2)	6(2)	0(2)
C(10)	36(2)	35(2)	38(2)	0(2)	5(2)	-3(2)
C(11)	33(2)	42(2)	32(2)	-1(2)	8(2)	-1(2)
C(12)	48(2)	44(2)	51(2)	-1(2)	-4(2)	-6(2)
C(13)	34(2)	38(2)	30(2)	2(2)	9(2)	1(2)
C(14)	34(2)	39(2)	37(2)	1(2)	6(2)	-1(2)
C(15)	51(2)	35(2)	60(3)	0(2)	-2(2)	2(2)
C(16)	39(2)	41(2)	34(2)	5(2)	3(2)	0(2)
C(17)	48(2)	53(2)	33(2)	3(2)	5(2)	10(2)
C(18)	44(2)	65(3)	49(3)	8(2)	-7(2)	7(2)
N(1)	40(2)	32(2)	44(2)	1(1)	-5(1)	-1(1)
N(2)	35(2)	34(2)	40(2)	1(1)	5(1)	-1(1)
O(1)	40(1)	35(1)	66(2)	4(1)	-1(1)	-7(1)
O(2)	55(2)	55(2)	47(2)	13(1)	-12(1)	4(1)
S(1)	38(1)	36(1)	49(1)	7(1)	-1(1)	-1(1)

Table 5. Hydrogen coordinates (x 10^4) and isotropic displacement parameters (Å^{2}x 10^3) for d19315.

	x	y	z	U(eq)
H(2)	464	3923	5633	74
H(3)	-463	3679	7759	79
H(5A)	-634	3912	10478	97
H(5B)	418	4192	11520	97
H(5C)	268	3244	11026	97
H(6)	2160	4354	10433	58
H(7)	3108	4595	8314	53
H(8A)	2003	3186	3187	79
H(8B)	1984	2662	4716	79
H(8C)	2894	2481	3561	79
H(10)	4864	4278	6327	43
H(12A)	7254	3725	7761	72
H(12B)	6620	3751	9271	72
H(12C)	6389	4442	7997	72
H(15A)	4329	894	5934	73
H(15B)	4548	898	7736	73
H(15C)	5548	867	6686	73
H(16A)	6641	2378	9572	46
H(16B)	6233	1499	8937	46
H(18A)	9064	1358	8507	80
H(18B)	8235	1239	9797	80
H(18C)	8828	2109	9611	80

X-ray crystallographic data of compound (5i).

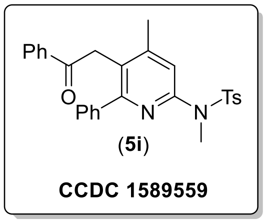

d19363

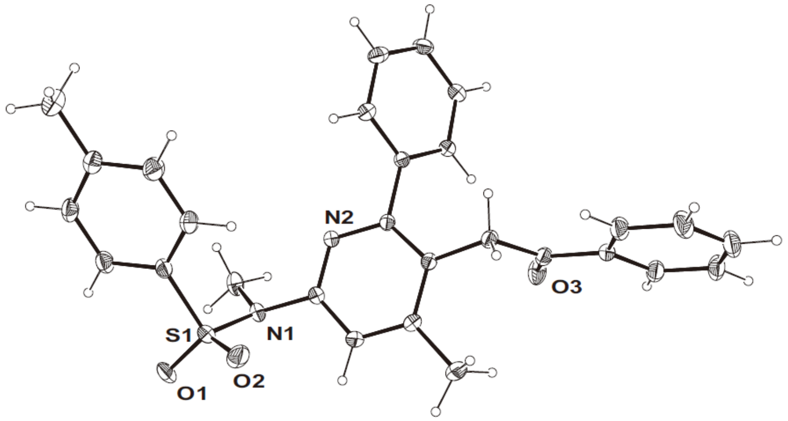

Table 1. Crystal data and structure refinement for d19363.

Identification code	d19363	
Empirical formula	C28 H26 N2 O3 S	
Formula weight	470.57	
Temperature	200(2) K	
Wavelength	0.71073 Å	
Crystal system	Monoclinic	
Space group	P 21/c	
Unit cell dimensions	a = 13.9904(6) Å	a= 90°.
	b = 8.4064(3) Å	b= 104.4910(10)°.
	c = 21.3600(9) Å	g = 90°.
Volume	2432.21(17) $Å^3$	
Z	4	
Density (calculated)	1.285 Mg/m^3	
Absorption coefficient	0.166 mm^{-1}	
F(000)	992	

Crystal size	0.62 x 0.14 x 0.07 mm^3
Theta range for data collection	2.62 to 25.03°.
Index ranges	-16<=h<=16, -10<=k<=10, -25<=l<=25
Reflections collected	39822
Independent reflections	4281 [R(int) = 0.0544]
Completeness to theta = 25.03°	99.7 %
Absorption correction	multi-scan
Max. and min. transmission	0.9885 and 0.9043
Refinement method	Full-matrix least-squares on F^2
Data / restraints / parameters	4281 / 0 / 310
Goodness-of-fit on F^2	1.064
Final R indices [I>2sigma(I)]	R1 = 0.0398, wR2 = 0.0960
R indices (all data)	R1 = 0.0610, wR2 = 0.1141
Largest diff. peak and hole	0.138 and -0.330 e.$Å^{-3}$

Table 2. Atomic coordinates (x 10^4) and equivalent isotropic displacement parameters ($Å^2$x 10^3)
for d19363. U(eq) is defined as one third of the trace of the orthogonalized U^{ij} tensor.

	x	y	z	U(eq)
C(1)	1588(1)	7592(2)	5280(1)	43(1)
C(2)	621(1)	8075(3)	5057(1)	51(1)
C(3)	-103(2)	7374(3)	5304(1)	53(1)
C(4)	113(2)	6197(2)	5764(1)	48(1)
C(5)	-688(2)	5477(3)	6035(1)	67(1)
C(6)	1088(2)	5713(3)	5978(1)	58(1)
C(7)	1823(2)	6401(3)	5741(1)	56(1)
C(8)	2661(2)	10843(3)	5774(1)	57(1)
C(9)	4057(1)	8924(2)	5958(1)	37(1)
C(10)	4856(1)	8526(2)	5720(1)	40(1)
C(11)	5675(1)	7847(2)	6129(1)	38(1)
C(12)	6533(2)	7332(3)	5868(1)	53(1)
C(13)	5671(1)	7639(2)	6779(1)	33(1)
C(14)	6488(1)	6707(2)	7218(1)	38(1)
C(15)	7463(1)	7557(2)	7449(1)	39(1)
C(16)	8359(1)	6570(2)	7712(1)	39(1)
C(17)	9272(1)	7306(3)	7892(1)	50(1)
C(18)	10120(2)	6430(3)	8145(1)	61(1)
C(19)	10058(2)	4820(3)	8222(1)	66(1)
C(20)	9165(2)	4070(3)	8038(1)	68(1)
C(21)	8312(2)	4935(3)	7786(1)	54(1)
C(22)	4858(1)	8189(2)	6984(1)	32(1)
C(23)	4814(1)	8142(2)	7677(1)	33(1)
C(24)	5520(1)	8920(2)	8149(1)	38(1)
C(25)	5434(2)	8982(2)	8781(1)	45(1)
C(26)	4654(2)	8235(3)	8949(1)	50(1)
C(27)	3964(2)	7417(2)	8485(1)	47(1)

C(28)	4034(1)	7380(2)	7852(1)	39(1)
N(1)	3189(1)	9574(2)	5525(1)	45(1)
N(2)	4042(1)	8796(2)	6576(1)	34(1)
O(1)	2028(1)	9475(3)	4448(1)	82(1)
O(2)	3133(1)	7179(2)	4840(1)	71(1)
O(3)	7522(1)	9001(2)	7434(1)	58(1)
S(1)	2511(1)	8439(1)	4960(1)	54(1)

Table 3. Bond lengths [Å] and angles [°] for d19363.

C(1)-C(2)	1.378(3)
C(1)-C(7)	1.385(3)
C(1)-S(1)	1.756(2)
C(2)-C(3)	1.385(3)
C(2)-H(2)	0.9500
C(3)-C(4)	1.374(3)
C(3)-H(3)	0.9500
C(4)-C(6)	1.387(3)
C(4)-C(5)	1.510(3)
C(5)-H(5A)	0.9800
C(5)-H(5B)	0.9800
C(5)-H(5C)	0.9800
C(6)-C(7)	1.381(3)
C(6)-H(6)	0.9500
C(7)-H(7)	0.9500
C(8)-N(1)	1.470(3)
C(8)-H(8A)	0.9800
C(8)-H(8B)	0.9800
C(8)-H(8C)	0.9800
C(9)-N(2)	1.329(2)
C(9)-C(10)	1.380(3)
C(9)-N(1)	1.440(2)
C(10)-C(11)	1.379(3)
C(10)-H(10)	0.9500
C(11)-C(13)	1.403(2)
C(11)-C(12)	1.508(2)
C(12)-H(12A)	0.9800
C(12)-H(12B)	0.9800
C(12)-H(12C)	0.9800
C(13)-C(22)	1.395(2)
C(13)-C(14)	1.504(2)
C(14)-C(15)	1.509(2)
C(14)-H(14A)	0.9900
C(14)-H(14B)	0.9900
C(15)-O(3)	1.217(2)
C(15)-C(16)	1.492(2)
C(16)-C(17)	1.384(3)
C(16)-C(21)	1.387(3)
C(17)-C(18)	1.387(3)

C(17)-H(17)	0.9500
C(18)-C(19)	1.369(4)
C(18)-H(18)	0.9500
C(19)-C(20)	1.367(4)
C(19)-H(19)	0.9500
C(20)-C(21)	1.386(3)
C(20)-H(20)	0.9500
C(21)-H(21)	0.9500
C(22)-N(2)	1.351(2)
C(22)-C(23)	1.496(2)
C(23)-C(24)	1.388(2)
C(23)-C(28)	1.394(2)
C(24)-C(25)	1.386(3)
C(24)-H(24)	0.9500
C(25)-C(26)	1.381(3)
C(25)-H(25)	0.9500
C(26)-C(27)	1.381(3)
C(26)-H(26)	0.9500
C(27)-C(28)	1.380(3)
C(27)-H(27)	0.9500
C(28)-H(28)	0.9500
N(1)-S(1)	1.6410(17)
O(1)-S(1)	1.4288(17)
O(2)-S(1)	1.4330(18)
C(2)-C(1)-C(7)	119.7(2)
C(2)-C(1)-S(1)	120.01(16)
C(7)-C(1)-S(1)	120.27(15)
C(1)-C(2)-C(3)	119.5(2)
C(1)-C(2)-H(2)	120.3
C(3)-C(2)-H(2)	120.3
C(4)-C(3)-C(2)	121.80(19)
C(4)-C(3)-H(3)	119.1
C(2)-C(3)-H(3)	119.1
C(3)-C(4)-C(6)	118.0(2)
C(3)-C(4)-C(5)	120.77(19)
C(6)-C(4)-C(5)	121.2(2)
C(4)-C(5)-H(5A)	109.5
C(4)-C(5)-H(5B)	109.5
H(5A)-C(5)-H(5B)	109.5
C(4)-C(5)-H(5C)	109.5
H(5A)-C(5)-H(5C)	109.5
H(5B)-C(5)-H(5C)	109.5
C(7)-C(6)-C(4)	121.1(2)
C(7)-C(6)-H(6)	119.4
C(4)-C(6)-H(6)	119.4
C(6)-C(7)-C(1)	119.89(19)
C(6)-C(7)-H(7)	120.1
C(1)-C(7)-H(7)	120.1
N(1)-C(8)-H(8A)	109.5
N(1)-C(8)-H(8B)	109.5

H(8A)-C(8)-H(8B)	109.5
N(1)-C(8)-H(8C)	109.5
H(8A)-C(8)-H(8C)	109.5
H(8B)-C(8)-H(8C)	109.5
N(2)-C(9)-C(10)	124.20(16)
N(2)-C(9)-N(1)	116.67(15)
C(10)-C(9)-N(1)	119.08(16)
C(11)-C(10)-C(9)	119.23(17)
C(11)-C(10)-H(10)	120.4
C(9)-C(10)-H(10)	120.4
C(10)-C(11)-C(13)	118.04(16)
C(10)-C(11)-C(12)	119.85(17)
C(13)-C(11)-C(12)	122.11(17)
C(11)-C(12)-H(12A)	109.5
C(11)-C(12)-H(12B)	109.5
H(12A)-C(12)-H(12B)	109.5
C(11)-C(12)-H(12C)	109.5
H(12A)-C(12)-H(12C)	109.5
H(12B)-C(12)-H(12C)	109.5
C(22)-C(13)-C(11)	118.38(16)
C(22)-C(13)-C(14)	121.98(16)
C(11)-C(13)-C(14)	119.39(15)
C(13)-C(14)-C(15)	116.16(15)
C(13)-C(14)-H(14A)	108.2
C(15)-C(14)-H(14A)	108.2
C(13)-C(14)-H(14B)	108.2
C(15)-C(14)-H(14B)	108.2
H(14A)-C(14)-H(14B)	107.4
O(3)-C(15)-C(16)	120.55(17)
O(3)-C(15)-C(14)	121.72(17)
C(16)-C(15)-C(14)	117.72(16)
C(17)-C(16)-C(21)	118.61(18)
C(17)-C(16)-C(15)	119.06(18)
C(21)-C(16)-C(15)	122.33(17)
C(16)-C(17)-C(18)	120.7(2)
C(16)-C(17)-H(17)	119.6
C(18)-C(17)-H(17)	119.6
C(19)-C(18)-C(17)	119.9(2)
C(19)-C(18)-H(18)	120.1
C(17)-C(18)-H(18)	120.1
C(20)-C(19)-C(18)	120.2(2)
C(20)-C(19)-H(19)	119.9
C(18)-C(19)-H(19)	119.9
C(19)-C(20)-C(21)	120.4(2)
C(19)-C(20)-H(20)	119.8
C(21)-C(20)-H(20)	119.8
C(20)-C(21)-C(16)	120.2(2)
C(20)-C(21)-H(21)	119.9
C(16)-C(21)-H(21)	119.9
N(2)-C(22)-C(13)	123.06(15)

N(2)-C(22)-C(23)	114.27(14)
C(13)-C(22)-C(23)	122.67(15)
C(24)-C(23)-C(28)	118.92(16)
C(24)-C(23)-C(22)	120.57(15)
C(28)-C(23)-C(22)	120.43(16)
C(25)-C(24)-C(23)	120.44(17)
C(25)-C(24)-H(24)	119.8
C(23)-C(24)-H(24)	119.8
C(26)-C(25)-C(24)	120.18(19)
C(26)-C(25)-H(25)	119.9
C(24)-C(25)-H(25)	119.9
C(25)-C(26)-C(27)	119.69(18)
C(25)-C(26)-H(26)	120.2
C(27)-C(26)-H(26)	120.2
C(28)-C(27)-C(26)	120.41(18)
C(28)-C(27)-H(27)	119.8
C(26)-C(27)-H(27)	119.8
C(27)-C(28)-C(23)	120.31(18)
C(27)-C(28)-H(28)	119.8
C(23)-C(28)-H(28)	119.8
C(9)-N(1)-C(8)	117.51(15)
C(9)-N(1)-S(1)	119.42(14)
C(8)-N(1)-S(1)	116.02(13)
C(9)-N(2)-C(22)	116.79(15)
O(1)-S(1)-O(2)	120.46(11)
O(1)-S(1)-N(1)	106.46(11)
O(2)-S(1)-N(1)	107.26(9)
O(1)-S(1)-C(1)	106.94(10)
O(2)-S(1)-C(1)	107.92(11)
N(1)-S(1)-C(1)	107.15(9)

Symmetry transformations used to generate equivalent atoms:

Table 4. Anisotropic displacement parameters ($Å^2$x 10^3)for d19363. The anisotropic displacement factor exponent takes the form: $-2p^2[h^2a^{*2}U^{11} + ... + 2 h k a^* b^* U^{12}]$

	U^{11}	U^{22}	U^{33}	U^{23}	U^{13}	U^{12}
C(1)	36(1)	56(1)	34(1)	-5(1)	3(1)	1(1)
C(2)	42(1)	58(1)	47(1)	3(1)	1(1)	9(1)
C(3)	31(1)	64(1)	60(1)	-1(1)	3(1)	8(1)
C(4)	40(1)	48(1)	55(1)	-8(1)	10(1)	1(1)
C(5)	51(1)	64(2)	88(2)	-3(1)	22(1)	-6(1)
C(6)	49(1)	59(1)	68(2)	14(1)	15(1)	11(1)
C(7)	38(1)	72(2)	58(1)	12(1)	8(1)	15(1)
C(8)	44(1)	57(1)	61(1)	4(1)	-3(1)	12(1)
C(9)	34(1)	43(1)	33(1)	1(1)	5(1)	-1(1)
C(10)	40(1)	49(1)	32(1)	1(1)	11(1)	-2(1)
C(11)	35(1)	40(1)	41(1)	0(1)	15(1)	-2(1)

C(12)	47(1)	64(1)	54(1)	1(1)	25(1)	6(1)
C(13)	30(1)	32(1)	37(1)	1(1)	8(1)	0(1)
C(14)	32(1)	38(1)	44(1)	5(1)	12(1)	4(1)
C(15)	33(1)	42(1)	44(1)	4(1)	11(1)	3(1)
C(16)	32(1)	50(1)	34(1)	5(1)	9(1)	6(1)
C(17)	37(1)	61(1)	50(1)	-5(1)	7(1)	3(1)
C(18)	33(1)	85(2)	59(1)	-10(1)	-2(1)	8(1)
C(19)	49(1)	84(2)	57(1)	2(1)	-3(1)	24(1)
C(20)	60(2)	63(2)	75(2)	21(1)	4(1)	21(1)
C(21)	41(1)	54(1)	64(1)	16(1)	8(1)	7(1)
C(22)	29(1)	32(1)	34(1)	1(1)	7(1)	0(1)
C(23)	31(1)	33(1)	34(1)	6(1)	9(1)	5(1)
C(24)	36(1)	41(1)	36(1)	5(1)	8(1)	1(1)
C(25)	47(1)	53(1)	34(1)	2(1)	5(1)	1(1)
C(26)	59(1)	59(1)	34(1)	8(1)	18(1)	6(1)
C(27)	51(1)	47(1)	52(1)	8(1)	26(1)	-1(1)
C(28)	38(1)	39(1)	43(1)	0(1)	15(1)	-1(1)
N(1)	34(1)	60(1)	36(1)	6(1)	0(1)	5(1)
N(2)	30(1)	40(1)	32(1)	2(1)	6(1)	1(1)
O(1)	61(1)	137(2)	37(1)	31(1)	-9(1)	-21(1)
O(2)	51(1)	111(1)	57(1)	-36(1)	23(1)	-9(1)
O(3)	40(1)	40(1)	88(1)	5(1)	5(1)	-2(1)
S(1)	42(1)	89(1)	30(1)	-1(1)	4(1)	-7(1)

Table 5. Hydrogen coordinates (x 10^4) and isotropic displacement parameters ($Å^2$x 10^3) for d19363.

	x	y	z	U(eq)
H(2)	453	8882	4737	61
H(3)	-768	7716	5150	64
H(5A)	-578	5771	6491	100
H(5B)	-672	4316	5997	100
H(5C)	-1333	5876	5793	100
H(6)	1253	4895	6294	70
H(7)	2487	6059	5895	67
H(8A)	2190	10367	5990	85
H(8B)	2306	11510	5415	85
H(8C)	3136	11496	6084	85
H(10)	4842	8719	5280	48
H(12A)	6391	7574	5405	79
H(12B)	6637	6185	5933	79
H(12C)	7130	7902	6096	79
H(14A)	6600	5730	6988	45
H(14B)	6262	6372	7602	45
H(17)	9317	8425	7842	60
H(18)	10744	6946	8264	74

H(19)	10637	4223	8404	80
H(20)	9128	2948	8083	82
H(21)	7693	4406	7664	65
H(24)	6066	9415	8039	45
H(25)	5912	9539	9100	55
H(26)	4593	8284	9382	59
H(27)	3439	6877	8602	57
H(28)	3549	6834	7534	47

X-ray crystallographic data of compound (6m).

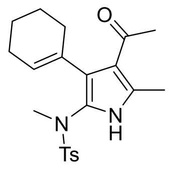

(**6m**)

CCDC 1589560

d19354

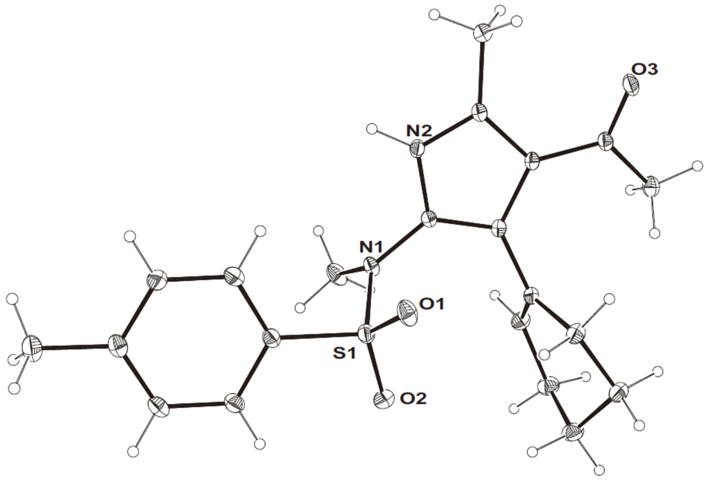

Table 1. Crystal data and structure refinement for d19354.

Identification code	d19354
Empirical formula	C21 H26 N2 O3 S
Formula weight	386.50
Temperature	200(2) K
Wavelength	0.71073 Å
Crystal system	Monoclinic
Space group	P 21/n

Unit cell dimensions	a = 9.0836(3) Å	a= 90°.
	b = 21.4854(9) Å	b= 92.1120(10)°.
	c = 9.9912(4) Å	g = 90°.
Volume	1948.61(13) Å^3	
Z	4	
Density (calculated)	1.317 Mg/m^3	
Absorption coefficient	0.190 mm^{-1}	
F(000)	824	
Crystal size	0.36 x 0.04 x 0.02 mm^3	
Theta range for data collection	2.25 to 25.03°.	
Index ranges	-10<=h<=10, -25<=k<=25, -11<=l<=9	
Reflections collected	17674	
Independent reflections	3430 [R(int) = 0.0569]	
Completeness to theta = 25.03°	99.6 %	
Absorption correction	multi-scan	
Max. and min. transmission	0.9962 and 0.9347	
Refinement method	Full-matrix least-squares on F^2	
Data / restraints / parameters	3430 / 0 / 244	
Goodness-of-fit on F^2	1.059	
Final R indices [I>2sigma(I)]	R1 = 0.0494, wR2 = 0.1195	
R indices (all data)	R1 = 0.0708, wR2 = 0.1372	
Largest diff. peak and hole	0.362 and -0.386 e.Å^{-3}	

Table 2. Atomic coordinates (x 10^4) and equivalent isotropic displacement parameters (Å^{2}x 10^3)
for d19354. U(eq) is defined as one third of the trace of the orthogonalized U^{ij} tensor.

	x	y	z	U(eq)
C(1)	2597(3)	6349(1)	5365(3)	29(1)
C(2)	2560(3)	6898(1)	4648(3)	37(1)
C(3)	1381(3)	7021(1)	3773(3)	37(1)
C(4)	233(3)	6600(1)	3597(3)	33(1)
C(5)	-1002(3)	6727(2)	2591(3)	44(1)
C(6)	278(3)	6059(1)	4349(3)	36(1)
C(7)	1449(3)	5925(1)	5229(3)	33(1)
C(8)	5672(3)	5807(1)	4469(3)	40(1)
C(9)	6817(3)	6547(1)	6101(2)	24(1)
C(10)	7783(3)	6378(1)	7114(2)	24(1)
C(11)	7644(3)	5814(1)	7962(3)	26(1)
C(12)	7944(3)	5246(1)	7481(3)	32(1)
C(13)	7778(3)	4656(1)	8264(3)	40(1)
C(14)	6844(3)	4762(1)	9470(3)	41(1)
C(15)	7349(3)	5337(1)	10226(3)	40(1)
C(16)	7137(3)	5909(1)	9352(3)	35(1)
C(17)	8834(3)	6877(1)	7242(2)	23(1)
C(18)	10106(3)	6936(1)	8155(3)	27(1)

C(19)	10733(3)	6378(1)	8864(3)	37(1)
C(20)	8447(3)	7325(1)	6274(3)	25(1)
C(21)	9161(3)	7923(1)	5917(3)	34(1)
N(1)	5531(2)	6252(1)	5563(2)	28(1)
N(2)	7230(2)	7119(1)	5600(2)	25(1)
O(1)	4223(2)	6671(1)	7477(2)	41(1)
O(2)	3909(2)	5557(1)	6944(2)	42(1)
O(3)	10708(2)	7446(1)	8347(2)	36(1)
S(1)	4092(1)	6187(1)	6498(1)	30(1)

Table 3. Bond lengths [Å] and angles [°] for d19354.

C(1)-C(2)	1.381(4)
C(1)-C(7)	1.387(4)
C(1)-S(1)	1.770(3)
C(2)-C(3)	1.382(4)
C(2)-H(2)	0.9500
C(3)-C(4)	1.387(4)
C(3)-H(3)	0.9500
C(4)-C(6)	1.383(4)
C(4)-C(5)	1.504(4)
C(5)-H(5A)	0.9800
C(5)-H(5B)	0.9800
C(5)-H(5C)	0.9800
C(6)-C(7)	1.385(4)
C(6)-H(6)	0.9500
C(7)-H(7)	0.9500
C(8)-N(1)	1.461(3)
C(8)-H(8A)	0.9800
C(8)-H(8B)	0.9800
C(8)-H(8C)	0.9800
C(9)-C(10)	1.365(3)
C(9)-N(2)	1.384(3)
C(9)-N(1)	1.418(3)
C(10)-C(17)	1.438(3)
C(10)-C(11)	1.487(3)
C(11)-C(12)	1.342(4)
C(11)-C(16)	1.493(4)
C(12)-C(13)	1.501(4)
C(12)-H(12)	0.9500
C(13)-C(14)	1.516(4)
C(13)-H(13A)	0.9900
C(13)-H(13B)	0.9900
C(14)-C(15)	1.512(4)
C(14)-H(14A)	0.9900
C(14)-H(14B)	0.9900
C(15)-C(16)	1.516(4)
C(15)-H(15A)	0.9900
C(15)-H(15B)	0.9900

C(16)-H(16A)	0.9900
C(16)-H(16B)	0.9900
C(17)-C(20)	1.400(3)
C(17)-C(18)	1.451(3)
C(18)-O(3)	1.235(3)
C(18)-C(19)	1.495(4)
C(19)-H(19A)	0.9800
C(19)-H(19B)	0.9800
C(19)-H(19C)	0.9800
C(20)-N(2)	1.348(3)
C(20)-C(21)	1.488(4)
C(21)-H(21A)	0.9800
C(21)-H(21B)	0.9800
C(21)-H(21C)	0.9800
N(1)-S(1)	1.640(2)
N(2)-H(2')	0.9278
O(1)-S(1)	1.430(2)
O(2)-S(1)	1.435(2)
C(2)-C(1)-C(7)	120.4(3)
C(2)-C(1)-S(1)	120.2(2)
C(7)-C(1)-S(1)	119.4(2)
C(1)-C(2)-C(3)	119.6(3)
C(1)-C(2)-H(2)	120.2
C(3)-C(2)-H(2)	120.2
C(2)-C(3)-C(4)	121.1(3)
C(2)-C(3)-H(3)	119.4
C(4)-C(3)-H(3)	119.4
C(6)-C(4)-C(3)	118.2(3)
C(6)-C(4)-C(5)	121.5(3)
C(3)-C(4)-C(5)	120.3(3)
C(4)-C(5)-H(5A)	109.5
C(4)-C(5)-H(5B)	109.5
H(5A)-C(5)-H(5B)	109.5
C(4)-C(5)-H(5C)	109.5
H(5A)-C(5)-H(5C)	109.5
H(5B)-C(5)-H(5C)	109.5
C(4)-C(6)-C(7)	121.7(3)
C(4)-C(6)-H(6)	119.2
C(7)-C(6)-H(6)	119.2
C(6)-C(7)-C(1)	118.9(3)
C(6)-C(7)-H(7)	120.5
C(1)-C(7)-H(7)	120.5
N(1)-C(8)-H(8A)	109.5
N(1)-C(8)-H(8B)	109.5
H(8A)-C(8)-H(8B)	109.5
N(1)-C(8)-H(8C)	109.5
H(8A)-C(8)-H(8C)	109.5
H(8B)-C(8)-H(8C)	109.5
C(10)-C(9)-N(2)	109.2(2)
C(10)-C(9)-N(1)	131.4(2)

N(2)-C(9)-N(1)	119.4(2)
C(9)-C(10)-C(17)	105.9(2)
C(9)-C(10)-C(11)	125.0(2)
C(17)-C(10)-C(11)	128.9(2)
C(12)-C(11)-C(10)	120.9(2)
C(12)-C(11)-C(16)	122.1(2)
C(10)-C(11)-C(16)	117.0(2)
C(11)-C(12)-C(13)	123.8(3)
C(11)-C(12)-H(12)	118.1
C(13)-C(12)-H(12)	118.1
C(12)-C(13)-C(14)	110.9(2)
C(12)-C(13)-H(13A)	109.5
C(14)-C(13)-H(13A)	109.5
C(12)-C(13)-H(13B)	109.5
C(14)-C(13)-H(13B)	109.5
H(13A)-C(13)-H(13B)	108.1
C(15)-C(14)-C(13)	110.6(2)
C(15)-C(14)-H(14A)	109.5
C(13)-C(14)-H(14A)	109.5
C(15)-C(14)-H(14B)	109.5
C(13)-C(14)-H(14B)	109.5
H(14A)-C(14)-H(14B)	108.1
C(14)-C(15)-C(16)	110.2(2)
C(14)-C(15)-H(15A)	109.6
C(16)-C(15)-H(15A)	109.6
C(14)-C(15)-H(15B)	109.6
C(16)-C(15)-H(15B)	109.6
H(15A)-C(15)-H(15B)	108.1
C(11)-C(16)-C(15)	112.8(2)
C(11)-C(16)-H(16A)	109.0
C(15)-C(16)-H(16A)	109.0
C(11)-C(16)-H(16B)	109.0
C(15)-C(16)-H(16B)	109.0
H(16A)-C(16)-H(16B)	107.8
C(20)-C(17)-C(10)	107.6(2)
C(20)-C(17)-C(18)	123.2(2)
C(10)-C(17)-C(18)	129.1(2)
O(3)-C(18)-C(17)	120.8(2)
O(3)-C(18)-C(19)	118.6(2)
C(17)-C(18)-C(19)	120.5(2)
C(18)-C(19)-H(19A)	109.5
C(18)-C(19)-H(19B)	109.5
H(19A)-C(19)-H(19B)	109.5
C(18)-C(19)-H(19C)	109.5
H(19A)-C(19)-H(19C)	109.5
H(19B)-C(19)-H(19C)	109.5
N(2)-C(20)-C(17)	107.4(2)
N(2)-C(20)-C(21)	121.4(2)
C(17)-C(20)-C(21)	131.2(2)
C(20)-C(21)-H(21A)	109.5

C(20)-C(21)-H(21B)	109.5
H(21A)-C(21)-H(21B)	109.5
C(20)-C(21)-H(21C)	109.5
H(21A)-C(21)-H(21C)	109.5
H(21B)-C(21)-H(21C)	109.5
C(9)-N(1)-C(8)	118.8(2)
C(9)-N(1)-S(1)	119.05(17)
C(8)-N(1)-S(1)	117.61(18)
C(20)-N(2)-C(9)	109.9(2)
C(20)-N(2)-H(2')	126.3
C(9)-N(2)-H(2')	123.4
O(1)-S(1)-O(2)	118.67(13)
O(1)-S(1)-N(1)	106.22(11)
O(2)-S(1)-N(1)	111.22(12)
O(1)-S(1)-C(1)	109.66(12)
O(2)-S(1)-C(1)	106.81(12)
N(1)-S(1)-C(1)	103.19(12)

Symmetry transformations used to generate equivalent atoms:

Table 4. Anisotropic displacement parameters ($Å^2$x 10^3)for d19354. The anisotropic displacement factor exponent takes the form: -2p^2[$h^2 a^{*2} U^{11}$ + ... + 2 h k a* b* U^{12}]

	U^{11}	U^{22}	U^{33}	U^{23}	U^{13}	U^{12}
C(1)	24(1)	34(2)	28(1)	-2(1)	1(1)	-1(1)
C(2)	29(1)	37(2)	46(2)	6(1)	-3(1)	-7(1)
C(3)	31(2)	41(2)	39(2)	10(1)	0(1)	0(1)
C(4)	26(1)	43(2)	30(2)	-8(1)	2(1)	3(1)
C(5)	35(2)	60(2)	36(2)	-3(2)	-5(1)	4(1)
C(6)	28(1)	40(2)	40(2)	-8(1)	0(1)	-7(1)
C(7)	30(1)	31(2)	38(2)	-1(1)	-1(1)	-4(1)
C(8)	36(2)	50(2)	35(2)	-19(1)	3(1)	-9(1)
C(9)	23(1)	26(1)	24(1)	0(1)	-2(1)	-1(1)
C(10)	24(1)	26(1)	21(1)	-1(1)	-1(1)	2(1)
C(11)	24(1)	30(2)	24(1)	2(1)	-3(1)	0(1)
C(12)	37(2)	29(2)	32(2)	2(1)	3(1)	2(1)
C(13)	41(2)	31(2)	47(2)	7(1)	4(1)	3(1)
C(14)	40(2)	36(2)	47(2)	15(1)	3(1)	1(1)
C(15)	39(2)	50(2)	30(2)	9(1)	1(1)	-3(1)
C(16)	41(2)	36(2)	28(2)	2(1)	0(1)	1(1)
C(17)	23(1)	27(1)	20(1)	-2(1)	-1(1)	2(1)
C(18)	24(1)	35(2)	21(1)	-4(1)	-1(1)	-1(1)
C(19)	29(1)	42(2)	39(2)	6(1)	-10(1)	2(1)
C(20)	24(1)	28(1)	23(1)	-3(1)	-2(1)	0(1)
C(21)	37(2)	32(2)	34(2)	5(1)	-4(1)	-4(1)
N(1)	24(1)	33(1)	27(1)	-7(1)	-4(1)	-3(1)
N(2)	27(1)	25(1)	24(1)	2(1)	-5(1)	1(1)
O(1)	35(1)	56(1)	33(1)	-16(1)	-1(1)	-2(1)

O(2)	42(1)	42(1)	42(1)	13(1)	-2(1)	-6(1)
O(3)	36(1)	37(1)	34(1)	-4(1)	-13(1)	-5(1)
S(1)	27(1)	37(1)	26(1)	-1(1)	-1(1)	-2(1)

Table 5. Hydrogen coordinates (x 10^4) and isotropic displacement parameters (Å^{2}x 10^3) for d19354.

	x	y	z	U(eq)
H(2)	3340	7190	4756	45
H(3)	1357	7400	3284	44
H(5A)	-838	7130	2160	66
H(5B)	-1939	6737	3046	66
H(5C)	-1033	6398	1911	66
H(6)	-514	5771	4259	43
H(7)	1466	5550	5731	39
H(8A)	4697	5642	4207	60
H(8B)	6318	5465	4765	60
H(8C)	6094	6016	3701	60
H(12)	8280	5217	6594	39
H(13A)	8763	4503	8567	48
H(13B)	7312	4333	7680	48
H(14A)	5799	4811	9170	49
H(14B)	6918	4396	10070	49
H(15A)	8403	5295	10501	48
H(15B)	6778	5383	11045	48
H(16A)	6079	6022	9309	42
H(16B)	7688	6261	9767	42
H(19A)	11580	6503	9437	55
H(19B)	11050	6073	8204	55
H(19C)	9980	6191	9415	55
H(21A)	8592	8122	5183	51
H(21B)	10165	7841	5635	51
H(21C)	9197	8199	6699	51
H(2')	6792	7298	4840	36

(7) ^{1}H-NOE:

^{1}H-NOE map of compound (7c).

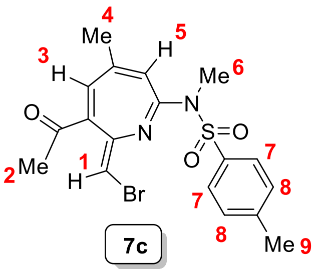

7c

Irradiation	Enhancement(%)
H^4 (δ 2.13)	H^5 (δ 6.87, 3.09%), H^3 (δ 6.89, 2.62%).
H^2 (δ 2.31)	H^1 (δ 5.45, 1.15%), H^3 (δ 6.89, 1.69%).
H^9 (δ 2.36)	H^8 (δ 7.21, 3.28%).
H^6 (δ 3.21)	H^7 (δ 7.44, 1.67%).
H^1 (δ 5.45)	H^2 (δ 2.31, 1.31%), H^8 (δ 7.21, 1.54%).
H^5 (δ 6.87)	H^4 (δ 2.13, 6.04%).
H^3 (δ 6.89)	H^2 (δ 2.31, 2.86%), H^4 (δ 2.13, 4.43%).
H^7 (δ 7.44)	H^8 (δ 7.21, 7.87%), H^5 (δ 6.87, 0.94%).

(8) Spectral data ^{1}H, ^{13}C, NOE:

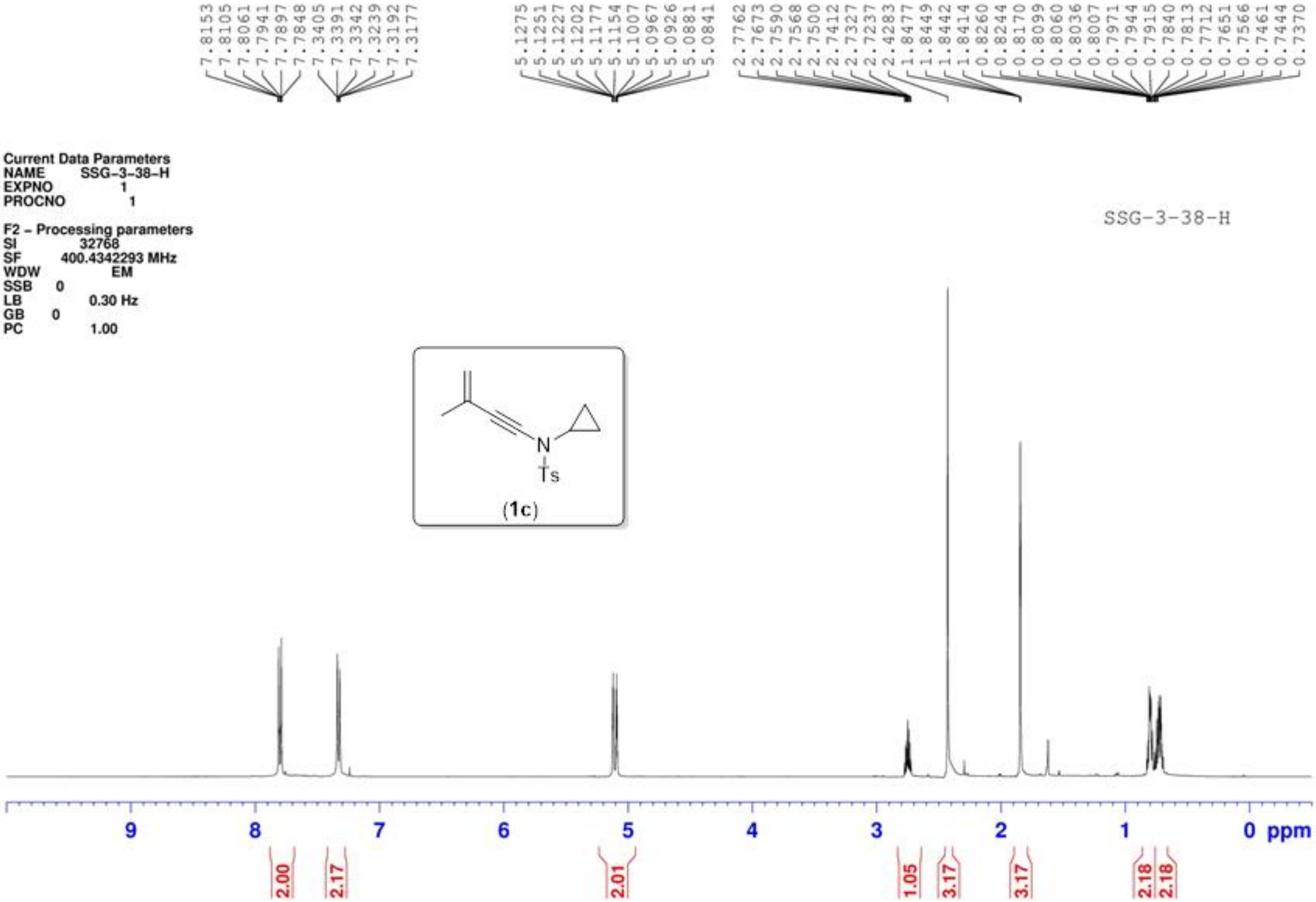
7.8153
7.8105
7.8061
7.7941
7.7897
7.7848
7.3405
7.3391
7.3342
7.3239
7.3192
7.3177
5.1275
5.1251
5.1227
5.1202
5.1177
5.1154
5.1007
5.0967
5.0926
5.0881
5.0841
2.7762
2.7673
2.7590
2.7568
2.7500
2.7412
2.7327
2.7237
2.4283
1.8477
1.8449
1.8442
1.8414
0.8260
0.8244
0.8170
0.8099
0.8060
0.8036
0.8007
0.7971
0.7944
0.7915
0.7840
0.7813
0.7712
0.7651
0.7566
0.7461
0.7444
0.7370
Current Data Parameters
NAME SSG-3-38-H
EXPNO 1
PROCNO 1
F2 - Processing parameters
SI 32768
SF 400.4342293 MHz
WDW EM
SSB 0
LB 0.30 Hz
GB 0
PC 1.00
SSG-3-38-H
N
Ts
(1c)
9
8
7
6
5
4
3
2
1
0 ppm
2.00
2.17
2.01
1.05
3.17
3.17
2.18
2.18

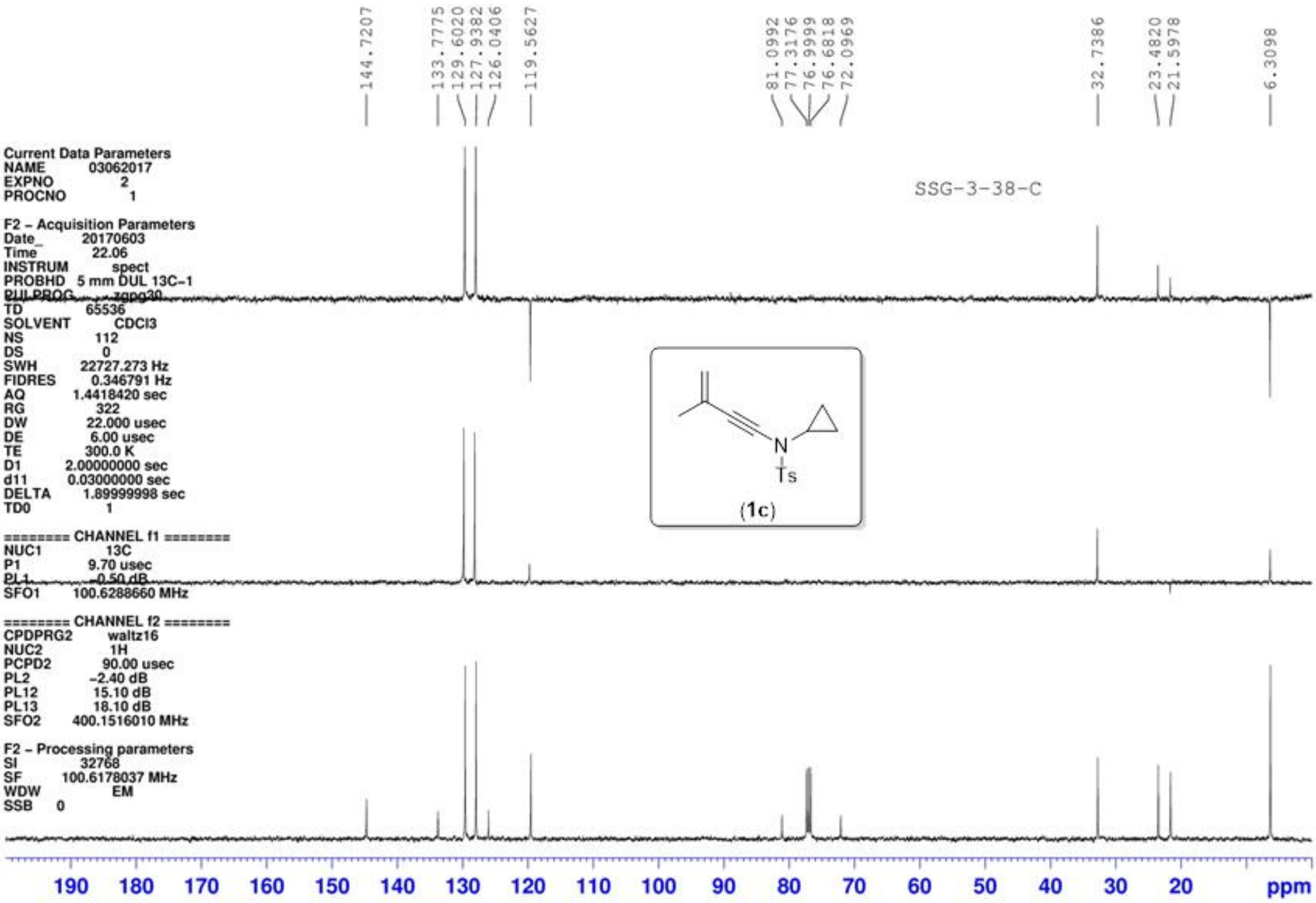
144.7207
133.7775
129.6020
127.9382
126.0406
119.5627
81.0992
77.3176
76.9999
76.6818
72.0969
32.7386
23.4820
21.5978
6.3098
SSG-3-38-C
Current Data Parameters
NAME 03062017
EXPNO 2
PROCNO 1
F2 - Acquisition Parameters
Date_ 20170603
Time 22.06
INSTRUM spect
PROBHD 5 mm DUL 13C-1
PULPROG zgpg30
TD 65536
SOLVENT CDCl3
NS 112
DS 0
SWH 22727.273 Hz
FIDRES 0.346791 Hz
AQ 1.4418420 sec
RG 322
DW 22.000 usec
DE 6.00 usec
TE 300.0 K
D1 2.00000000 sec
d11 0.03000000 sec
DELTA 1.89999998 sec
TD0 1
======== CHANNEL f1 ========
NUC1 13C
P1 9.70 usec
PL1 -0.50 dB
SFO1 100.6288660 MHz
======== CHANNEL f2 ========
CPDPRG2 waltz16
NUC2 1H
PCPD2 90.00 usec
PL2 -2.40 dB
PL12 15.10 dB
PL13 18.10 dB
SFO2 400.1516010 MHz
F2 - Processing parameters
SI 32768
SF 100.6178037 MHz
WDW EM
SSB 0
N
Ts
(1c)
190 180 170 160 150 140 130 120 110 100 90 80 70 60 50 40 30 20 ppm

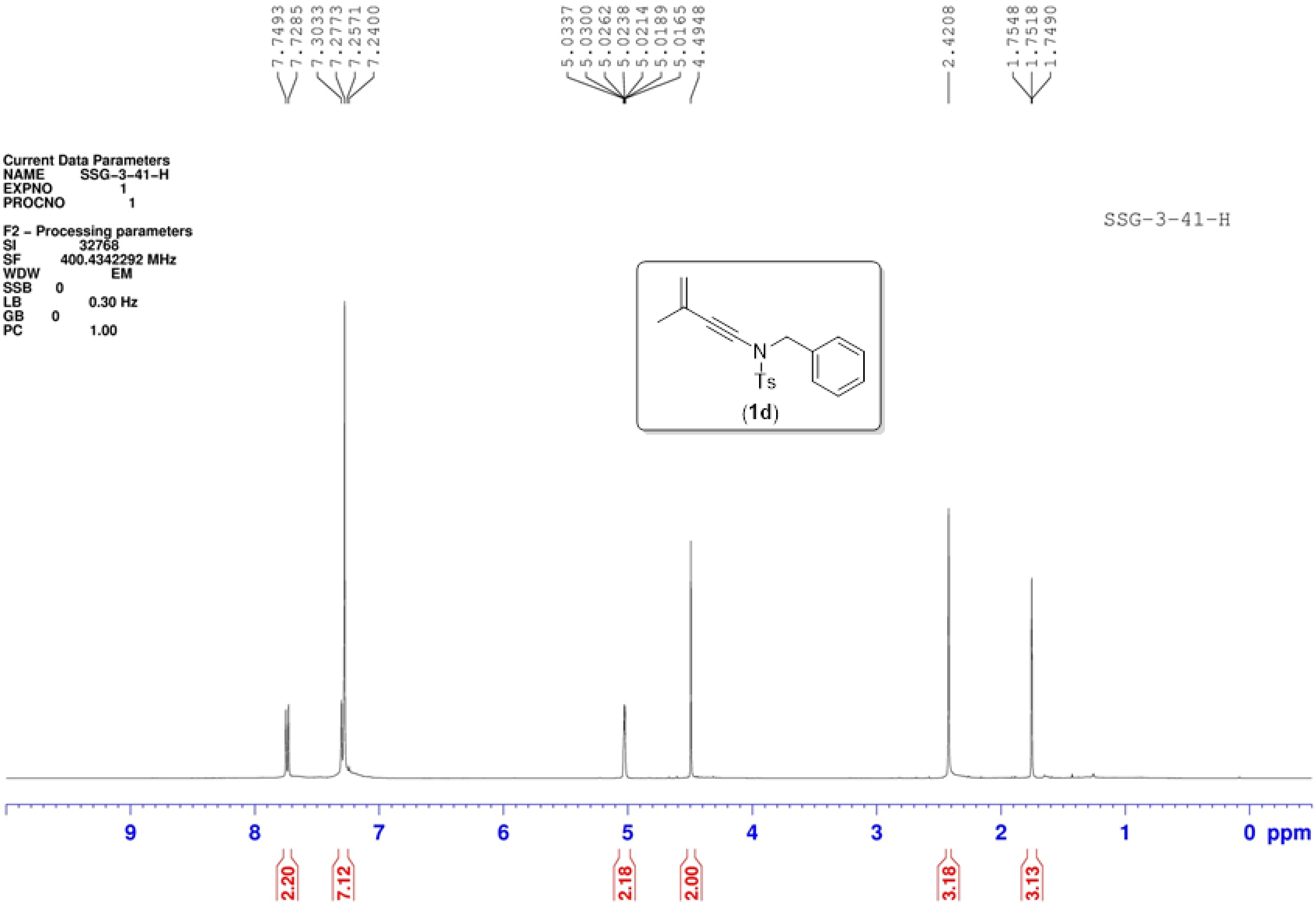
7.7493
7.7285
7.3033
7.2773
7.2571
7.2400
5.0337
5.0300
5.0262
5.0238
5.0214
5.0189
5.0165
4.4948
2.4208
1.7548
1.7518
1.7490
Current Data Parameters
NAME SSG-3-41-H
EXPNO 1
PROCNO 1
F2 - Processing parameters
SI 32768
SF 400.4342292 MHz
WDW EM
SSB 0
LB 0.30 Hz
GB 0
PC 1.00
SSG-3-41-H
N
Ts
(1d)
9
8
7
6
5
4
3
2
1
0 ppm
2.20
7.12
2.18
2.00
3.18
3.13

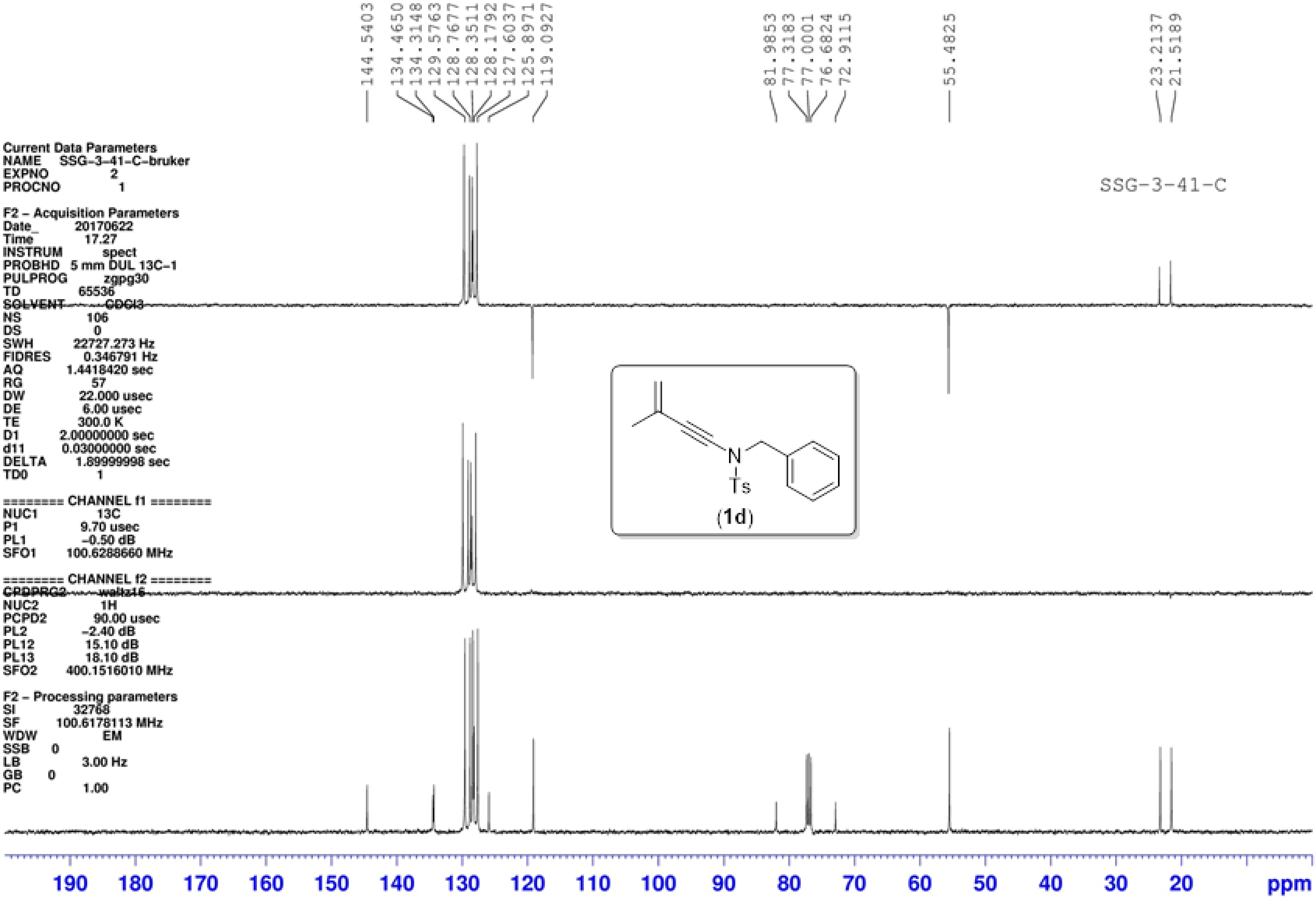
144.5403
134.4650
134.3148
129.5763
128.7677
128.3511
128.1792
127.6037
125.8971
119.0927
81.9853
77.3183
77.0001
76.6824
72.9115
55.4825
23.2137
21.5189
SSG-3-41-C
Current Data Parameters
NAME SSG-3-41-C-bruker
EXPNO 2
PROCNO 1
F2 – Acquisition Parameters
Date_ 20170622
Time 17.27
INSTRUM spect
PROBHD 5 mm DUL 13C-1
PULPROG zgpg30
TD 65536
SOLVENT CDCl3
NS 106
DS 0
SWH 22727.273 Hz
FIDRES 0.346791 Hz
AQ 1.4418420 sec
RG 57
DW 22.000 usec
DE 6.00 usec
TE 300.0 K
D1 2.00000000 sec
d11 0.03000000 sec
DELTA 1.89999998 sec
TD0 1
======== CHANNEL f1 ========
NUC1 13C
P1 9.70 usec
PL1 -0.50 dB
SFO1 100.6288660 MHz
======== CHANNEL f2 ========
CPDPRG2 waltz16
NUC2 1H
PCPD2 90.00 usec
PL2 -2.40 dB
PL12 15.10 dB
PL13 18.10 dB
SFO2 400.1516010 MHz
F2 – Processing parameters
SI 32768
SF 100.6178113 MHz
WDW EM
SSB 0
LB 3.00 Hz
GB 0
PC 1.00
N
Ts
(1d)
190 180 170 160 150 140 130 120 110 100 90 80 70 60 50 40 30 20 ppm

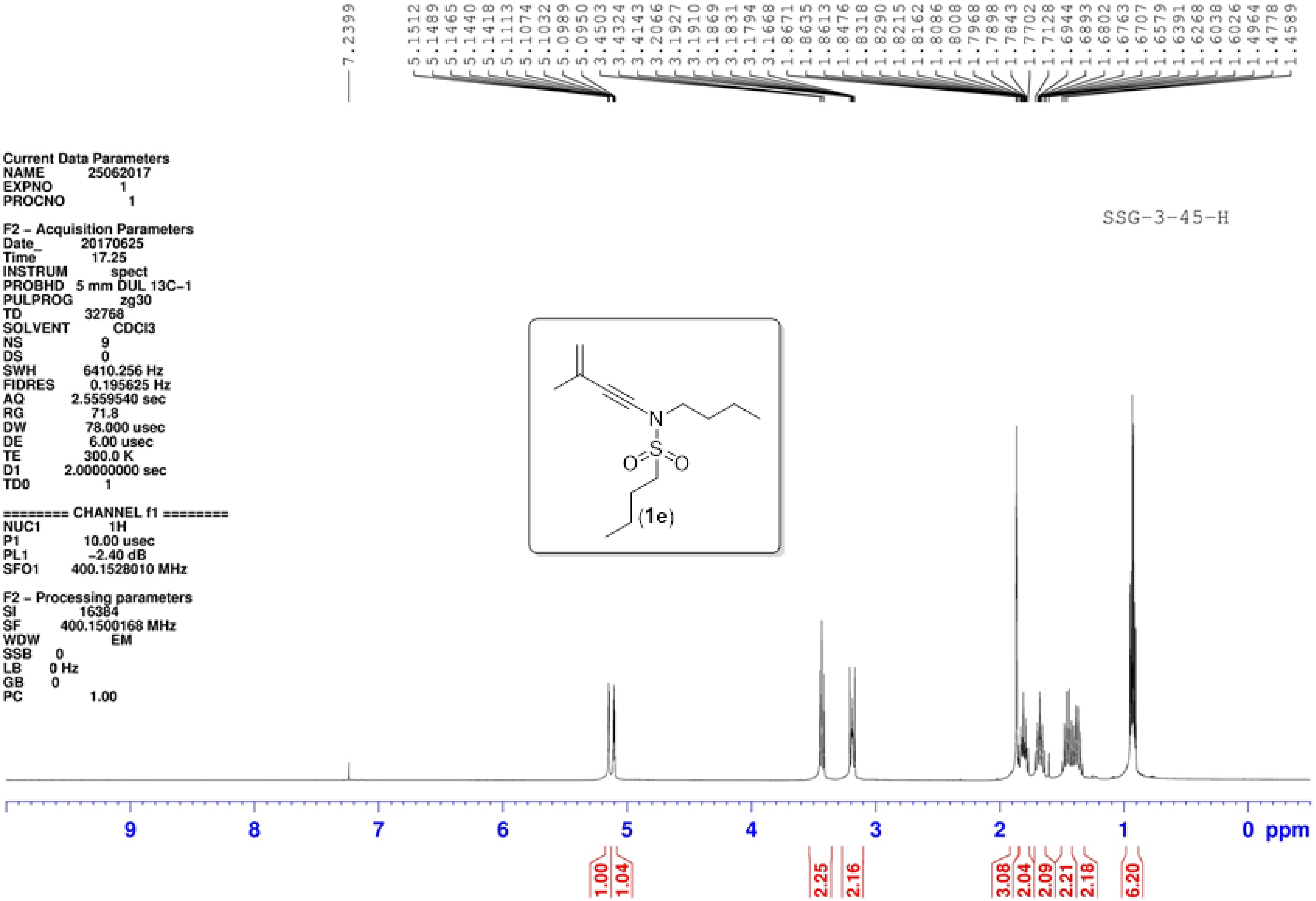
7.2399
5.1512 5.1489 5.1465 5.1440 5.1418 5.1113 5.1074 5.1032 5.0989 5.0950
3.4503 3.4324 3.4143 3.2066 3.1927 3.1910 3.1869 3.1831 3.1794 3.1668
1.8671 1.8635 1.8613 1.8476 1.8318 1.8290 1.8215 1.8162 1.8086 1.8008 1.7968 1.7898 1.7843 1.7702 1.7128 1.6944 1.6893 1.6802 1.6763 1.6707 1.6579 1.6391 1.6268 1.6038 1.6026 1.4964 1.4778 1.4589
Current Data Parameters
NAME 25062017
EXPNO 1
PROCNO 1
F2 - Acquisition Parameters
Date_ 20170625
Time 17.25
INSTRUM spect
PROBHD 5 mm DUL 13C-1
PULPROG zg30
TD 32768
SOLVENT CDCl3
NS 9
DS 0
SWH 6410.256 Hz
FIDRES 0.195625 Hz
AQ 2.5559540 sec
RG 71.8
DW 78.000 usec
DE 6.00 usec
TE 300.0 K
D1 2.00000000 sec
TD0 1
======== CHANNEL f1 ========
NUC1 1H
P1 10.00 usec
PL1 -2.40 dB
SFO1 400.1528010 MHz
F2 - Processing parameters
SI 16384
SF 400.1500168 MHz
WDW EM
SSB 0
LB 0 Hz
GB 0
PC 1.00
SSG-3-45-H
N
S
O
O
(1e)
9 8 7 6 5 4 3 2 1 0 ppm
1.00 1.04 2.25 2.16 3.08 2.04 2.09 2.21 2.18 6.20

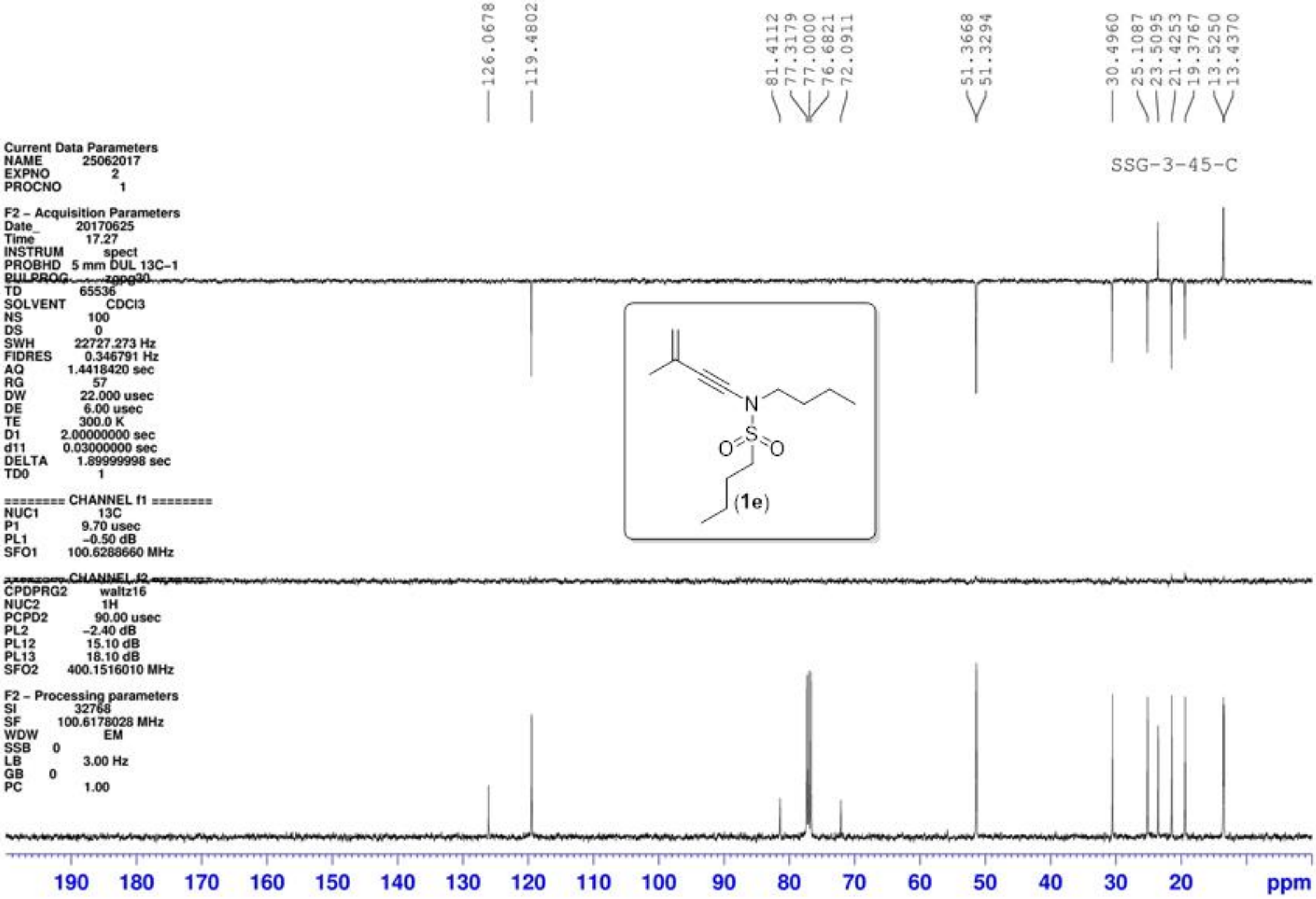
126.0678
119.4802
81.4112
77.3179
77.0000
76.6821
72.0911
51.3668
51.3294
30.4960
25.1087
23.5095
21.4253
19.3767
13.5250
13.4370
SSG-3-45-C
Current Data Parameters
NAME 25062017
EXPNO 2
PROCNO 1
F2 - Acquisition Parameters
Date_ 20170625
Time 17.27
INSTRUM spect
PROBHD 5 mm DUL 13C-1
PULPROG zgpg30
TD 65536
SOLVENT CDCl3
NS 100
DS 0
SWH 22727.273 Hz
FIDRES 0.346791 Hz
AQ 1.4418420 sec
RG 57
DW 22.000 usec
DE 6.00 usec
TE 300.0 K
D1 2.00000000 sec
d11 0.03000000 sec
DELTA 1.89999998 sec
TD0 1
======== CHANNEL f1 ========
NUC1 13C
P1 9.70 usec
PL1 -0.50 dB
SFO1 100.6288660 MHz
======== CHANNEL f2 ========
CPDPRG2 waltz16
NUC2 1H
PCPD2 90.00 usec
PL2 -2.40 dB
PL12 15.10 dB
PL13 18.10 dB
SFO2 400.1516010 MHz
F2 - Processing parameters
SI 32768
SF 100.6178028 MHz
WDW EM
SSB 0
LB 3.00 Hz
GB 0
PC 1.00
N
S
O
O
(1e)
190
180
170
160
150
140
130
120
110
100
90
80
70
60
50
40
30
20
ppm

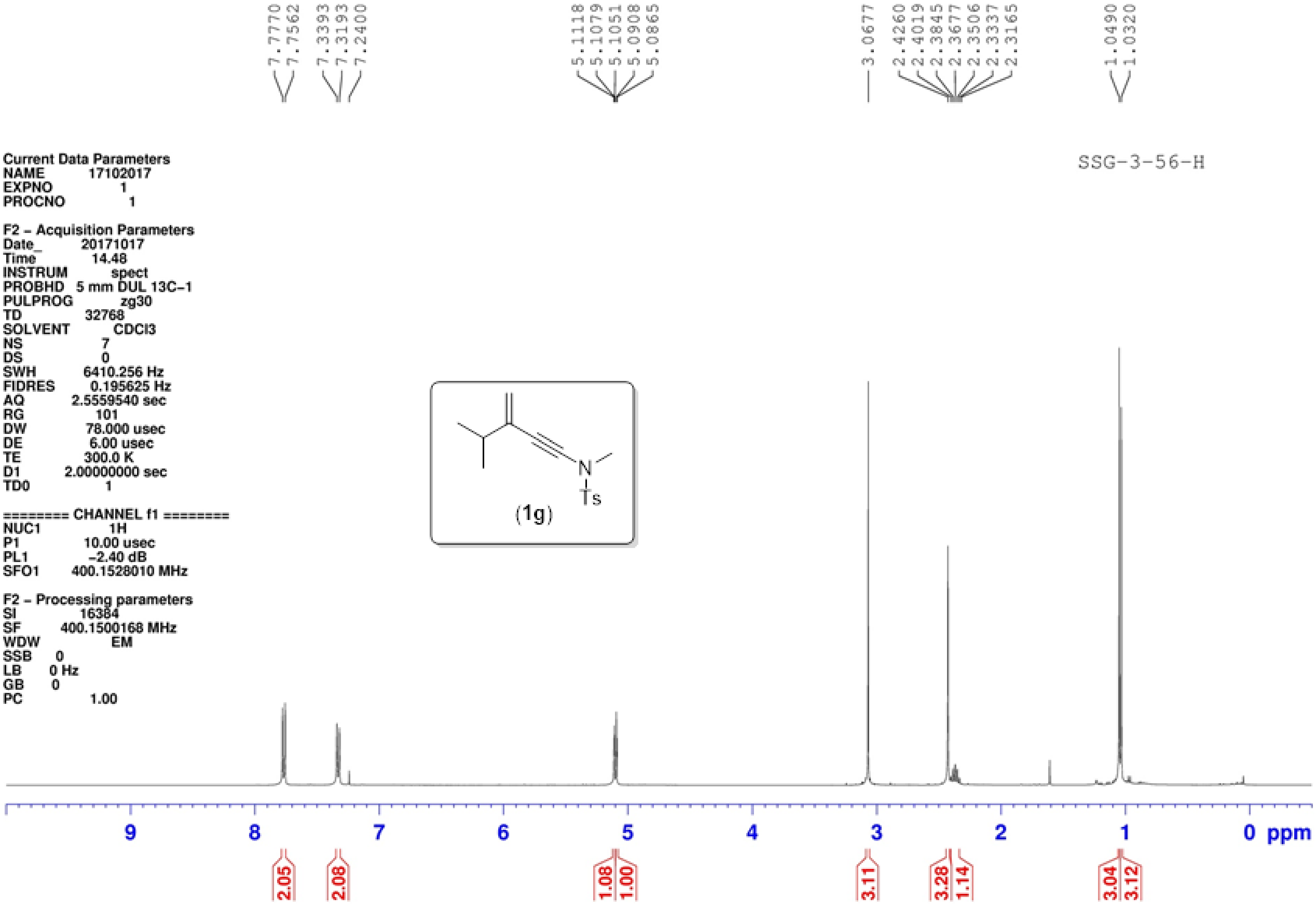
7.7770
7.7562
7.3393
7.3193
7.2400
5.1118
5.1079
5.1051
5.0908
5.0865
3.0677
2.4260
2.4019
2.3845
2.3677
2.3506
2.3337
2.3165
1.0490
1.0320
Current Data Parameters
NAME 17102017
EXPNO 1
PROCNO 1
F2 - Acquisition Parameters
Date_ 20171017
Time 14.48
INSTRUM spect
PROBHD 5 mm DUL 13C-1
PULPROG zg30
TD 32768
SOLVENT CDCl3
NS 7
DS 0
SWH 6410.256 Hz
FIDRES 0.195625 Hz
AQ 2.5559540 sec
RG 101
DW 78.000 usec
DE 6.00 usec
TE 300.0 K
D1 2.00000000 sec
TD0 1
======== CHANNEL f1 ========
NUC1 1H
P1 10.00 usec
PL1 -2.40 dB
SFO1 400.1528010 MHz
F2 - Processing parameters
SI 16384
SF 400.1500168 MHz
WDW EM
SSB 0
LB 0 Hz
GB 0
PC 1.00
SSG-3-56-H
N
Ts
(1g)
9
8
7
6
5
4
3
2
1
0 ppm
2.05
2.08
1.08
1.00
3.11
3.28
1.14
3.04
3.12

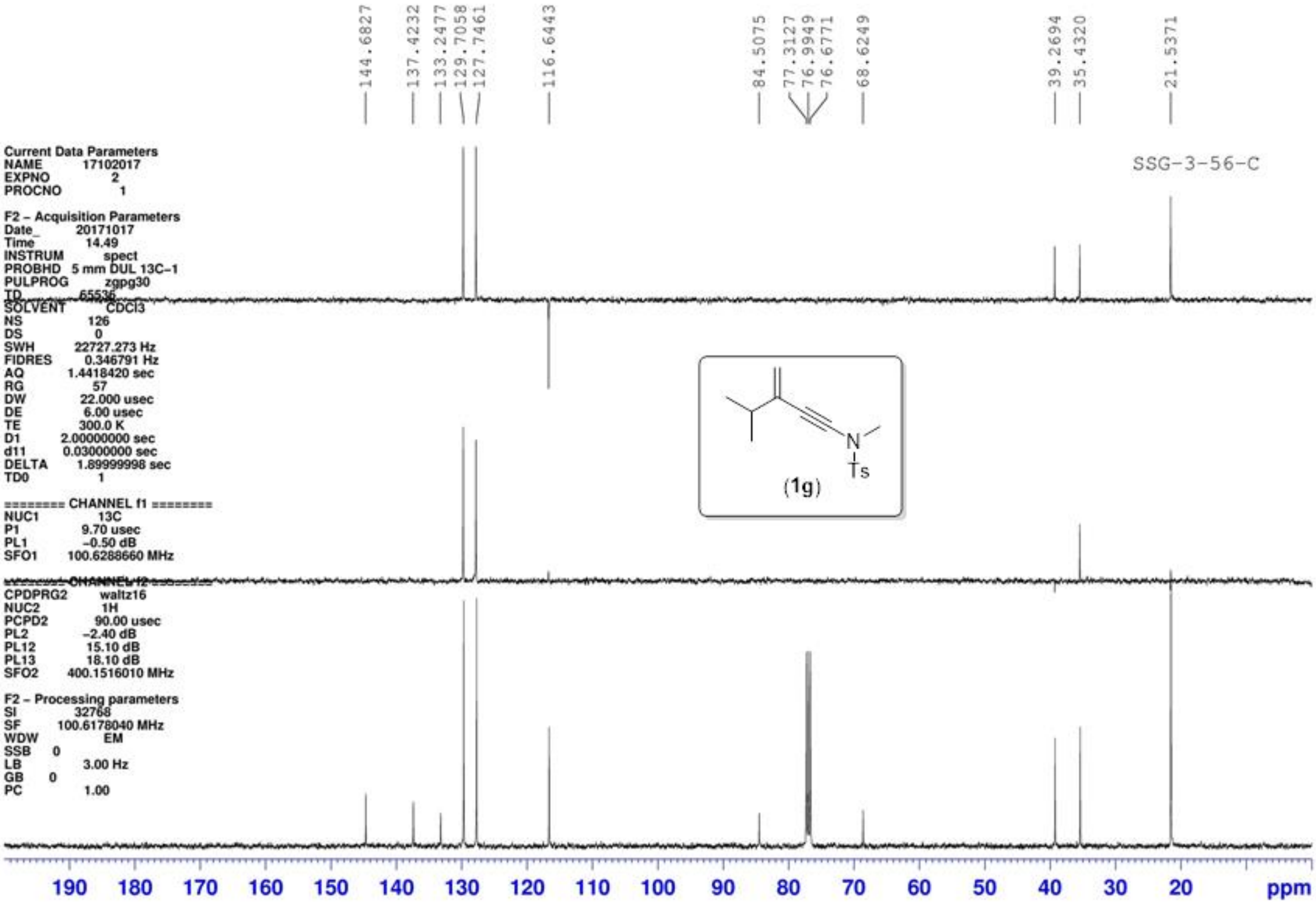

144.6827
137.4232
133.2477
129.7058
127.7461
116.6443
84.5075
77.3127
76.9949
76.6771
68.6249
39.2694
35.4320
21.5371
SSG-3-56-C
Current Data Parameters
NAME 17102017
EXPNO 2
PROCNO 1
F2 - Acquisition Parameters
Date_ 20171017
Time 14.49
INSTRUM spect
PROBHD 5 mm DUL 13C-1
PULPROG zgpg30
TD 65536
SOLVENT CDCl3
NS 126
DS 0
SWH 22727.273 Hz
FIDRES 0.346791 Hz
AQ 1.4418420 sec
RG 57
DW 22.000 usec
DE 6.00 usec
TE 300.0 K
D1 2.00000000 sec
d11 0.03000000 sec
DELTA 1.89999998 sec
TD0 1
======== CHANNEL f1 ========
NUC1 13C
P1 9.70 usec
PL1 -0.50 dB
SFO1 100.6288660 MHz
======== CHANNEL f2 ========
CPDPRG2 waltz16
NUC2 1H
PCPD2 90.00 usec
PL2 -2.40 dB
PL12 15.10 dB
PL13 18.10 dB
SFO2 400.1516010 MHz
F2 - Processing parameters
SI 32768
SF 100.6178040 MHz
WDW EM
SSB 0
LB 3.00 Hz
GB 0
PC 1.00
N
Ts
(1g)
190 180 170 160 150 140 130 120 110 100 90 80 70 60 50 40 30 20 ppm

Current Data Parameters
NAME 09092017
EXPNO 2
PROCNO 1

F2 - Acquisition Parameters
Date_ 20170909
Time 22.39
INSTRUM spect
PROBHD 5 mm DUL 13C-1
PULPROG zg30
TD 32768
SOLVENT CDCl3
NS 10
DS 0
SWH 6410.256 Hz
FIDRES 0.195625 Hz
AQ 2.5559540 sec
RG 101
DW 78.000 usec
DE 6.00 usec
TE 300.0 K
D1 2.00000000 sec
TD0 1

======== CHANNEL f1 ========
NUC1 1H
P1 10.00 usec
PL1 -2.40 dB
SFO1 400.1528010 MHz

F2 - Processing parameters
SI 16384
SF 400.1500166 MHz
WDW EM
SSB 0
LB 0 Hz
GB 0
PC 1.00

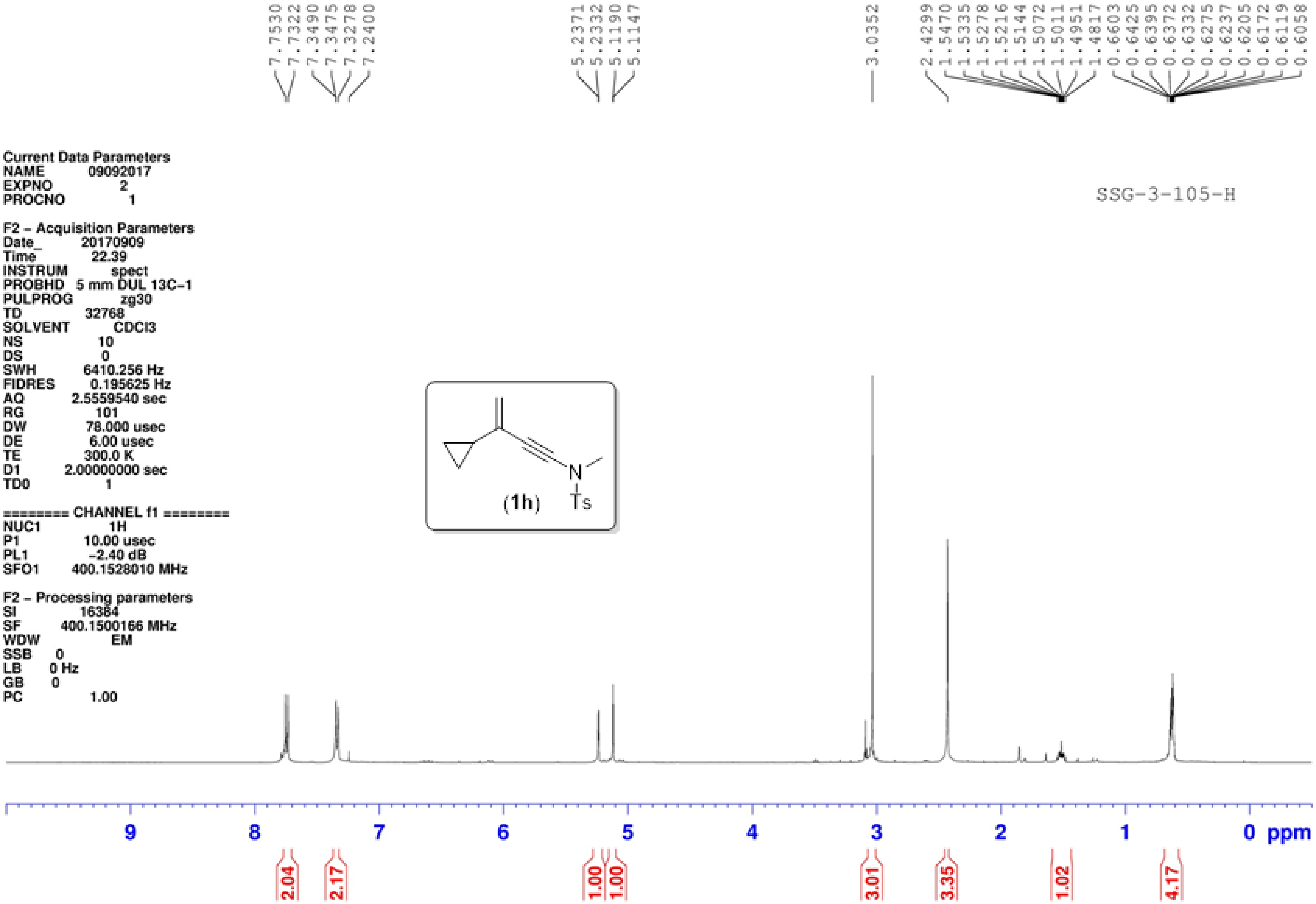

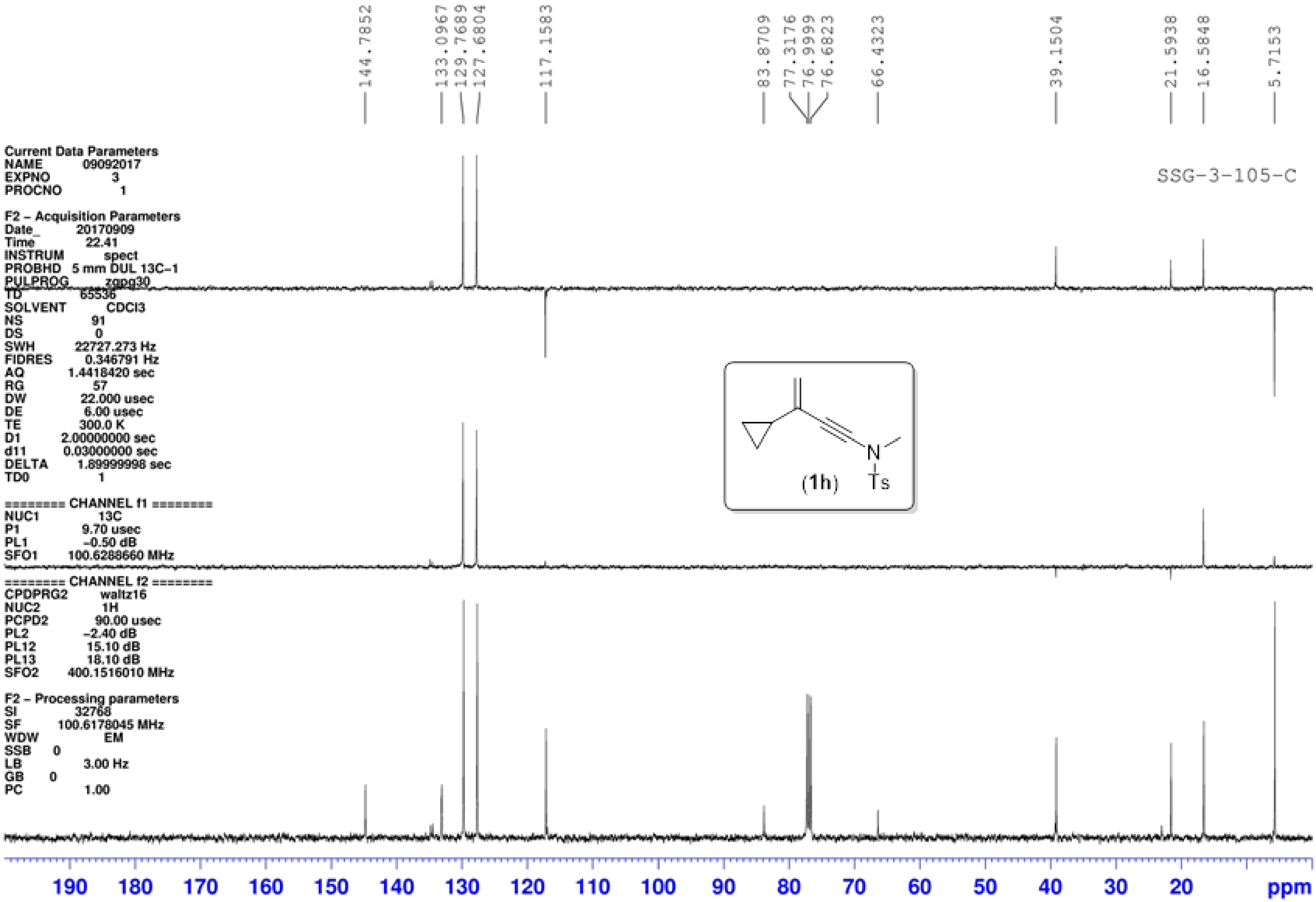
144.7852
133.0967
129.7689
127.6804
117.1583
83.8709
77.3176
76.9999
76.6823
66.4323
39.1504
21.5938
16.5848
5.7153
SSG-3-105-C
Current Data Parameters
NAME 09092017
EXPNO 3
PROCNO 1
F2 – Acquisition Parameters
Date_ 20170909
Time 22.41
INSTRUM spect
PROBHD 5 mm DUL 13C-1
PULPROG zgpg30
TD 65536
SOLVENT CDCl3
NS 91
DS 0
SWH 22727.273 Hz
FIDRES 0.346791 Hz
AQ 1.4418420 sec
RG 57
DW 22.000 usec
DE 6.00 usec
TE 300.0 K
D1 2.00000000 sec
d11 0.03000000 sec
DELTA 1.89999998 sec
TD0 1
======== CHANNEL f1 ========
NUC1 13C
P1 9.70 usec
PL1 -0.50 dB
SFO1 100.6288660 MHz
======== CHANNEL f2 ========
CPDPRG2 waltz16
NUC2 1H
PCPD2 90.00 usec
PL2 -2.40 dB
PL12 15.10 dB
PL13 18.10 dB
SFO2 400.1516010 MHz
F2 - Processing parameters
SI 32768
SF 100.6178045 MHz
WDW EM
SSB 0
LB 3.00 Hz
GB 0
PC 1.00
N
Ts
(1h)
190
180
170
160
150
140
130
120
110
100
90
80
70
60
50
40
30
20
ppm

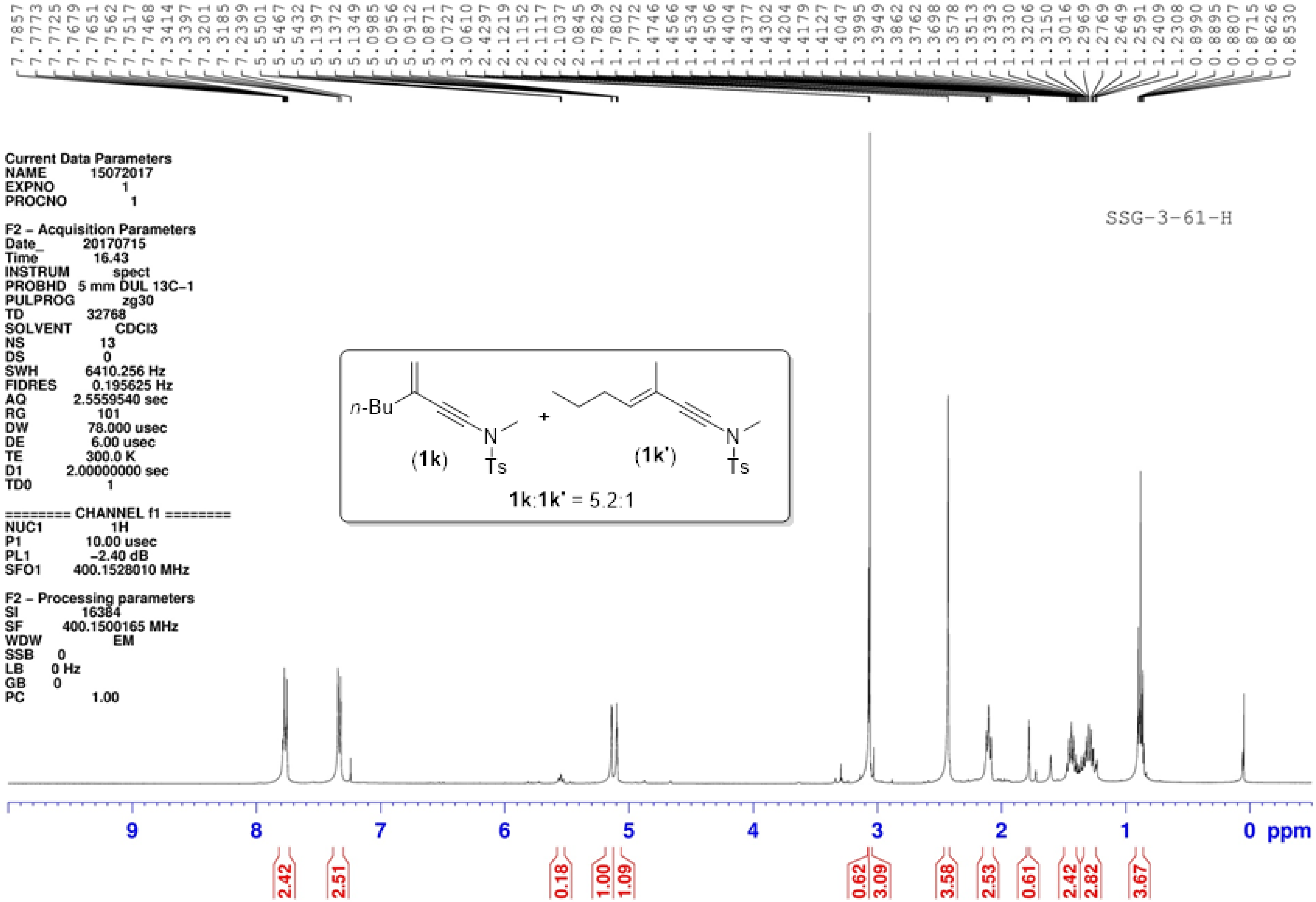
7.7857
7.7773
7.7725
7.7679
7.7651
7.7562
7.7517
7.7468
7.3414
7.3397
7.3201
7.3185
7.2399
5.5501
5.5467
5.5432
5.1397
5.1372
5.1349
5.0985
5.0956
5.0912
5.0871
3.0727
3.0610
2.4297
2.1219
2.1152
2.1117
2.1037
2.0845
1.7829
1.7802
1.7772
1.4746
1.4566
1.4534
1.4506
1.4404
1.4377
1.4302
1.4204
1.4179
1.4127
1.4047
1.3995
1.3949
1.3862
1.3762
1.3698
1.3578
1.3513
1.3393
1.3330
1.3206
1.3150
1.3016
1.2969
1.2769
1.2649
1.2591
1.2409
1.2308
0.8990
0.8895
0.8807
0.8715
0.8626
0.8530
Current Data Parameters
NAME 15072017
EXPNO 1
PROCNO 1
F2 - Acquisition Parameters
Date_ 20170715
Time 16.43
INSTRUM spect
PROBHD 5 mm DUL 13C-1
PULPROG zg30
TD 32768
SOLVENT CDCl3
NS 13
DS 0
SWH 6410.256 Hz
FIDRES 0.195625 Hz
AQ 2.5559540 sec
RG 101
DW 78.000 usec
DE 6.00 usec
TE 300.0 K
D1 2.00000000 sec
TD0 1
======== CHANNEL f1 ========
NUC1 1H
P1 10.00 usec
PL1 -2.40 dB
SFO1 400.1528010 MHz
F2 - Processing parameters
SI 16384
SF 400.1500165 MHz
WDW EM
SSB 0
LB 0 Hz
GB 0
PC 1.00
SSG-3-61-H
n-Bu
N
Ts
(1k)
+
(1k')
N
Ts
1k:1k' = 5.2:1
9
8
7
6
5
4
3
2
1
0 ppm
2.42
2.51
0.18
1.00
1.09
0.62
3.09
3.58
2.53
0.61
2.42
2.82
3.67

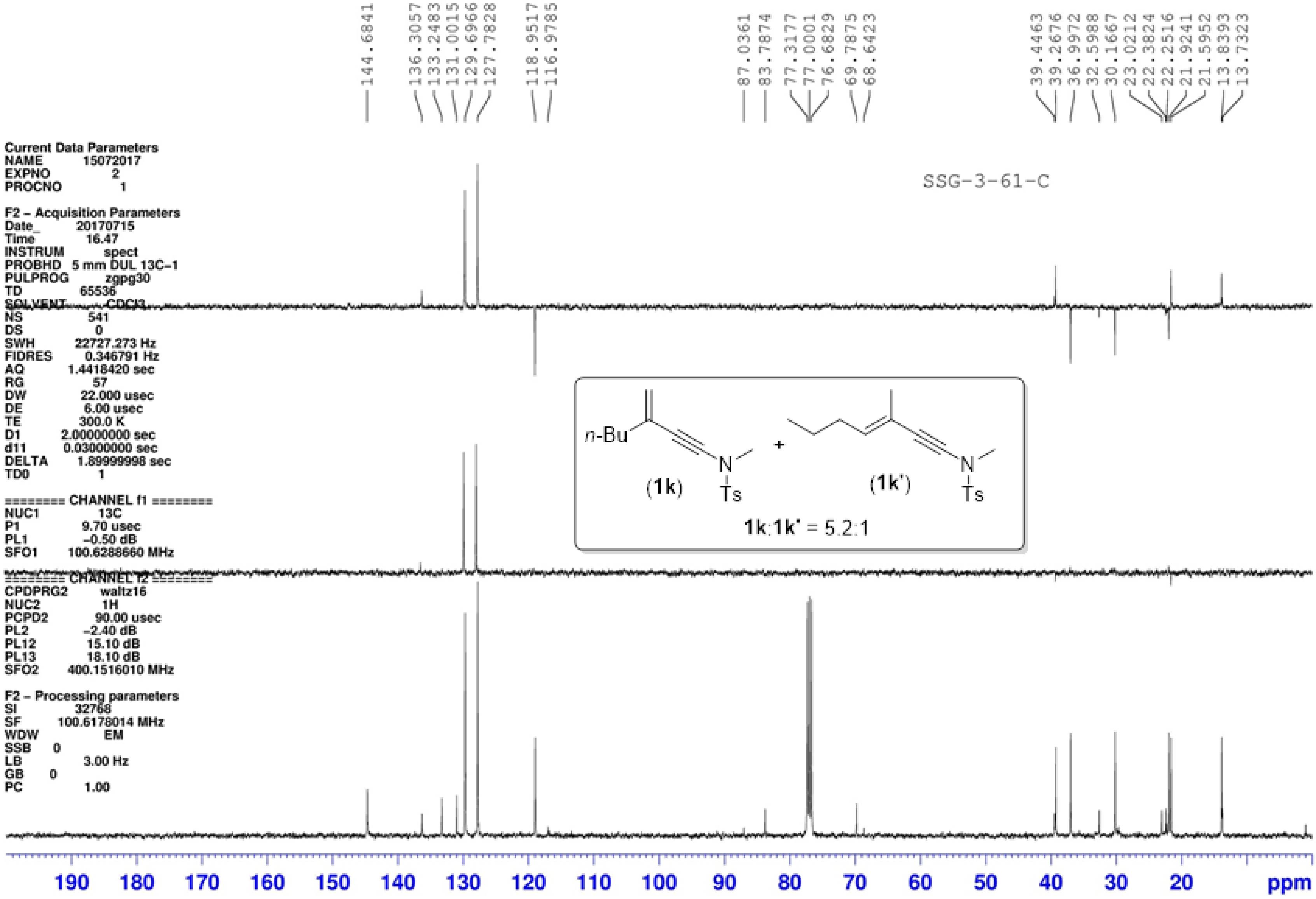

144.6841
136.3057
133.2483
131.0015
129.6966
127.7828
118.9517
116.9785
87.0361
83.7874
77.3177
77.0001
76.6829
69.7875
68.6423
39.4463
39.2676
36.9972
32.5988
30.1667
23.0212
22.3824
22.2516
21.9241
21.5952
13.8393
13.7323
SSG-3-61-C
Current Data Parameters
NAME 15072017
EXPNO 2
PROCNO 1
F2 – Acquisition Parameters
Date_ 20170715
Time 16.47
INSTRUM spect
PROBHD 5 mm DUL 13C-1
PULPROG zgpg30
TD 65536
SOLVENT CDCl3
NS 541
DS 0
SWH 22727.273 Hz
FIDRES 0.346791 Hz
AQ 1.4418420 sec
RG 57
DW 22.000 usec
DE 6.00 usec
TE 300.0 K
D1 2.00000000 sec
d11 0.03000000 sec
DELTA 1.89999998 sec
TD0 1
======== CHANNEL f1 ========
NUC1 13C
P1 9.70 usec
PL1 -0.50 dB
SFO1 100.6288660 MHz
======== CHANNEL f2 ========
CPDPRG2 waltz16
NUC2 1H
PCPD2 90.00 usec
PL2 -2.40 dB
PL12 15.10 dB
PL13 18.10 dB
SFO2 400.1516010 MHz
F2 – Processing parameters
SI 32768
SF 100.6178014 MHz
WDW EM
SSB 0
LB 3.00 Hz
GB 0
PC 1.00
n-Bu
N
Ts
(1k)
+
N
Ts
(1k')
1k:1k' = 5.2:1
190 180 170 160 150 140 130 120 110 100 90 80 70 60 50 40 30 20 ppm

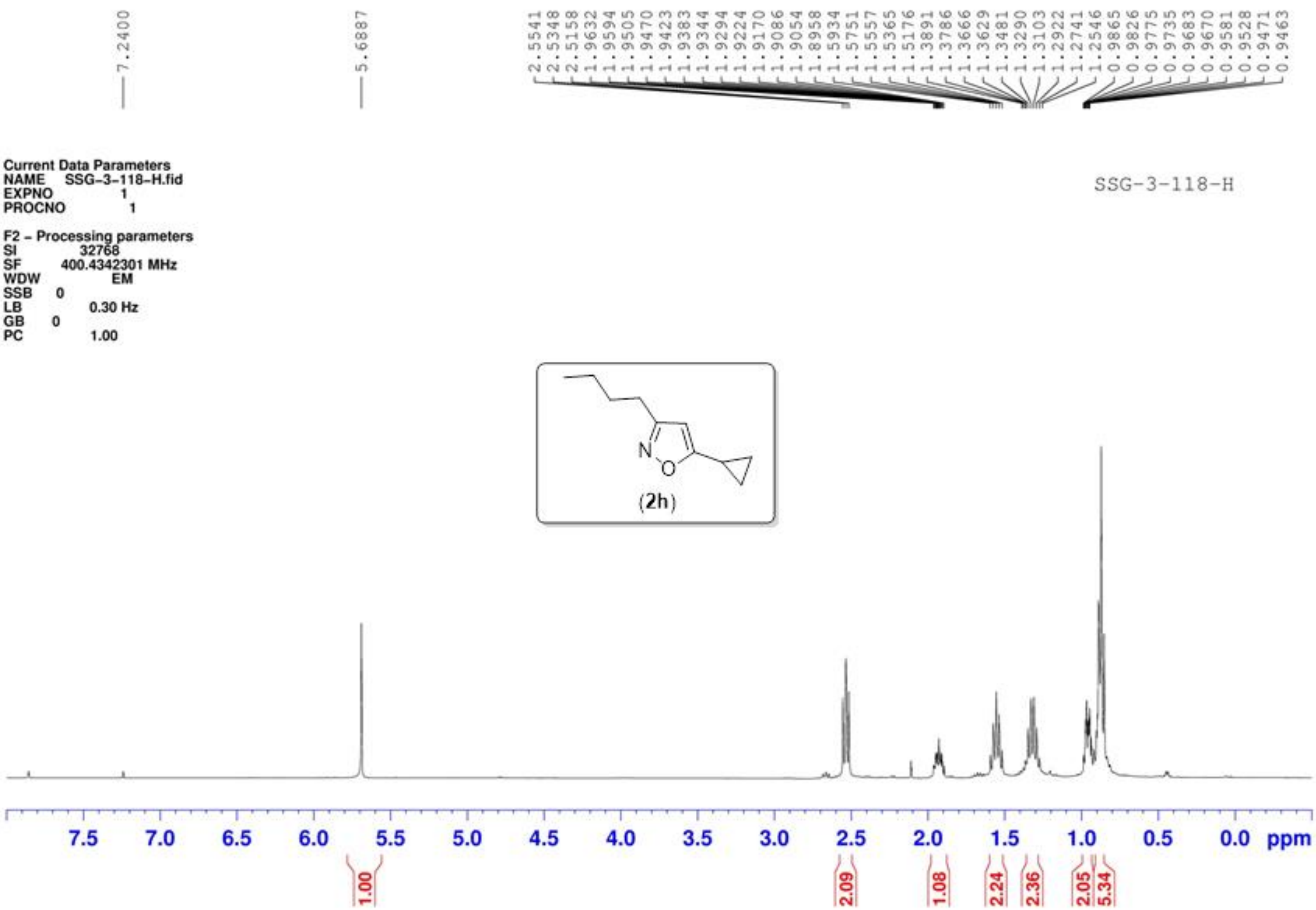
7.2400
5.6887
2.5541
2.5348
2.5158
1.9632
1.9594
1.9505
1.9470
1.9423
1.9383
1.9344
1.9294
1.9224
1.9170
1.9086
1.9054
1.8958
1.5934
1.5751
1.5557
1.5365
1.5176
1.3891
1.3786
1.3666
1.3629
1.3481
1.3290
1.3103
1.2922
1.2741
1.2546
0.9865
0.9826
0.9775
0.9735
0.9683
0.9670
0.9581
0.9528
0.9471
0.9463
Current Data Parameters
NAME SSG-3-118-H.fid
EXPNO 1
PROCNO 1
F2 - Processing parameters
SI 32768
SF 400.4342301 MHz
WDW EM
SSB 0
LB 0.30 Hz
GB 0
PC 1.00
SSG-3-118-H
N
O
(2h)
7.5
7.0
6.5
6.0
5.5
5.0
4.5
4.0
3.5
3.0
2.5
2.0
1.5
1.0
0.5
0.0 ppm
1.00
2.09
1.08
2.24
2.36
2.05
5.34

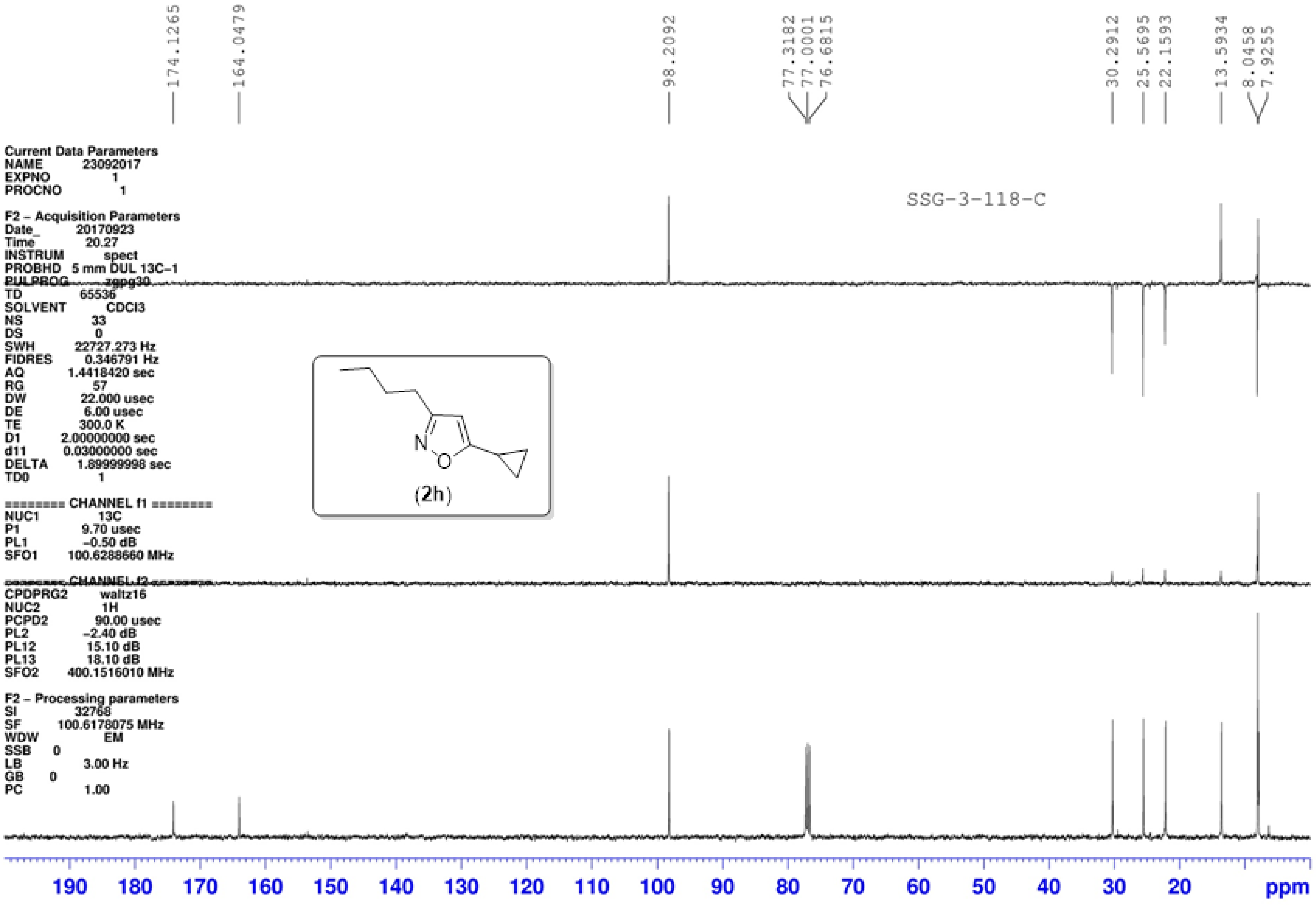
174.1265
164.0479
98.2092
77.3182
77.0001
76.6815
30.2912
25.5695
22.1593
13.5934
8.0458
7.9255
SSG-3-118-C
Current Data Parameters
NAME 23092017
EXPNO 1
PROCNO 1
F2 – Acquisition Parameters
Date_ 20170923
Time 20.27
INSTRUM spect
PROBHD 5 mm DUL 13C-1
PULPROG zgpg30
TD 65536
SOLVENT CDCl3
NS 33
DS 0
SWH 22727.273 Hz
FIDRES 0.346791 Hz
AQ 1.4418420 sec
RG 57
DW 22.000 usec
DE 6.00 usec
TE 300.0 K
D1 2.00000000 sec
d11 0.03000000 sec
DELTA 1.89999998 sec
TD0 1
======== CHANNEL f1 ========
NUC1 13C
P1 9.70 usec
PL1 -0.50 dB
SFO1 100.6288660 MHz
======== CHANNEL f2 ========
CPDPRG2 waltz16
NUC2 1H
PCPD2 90.00 usec
PL2 -2.40 dB
PL12 15.10 dB
PL13 18.10 dB
SFO2 400.1516010 MHz
F2 – Processing parameters
SI 32768
SF 100.6178075 MHz
WDW EM
SSB 0
LB 3.00 Hz
GB 0
PC 1.00
N
O
(2h)
190 180 170 160 150 140 130 120 110 100 90 80 70 60 50 40 30 20 ppm

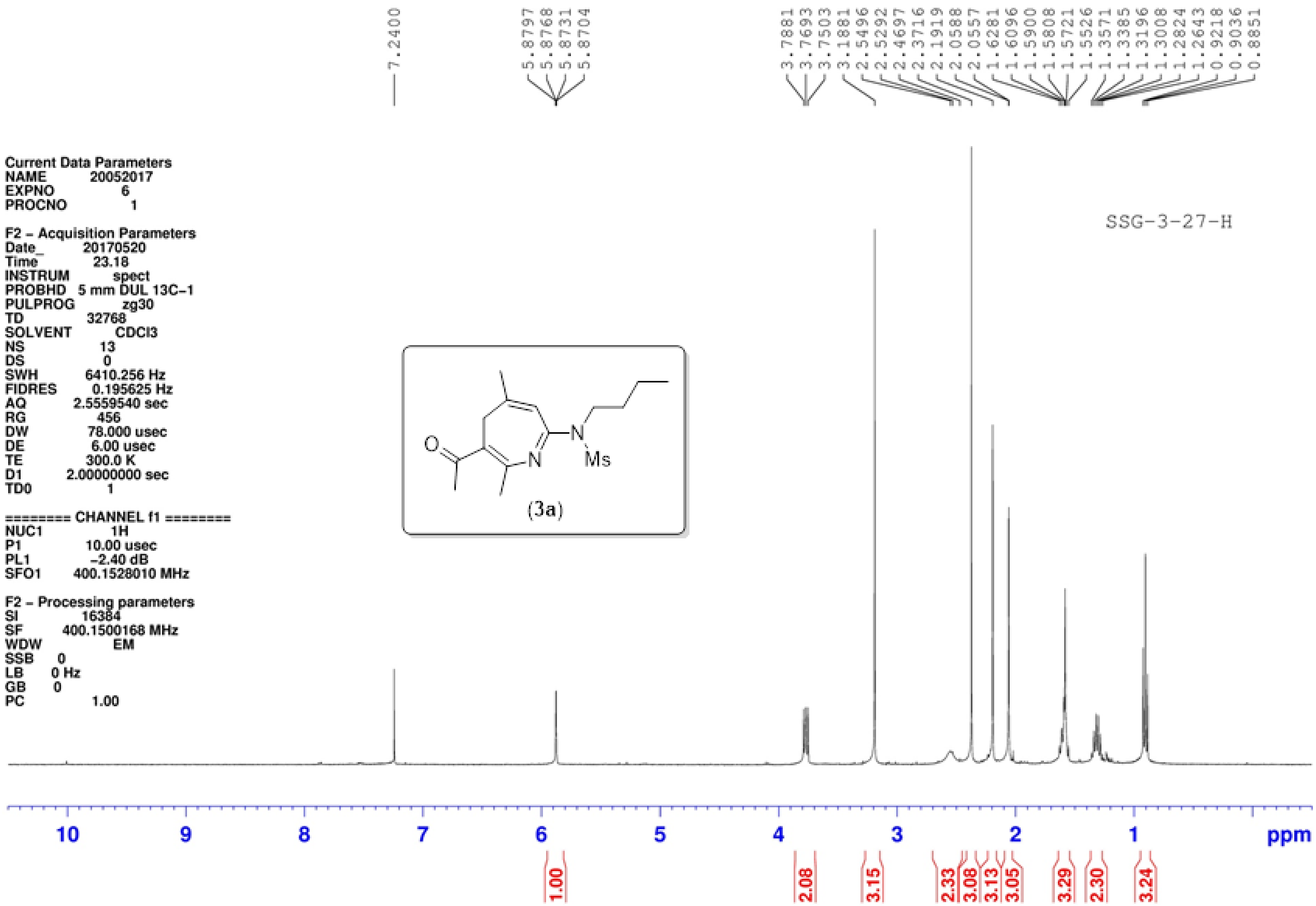
7.2400
5.8797
5.8768
5.8731
5.8704
3.7881
3.7693
3.7503
3.1881
2.5496
2.5292
2.4697
2.3716
2.1919
2.0588
2.0557
1.6281
1.6096
1.5900
1.5808
1.5721
1.5526
1.3571
1.3385
1.3196
1.3008
1.2824
1.2643
0.9218
0.9036
0.8851
SSG-3-27-H
Current Data Parameters
NAME 20052017
EXPNO 6
PROCNO 1
F2 - Acquisition Parameters
Date_ 20170520
Time 23.18
INSTRUM spect
PROBHD 5 mm DUL 13C-1
PULPROG zg30
TD 32768
SOLVENT CDCl3
NS 13
DS 0
SWH 6410.256 Hz
FIDRES 0.195625 Hz
AQ 2.5559540 sec
RG 456
DW 78.000 usec
DE 6.00 usec
TE 300.0 K
D1 2.00000000 sec
TD0 1
======== CHANNEL f1 ========
NUC1 1H
P1 10.00 usec
PL1 -2.40 dB
SFO1 400.1528010 MHz
F2 - Processing parameters
SI 16384
SF 400.1500168 MHz
WDW EM
SSB 0
LB 0 Hz
GB 0
PC 1.00
O
N
N
Ms
(3a)
10
9
8
7
6
5
4
3
2
1
ppm
1.00
2.08
3.15
2.33
3.08
3.13
3.05
3.29
2.30
3.24

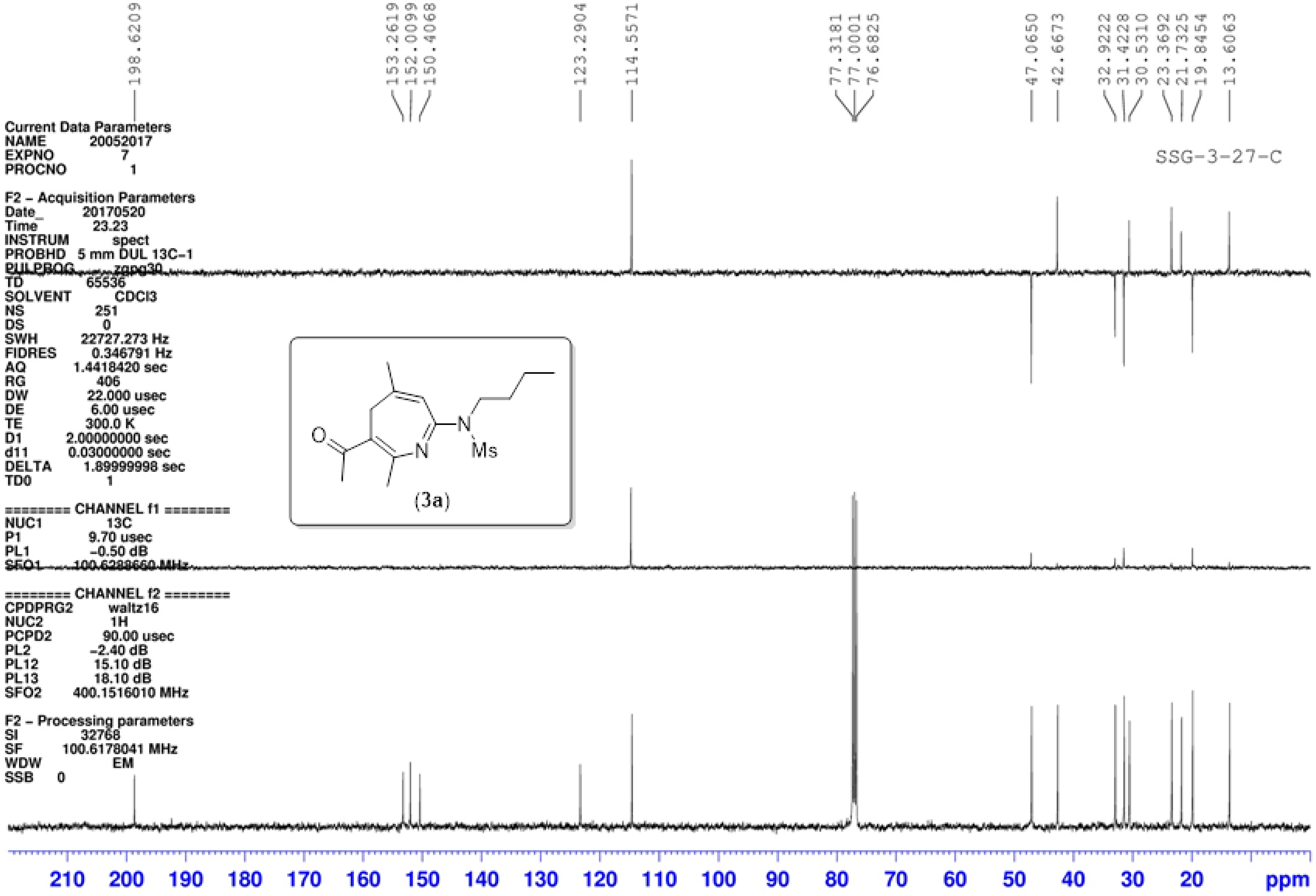
198.6209
153.2619
152.0099
150.4068
123.2904
114.5571
77.3181
77.0001
76.6825
47.0650
42.6673
32.9222
31.4228
30.5310
23.3692
21.7325
19.8454
13.6063
SSG-3-27-C
Current Data Parameters
NAME 20052017
EXPNO 7
PROCNO 1
F2 - Acquisition Parameters
Date_ 20170520
Time 23.23
INSTRUM spect
PROBHD 5 mm DUL 13C-1
PULPROG zgpg30
TD 65536
SOLVENT CDCl3
NS 251
DS 0
SWH 22727.273 Hz
FIDRES 0.346791 Hz
AQ 1.4418420 sec
RG 406
DW 22.000 usec
DE 6.00 usec
TE 300.0 K
D1 2.00000000 sec
d11 0.03000000 sec
DELTA 1.89999998 sec
TD0 1
======== CHANNEL f1 ========
NUC1 13C
P1 9.70 usec
PL1 -0.50 dB
SFO1 100.6288660 MHz
======== CHANNEL f2 ========
CPDPRG2 waltz16
NUC2 1H
PCPD2 90.00 usec
PL2 -2.40 dB
PL12 15.10 dB
PL13 18.10 dB
SFO2 400.1516010 MHz
F2 - Processing parameters
SI 32768
SF 100.6178041 MHz
WDW EM
SSB 0
O
N
N
Ms
(3a)
210 200 190 180 170 160 150 140 130 120 110 100 90 80 70 60 50 40 30 20 ppm

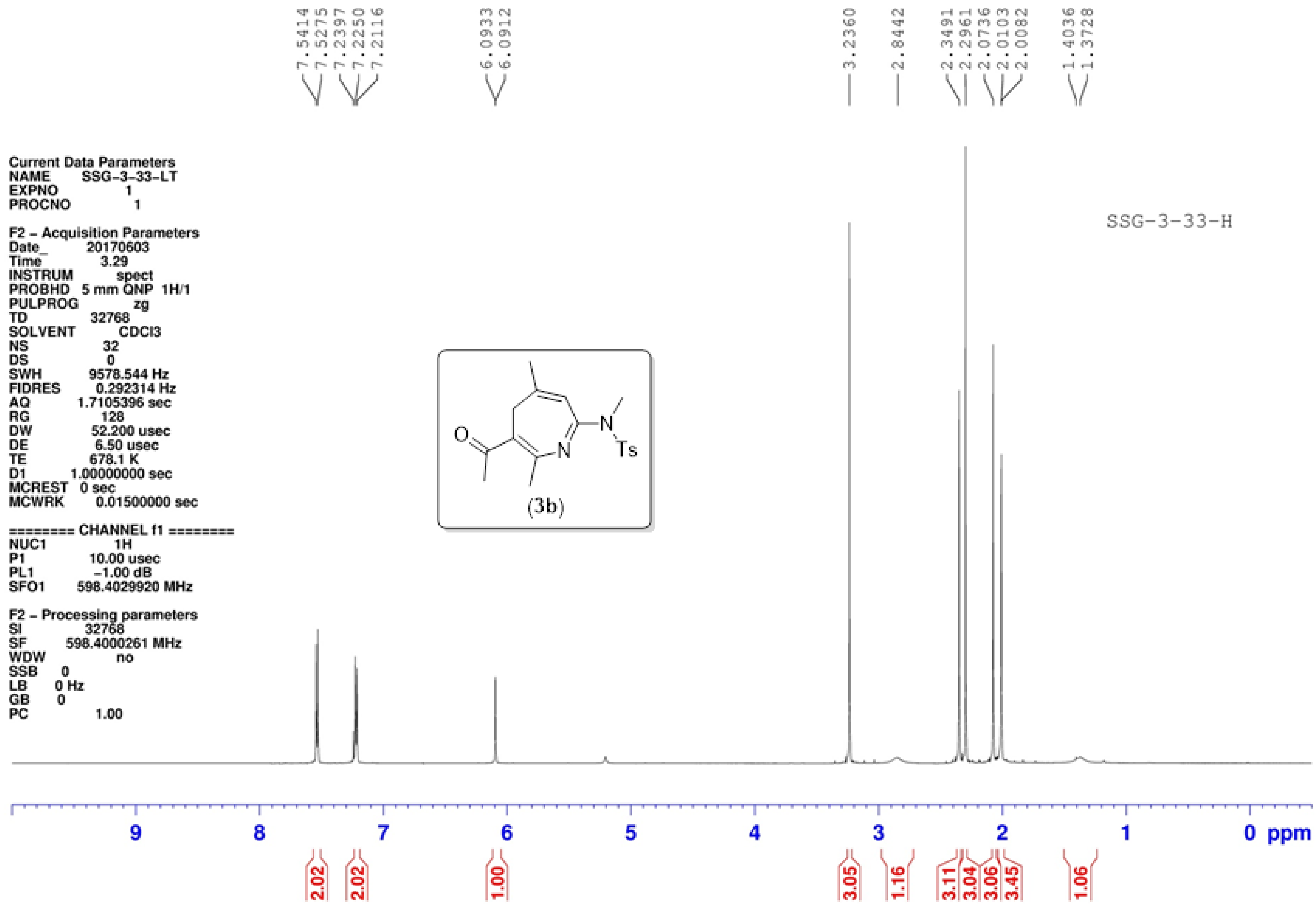
7.5414
7.5275
7.2397
7.2250
7.2116
6.0933
6.0912
3.2360
2.8442
2.3491
2.2961
2.0736
2.0103
2.0082
1.4036
1.3728
Current Data Parameters
NAME SSG-3-33-LT
EXPNO 1
PROCNO 1
F2 - Acquisition Parameters
Date_ 20170603
Time 3.29
INSTRUM spect
PROBHD 5 mm QNP 1H/1
PULPROG zg
TD 32768
SOLVENT CDCl3
NS 32
DS 0
SWH 9578.544 Hz
FIDRES 0.292314 Hz
AQ 1.7105396 sec
RG 128
DW 52.200 usec
DE 6.50 usec
TE 678.1 K
D1 1.00000000 sec
MCREST 0 sec
MCWRK 0.01500000 sec
======== CHANNEL f1 ========
NUC1 1H
P1 10.00 usec
PL1 -1.00 dB
SFO1 598.4029920 MHz
F2 - Processing parameters
SI 32768
SF 598.4000261 MHz
WDW no
SSB 0
LB 0 Hz
GB 0
PC 1.00
SSG-3-33-H
O
N
N
Ts
(3b)
9
8
7
6
5
4
3
2
1
0 ppm
2.02
2.02
1.00
3.05
1.16
3.11
3.04
3.06
3.45
1.06

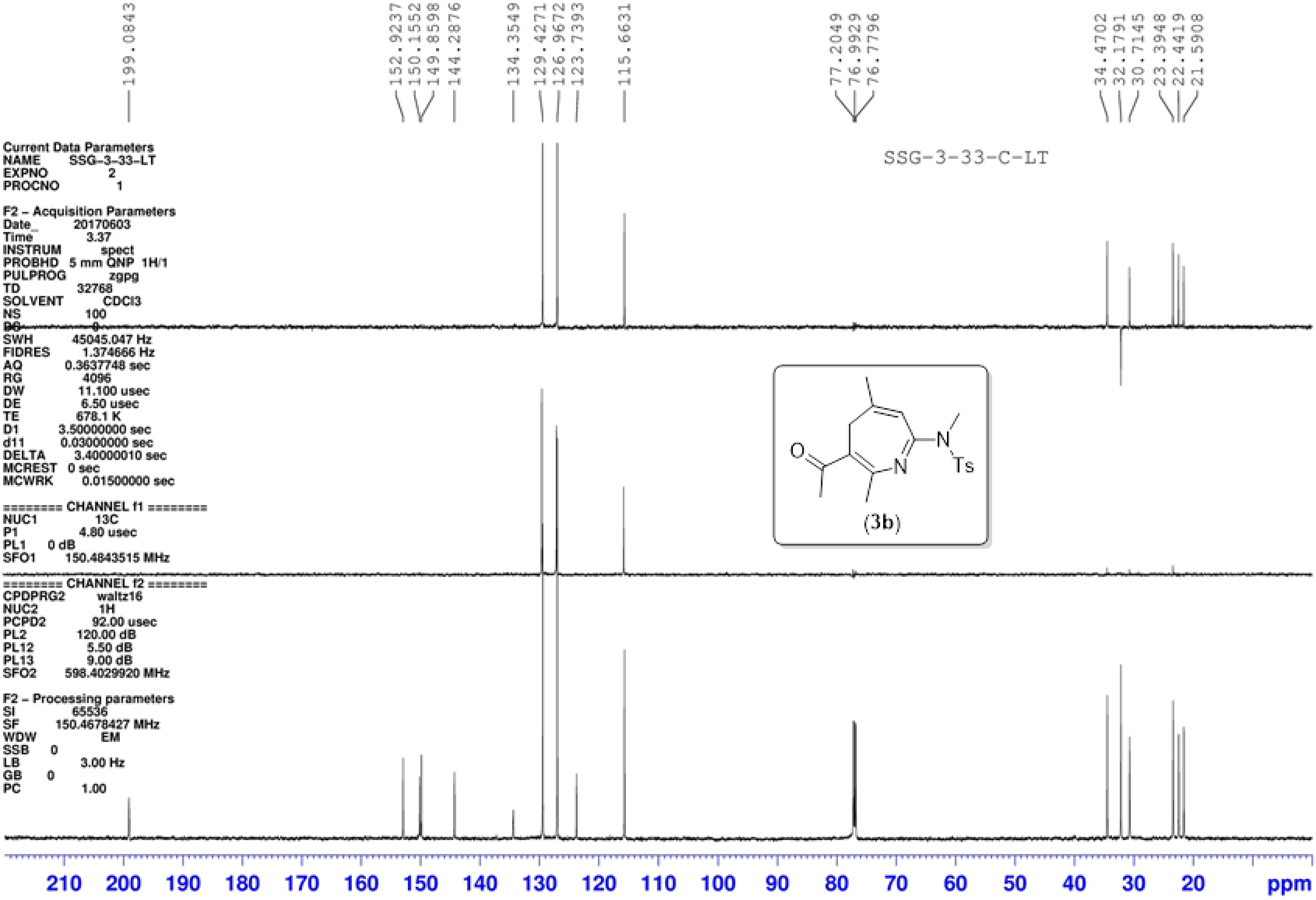
199.0843
152.9237
150.1552
149.8598
144.2876
134.3549
129.4271
126.9672
123.7393
115.6631
77.2049
76.9929
76.7796
34.4702
32.1791
30.7145
23.3948
22.4419
21.5908
SSG-3-33-C-LT
Current Data Parameters
NAME SSG-3-33-LT
EXPNO 2
PROCNO 1
F2 - Acquisition Parameters
Date_ 20170603
Time 3.37
INSTRUM spect
PROBHD 5 mm QNP 1H/1
PULPROG zgpg
TD 32768
SOLVENT CDCl3
NS 100
SWH 45045.047 Hz
FIDRES 1.374666 Hz
AQ 0.3637748 sec
RG 4096
DW 11.100 usec
DE 6.50 usec
TE 678.1 K
D1 3.50000000 sec
d11 0.03000000 sec
DELTA 3.40000010 sec
MCREST 0 sec
MCWRK 0.01500000 sec
======== CHANNEL f1 ========
NUC1 13C
P1 4.80 usec
PL1 0 dB
SFO1 150.4843515 MHz
======== CHANNEL f2 ========
CPDPRG2 waltz16
NUC2 1H
PCPD2 92.00 usec
PL2 120.00 dB
PL12 5.50 dB
PL13 9.00 dB
SFO2 598.4029920 MHz
F2 - Processing parameters
SI 65536
SF 150.4678427 MHz
WDW EM
SSB 0
LB 3.00 Hz
GB 0
PC 1.00
O
N
N
Ts
(3b)
210 200 190 180 170 160 150 140 130 120 110 100 90 80 70 60 50 40 30 20 ppm

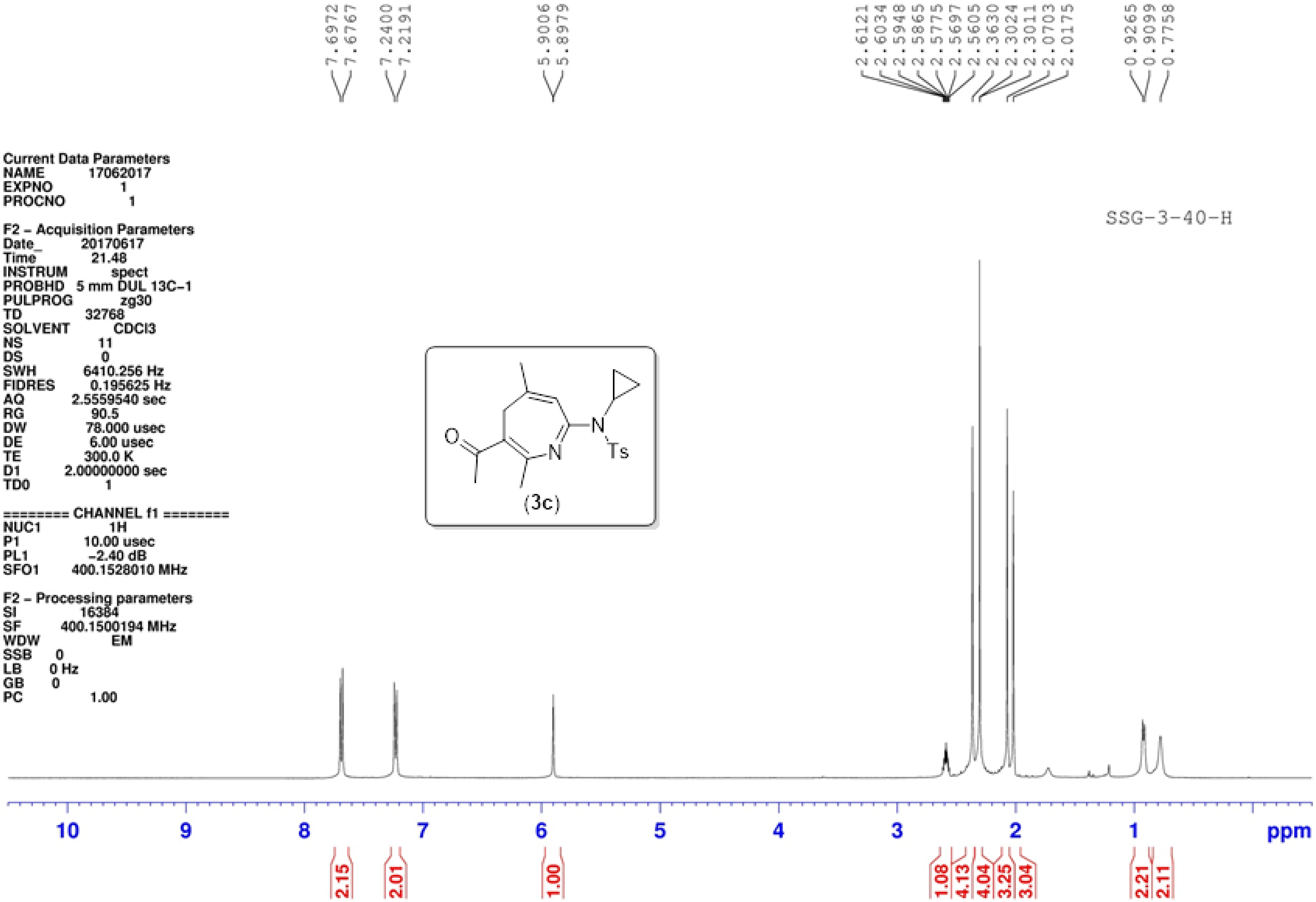
7.6972
7.6767
7.2400
7.2191
5.9006
5.8979
2.6121
2.6034
2.5948
2.5865
2.5775
2.5697
2.5605
2.3630
2.3024
2.3011
2.0703
2.0175
0.9265
0.9099
0.7758
Current Data Parameters
NAME 17062017
EXPNO 1
PROCNO 1
F2 - Acquisition Parameters
Date_ 20170617
Time 21.48
INSTRUM spect
PROBHD 5 mm DUL 13C-1
PULPROG zg30
TD 32768
SOLVENT CDCl3
NS 11
DS 0
SWH 6410.256 Hz
FIDRES 0.195625 Hz
AQ 2.5559540 sec
RG 90.5
DW 78.000 usec
DE 6.00 usec
TE 300.0 K
D1 2.00000000 sec
TD0 1
======== CHANNEL f1 ========
NUC1 1H
P1 10.00 usec
PL1 -2.40 dB
SFO1 400.1528010 MHz
F2 - Processing parameters
SI 16384
SF 400.1500194 MHz
WDW EM
SSB 0
LB 0 Hz
GB 0
PC 1.00
SSG-3-40-H
O
N
N
Ts
(3c)
10
9
8
7
6
5
4
3
2
1
ppm
2.15
2.01
1.00
1.08
4.13
4.04
3.25
3.04
2.21
2.11

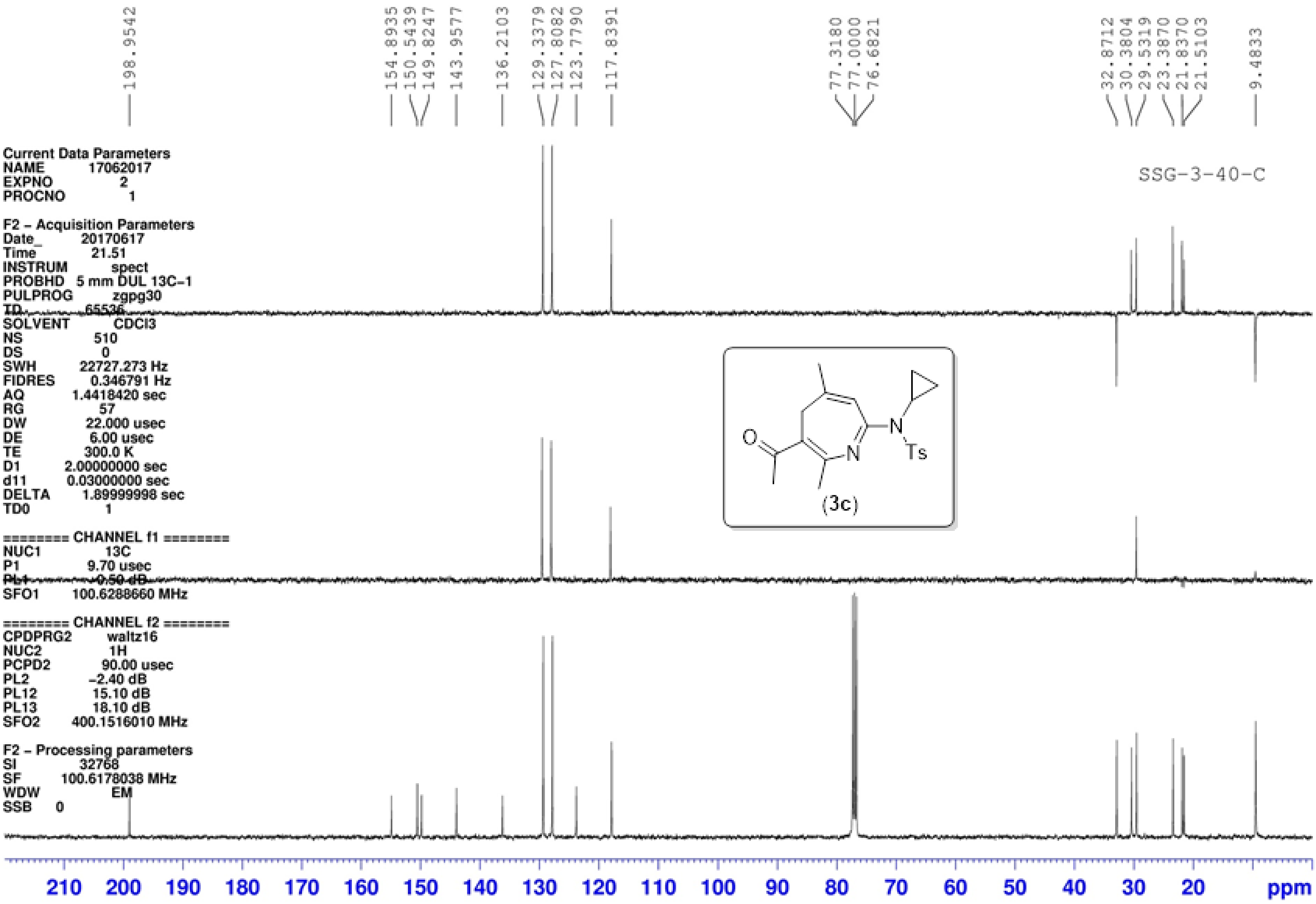
198.9542
154.8935
150.5439
149.8247
143.9577
136.2103
129.3379
127.8082
123.7790
117.8391
77.3180
77.0000
76.6821
32.8712
30.3804
29.5319
23.3870
21.8370
21.5103
9.4833
Current Data Parameters
NAME 17062017
EXPNO 2
PROCNO 1
F2 - Acquisition Parameters
Date_ 20170617
Time 21.51
INSTRUM spect
PROBHD 5 mm DUL 13C-1
PULPROG zgpg30
TD 65536
SOLVENT CDCl3
NS 510
DS 0
SWH 22727.273 Hz
FIDRES 0.346791 Hz
AQ 1.4418420 sec
RG 57
DW 22.000 usec
DE 6.00 usec
TE 300.0 K
D1 2.00000000 sec
d11 0.03000000 sec
DELTA 1.89999998 sec
TD0 1
======== CHANNEL f1 ========
NUC1 13C
P1 9.70 usec
PL1 -0.50 dB
SFO1 100.6288660 MHz
======== CHANNEL f2 ========
CPDPRG2 waltz16
NUC2 1H
PCPD2 90.00 usec
PL2 -2.40 dB
PL12 15.10 dB
PL13 18.10 dB
SFO2 400.1516010 MHz
F2 - Processing parameters
SI 32768
SF 100.6178038 MHz
WDW EM
SSB 0
SSG-3-40-C
O
N
N
Ts
(3c)
210 200 190 180 170 160 150 140 130 120 110 100 90 80 70 60 50 40 30 20 ppm

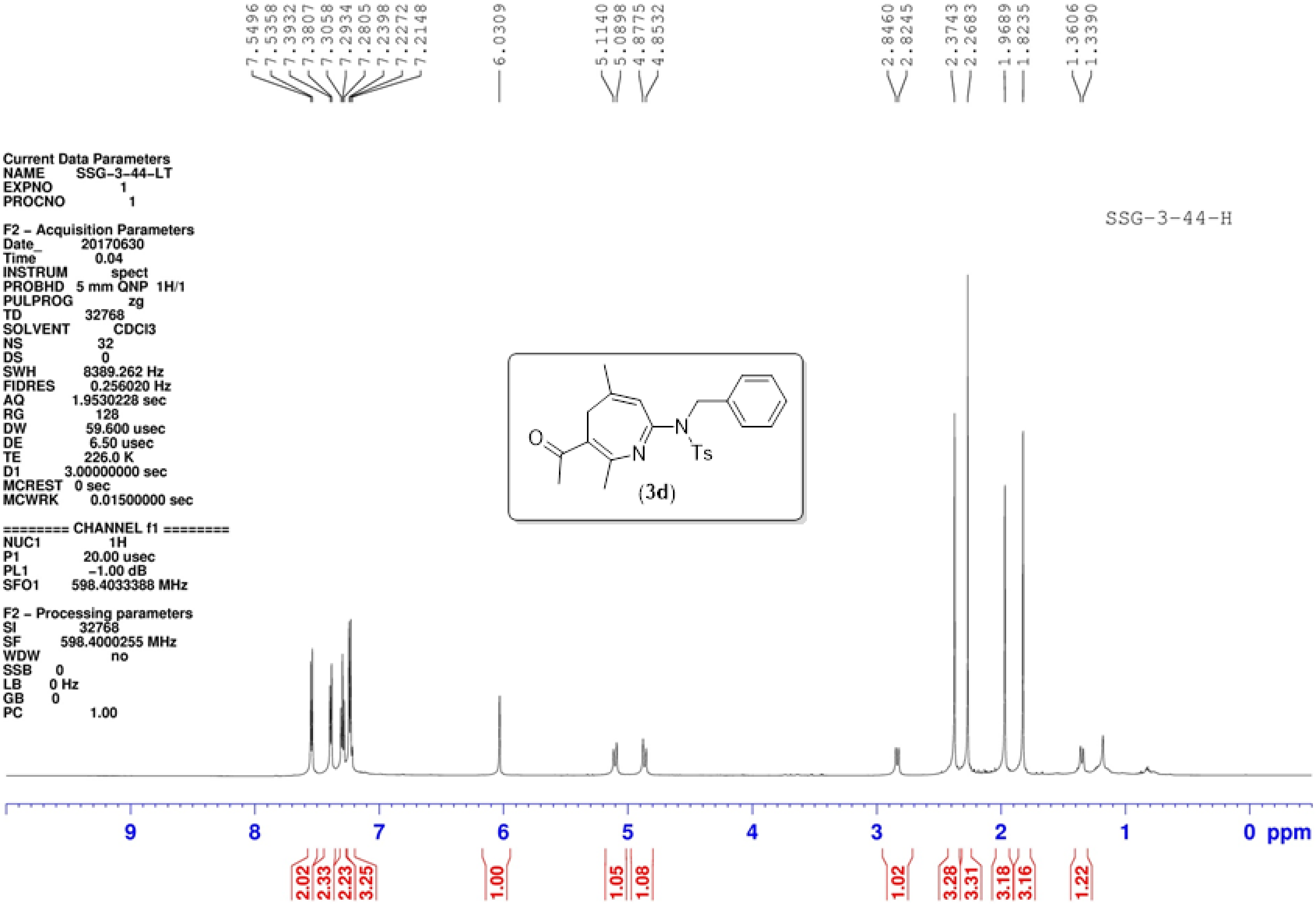
7.5496
7.5358
7.3932
7.3807
7.3058
7.2934
7.2805
7.2398
7.2272
7.2148
6.0309
5.1140
5.0898
4.8775
4.8532
2.8460
2.8245
2.3743
2.2683
1.9689
1.8235
1.3606
1.3390
Current Data Parameters
NAME SSG-3-44-LT
EXPNO 1
PROCNO 1
F2 - Acquisition Parameters
Date_ 20170630
Time 0.04
INSTRUM spect
PROBHD 5 mm QNP 1H/1
PULPROG zg
TD 32768
SOLVENT CDCl3
NS 32
DS 0
SWH 8389.262 Hz
FIDRES 0.256020 Hz
AQ 1.9530228 sec
RG 128
DW 59.600 usec
DE 6.50 usec
TE 226.0 K
D1 3.00000000 sec
MCREST 0 sec
MCWRK 0.01500000 sec
======== CHANNEL f1 ========
NUC1 1H
P1 20.00 usec
PL1 -1.00 dB
SFO1 598.4033388 MHz
F2 - Processing parameters
SI 32768
SF 598.4000255 MHz
WDW no
SSB 0
LB 0 Hz
GB 0
PC 1.00
SSG-3-44-H
O
N
N
Ts
(3d)
9
8
7
6
5
4
3
2
1
0 ppm
2.02
2.33
2.23
3.25
1.00
1.05
1.08
1.02
3.28
3.31
3.18
3.16
1.22

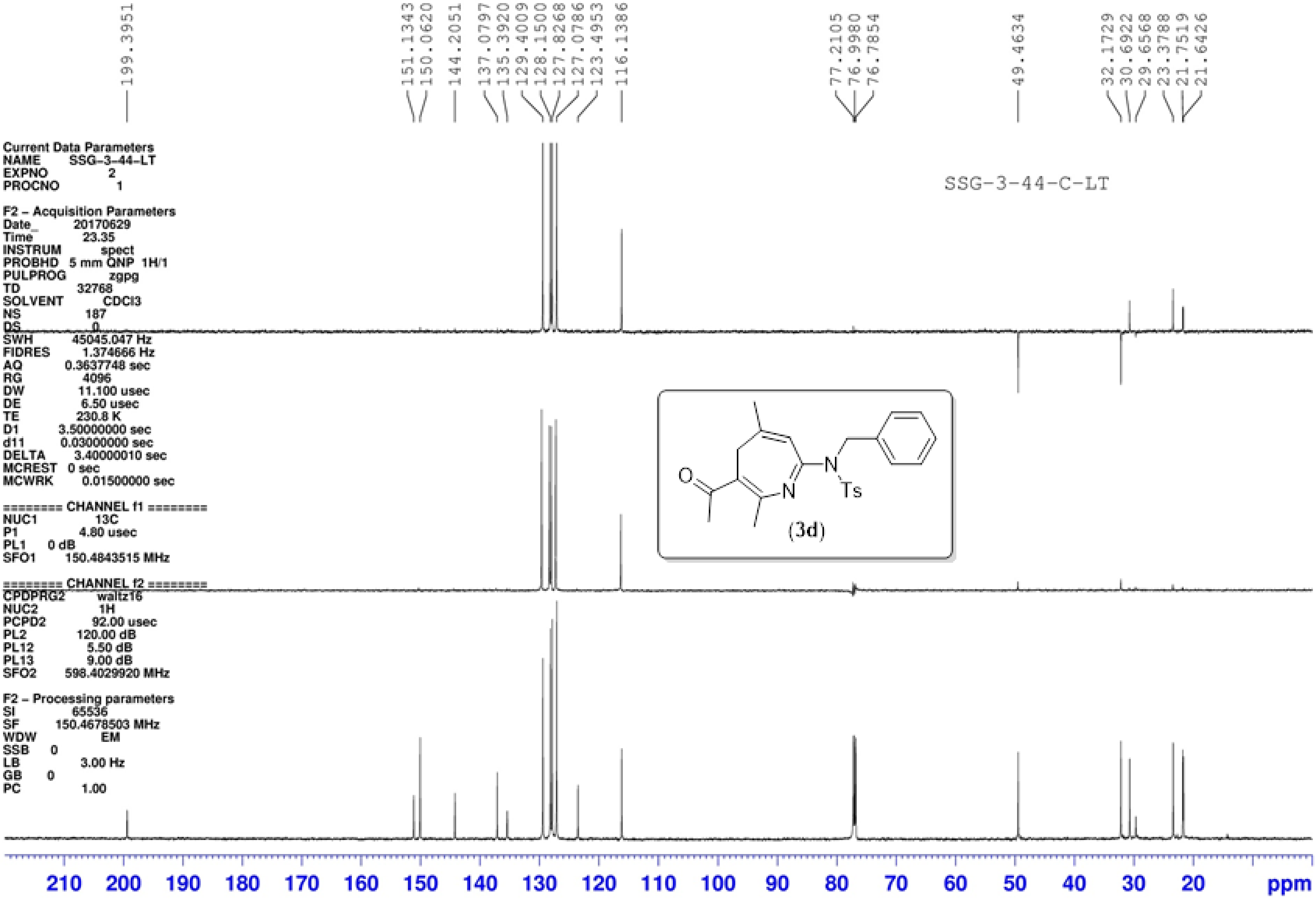

199.3951
151.1343
150.0620
144.2051
137.0797
135.3920
129.4009
128.1500
127.8268
127.0786
123.4953
116.1386
77.2105
76.9980
76.7854
49.4634
32.1729
30.6922
29.6568
23.3788
21.7519
21.6426
Current Data Parameters
NAME SSG-3-44-LT
EXPNO 2
PROCNO 1
F2 - Acquisition Parameters
Date_ 20170629
Time 23.35
INSTRUM spect
PROBHD 5 mm QNP 1H/1
PULPROG zgpg
TD 32768
SOLVENT CDCl3
NS 187
DS 0
SWH 45045.047 Hz
FIDRES 1.374666 Hz
AQ 0.3637748 sec
RG 4096
DW 11.100 usec
DE 6.50 usec
TE 230.8 K
D1 3.50000000 sec
d11 0.03000000 sec
DELTA 3.40000010 sec
MCREST 0 sec
MCWRK 0.01500000 sec
======== CHANNEL f1 ========
NUC1 13C
P1 4.80 usec
PL1 0 dB
SFO1 150.4843515 MHz
======== CHANNEL f2 ========
CPDPRG2 waltz16
NUC2 1H
PCPD2 92.00 usec
PL2 120.00 dB
PL12 5.50 dB
PL13 9.00 dB
SFO2 598.4029920 MHz
F2 - Processing parameters
SI 65536
SF 150.4678503 MHz
WDW EM
SSB 0
LB 3.00 Hz
GB 0
PC 1.00
SSG-3-44-C-LT
O
N
N
Ts
(3d)
210 200 190 180 170 160 150 140 130 120 110 100 90 80 70 60 50 40 30 20 ppm

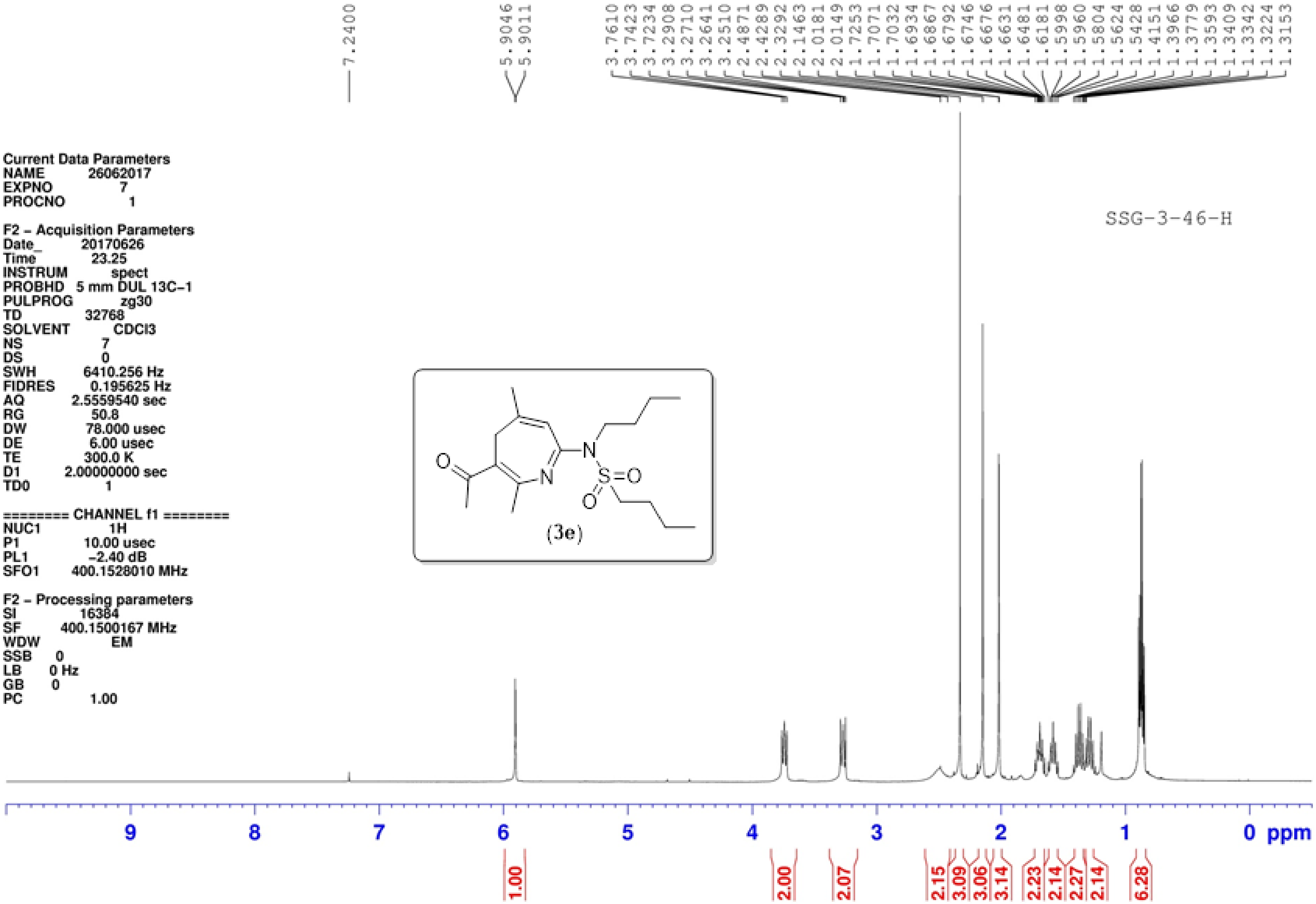
7.2400
5.9046
5.9011
3.7610
3.7423
3.7234
3.2908
3.2710
3.2641
3.2510
2.4871
2.4289
2.3292
2.1463
2.0181
2.0149
1.7253
1.7071
1.7032
1.6934
1.6867
1.6792
1.6746
1.6676
1.6631
1.6481
1.6181
1.5998
1.5960
1.5804
1.5624
1.5428
1.4151
1.3966
1.3779
1.3593
1.3409
1.3342
1.3224
1.3153
SSG-3-46-H
Current Data Parameters
NAME 26062017
EXPNO 7
PROCNO 1
F2 - Acquisition Parameters
Date_ 20170626
Time 23.25
INSTRUM spect
PROBHD 5 mm DUL 13C-1
PULPROG zg30
TD 32768
SOLVENT CDCl3
NS 7
DS 0
SWH 6410.256 Hz
FIDRES 0.195625 Hz
AQ 2.5559540 sec
RG 50.8
DW 78.000 usec
DE 6.00 usec
TE 300.0 K
D1 2.00000000 sec
TD0 1
======== CHANNEL f1 ========
NUC1 1H
P1 10.00 usec
PL1 -2.40 dB
SFO1 400.1528010 MHz
F2 - Processing parameters
SI 16384
SF 400.1500167 MHz
WDW EM
SSB 0
LB 0 Hz
GB 0
PC 1.00
(3e)
9
8
7
6
5
4
3
2
1
0 ppm
1.00
2.00
2.07
2.15
3.09
3.06
3.14
2.23
2.14
2.27
2.14
6.28

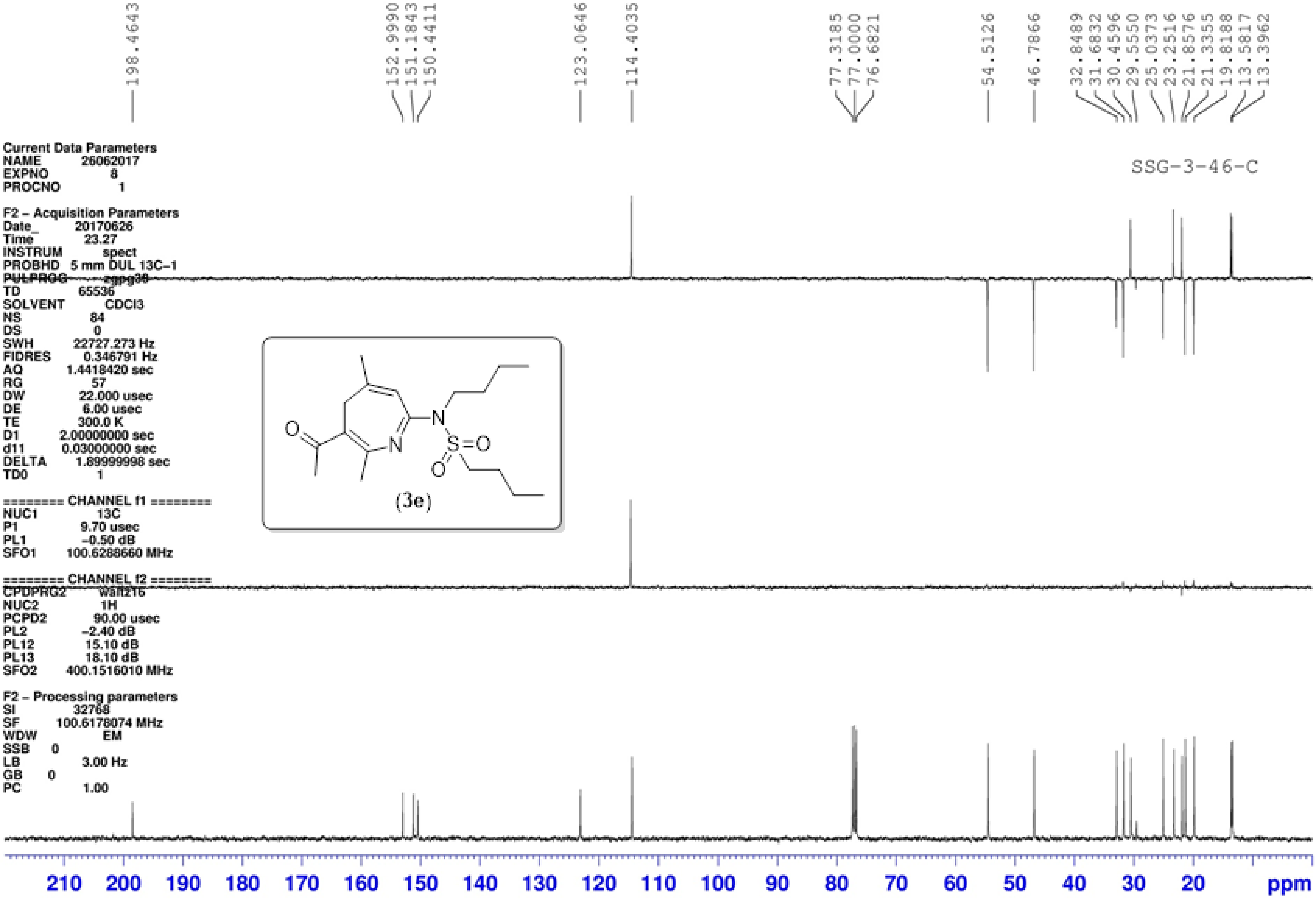
198.4643
152.9990
151.1843
150.4411
123.0646
114.4035
77.3185
77.0000
76.6821
54.5126
46.7866
32.8489
31.6832
30.4596
29.5550
25.0373
23.2516
21.8576
21.3355
19.8188
13.5817
13.3962
SSG-3-46-C
Current Data Parameters
NAME 26062017
EXPNO 8
PROCNO 1
F2 – Acquisition Parameters
Date_ 20170626
Time 23.27
INSTRUM spect
PROBHD 5 mm DUL 13C-1
PULPROG zgpg30
TD 65536
SOLVENT CDCl3
NS 84
DS 0
SWH 22727.273 Hz
FIDRES 0.346791 Hz
AQ 1.4418420 sec
RG 57
DW 22.000 usec
DE 6.00 usec
TE 300.0 K
D1 2.00000000 sec
d11 0.03000000 sec
DELTA 1.89999998 sec
TD0 1
======== CHANNEL f1 ========
NUC1 13C
P1 9.70 usec
PL1 -0.50 dB
SFO1 100.6288660 MHz
======== CHANNEL f2 ========
CPDPRG2 waltz16
NUC2 1H
PCPD2 90.00 usec
PL2 -2.40 dB
PL12 15.10 dB
PL13 18.10 dB
SFO2 400.1516010 MHz
F2 – Processing parameters
SI 32768
SF 100.6178074 MHz
WDW EM
SSB 0
LB 3.00 Hz
GB 0
PC 1.00
(3e)
210 200 190 180 170 160 150 140 130 120 110 100 90 80 70 60 50 40 30 20 ppm

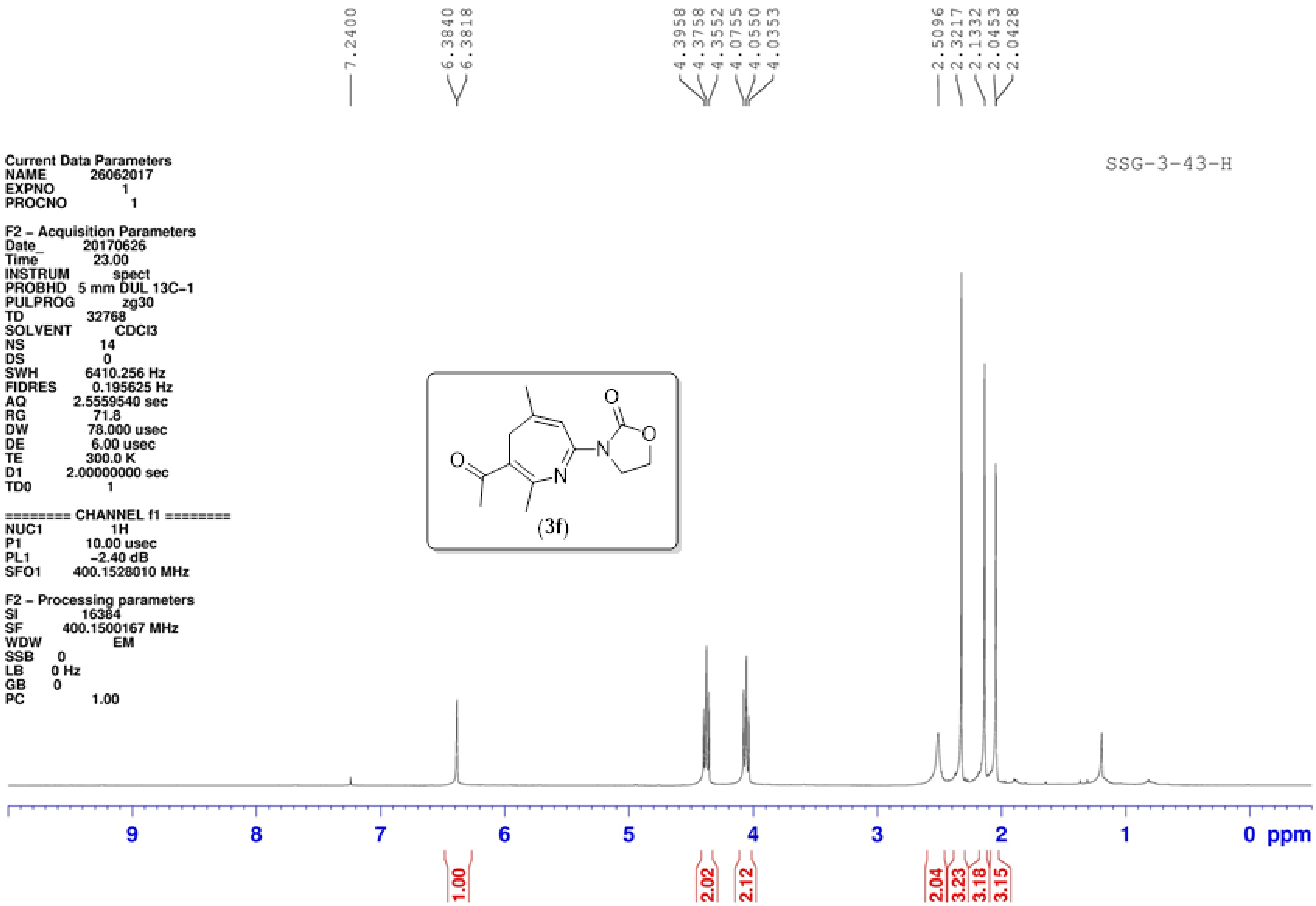

7.2400
6.3840
6.3818
4.3958
4.3758
4.3552
4.0755
4.0550
4.0353
2.5096
2.3217
2.1332
2.0453
2.0428
SSG-3-43-H
Current Data Parameters
NAME 26062017
EXPNO 1
PROCNO 1
F2 - Acquisition Parameters
Date_ 20170626
Time 23.00
INSTRUM spect
PROBHD 5 mm DUL 13C-1
PULPROG zg30
TD 32768
SOLVENT CDCl3
NS 14
DS 0
SWH 6410.256 Hz
FIDRES 0.195625 Hz
AQ 2.5559540 sec
RG 71.8
DW 78.000 usec
DE 6.00 usec
TE 300.0 K
D1 2.00000000 sec
TD0 1
======== CHANNEL f1 ========
NUC1 1H
P1 10.00 usec
PL1 -2.40 dB
SFO1 400.1528010 MHz
F2 - Processing parameters
SI 16384
SF 400.1500167 MHz
WDW EM
SSB 0
LB 0 Hz
GB 0
PC 1.00
(3f)
9 8 7 6 5 4 3 2 1 0 ppm
1.00
2.02
2.12
2.04
3.23
3.18
3.15

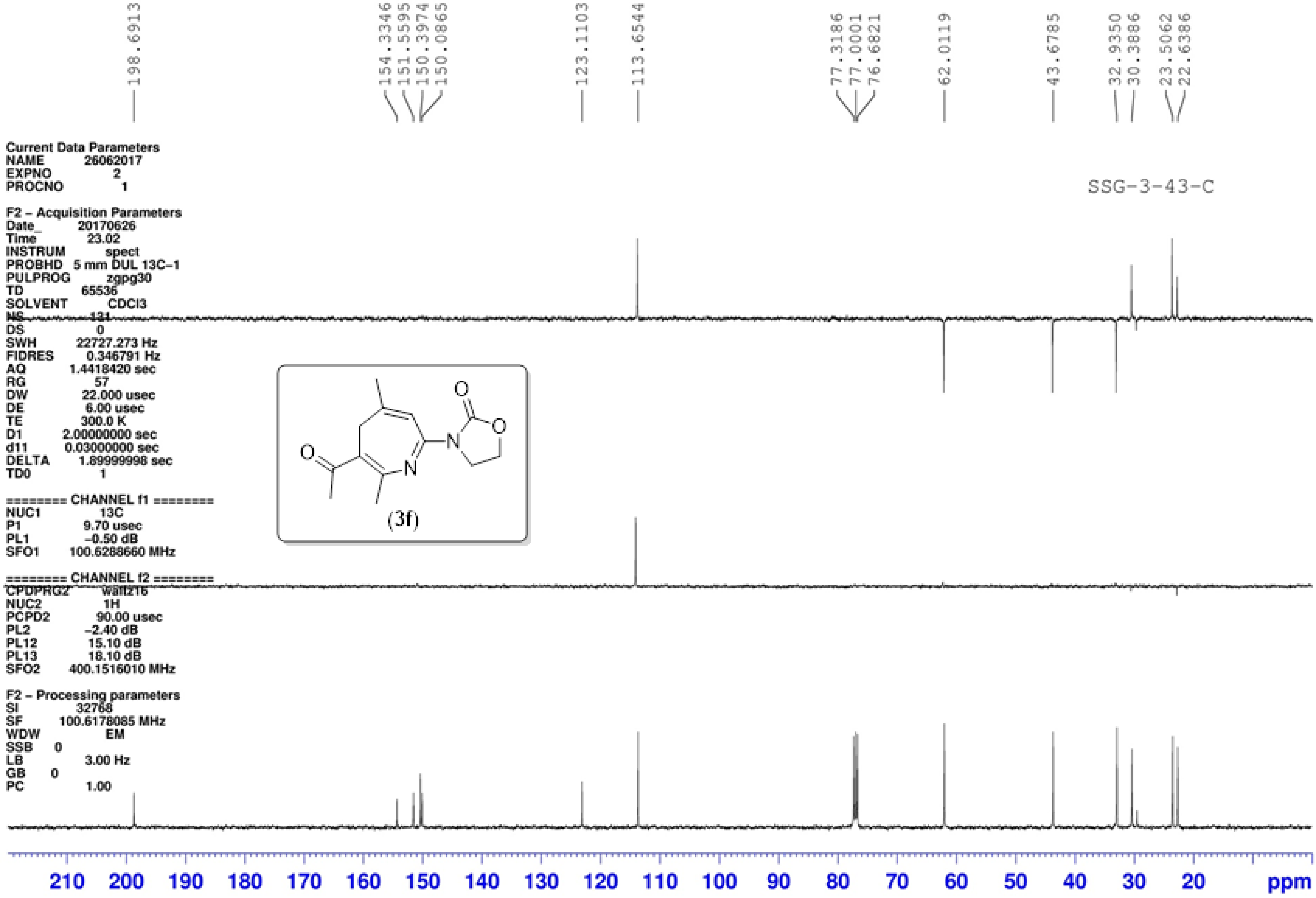
198.6913
154.3346
151.5595
150.3974
150.0865
123.1103
113.6544
77.3186
77.0001
76.6821
62.0119
43.6785
32.9350
30.3886
23.5062
22.6386
Current Data Parameters
NAME 26062017
EXPNO 2
PROCNO 1
F2 – Acquisition Parameters
Date_ 20170626
Time 23.02
INSTRUM spect
PROBHD 5 mm DUL 13C-1
PULPROG zgpg30
TD 65536
SOLVENT CDCl3
NS 131
DS 0
SWH 22727.273 Hz
FIDRES 0.346791 Hz
AQ 1.4418420 sec
RG 57
DW 22.000 usec
DE 6.00 usec
TE 300.0 K
D1 2.00000000 sec
d11 0.03000000 sec
DELTA 1.89999998 sec
TD0 1
======== CHANNEL f1 ========
NUC1 13C
P1 9.70 usec
PL1 -0.50 dB
SFO1 100.6288660 MHz
======== CHANNEL f2 ========
CPDPRG2 waltz16
NUC2 1H
PCPD2 90.00 usec
PL2 -2.40 dB
PL12 15.10 dB
PL13 18.10 dB
SFO2 400.1516010 MHz
F2 – Processing parameters
SI 32768
SF 100.6178085 MHz
WDW EM
SSB 0
LB 3.00 Hz
GB 0
PC 1.00
SSG-3-43-C
(3f)
210 200 190 180 170 160 150 140 130 120 110 100 90 80 70 60 50 40 30 20 ppm

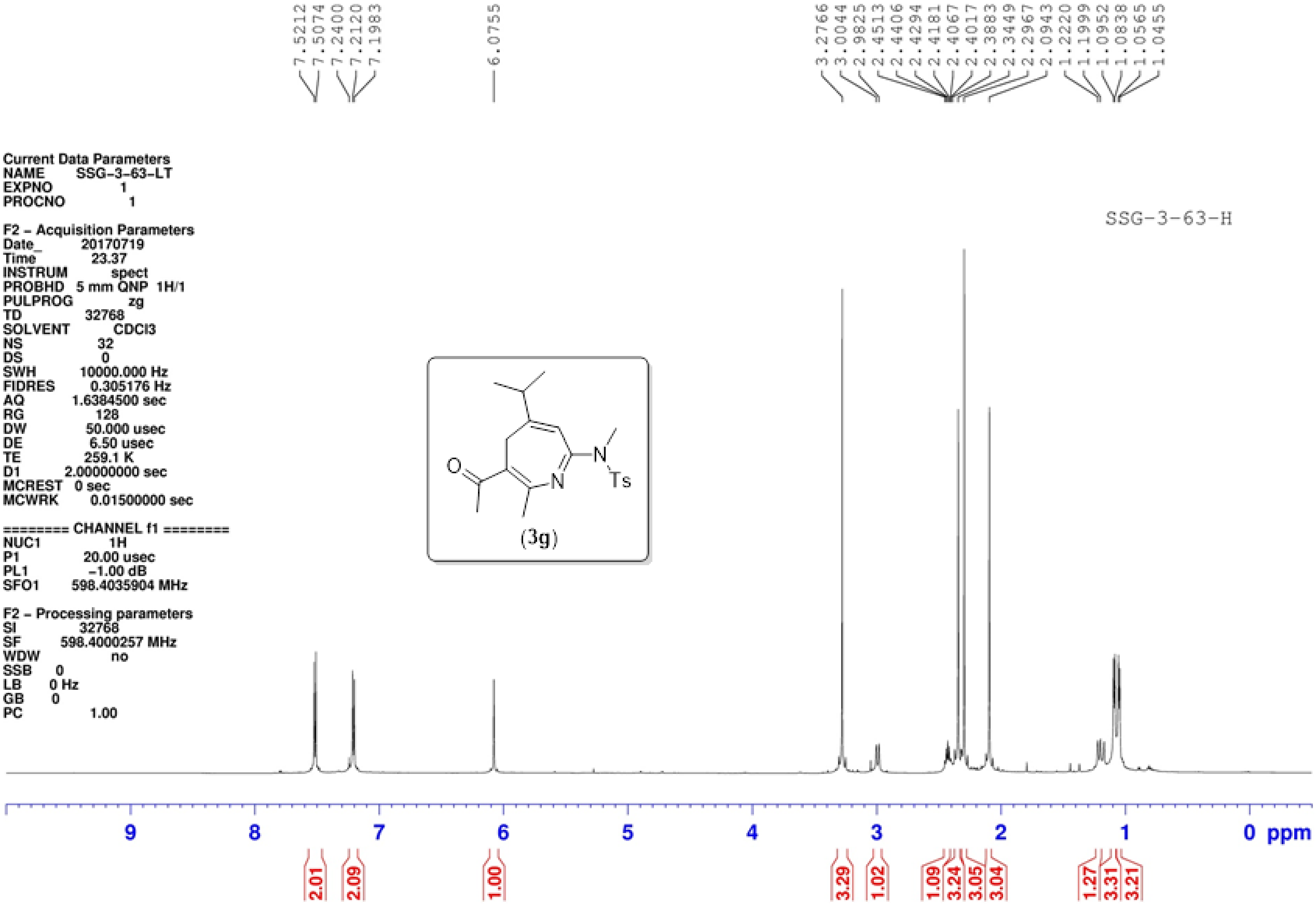
7.5212
7.5074
7.2400
7.2120
7.1983
6.0755
3.2766
3.0044
2.9825
2.4513
2.4406
2.4294
2.4181
2.4067
2.4017
2.3883
2.3449
2.2967
2.0943
1.2220
1.1999
1.0952
1.0838
1.0565
1.0455
Current Data Parameters
NAME SSG-3-63-LT
EXPNO 1
PROCNO 1
F2 - Acquisition Parameters
Date_ 20170719
Time 23.37
INSTRUM spect
PROBHD 5 mm QNP 1H/1
PULPROG zg
TD 32768
SOLVENT CDCl3
NS 32
DS 0
SWH 10000.000 Hz
FIDRES 0.305176 Hz
AQ 1.6384500 sec
RG 128
DW 50.000 usec
DE 6.50 usec
TE 259.1 K
D1 2.00000000 sec
MCREST 0 sec
MCWRK 0.01500000 sec
======== CHANNEL f1 ========
NUC1 1H
P1 20.00 usec
PL1 -1.00 dB
SFO1 598.4035904 MHz
F2 - Processing parameters
SI 32768
SF 598.4000257 MHz
WDW no
SSB 0
LB 0 Hz
GB 0
PC 1.00
SSG-3-63-H
O
N
N
Ts
(3g)
9
8
7
6
5
4
3
2
1
0 ppm
2.01
2.09
1.00
3.29
1.02
1.09
3.24
3.05
3.04
1.27
3.31
3.21

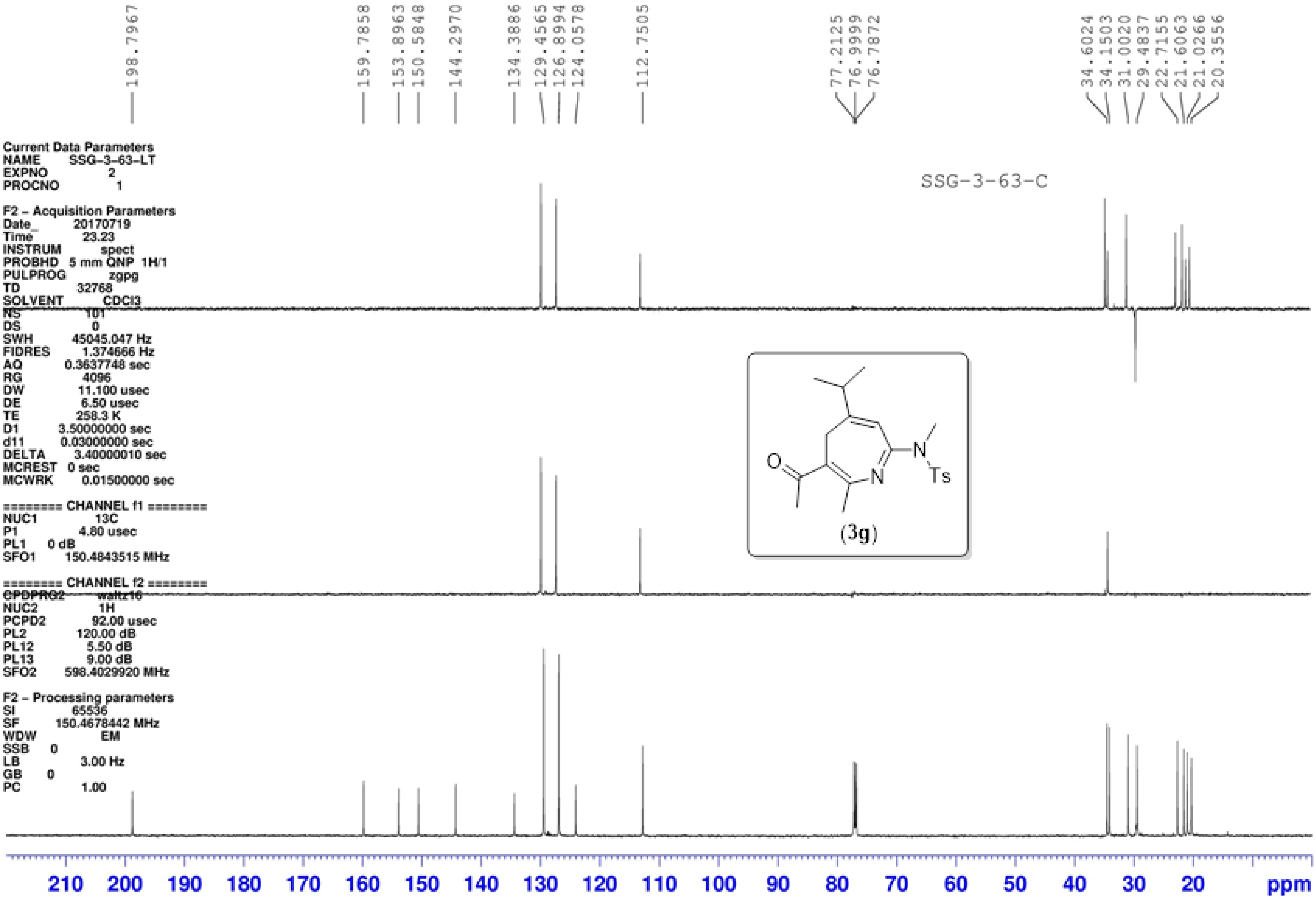

198.7967
159.7858
153.8963
150.5848
144.2970
134.3886
129.4565
126.8994
124.0578
112.7505
77.2125
76.9999
76.7872
34.6024
34.1503
31.0020
29.4837
22.7155
21.6063
21.0266
20.3556
Current Data Parameters
NAME SSG-3-63-LT
EXPNO 2
PROCNO 1
F2 - Acquisition Parameters
Date_ 20170719
Time 23.23
INSTRUM spect
PROBHD 5 mm QNP 1H/1
PULPROG zgpg
TD 32768
SOLVENT CDCl3
NS 101
DS 0
SWH 45045.047 Hz
FIDRES 1.374666 Hz
AQ 0.3637748 sec
RG 4096
DW 11.100 usec
DE 6.50 usec
TE 258.3 K
D1 3.50000000 sec
d11 0.03000000 sec
DELTA 3.40000010 sec
MCREST 0 sec
MCWRK 0.01500000 sec
======== CHANNEL f1 ========
NUC1 13C
P1 4.80 usec
PL1 0 dB
SFO1 150.4843515 MHz
======== CHANNEL f2 ========
CPDPRG2 waltz16
NUC2 1H
PCPD2 92.00 usec
PL2 120.00 dB
PL12 5.50 dB
PL13 9.00 dB
SFO2 598.4029920 MHz
F2 - Processing parameters
SI 65536
SF 150.4678442 MHz
WDW EM
SSB 0
LB 3.00 Hz
GB 0
PC 1.00
SSG-3-63-C
O
N
N
Ts
(3g)
210 200 190 180 170 160 150 140 130 120 110 100 90 80 70 60 50 40 30 20 ppm

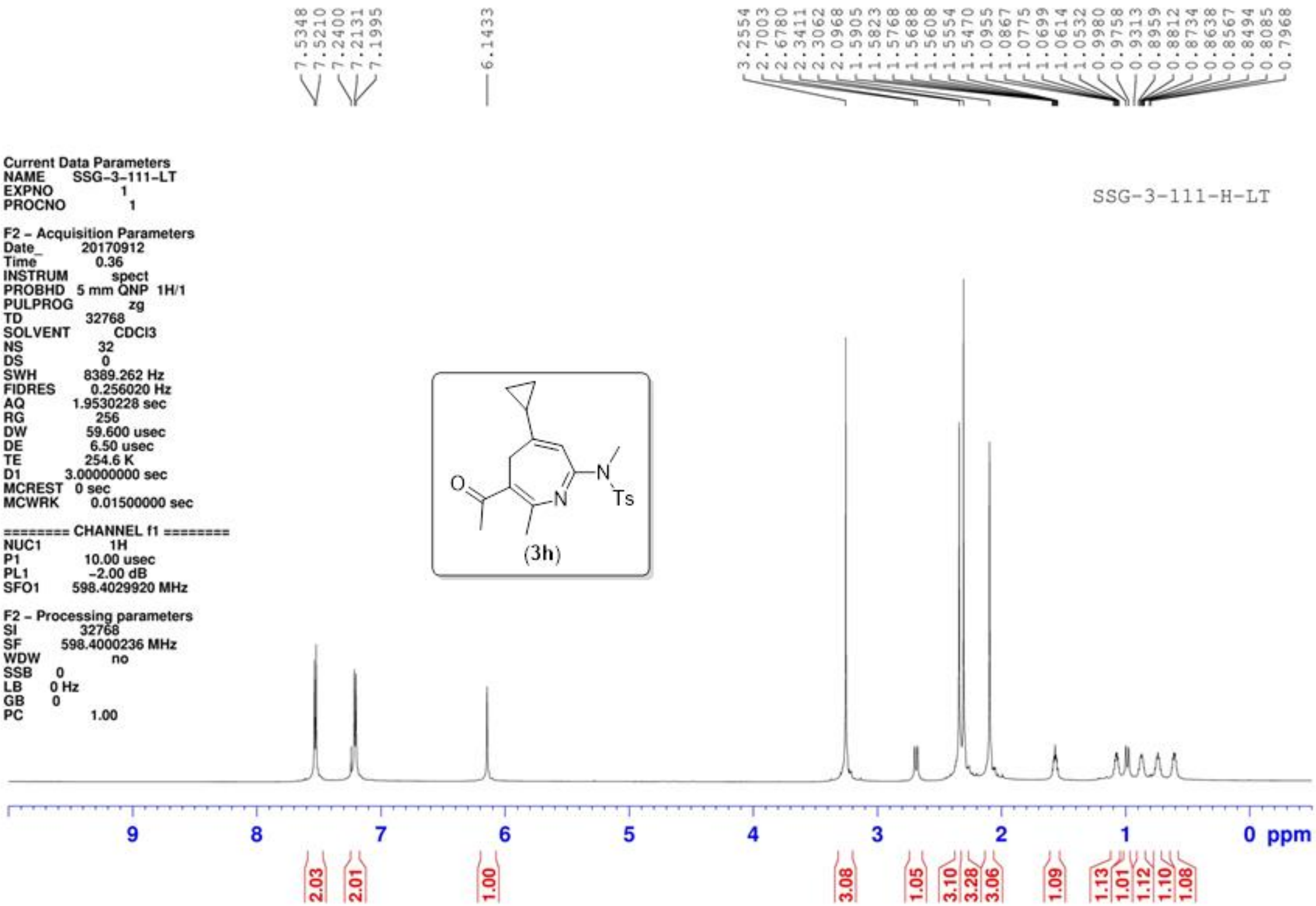
Current Data Parameters
NAME SSG-3-111-LT
EXPNO 1
PROCNO 1
F2 - Acquisition Parameters
Date_ 20170912
Time 0.36
INSTRUM spect
PROBHD 5 mm QNP 1H/1
PULPROG zg
TD 32768
SOLVENT CDCl3
NS 32
DS 0
SWH 8389.262 Hz
FIDRES 0.256020 Hz
AQ 1.9530228 sec
RG 256
DW 59.600 usec
DE 6.50 usec
TE 254.6 K
D1 3.00000000 sec
MCREST 0 sec
MCWRK 0.01500000 sec
======== CHANNEL f1 ========
NUC1 1H
P1 10.00 usec
PL1 -2.00 dB
SFO1 598.4029920 MHz
F2 - Processing parameters
SI 32768
SF 598.4000236 MHz
WDW no
SSB 0
LB 0 Hz
GB 0
PC 1.00
SSG-3-111-H-LT
7.5348
7.5210
7.2400
7.2131
7.1995
6.1433
3.2554
2.7003
2.6780
2.3411
2.3062
2.0968
1.5905
1.5823
1.5768
1.5688
1.5608
1.5554
1.5470
1.0955
1.0867
1.0775
1.0699
1.0614
1.0532
0.9980
0.9758
0.9313
0.8959
0.8812
0.8734
0.8638
0.8567
0.8494
0.8085
0.7968
(3h)
O
N
N
Ts
9
8
7
6
5
4
3
2
1
0 ppm
2.03
2.01
1.00
3.08
1.05
3.10
3.28
3.06
1.09
1.13
1.01
1.12
1.10
1.08

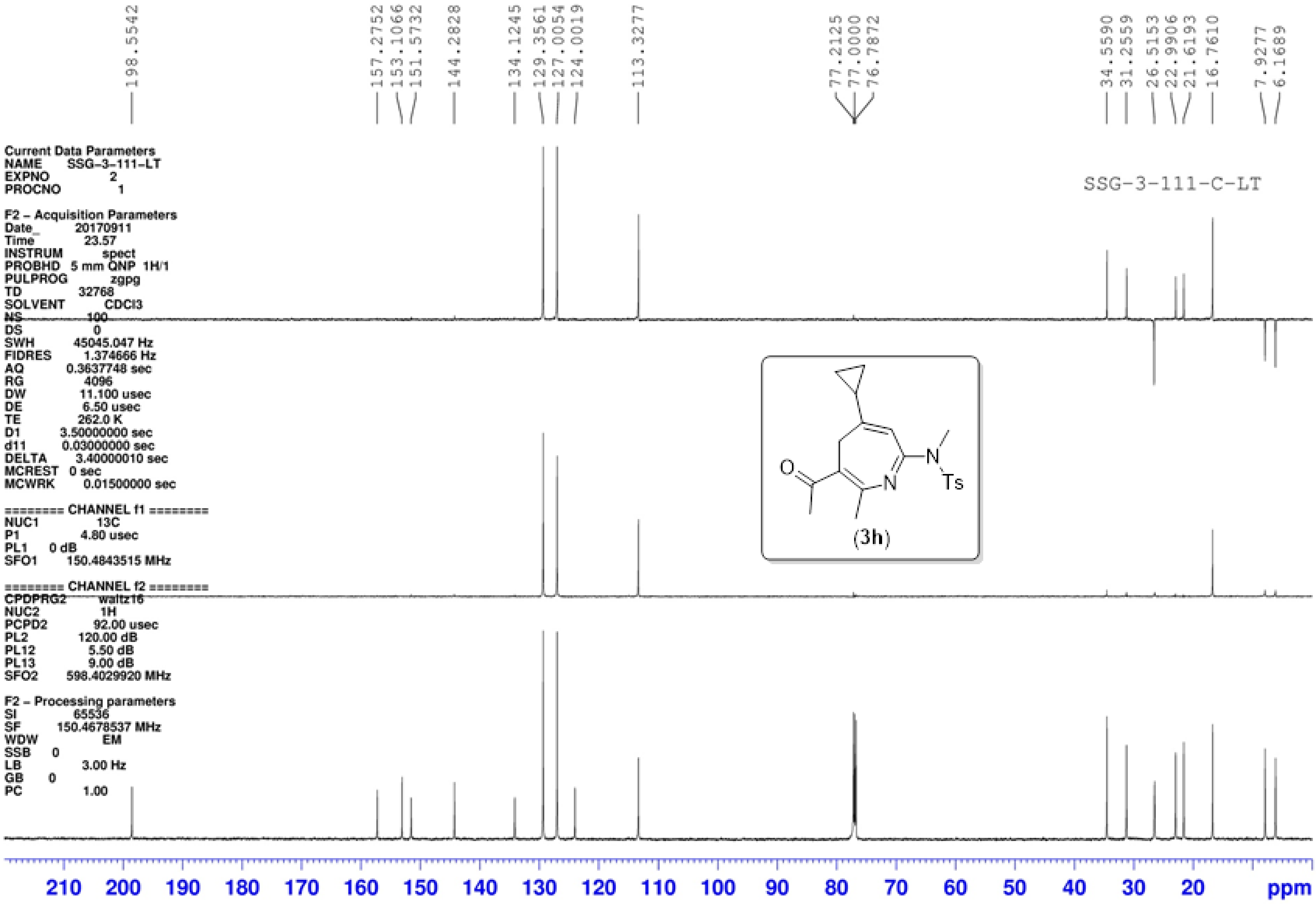

198.5542
157.2752
153.1066
151.5732
144.2828
134.1245
129.3561
127.0054
124.0019
113.3277
77.2125
77.0000
76.7872
34.5590
31.2559
26.5153
22.9906
21.6193
16.7610
7.9277
6.1689
Current Data Parameters
NAME SSG-3-111-LT
EXPNO 2
PROCNO 1
F2 - Acquisition Parameters
Date_ 20170911
Time 23.57
INSTRUM spect
PROBHD 5 mm QNP 1H/1
PULPROG zgpg
TD 32768
SOLVENT CDCl3
NS 100
DS 0
SWH 45045.047 Hz
FIDRES 1.374666 Hz
AQ 0.3637748 sec
RG 4096
DW 11.100 usec
DE 6.50 usec
TE 262.0 K
D1 3.50000000 sec
d11 0.03000000 sec
DELTA 3.40000010 sec
MCREST 0 sec
MCWRK 0.01500000 sec
======== CHANNEL f1 ========
NUC1 13C
P1 4.80 usec
PL1 0 dB
SFO1 150.4843515 MHz
======== CHANNEL f2 ========
CPDPRG2 waltz16
NUC2 1H
PCPD2 92.00 usec
PL2 120.00 dB
PL12 5.50 dB
PL13 9.00 dB
SFO2 598.4029920 MHz
F2 - Processing parameters
SI 65536
SF 150.4678537 MHz
WDW EM
SSB 0
LB 3.00 Hz
GB 0
PC 1.00
SSG-3-111-C-LT
N
Ts
O
N
(3h)
210 200 190 180 170 160 150 140 130 120 110 100 90 80 70 60 50 40 30 20 ppm

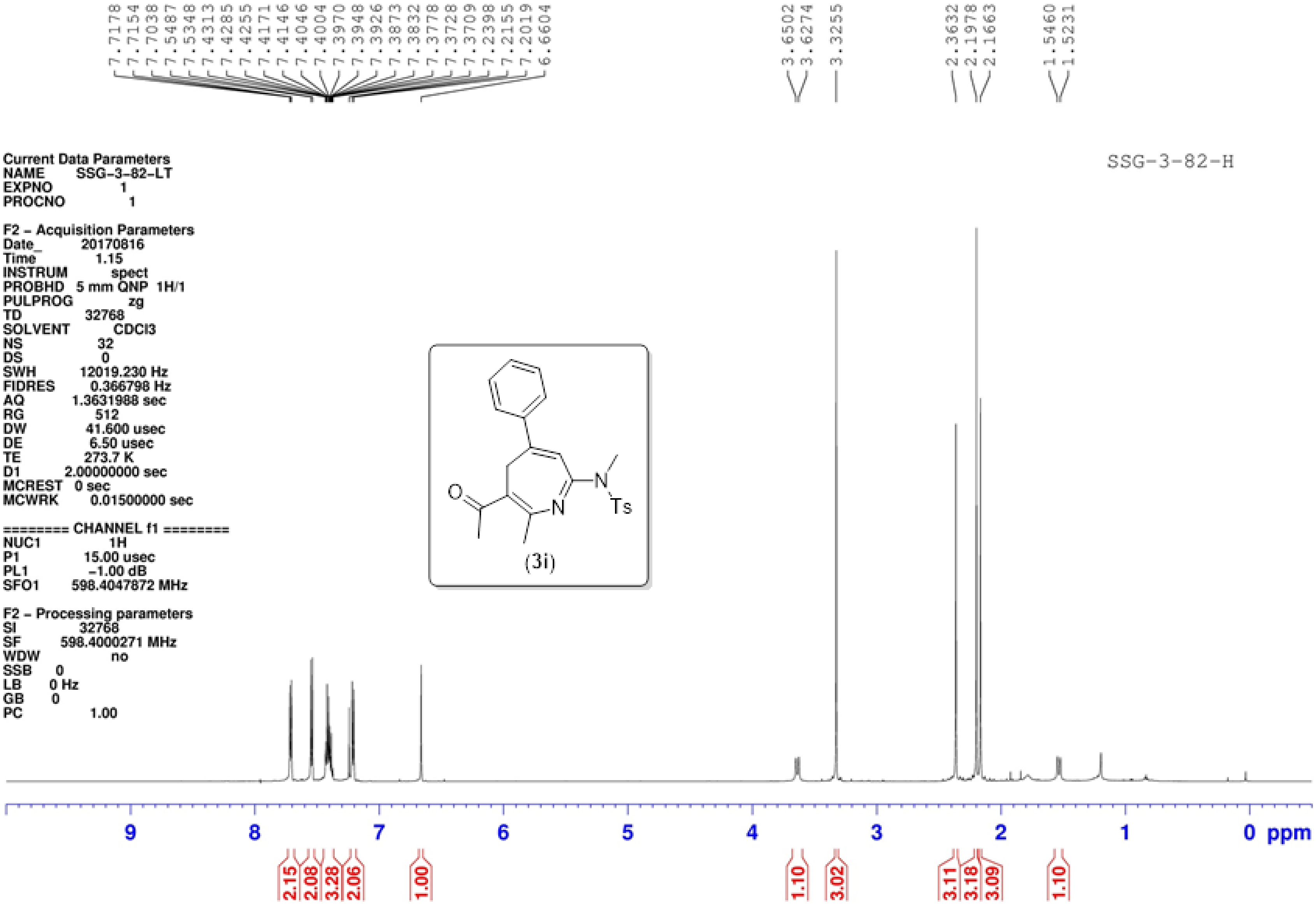
7.7178
7.7154
7.7038
7.5487
7.5348
7.4313
7.4285
7.4255
7.4171
7.4146
7.4046
7.4004
7.3970
7.3948
7.3926
7.3873
7.3832
7.3778
7.3728
7.3709
7.2398
7.2155
7.2019
6.6604
3.6502
3.6274
3.3255
2.3632
2.1978
2.1663
1.5460
1.5231
SSG-3-82-H
Current Data Parameters
NAME SSG-3-82-LT
EXPNO 1
PROCNO 1
F2 - Acquisition Parameters
Date_ 20170816
Time 1.15
INSTRUM spect
PROBHD 5 mm QNP 1H/1
PULPROG zg
TD 32768
SOLVENT CDCl3
NS 32
DS 0
SWH 12019.230 Hz
FIDRES 0.366798 Hz
AQ 1.3631988 sec
RG 512
DW 41.600 usec
DE 6.50 usec
TE 273.7 K
D1 2.00000000 sec
MCREST 0 sec
MCWRK 0.01500000 sec
======== CHANNEL f1 ========
NUC1 1H
P1 15.00 usec
PL1 -1.00 dB
SFO1 598.4047872 MHz
F2 - Processing parameters
SI 32768
SF 598.4000271 MHz
WDW no
SSB 0
LB 0 Hz
GB 0
PC 1.00
O
N
N
Ts
(3i)
9
8
7
6
5
4
3
2
1
0 ppm
2.15
2.08
3.28
2.06
1.00
1.10
3.02
3.11
3.18
3.09
1.10

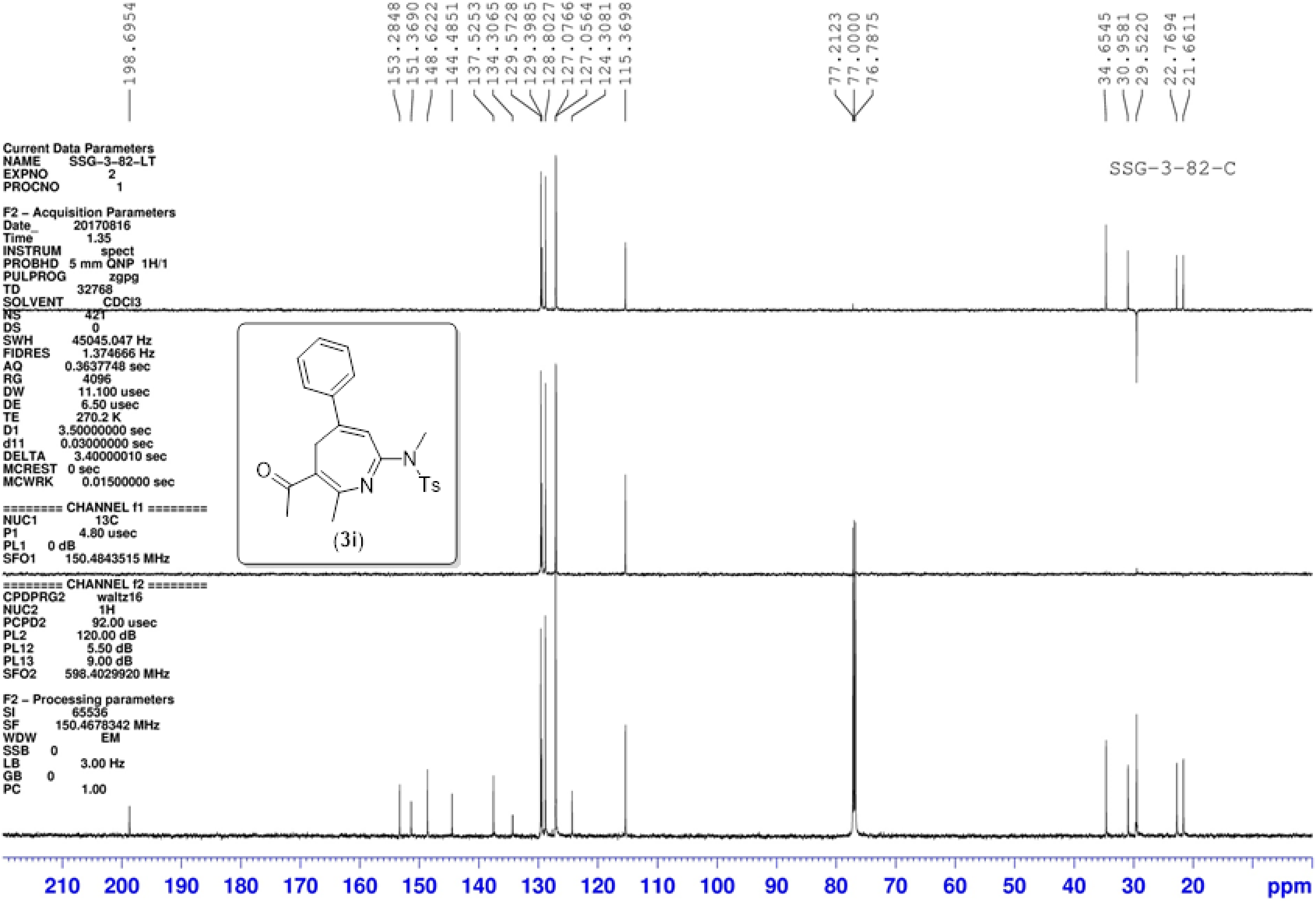

198.6954
153.2848
151.3690
148.6222
144.4851
137.5253
134.3065
129.5728
129.3985
128.8027
127.0766
127.0564
124.3081
115.3698
77.2123
77.0000
76.7875
34.6545
30.9581
29.5220
22.7694
21.6611
SSG-3-82-C
Current Data Parameters
NAME SSG-3-82-LT
EXPNO 2
PROCNO 1
F2 – Acquisition Parameters
Date_ 20170816
Time 1.35
INSTRUM spect
PROBHD 5 mm QNP 1H/1
PULPROG zgpg
TD 32768
SOLVENT CDCl3
NS 421
DS 0
SWH 45045.047 Hz
FIDRES 1.374666 Hz
AQ 0.3637748 sec
RG 4096
DW 11.100 usec
DE 6.50 usec
TE 270.2 K
D1 3.50000000 sec
d11 0.03000000 sec
DELTA 3.40000010 sec
MCREST 0 sec
MCWRK 0.01500000 sec
======== CHANNEL f1 ========
NUC1 13C
P1 4.80 usec
PL1 0 dB
SFO1 150.4843515 MHz
======== CHANNEL f2 ========
CPDPRG2 waltz16
NUC2 1H
PCPD2 92.00 usec
PL2 120.00 dB
PL12 5.50 dB
PL13 9.00 dB
SFO2 598.4029920 MHz
F2 – Processing parameters
SI 65536
SF 150.4678342 MHz
WDW EM
SSB 0
LB 3.00 Hz
GB 0
PC 1.00
O
N
N
Ts
(3i)
210 200 190 180 170 160 150 140 130 120 110 100 90 80 70 60 50 40 30 20 ppm

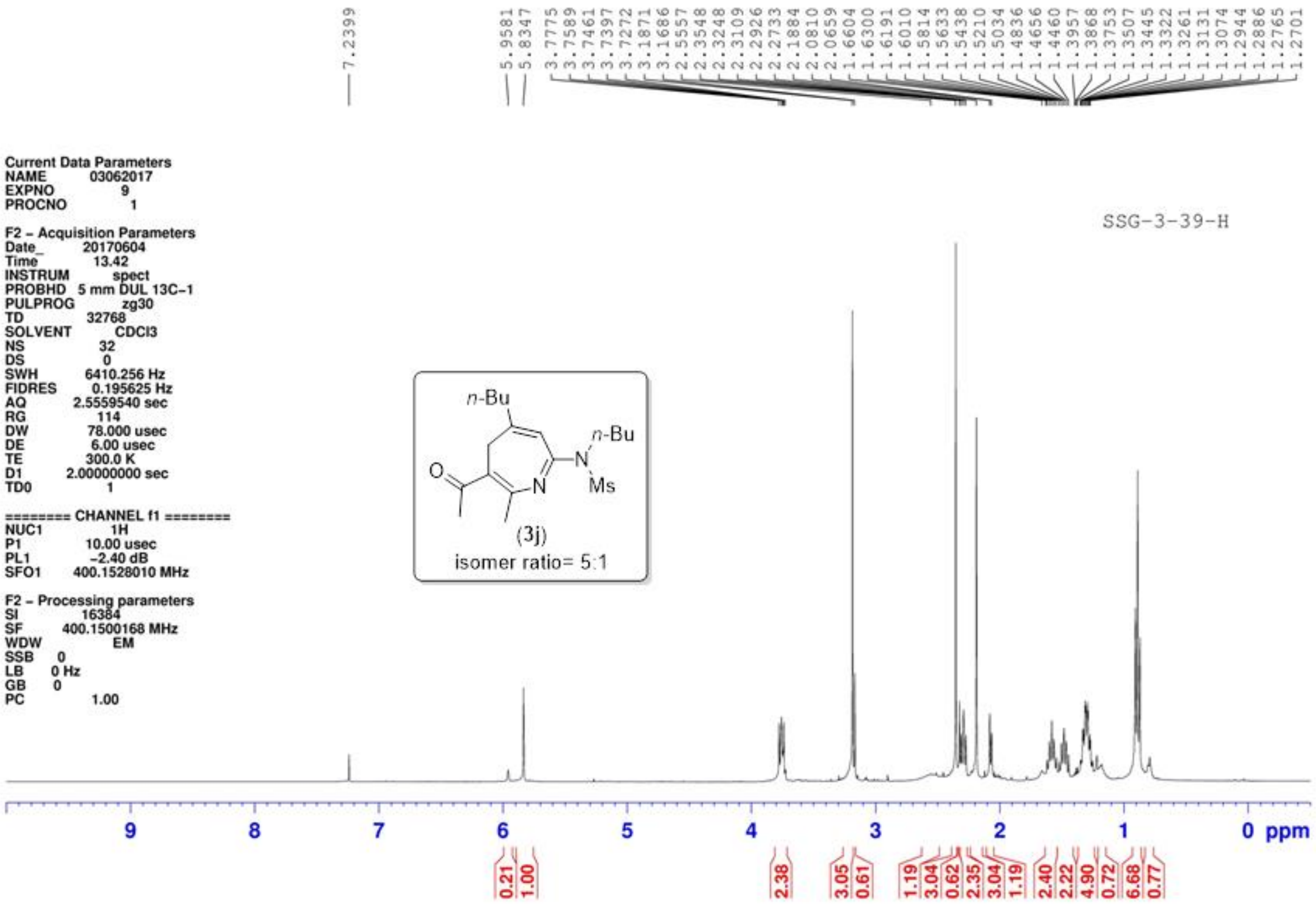
7.2399
5.9581
5.8347
3.7775
3.7589
3.7461
3.7397
3.7272
3.1871
3.1686
2.5557
2.3548
2.3248
2.3109
2.2926
2.2733
2.1884
2.0810
2.0659
1.6604
1.6300
1.6191
1.6010
1.5814
1.5633
1.5438
1.5210
1.5034
1.4836
1.4656
1.4460
1.3957
1.3868
1.3753
1.3507
1.3445
1.3322
1.3261
1.3131
1.3074
1.2944
1.2886
1.2765
1.2701
Current Data Parameters
NAME 03062017
EXPNO 9
PROCNO 1
F2 - Acquisition Parameters
Date_ 20170604
Time 13.42
INSTRUM spect
PROBHD 5 mm DUL 13C-1
PULPROG zg30
TD 32768
SOLVENT CDCl3
NS 32
DS 0
SWH 6410.256 Hz
FIDRES 0.195625 Hz
AQ 2.5559540 sec
RG 114
DW 78.000 usec
DE 6.00 usec
TE 300.0 K
D1 2.00000000 sec
TD0 1
======== CHANNEL f1 ========
NUC1 1H
P1 10.00 usec
PL1 -2.40 dB
SFO1 400.1528010 MHz
F2 - Processing parameters
SI 16384
SF 400.1500168 MHz
WDW EM
SSB 0
LB 0 Hz
GB 0
PC 1.00
SSG-3-39-H
n-Bu
n-Bu
O
N
N
Ms
(3j)
isomer ratio= 5:1
9
8
7
6
5
4
3
2
1
0 ppm
0.21
1.00
2.38
3.05
0.61
1.19
3.04
0.62
2.35
3.04
1.19
2.40
2.22
4.90
0.72
6.68
0.77

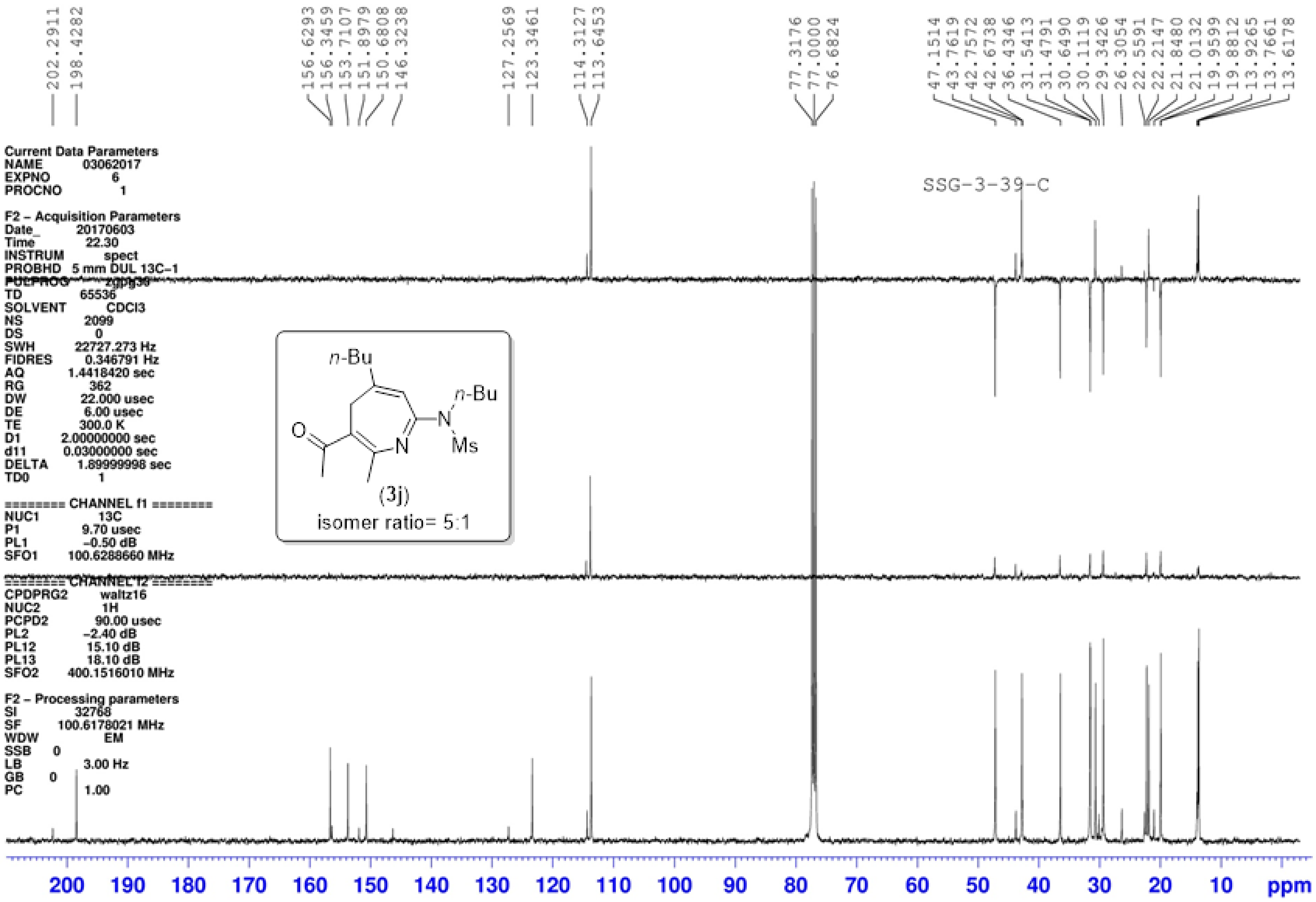

202.2911
198.4282
156.6293
156.3459
153.7107
151.8979
150.6808
146.3238
127.2569
123.3461
114.3127
113.6453
77.3176
77.0000
76.6824
47.1514
43.7619
42.7572
42.6738
36.4346
31.5413
31.4791
30.6490
30.1119
29.3426
26.3054
22.5591
22.2147
21.8480
21.0132
19.9599
19.8812
13.9265
13.7661
13.6178
SSG-3-39-C
Current Data Parameters
NAME 03062017
EXPNO 6
PROCNO 1
F2 – Acquisition Parameters
Date_ 20170603
Time 22.30
INSTRUM spect
PROBHD 5 mm DUL 13C-1
PULPROG zgpg30
TD 65536
SOLVENT CDCl3
NS 2099
DS 0
SWH 22727.273 Hz
FIDRES 0.346791 Hz
AQ 1.4418420 sec
RG 362
DW 22.000 usec
DE 6.00 usec
TE 300.0 K
D1 2.00000000 sec
d11 0.03000000 sec
DELTA 1.89999998 sec
TD0 1
======== CHANNEL f1 ========
NUC1 13C
P1 9.70 usec
PL1 -0.50 dB
SFO1 100.6288660 MHz
======== CHANNEL f2 ========
CPDPRG2 waltz16
NUC2 1H
PCPD2 90.00 usec
PL2 -2.40 dB
PL12 15.10 dB
PL13 18.10 dB
SFO2 400.1516010 MHz
F2 – Processing parameters
SI 32768
SF 100.6178021 MHz
WDW EM
SSB 0
LB 3.00 Hz
GB 0
PC 1.00
n-Bu
n-Bu
O
N
N
Ms
(3j)
isomer ratio= 5:1
200
190
180
170
160
150
140
130
120
110
100
90
80
70
60
50
40
30
20
10
ppm

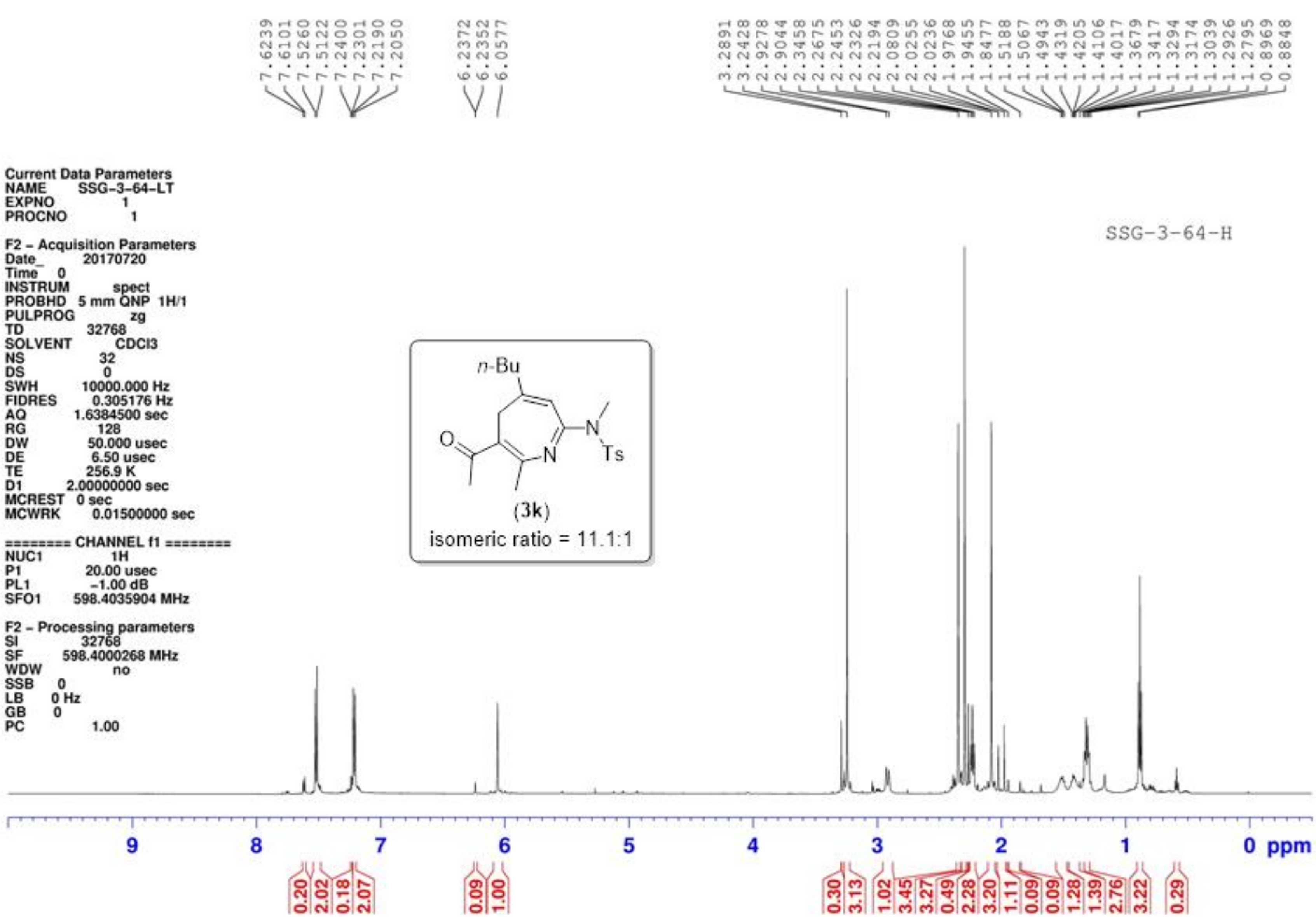
7.6239
7.6101
7.5260
7.5122
7.2400
7.2301
7.2190
7.2050
6.2372
6.2352
6.0577
3.2891
3.2428
2.9278
2.9044
2.3458
2.2675
2.2453
2.2326
2.2194
2.0809
2.0255
2.0236
1.9768
1.9455
1.8477
1.5188
1.5067
1.4943
1.4319
1.4205
1.4106
1.4017
1.3679
1.3417
1.3294
1.3174
1.3039
1.2926
1.2795
0.8969
0.8848
Current Data Parameters
NAME SSG-3-64-LT
EXPNO 1
PROCNO 1
F2 - Acquisition Parameters
Date_ 20170720
Time 0
INSTRUM spect
PROBHD 5 mm QNP 1H/1
PULPROG zg
TD 32768
SOLVENT CDCl3
NS 32
DS 0
SWH 10000.000 Hz
FIDRES 0.305176 Hz
AQ 1.6384500 sec
RG 128
DW 50.000 usec
DE 6.50 usec
TE 256.9 K
D1 2.00000000 sec
MCREST 0 sec
MCWRK 0.01500000 sec
======== CHANNEL f1 ========
NUC1 1H
P1 20.00 usec
PL1 -1.00 dB
SFO1 598.4035904 MHz
F2 - Processing parameters
SI 32768
SF 598.4000268 MHz
WDW no
SSB 0
LB 0 Hz
GB 0
PC 1.00
SSG-3-64-H
n-Bu
O
N
N
Ts
(3k)
isomeric ratio = 11.1:1
9
8
7
6
5
4
3
2
1
0 ppm
0.20
2.02
0.18
2.07
0.09
1.00
0.30
3.13
1.02
3.45
3.27
0.49
2.28
3.20
1.11
0.09
0.09
1.28
1.39
2.76
3.22
0.29

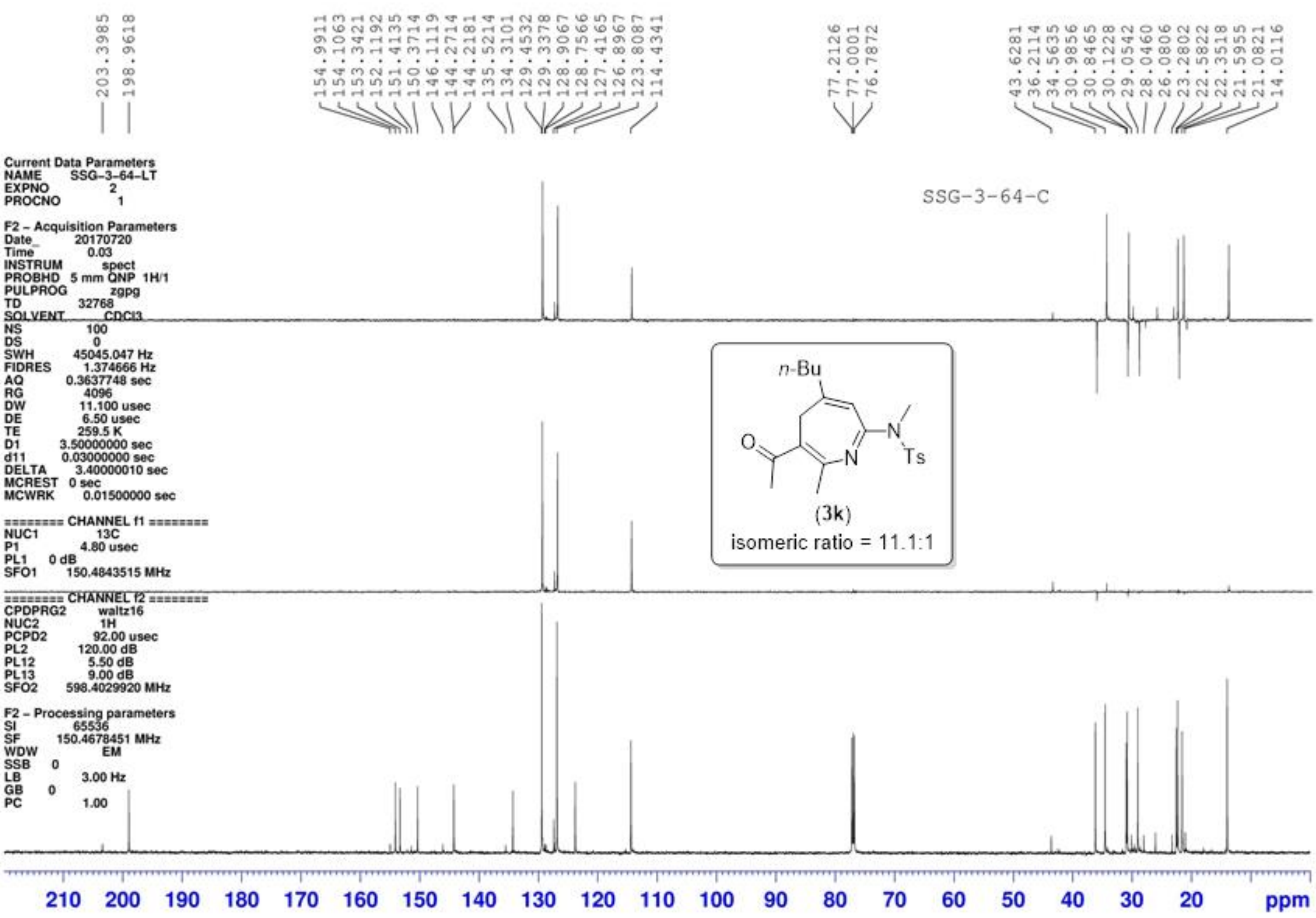

203.3985
198.9618
154.9911
154.1063
153.3421
152.1192
151.4135
150.3714
146.1119
144.2714
144.2181
135.5214
134.3101
129.4532
129.3378
128.9067
128.7566
127.4165
126.8967
123.8087
114.4341
77.2126
77.0001
76.7872
43.6281
36.2114
34.5635
30.9856
30.8465
30.1228
29.0542
28.0460
26.0806
23.2802
22.5822
22.3518
21.5955
21.0821
14.0116
Current Data Parameters
NAME SSG-3-64-LT
EXPNO 2
PROCNO 1
F2 - Acquisition Parameters
Date_ 20170720
Time 0.03
INSTRUM spect
PROBHD 5 mm QNP 1H/1
PULPROG zgpg
TD 32768
SOLVENT CDCl3
NS 100
DS 0
SWH 45045.047 Hz
FIDRES 1.374666 Hz
AQ 0.3637748 sec
RG 4096
DW 11.100 usec
DE 6.50 usec
TE 259.5 K
D1 3.50000000 sec
d11 0.03000000 sec
DELTA 3.40000010 sec
MCREST 0 sec
MCWRK 0.01500000 sec
======== CHANNEL f1 ========
NUC1 13C
P1 4.80 usec
PL1 0 dB
SFO1 150.4843515 MHz
======== CHANNEL f2 ========
CPDPRG2 waltz16
NUC2 1H
PCPD2 92.00 usec
PL2 120.00 dB
PL12 5.50 dB
PL13 9.00 dB
SFO2 598.4029920 MHz
F2 - Processing parameters
SI 65536
SF 150.4678451 MHz
WDW EM
SSB 0
LB 3.00 Hz
GB 0
PC 1.00
SSG-3-64-C
n-Bu
O
N
N
Ts
(3k)
isomeric ratio = 11.1:1
210 200 190 180 170 160 150 140 130 120 110 100 90 80 70 60 50 40 30 20 ppm

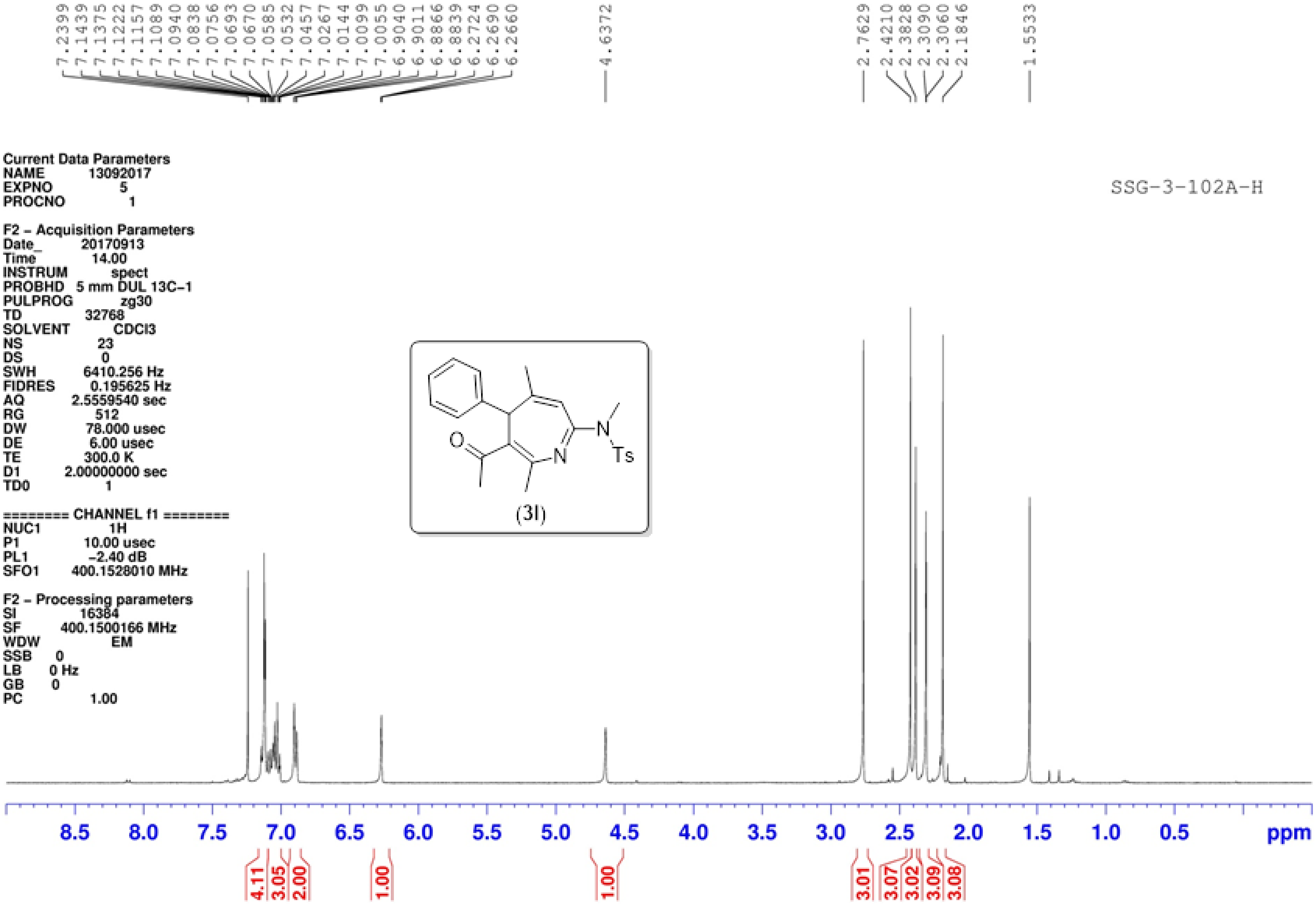
7.2399
7.1439
7.1375
7.1222
7.1157
7.1089
7.0940
7.0838
7.0756
7.0693
7.0670
7.0585
7.0532
7.0457
7.0267
7.0144
7.0099
7.0055
6.9040
6.9011
6.8866
6.8839
6.2724
6.2690
6.2660
4.6372
2.7629
2.4210
2.3828
2.3090
2.3060
2.1846
1.5533
Current Data Parameters
NAME 13092017
EXPNO 5
PROCNO 1
F2 - Acquisition Parameters
Date_ 20170913
Time 14.00
INSTRUM spect
PROBHD 5 mm DUL 13C-1
PULPROG zg30
TD 32768
SOLVENT CDCl3
NS 23
DS 0
SWH 6410.256 Hz
FIDRES 0.195625 Hz
AQ 2.5559540 sec
RG 512
DW 78.000 usec
DE 6.00 usec
TE 300.0 K
D1 2.00000000 sec
TD0 1
======== CHANNEL f1 ========
NUC1 1H
P1 10.00 usec
PL1 -2.40 dB
SFO1 400.1528010 MHz
F2 - Processing parameters
SI 16384
SF 400.1500166 MHz
WDW EM
SSB 0
LB 0 Hz
GB 0
PC 1.00
SSG-3-102A-H
O
N
N
Ts
(3l)
8.5
8.0
7.5
7.0
6.5
6.0
5.5
5.0
4.5
4.0
3.5
3.0
2.5
2.0
1.5
1.0
0.5
ppm
4.11
3.05
2.00
1.00
1.00
3.01
3.07
3.02
3.09
3.08

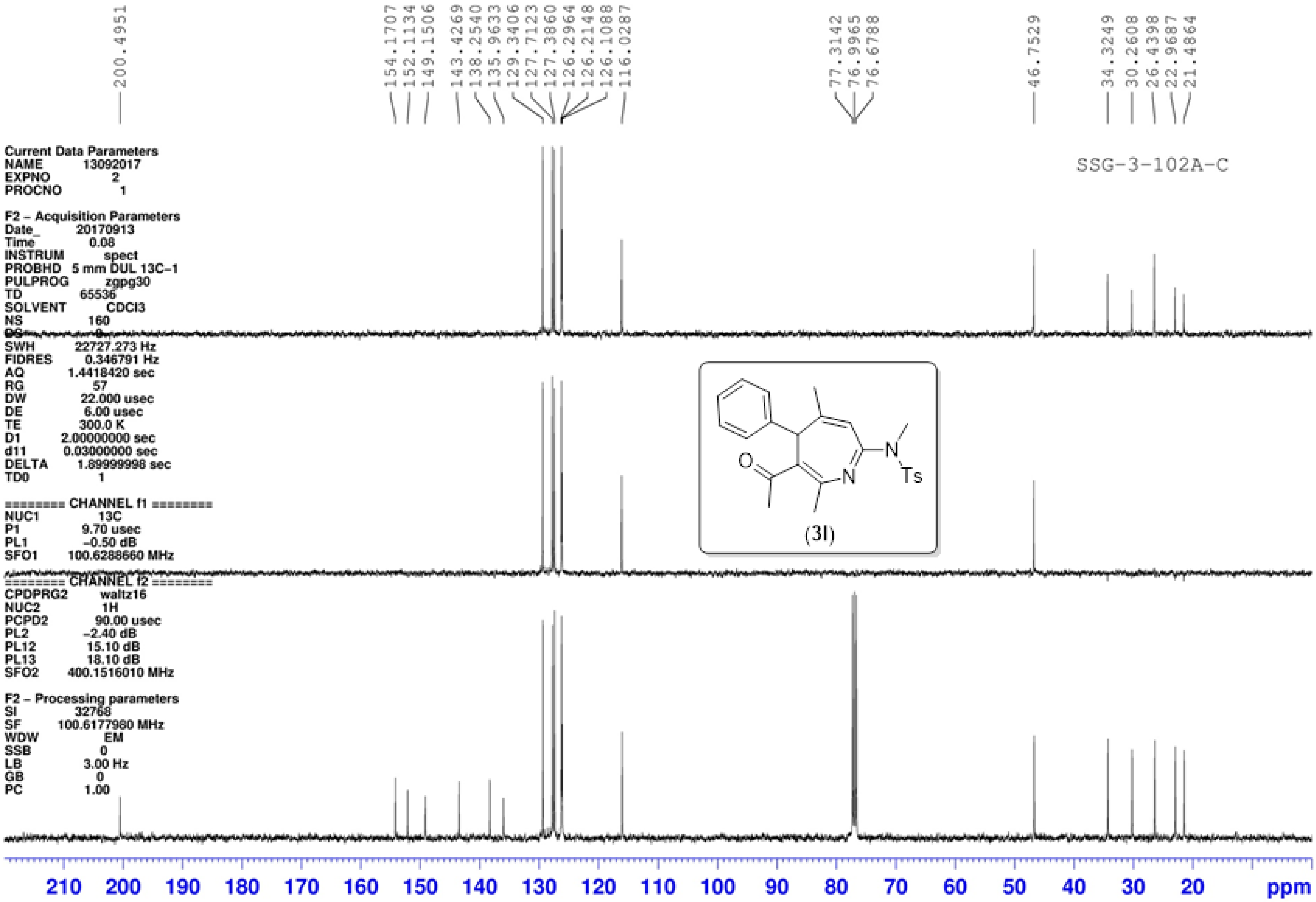

200.4951
154.1707
152.1134
149.1506
143.4269
138.2540
135.9633
129.3406
127.7123
127.3860
126.2964
126.2148
126.1088
116.0287
77.3142
76.9965
76.6788
46.7529
34.3249
30.2608
26.4398
22.9687
21.4864
SSG-3-102A-C
Current Data Parameters
NAME 13092017
EXPNO 2
PROCNO 1
F2 – Acquisition Parameters
Date_ 20170913
Time 0.08
INSTRUM spect
PROBHD 5 mm DUL 13C-1
PULPROG zgpg30
TD 65536
SOLVENT CDCl3
NS 160
DS 0
SWH 22727.273 Hz
FIDRES 0.346791 Hz
AQ 1.4418420 sec
RG 57
DW 22.000 usec
DE 6.00 usec
TE 300.0 K
D1 2.00000000 sec
d11 0.03000000 sec
DELTA 1.89999998 sec
TD0 1
======== CHANNEL f1 ========
NUC1 13C
P1 9.70 usec
PL1 -0.50 dB
SFO1 100.6288660 MHz
======== CHANNEL f2 ========
CPDPRG2 waltz16
NUC2 1H
PCPD2 90.00 usec
PL2 -2.40 dB
PL12 15.10 dB
PL13 18.10 dB
SFO2 400.1516010 MHz
F2 – Processing parameters
SI 32768
SF 100.6177980 MHz
WDW EM
SSB 0
LB 3.00 Hz
GB 0
PC 1.00
O
N
N
Ts
(3l)
210 200 190 180 170 160 150 140 130 120 110 100 90 80 70 60 50 40 30 20 ppm

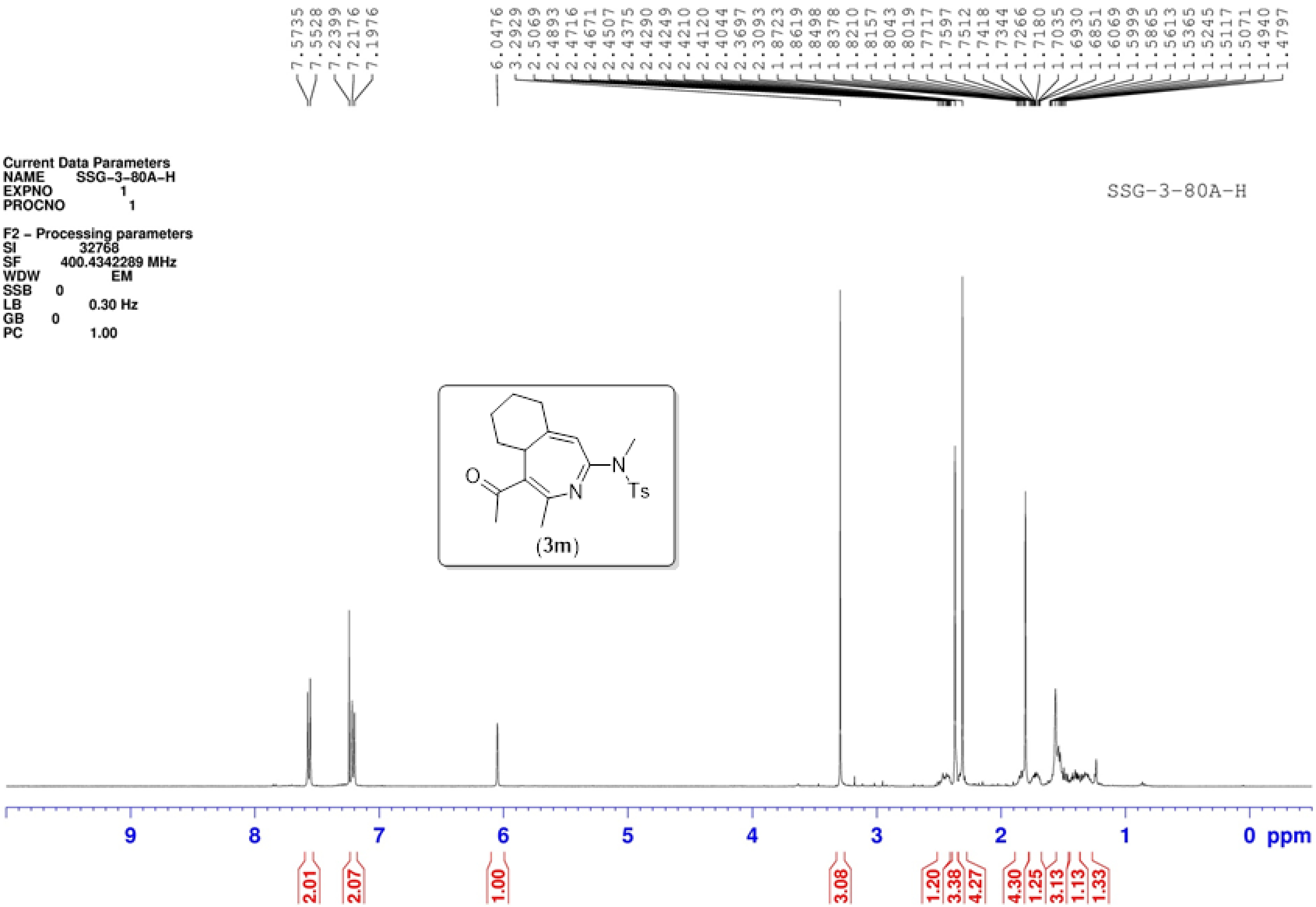
7.5735
7.5528
7.2399
7.2176
7.1976
6.0476
3.2929
2.5069
2.4893
2.4716
2.4671
2.4507
2.4375
2.4290
2.4249
2.4210
2.4120
2.4044
2.3697
2.3093
1.8723
1.8619
1.8498
1.8378
1.8210
1.8157
1.8043
1.8019
1.7717
1.7597
1.7512
1.7418
1.7344
1.7266
1.7180
1.7035
1.6930
1.6851
1.6069
1.5999
1.5865
1.5613
1.5365
1.5245
1.5117
1.5071
1.4940
1.4797
Current Data Parameters
NAME SSG-3-80A-H
EXPNO 1
PROCNO 1
F2 - Processing parameters
SI 32768
SF 400.4342289 MHz
WDW EM
SSB 0
LB 0.30 Hz
GB 0
PC 1.00
SSG-3-80A-H
O
N
Ts
N
(3m)
9
8
7
6
5
4
3
2
1
0 ppm
2.01
2.07
1.00
3.08
1.20
3.38
4.27
4.30
1.25
3.13
1.13
1.33

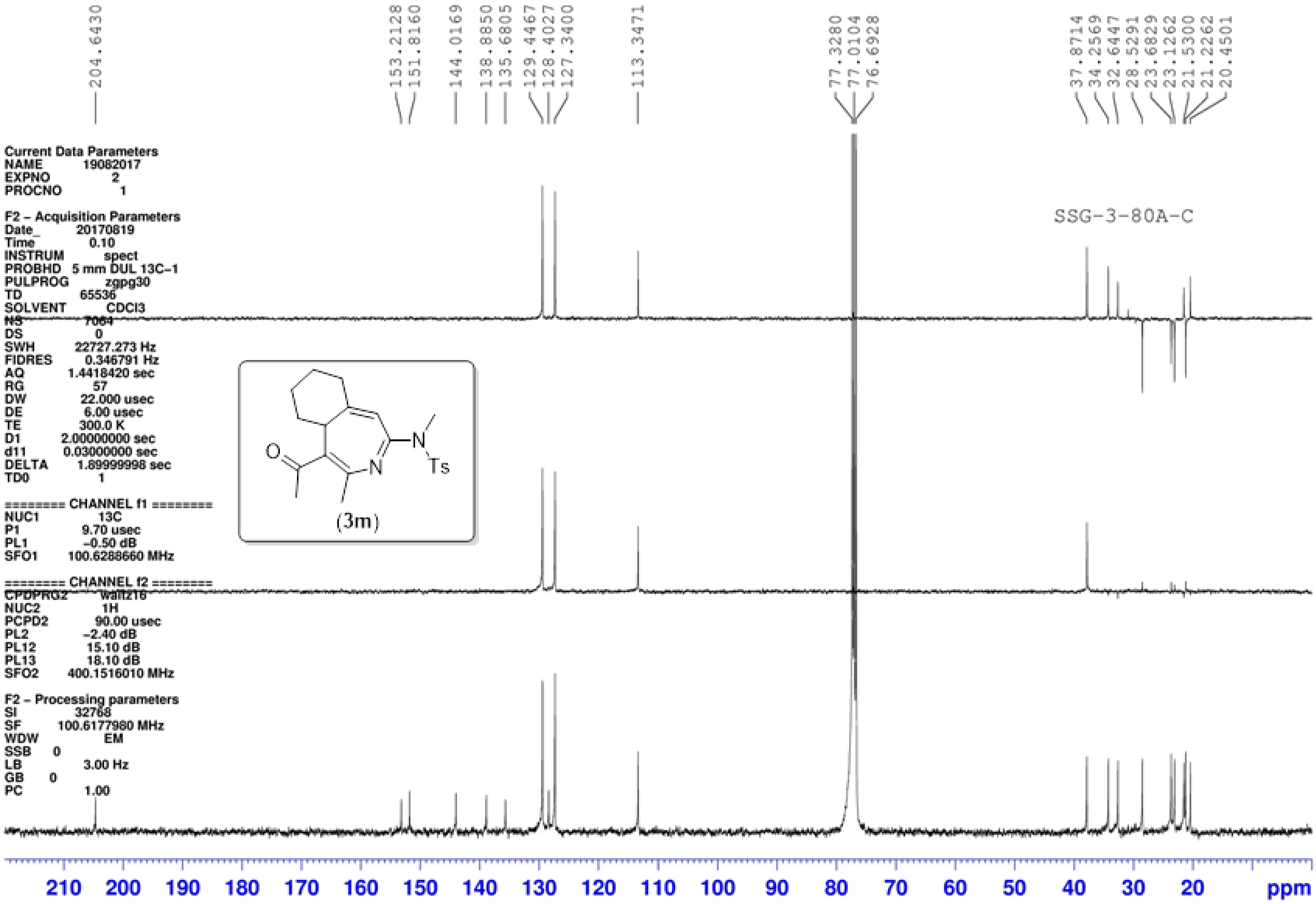
204.6430
153.2128
151.8160
144.0169
138.8850
135.6805
129.4467
128.4027
127.3400
113.3471
77.3280
77.0104
76.6928
37.8714
34.2569
32.6447
28.5291
23.6829
23.1262
21.5300
21.2262
20.4501
SSG-3-80A-C
Current Data Parameters
NAME 19082017
EXPNO 2
PROCNO 1
F2 – Acquisition Parameters
Date_ 20170819
Time 0.10
INSTRUM spect
PROBHD 5 mm DUL 13C-1
PULPROG zgpg30
TD 65536
SOLVENT CDCl3
NS 7064
DS 0
SWH 22727.273 Hz
FIDRES 0.346791 Hz
AQ 1.4418420 sec
RG 57
DW 22.000 usec
DE 6.00 usec
TE 300.0 K
D1 2.00000000 sec
d11 0.03000000 sec
DELTA 1.89999998 sec
TD0 1
======== CHANNEL f1 ========
NUC1 13C
P1 9.70 usec
PL1 -0.50 dB
SFO1 100.6288660 MHz
======== CHANNEL f2 ========
CPDPRG2 waltz16
NUC2 1H
PCPD2 90.00 usec
PL2 -2.40 dB
PL12 15.10 dB
PL13 18.10 dB
SFO2 400.1516010 MHz
F2 – Processing parameters
SI 32768
SF 100.6177980 MHz
WDW EM
SSB 0
LB 3.00 Hz
GB 0
PC 1.00
O
N
N
Ts
(3m)
210 200 190 180 170 160 150 140 130 120 110 100 90 80 70 60 50 40 30 20 ppm

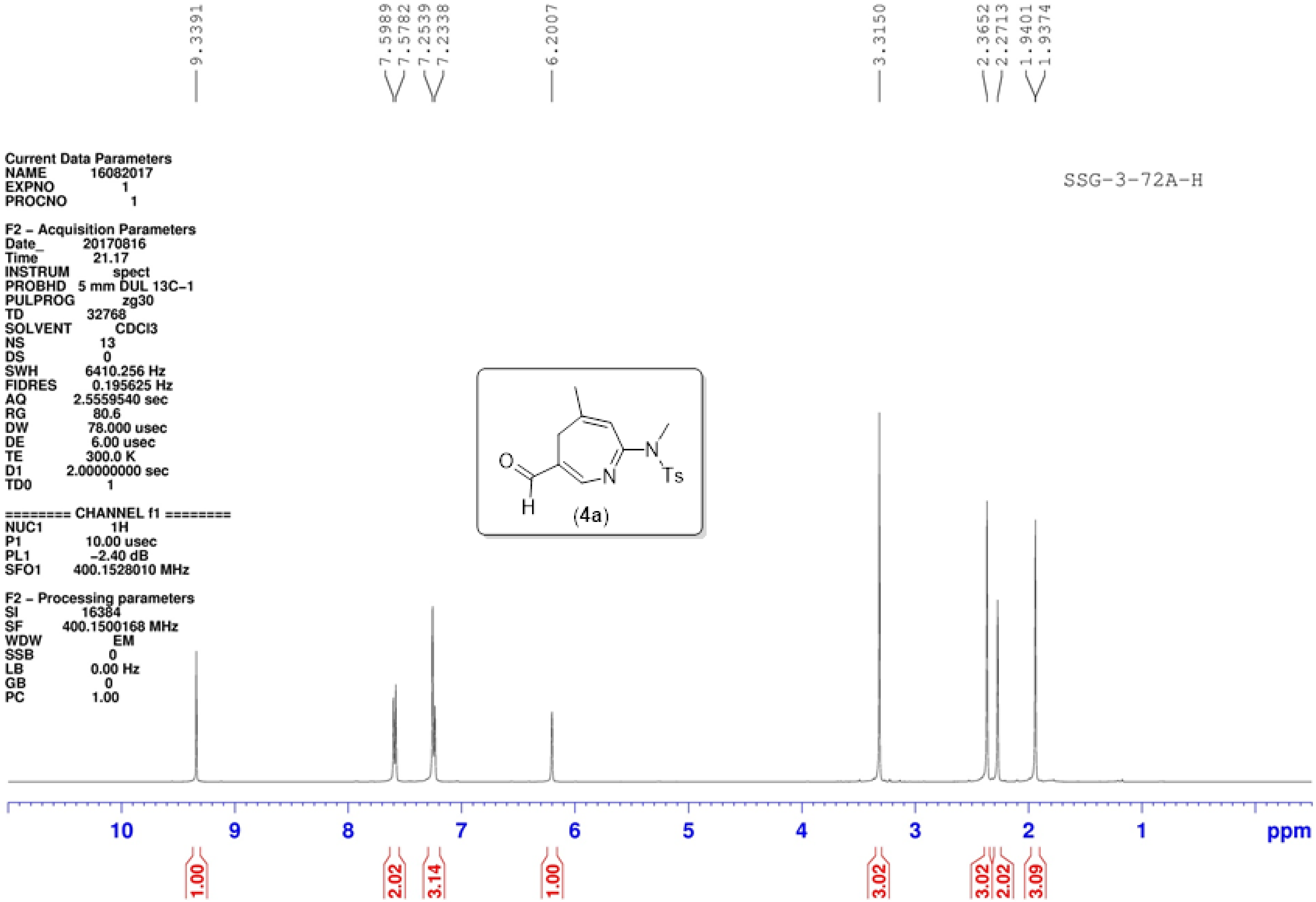
9.3391
7.5989
7.5782
7.2539
7.2338
6.2007
3.3150
2.3652
2.2713
1.9401
1.9374
Current Data Parameters
NAME 16082017
EXPNO 1
PROCNO 1
F2 - Acquisition Parameters
Date_ 20170816
Time 21.17
INSTRUM spect
PROBHD 5 mm DUL 13C-1
PULPROG zg30
TD 32768
SOLVENT CDCl3
NS 13
DS 0
SWH 6410.256 Hz
FIDRES 0.195625 Hz
AQ 2.5559540 sec
RG 80.6
DW 78.000 usec
DE 6.00 usec
TE 300.0 K
D1 2.00000000 sec
TD0 1
======== CHANNEL f1 ========
NUC1 1H
P1 10.00 usec
PL1 -2.40 dB
SFO1 400.1528010 MHz
F2 - Processing parameters
SI 16384
SF 400.1500168 MHz
WDW EM
SSB 0
LB 0.00 Hz
GB 0
PC 1.00
SSG-3-72A-H
O
H
N
N
Ts
(4a)
10
9
8
7
6
5
4
3
2
1
ppm
1.00
2.02
3.14
1.00
3.02
3.02
2.02
3.09

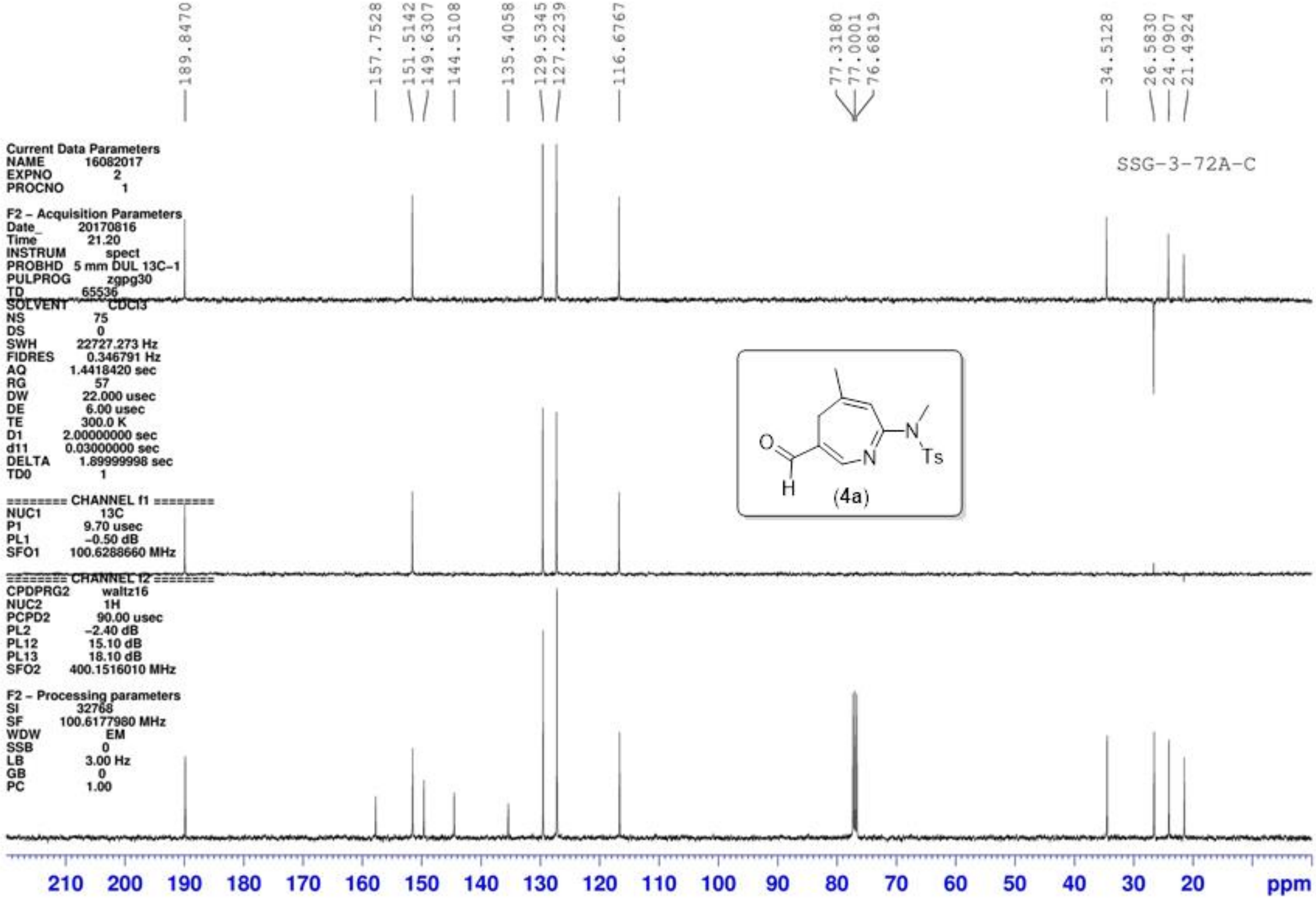
189.8470
157.7528
151.5142
149.6307
144.5108
135.4058
129.5345
127.2239
116.6767
77.3180
77.0001
76.6819
34.5128
26.5830
24.0907
21.4924
SSG-3-72A-C
Current Data Parameters
NAME 16082017
EXPNO 2
PROCNO 1
F2 - Acquisition Parameters
Date_ 20170816
Time 21.20
INSTRUM spect
PROBHD 5 mm DUL 13C-1
PULPROG zgpg30
TD 65536
SOLVENT CDCl3
NS 75
DS 0
SWH 22727.273 Hz
FIDRES 0.346791 Hz
AQ 1.4418420 sec
RG 57
DW 22.000 usec
DE 6.00 usec
TE 300.0 K
D1 2.00000000 sec
d11 0.03000000 sec
DELTA 1.89999998 sec
TD0 1
======== CHANNEL f1 ========
NUC1 13C
P1 9.70 usec
PL1 -0.50 dB
SFO1 100.6288660 MHz
======== CHANNEL f2 ========
CPDPRG2 waltz16
NUC2 1H
PCPD2 90.00 usec
PL2 -2.40 dB
PL12 15.10 dB
PL13 18.10 dB
SFO2 400.1516010 MHz
F2 - Processing parameters
SI 32768
SF 100.6177980 MHz
WDW EM
SSB 0
LB 3.00 Hz
GB 0
PC 1.00
O
H
N
N
Ts
(4a)
210 200 190 180 170 160 150 140 130 120 110 100 90 80 70 60 50 40 30 20 ppm

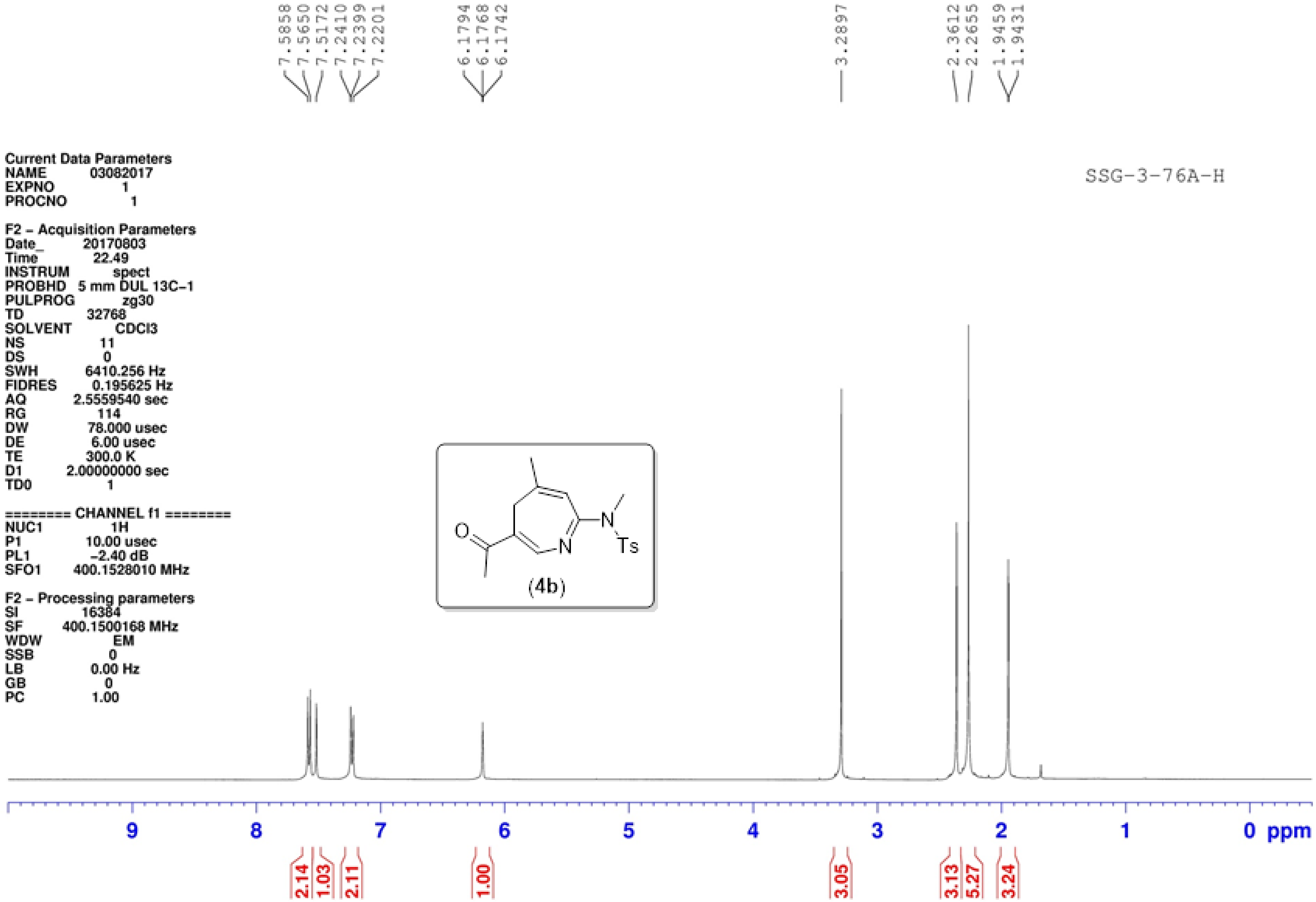
7.5858
7.5650
7.5172
7.2410
7.2399
7.2201
6.1794
6.1768
6.1742
3.2897
2.3612
2.2655
1.9459
1.9431
Current Data Parameters
NAME 03082017
EXPNO 1
PROCNO 1
F2 - Acquisition Parameters
Date_ 20170803
Time 22.49
INSTRUM spect
PROBHD 5 mm DUL 13C-1
PULPROG zg30
TD 32768
SOLVENT CDCl3
NS 11
DS 0
SWH 6410.256 Hz
FIDRES 0.195625 Hz
AQ 2.5559540 sec
RG 114
DW 78.000 usec
DE 6.00 usec
TE 300.0 K
D1 2.00000000 sec
TD0 1
======== CHANNEL f1 ========
NUC1 1H
P1 10.00 usec
PL1 -2.40 dB
SFO1 400.1528010 MHz
F2 - Processing parameters
SI 16384
SF 400.1500168 MHz
WDW EM
SSB 0
LB 0.00 Hz
GB 0
PC 1.00
SSG-3-76A-H
O
N
N
Ts
(4b)
9
8
7
6
5
4
3
2
1
0 ppm
2.14
1.03
2.11
1.00
3.05
3.13
5.27
3.24

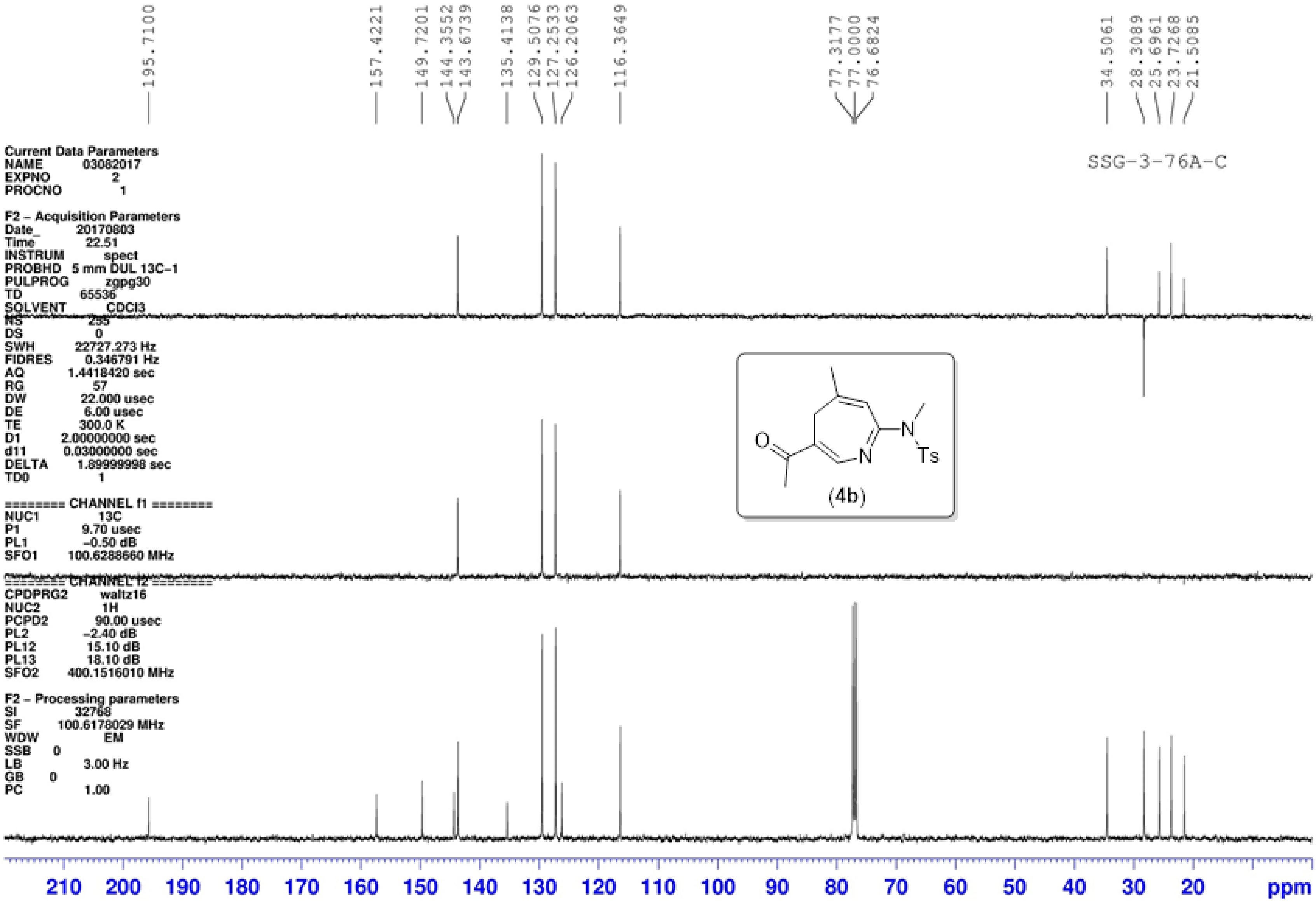
195.7100
157.4221
149.7201
144.3552
143.6739
135.4138
129.5076
127.2533
126.2063
116.3649
77.3177
77.0000
76.6824
34.5061
28.3089
25.6961
23.7268
21.5085
SSG-3-76A-C
Current Data Parameters
NAME 03082017
EXPNO 2
PROCNO 1
F2 – Acquisition Parameters
Date_ 20170803
Time 22.51
INSTRUM spect
PROBHD 5 mm DUL 13C-1
PULPROG zgpg30
TD 65536
SOLVENT CDCl3
NS 256
DS 0
SWH 22727.273 Hz
FIDRES 0.346791 Hz
AQ 1.4418420 sec
RG 57
DW 22.000 usec
DE 6.00 usec
TE 300.0 K
D1 2.00000000 sec
d11 0.03000000 sec
DELTA 1.89999998 sec
TD0 1
======== CHANNEL f1 ========
NUC1 13C
P1 9.70 usec
PL1 -0.50 dB
SFO1 100.6288660 MHz
======== CHANNEL f2 ========
CPDPRG2 waltz16
NUC2 1H
PCPD2 90.00 usec
PL2 -2.40 dB
PL12 15.10 dB
PL13 18.10 dB
SFO2 400.1516010 MHz
F2 – Processing parameters
SI 32768
SF 100.6178029 MHz
WDW EM
SSB 0
LB 3.00 Hz
GB 0
PC 1.00
O
N
N
Ts
(4b)
210 200 190 180 170 160 150 140 130 120 110 100 90 80 70 60 50 40 30 20 ppm

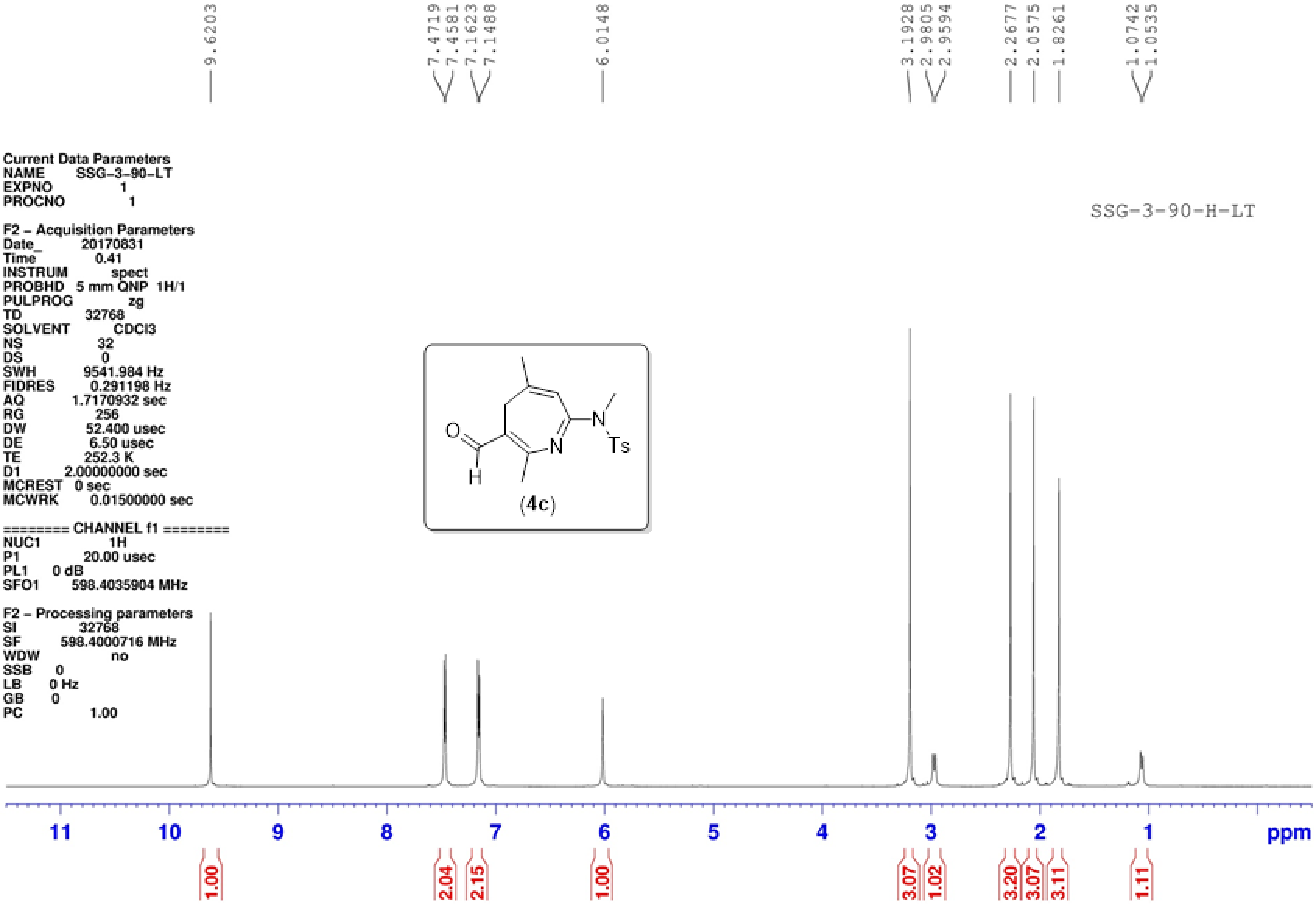
9.6203
7.4719
7.4581
7.1623
7.1488
6.0148
3.1928
2.9805
2.9594
2.2677
2.0575
1.8261
1.0742
1.0535
Current Data Parameters
NAME SSG-3-90-LT
EXPNO 1
PROCNO 1
F2 - Acquisition Parameters
Date_ 20170831
Time 0.41
INSTRUM spect
PROBHD 5 mm QNP 1H/1
PULPROG zg
TD 32768
SOLVENT CDCl3
NS 32
DS 0
SWH 9541.984 Hz
FIDRES 0.291198 Hz
AQ 1.7170932 sec
RG 256
DW 52.400 usec
DE 6.50 usec
TE 252.3 K
D1 2.00000000 sec
MCREST 0 sec
MCWRK 0.01500000 sec
======== CHANNEL f1 ========
NUC1 1H
P1 20.00 usec
PL1 0 dB
SFO1 598.4035904 MHz
F2 - Processing parameters
SI 32768
SF 598.4000716 MHz
WDW no
SSB 0
LB 0 Hz
GB 0
PC 1.00
SSG-3-90-H-LT
O
H
N
N
Ts
(4c)
11
10
9
8
7
6
5
4
3
2
1
ppm
1.00
2.04
2.15
1.00
3.07
1.02
3.20
3.07
3.11
1.11

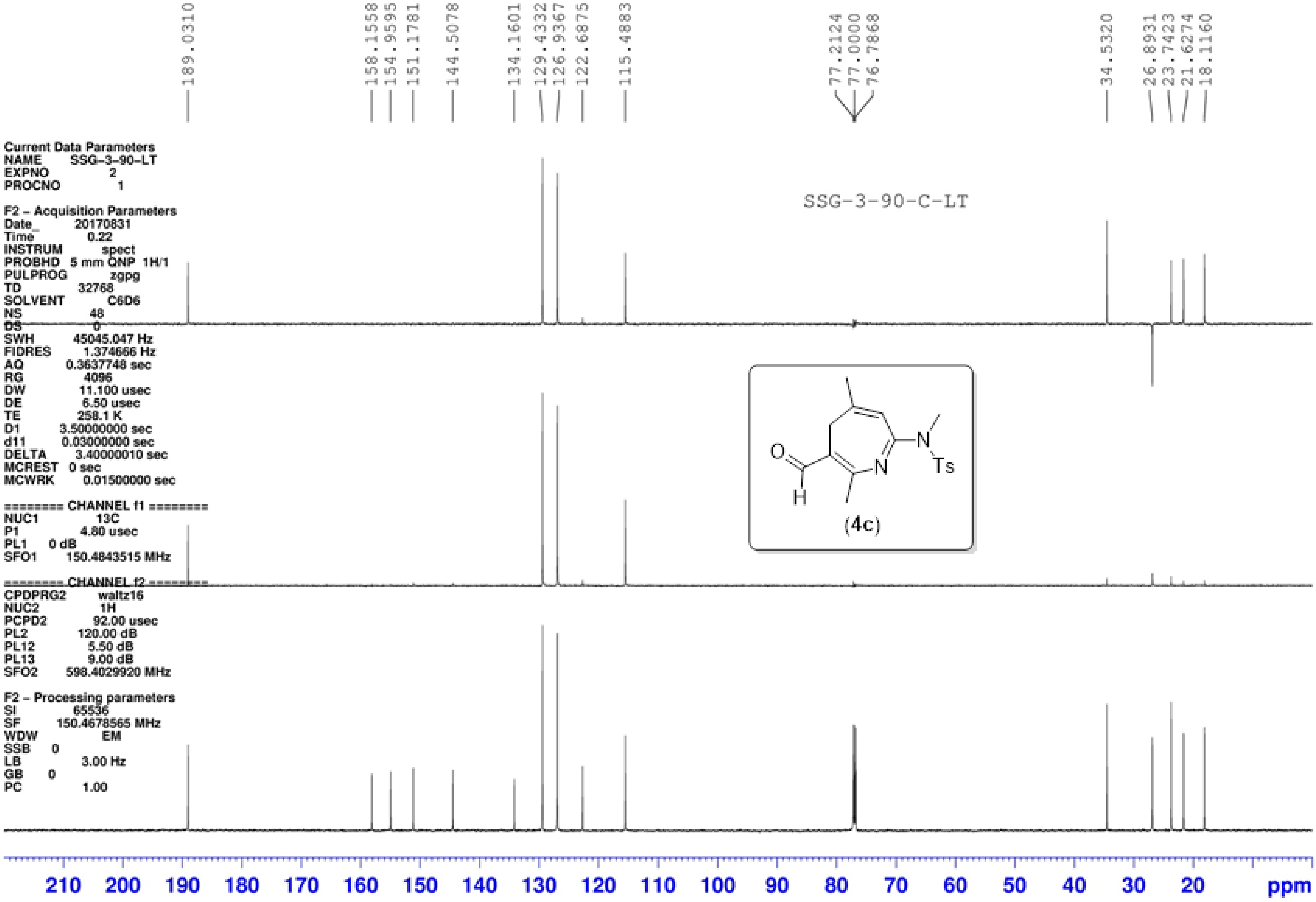

189.0310
158.1558
154.9595
151.1781
144.5078
134.1601
129.4332
126.9367
122.6875
115.4883
77.2124
77.0000
76.7868
34.5320
26.8931
23.7423
21.6274
18.1160
Current Data Parameters
NAME SSG-3-90-LT
EXPNO 2
PROCNO 1
F2 - Acquisition Parameters
Date_ 20170831
Time 0.22
INSTRUM spect
PROBHD 5 mm QNP 1H/1
PULPROG zgpg
TD 32768
SOLVENT C6D6
NS 48
DS 0
SWH 45045.047 Hz
FIDRES 1.374666 Hz
AQ 0.3637748 sec
RG 4096
DW 11.100 usec
DE 6.50 usec
TE 258.1 K
D1 3.50000000 sec
d11 0.03000000 sec
DELTA 3.40000010 sec
MCREST 0 sec
MCWRK 0.01500000 sec
======== CHANNEL f1 ========
NUC1 13C
P1 4.80 usec
PL1 0 dB
SFO1 150.4843515 MHz
======== CHANNEL f2 ========
CPDPRG2 waltz16
NUC2 1H
PCPD2 92.00 usec
PL2 120.00 dB
PL12 5.50 dB
PL13 9.00 dB
SFO2 598.4029920 MHz
F2 - Processing parameters
SI 65536
SF 150.4678565 MHz
WDW EM
SSB 0
LB 3.00 Hz
GB 0
PC 1.00
SSG-3-90-C-LT
O
H
N
N
Ts
(4c)
210 200 190 180 170 160 150 140 130 120 110 100 90 80 70 60 50 40 30 20 ppm

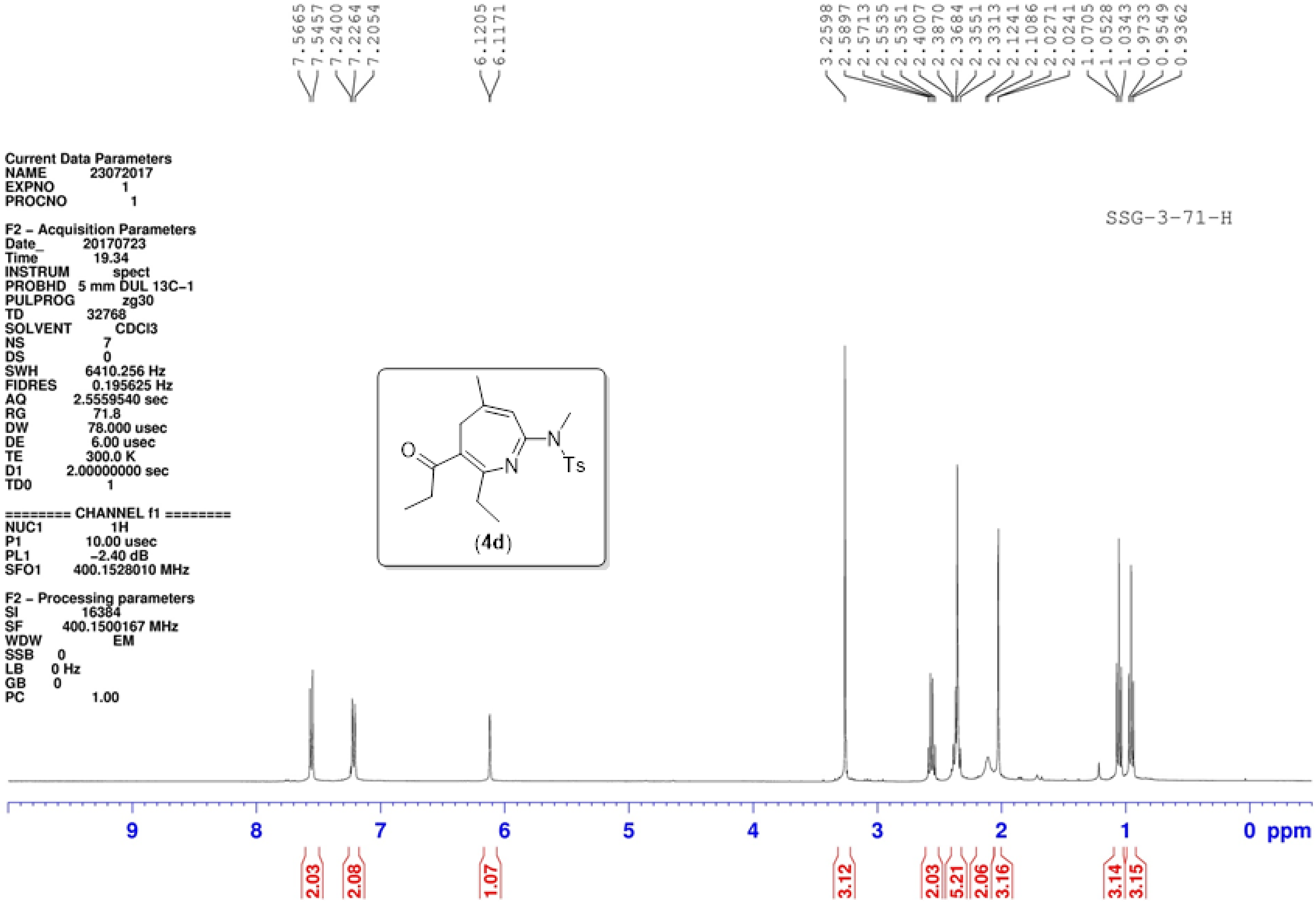
7.5665
7.5457
7.2400
7.2264
7.2054
6.1205
6.1171
3.2598
2.5897
2.5713
2.5535
2.5351
2.4007
2.3870
2.3684
2.3551
2.3313
2.1241
2.1086
2.0271
2.0241
1.0705
1.0528
1.0343
0.9733
0.9549
0.9362
Current Data Parameters
NAME 23072017
EXPNO 1
PROCNO 1
F2 - Acquisition Parameters
Date_ 20170723
Time 19.34
INSTRUM spect
PROBHD 5 mm DUL 13C-1
PULPROG zg30
TD 32768
SOLVENT CDCl3
NS 7
DS 0
SWH 6410.256 Hz
FIDRES 0.195625 Hz
AQ 2.5559540 sec
RG 71.8
DW 78.000 usec
DE 6.00 usec
TE 300.0 K
D1 2.00000000 sec
TD0 1
======== CHANNEL f1 ========
NUC1 1H
P1 10.00 usec
PL1 -2.40 dB
SFO1 400.1528010 MHz
F2 - Processing parameters
SI 16384
SF 400.1500167 MHz
WDW EM
SSB 0
LB 0 Hz
GB 0
PC 1.00
SSG-3-71-H
O
N
N
Ts
(4d)
9
8
7
6
5
4
3
2
1
0 ppm
2.03
2.08
1.07
3.12
2.03
5.21
2.06
3.16
3.14
3.15

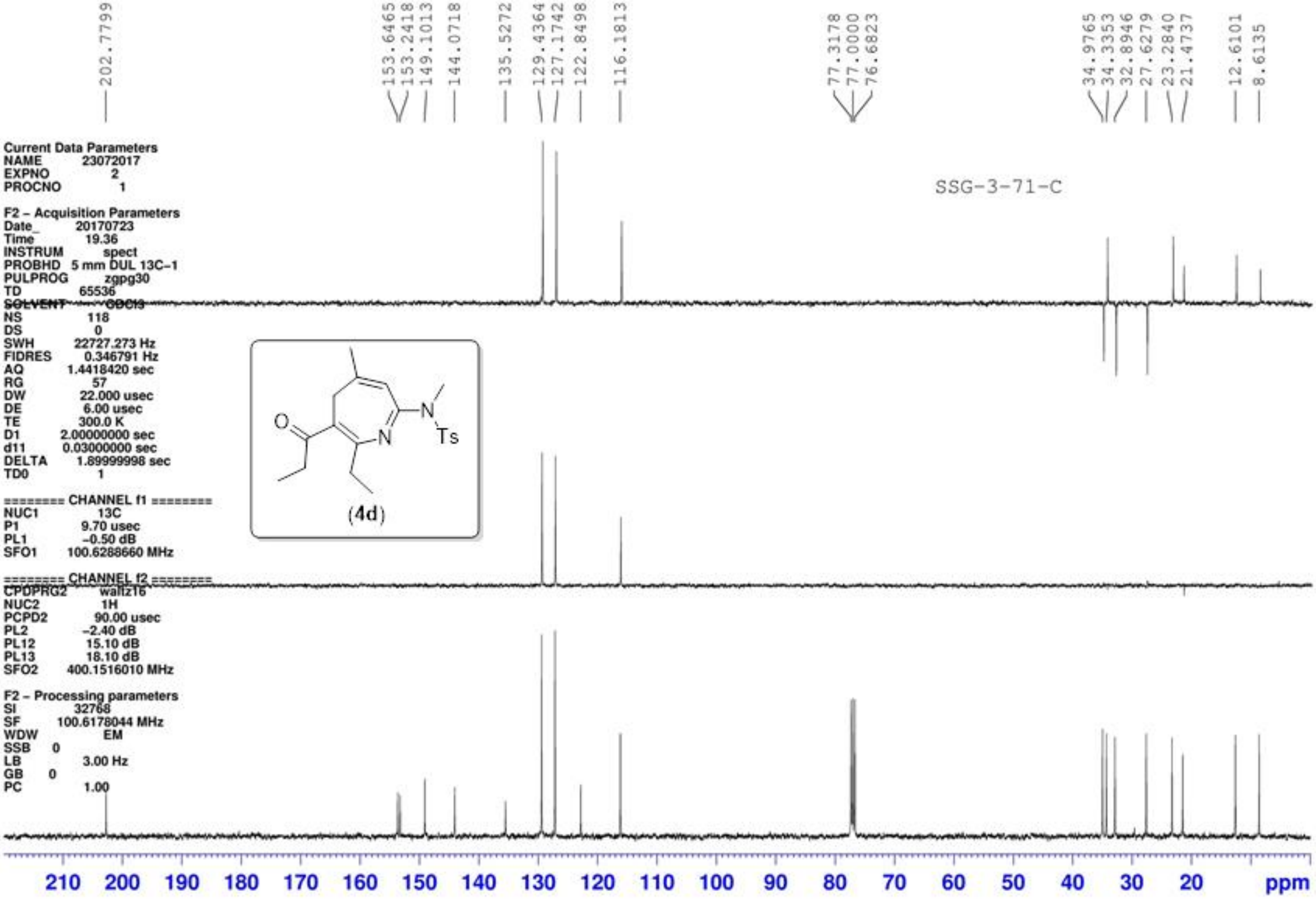
202.7799
153.6465
153.2418
149.1013
144.0718
135.5272
129.4364
127.1742
122.8498
116.1813
77.3178
77.0000
76.6823
34.9765
34.3353
32.8946
27.6279
23.2840
21.4737
12.6101
8.6135
SSG-3-71-C
Current Data Parameters
NAME 23072017
EXPNO 2
PROCNO 1
F2 - Acquisition Parameters
Date_ 20170723
Time 19.36
INSTRUM spect
PROBHD 5 mm DUL 13C-1
PULPROG zgpg30
TD 65536
SOLVENT CDCl3
NS 118
DS 0
SWH 22727.273 Hz
FIDRES 0.346791 Hz
AQ 1.4418420 sec
RG 57
DW 22.000 usec
DE 6.00 usec
TE 300.0 K
D1 2.00000000 sec
d11 0.03000000 sec
DELTA 1.89999998 sec
TD0 1
======== CHANNEL f1 ========
NUC1 13C
P1 9.70 usec
PL1 -0.50 dB
SFO1 100.6288660 MHz
======== CHANNEL f2 ========
CPDPRG2 waltz16
NUC2 1H
PCPD2 90.00 usec
PL2 -2.40 dB
PL12 15.10 dB
PL13 18.10 dB
SFO2 400.1516010 MHz
F2 - Processing parameters
SI 32768
SF 100.6178044 MHz
WDW EM
SSB 0
LB 3.00 Hz
GB 0
PC 1.00
O
N
Ts
(4d)
210 200 190 180 170 160 150 140 130 120 110 100 90 80 70 60 50 40 30 20 ppm

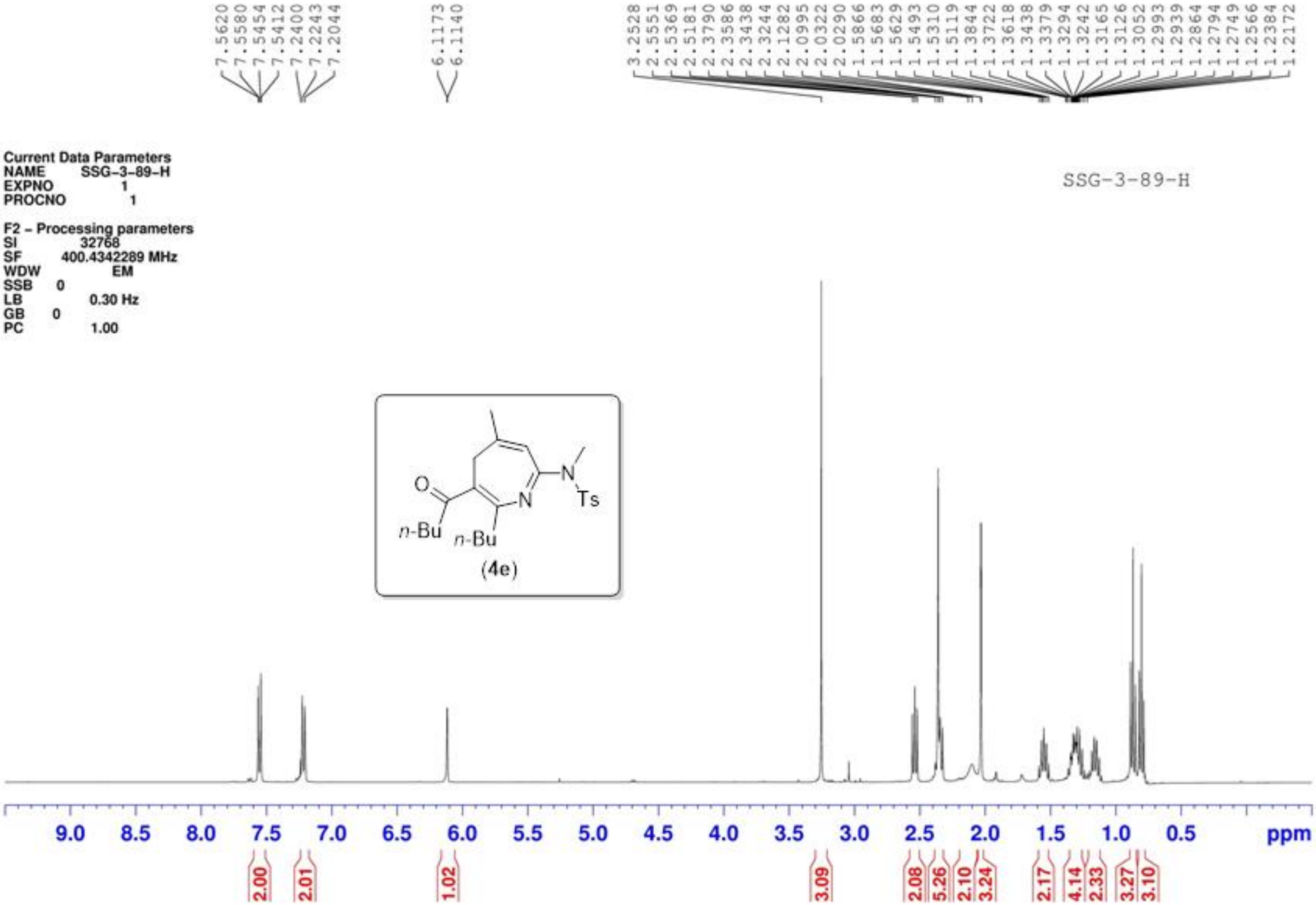
7.5620
7.5580
7.5454
7.5412
7.2400
7.2243
7.2044
6.1173
6.1140
3.2528
2.5551
2.5369
2.5181
2.3790
2.3586
2.3438
2.3244
2.1282
2.0995
2.0322
2.0290
1.5866
1.5683
1.5629
1.5493
1.5310
1.5119
1.3844
1.3722
1.3618
1.3438
1.3379
1.3294
1.3242
1.3165
1.3126
1.3052
1.2993
1.2939
1.2864
1.2794
1.2749
1.2566
1.2384
1.2172
Current Data Parameters
NAME SSG-3-89-H
EXPNO 1
PROCNO 1
F2 - Processing parameters
SI 32768
SF 400.4342289 MHz
WDW EM
SSB 0
LB 0.30 Hz
GB 0
PC 1.00
SSG-3-89-H
O
n-Bu
n-Bu
N
N
Ts
(4e)
9.0
8.5
8.0
7.5
7.0
6.5
6.0
5.5
5.0
4.5
4.0
3.5
3.0
2.5
2.0
1.5
1.0
0.5
ppm
2.00
2.01
1.02
3.09
2.08
5.26
2.10
3.24
2.17
4.14
2.33
3.27
3.10

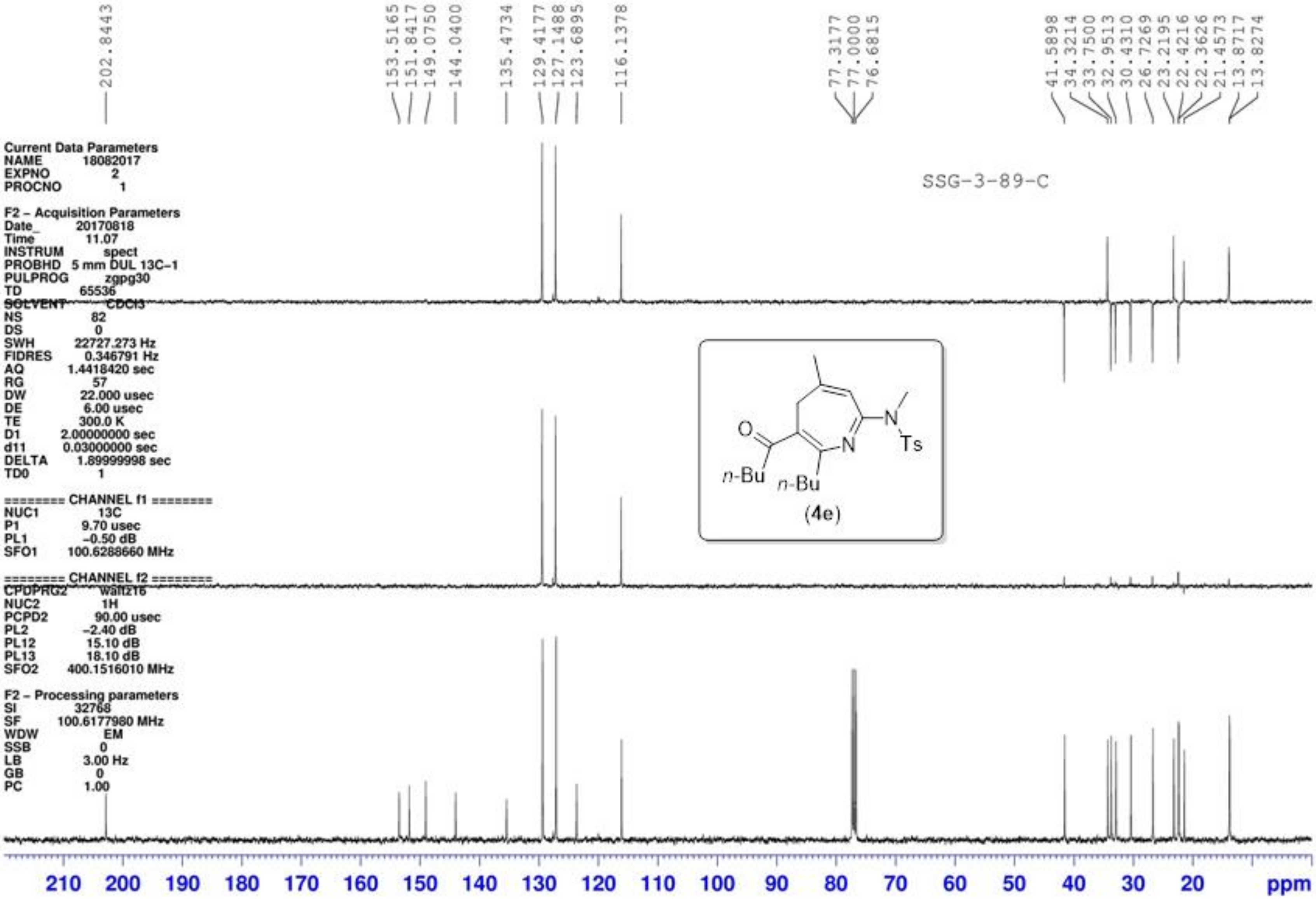
202.8443
153.5165
151.8417
149.0750
144.0400
135.4734
129.4177
127.1488
123.6895
116.1378
77.3177
77.0000
76.6815
41.5898
34.3214
33.7500
32.9513
30.4310
26.7269
23.2195
22.4216
22.3626
21.4573
13.8717
13.8274
Current Data Parameters
NAME 18082017
EXPNO 2
PROCNO 1
F2 - Acquisition Parameters
Date_ 20170818
Time 11.07
INSTRUM spect
PROBHD 5 mm DUL 13C-1
PULPROG zgpg30
TD 65536
SOLVENT CDCl3
NS 82
DS 0
SWH 22727.273 Hz
FIDRES 0.346791 Hz
AQ 1.4418420 sec
RG 57
DW 22.000 usec
DE 6.00 usec
TE 300.0 K
D1 2.00000000 sec
d11 0.03000000 sec
DELTA 1.89999998 sec
TD0 1
======== CHANNEL f1 ========
NUC1 13C
P1 9.70 usec
PL1 -0.50 dB
SFO1 100.6288660 MHz
======== CHANNEL f2 ========
CPDPRG2 waltz16
NUC2 1H
PCPD2 90.00 usec
PL2 -2.40 dB
PL12 15.10 dB
PL13 18.10 dB
SFO2 400.1516010 MHz
F2 - Processing parameters
SI 32768
SF 100.6177980 MHz
WDW EM
SSB 0
LB 3.00 Hz
GB 0
PC 1.00
SSG-3-89-C
O
N
N
Ts
n-Bu
n-Bu
(4e)
210 200 190 180 170 160 150 140 130 120 110 100 90 80 70 60 50 40 30 20 ppm

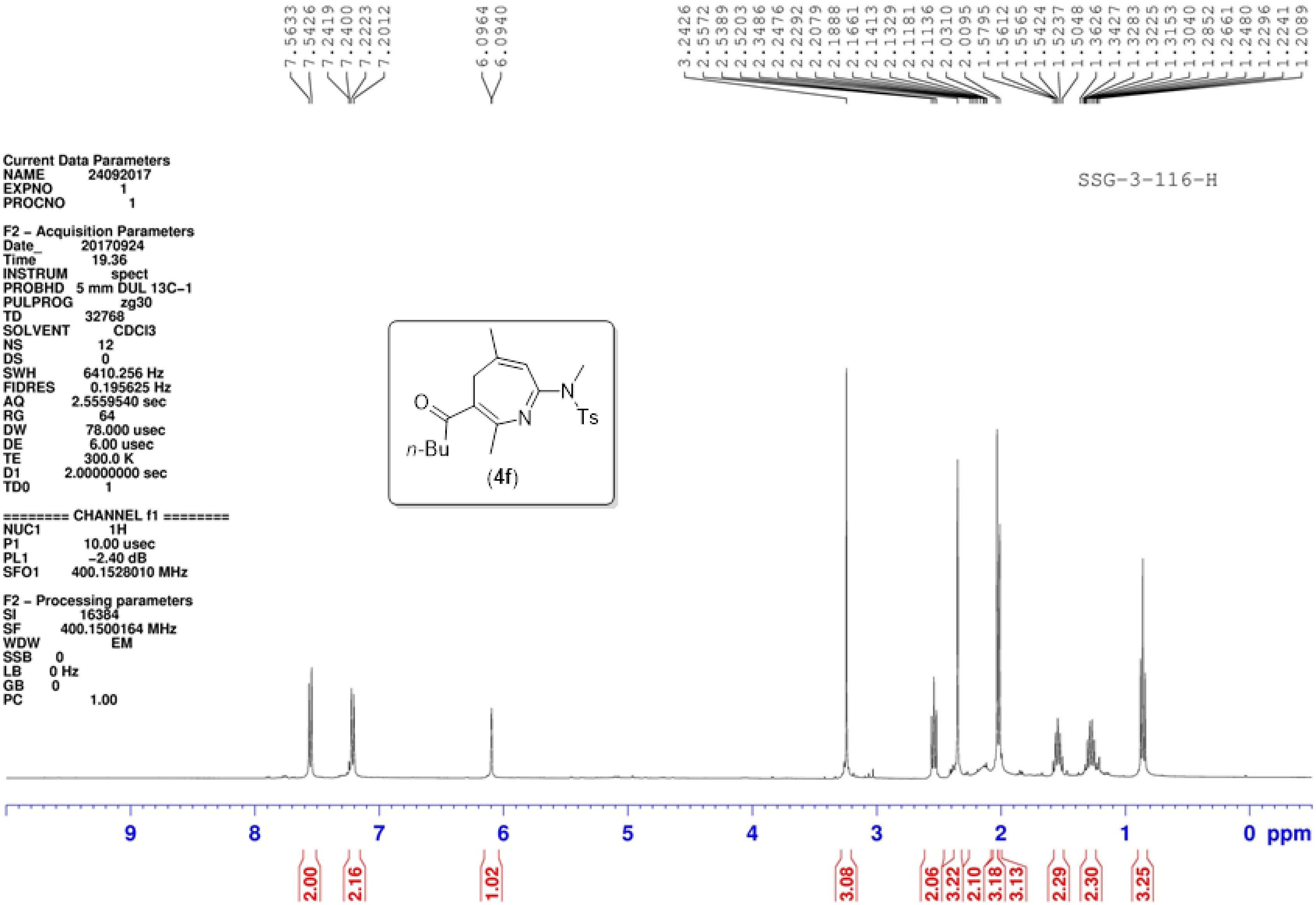
Current Data Parameters
NAME 24092017
EXPNO 1
PROCNO 1
F2 - Acquisition Parameters
Date_ 20170924
Time 19.36
INSTRUM spect
PROBHD 5 mm DUL 13C-1
PULPROG zg30
TD 32768
SOLVENT CDCl3
NS 12
DS 0
SWH 6410.256 Hz
FIDRES 0.195625 Hz
AQ 2.5559540 sec
RG 64
DW 78.000 usec
DE 6.00 usec
TE 300.0 K
D1 2.00000000 sec
TD0 1
======== CHANNEL f1 ========
NUC1 1H
P1 10.00 usec
PL1 -2.40 dB
SFO1 400.1528010 MHz
F2 - Processing parameters
SI 16384
SF 400.1500164 MHz
WDW EM
SSB 0
LB 0 Hz
GB 0
PC 1.00
SSG-3-116-H
7.5633
7.5426
7.2419
7.2400
7.2223
7.2012
6.0964
6.0940
3.2426
2.5572
2.5389
2.5203
2.3486
2.2476
2.2292
2.2079
2.1888
2.1661
2.1413
2.1329
2.1181
2.1136
2.0310
2.0095
1.5795
1.5612
1.5565
1.5424
1.5237
1.5048
1.3626
1.3427
1.3283
1.3225
1.3153
1.3040
1.2852
1.2661
1.2480
1.2296
1.2241
1.2089
O
n-Bu
N
N
Ts
(4f)
9
8
7
6
5
4
3
2
1
0 ppm
2.00
2.16
1.02
3.08
2.06
3.22
2.10
3.18
3.13
2.29
2.30
3.25

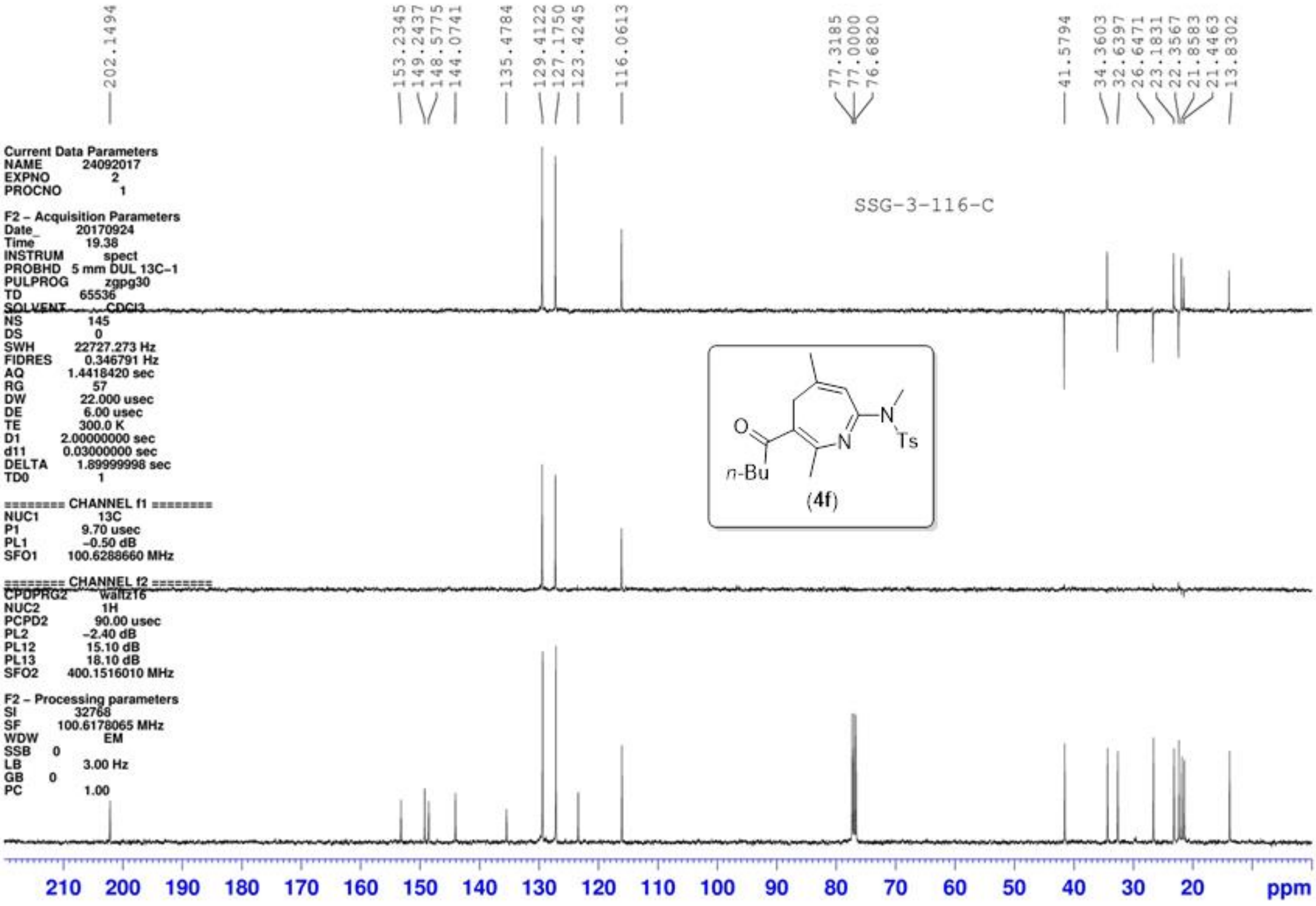
202.1494
153.2345
149.2437
148.5775
144.0741
135.4784
129.4122
127.1750
123.4245
116.0613
77.3185
77.0000
76.6820
41.5794
34.3603
32.6397
26.6471
23.1831
22.3567
21.8583
21.4463
13.8302
Current Data Parameters
NAME 24092017
EXPNO 2
PROCNO 1
F2 - Acquisition Parameters
Date_ 20170924
Time 19.38
INSTRUM spect
PROBHD 5 mm DUL 13C-1
PULPROG zgpg30
TD 65536
SOLVENT CDCl3
NS 145
DS 0
SWH 22727.273 Hz
FIDRES 0.346791 Hz
AQ 1.4418420 sec
RG 57
DW 22.000 usec
DE 6.00 usec
TE 300.0 K
D1 2.00000000 sec
d11 0.03000000 sec
DELTA 1.89999998 sec
TD0 1
======== CHANNEL f1 ========
NUC1 13C
P1 9.70 usec
PL1 -0.50 dB
SFO1 100.6288660 MHz
======== CHANNEL f2 ========
CPDPRG2 waltz16
NUC2 1H
PCPD2 90.00 usec
PL2 -2.40 dB
PL12 15.10 dB
PL13 18.10 dB
SFO2 400.1516010 MHz
F2 - Processing parameters
SI 32768
SF 100.6178065 MHz
WDW EM
SSB 0
LB 3.00 Hz
GB 0
PC 1.00
SSG-3-116-C
O
N
N
Ts
n-Bu
(4f)
210
200
190
180
170
160
150
140
130
120
110
100
90
80
70
60
50
40
30
20
ppm

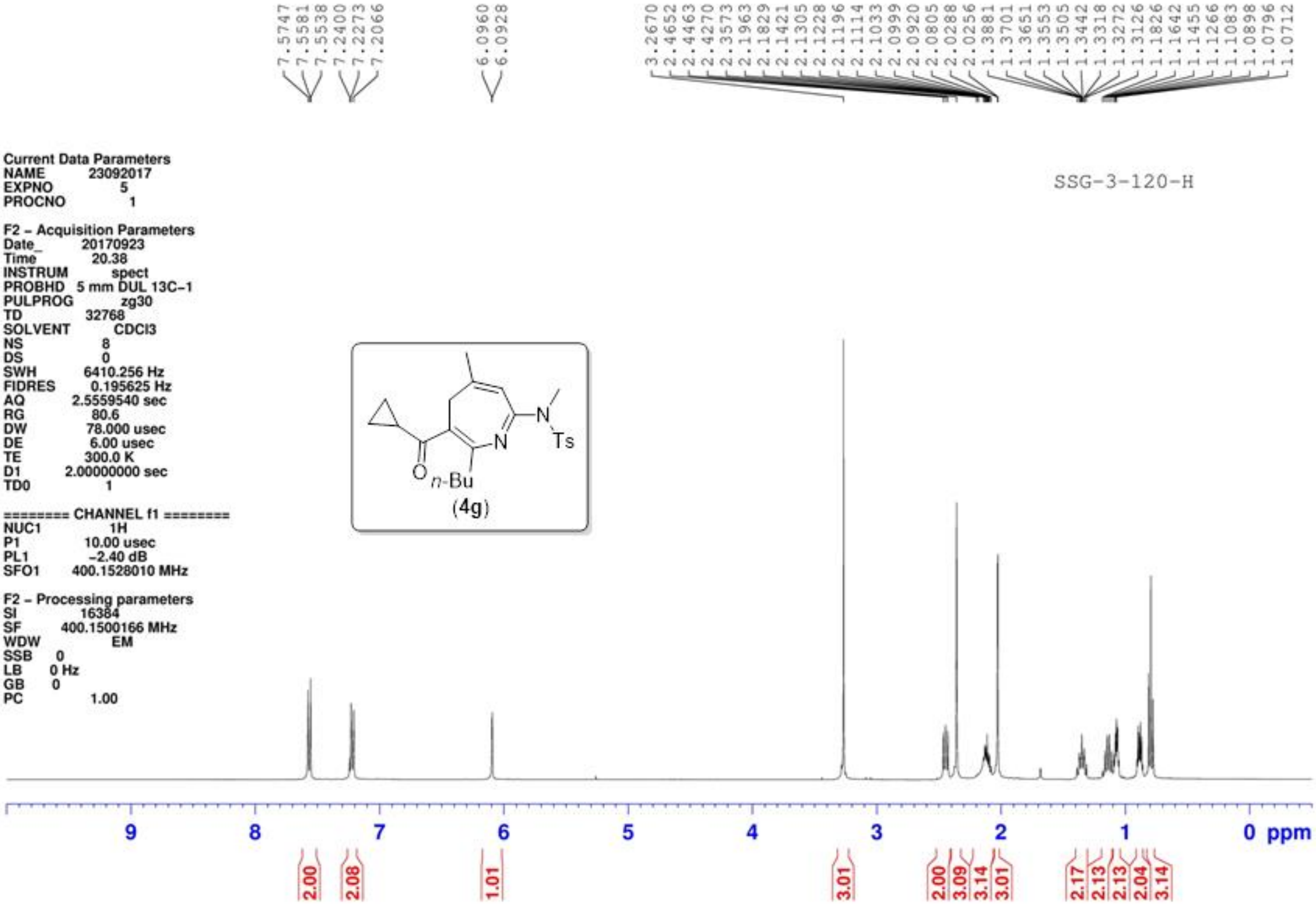
7.5747
7.5581
7.5538
7.2400
7.2273
7.2066
6.0960
6.0928
3.2670
2.4652
2.4463
2.4270
2.3573
2.1963
2.1829
2.1421
2.1305
2.1228
2.1196
2.1114
2.1033
2.0999
2.0920
2.0805
2.0288
2.0256
1.3881
1.3701
1.3651
1.3553
1.3505
1.3442
1.3318
1.3272
1.3126
1.1826
1.1642
1.1455
1.1266
1.1083
1.0898
1.0796
1.0712
Current Data Parameters
NAME 23092017
EXPNO 5
PROCNO 1
F2 - Acquisition Parameters
Date_ 20170923
Time 20.38
INSTRUM spect
PROBHD 5 mm DUL 13C-1
PULPROG zg30
TD 32768
SOLVENT CDCl3
NS 8
DS 0
SWH 6410.256 Hz
FIDRES 0.195625 Hz
AQ 2.5559540 sec
RG 80.6
DW 78.000 usec
DE 6.00 usec
TE 300.0 K
D1 2.00000000 sec
TD0 1
======== CHANNEL f1 ========
NUC1 1H
P1 10.00 usec
PL1 -2.40 dB
SFO1 400.1528010 MHz
F2 - Processing parameters
SI 16384
SF 400.1500166 MHz
WDW EM
SSB 0
LB 0 Hz
GB 0
PC 1.00
SSG-3-120-H
N
Ts
O
n-Bu
(4g)
9
8
7
6
5
4
3
2
1
0 ppm
2.00
2.08
1.01
3.01
2.00
3.09
3.14
3.01
2.17
2.13
2.13
2.04
3.14

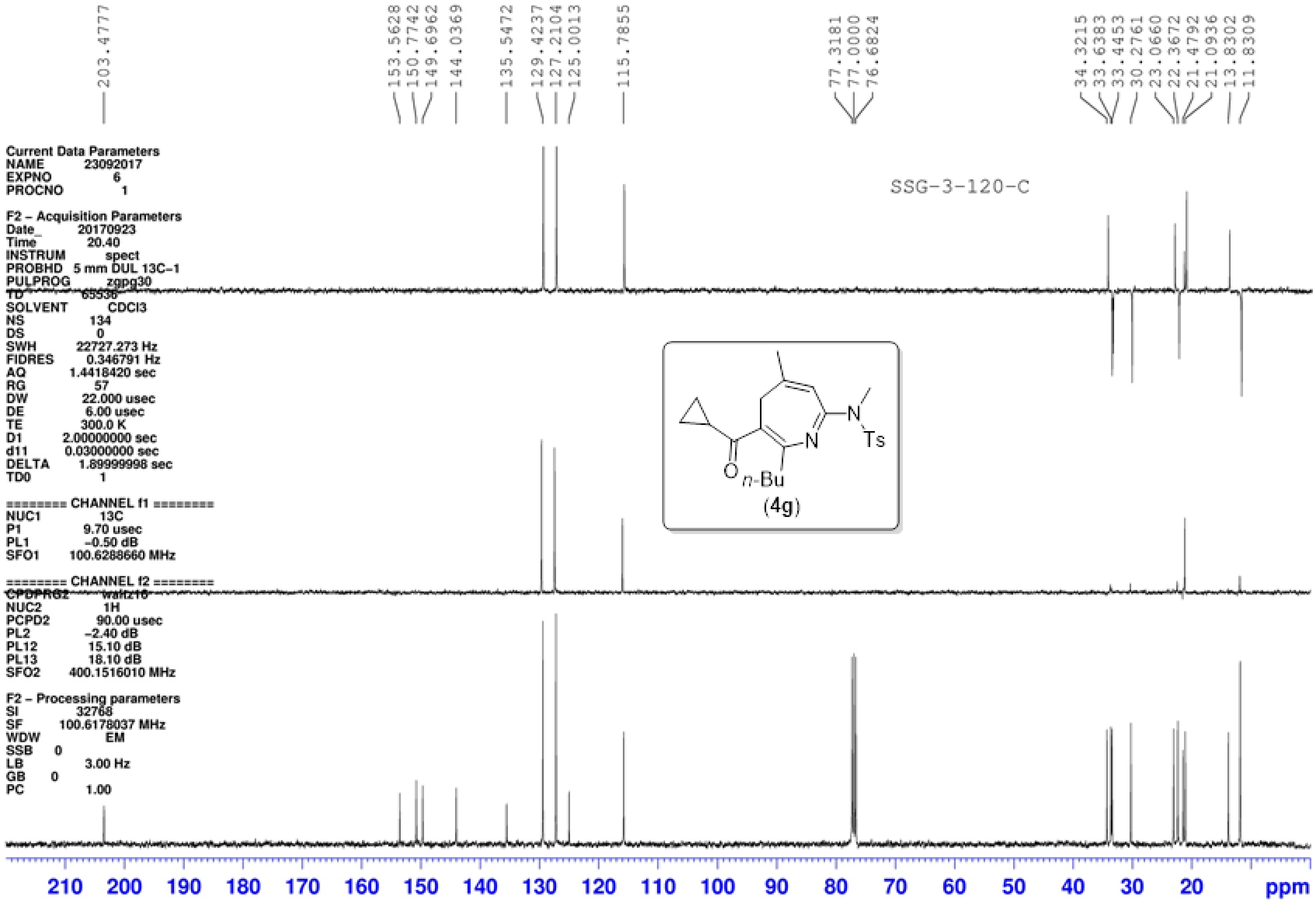
203.4777
153.5628
150.7742
149.6962
144.0369
135.5472
129.4237
127.2104
125.0013
115.7855
77.3181
77.0000
76.6824
34.3215
33.6383
33.4453
30.2761
23.0660
22.3672
21.4792
21.0936
13.8302
11.8309
SSG-3-120-C
Current Data Parameters
NAME 23092017
EXPNO 6
PROCNO 1
F2 - Acquisition Parameters
Date_ 20170923
Time 20.40
INSTRUM spect
PROBHD 5 mm DUL 13C-1
PULPROG zgpg30
TD 65536
SOLVENT CDCl3
NS 134
DS 0
SWH 22727.273 Hz
FIDRES 0.346791 Hz
AQ 1.4418420 sec
RG 57
DW 22.000 usec
DE 6.00 usec
TE 300.0 K
D1 2.00000000 sec
d11 0.03000000 sec
DELTA 1.89999998 sec
TD0 1
======== CHANNEL f1 ========
NUC1 13C
P1 9.70 usec
PL1 -0.50 dB
SFO1 100.6288660 MHz
======== CHANNEL f2 ========
CPDPRG2 waltz16
NUC2 1H
PCPD2 90.00 usec
PL2 -2.40 dB
PL12 15.10 dB
PL13 18.10 dB
SFO2 400.1516010 MHz
F2 - Processing parameters
SI 32768
SF 100.6178037 MHz
WDW EM
SSB 0
LB 3.00 Hz
GB 0
PC 1.00
N
Ts
O
n-Bu
(4g)
210 200 190 180 170 160 150 140 130 120 110 100 90 80 70 60 50 40 30 20 ppm

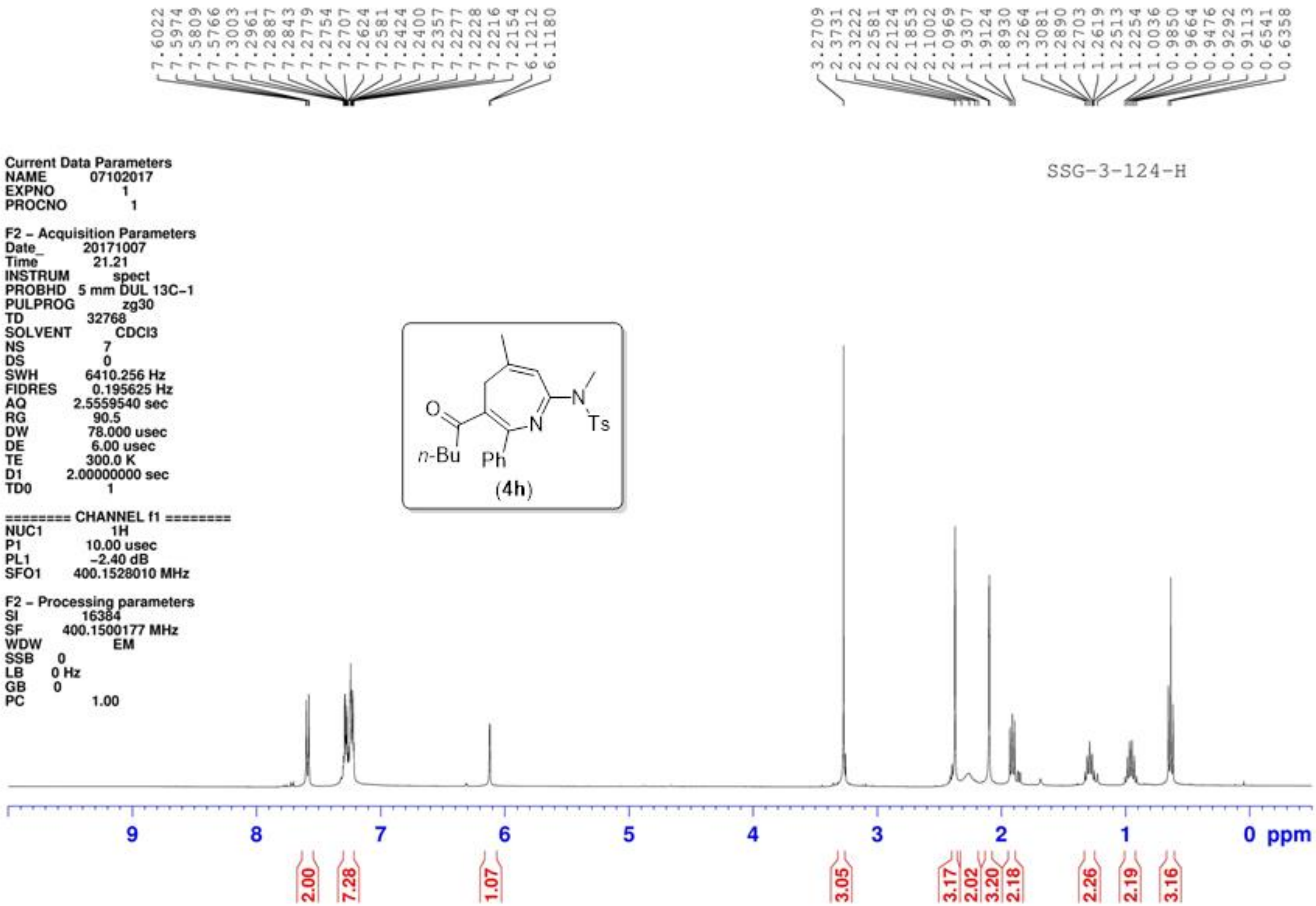
7.6022 7.5974 7.5809 7.5766 7.3003 7.2961 7.2887 7.2843 7.2779 7.2754 7.2707 7.2624 7.2581 7.2424 7.2400 7.2357 7.2277 7.2228 7.2216 7.2154 6.1212 6.1180
3.2709 2.3731 2.3222 2.2581 2.2124 2.1853 2.1002 2.0969 1.9307 1.9124 1.8930 1.3264 1.3081 1.2890 1.2703 1.2619 1.2513 1.2254 1.0036 0.9850 0.9664 0.9476 0.9292 0.9113 0.6541 0.6358
SSG-3-124-H
Current Data Parameters
NAME 07102017
EXPNO 1
PROCNO 1
F2 - Acquisition Parameters
Date_ 20171007
Time 21.21
INSTRUM spect
PROBHD 5 mm DUL 13C-1
PULPROG zg30
TD 32768
SOLVENT CDCl3
NS 7
DS 0
SWH 6410.256 Hz
FIDRES 0.195625 Hz
AQ 2.5559540 sec
RG 90.5
DW 78.000 usec
DE 6.00 usec
TE 300.0 K
D1 2.00000000 sec
TD0 1
======== CHANNEL f1 ========
NUC1 1H
P1 10.00 usec
PL1 -2.40 dB
SFO1 400.1528010 MHz
F2 - Processing parameters
SI 16384
SF 400.1500177 MHz
WDW EM
SSB 0
LB 0 Hz
GB 0
PC 1.00
O
N
N
Ts
n-Bu
Ph
(4h)
9 8 7 6 5 4 3 2 1 0 ppm
2.00 7.28 1.07 3.05 3.17 2.02 3.20 2.18 2.26 2.19 3.16

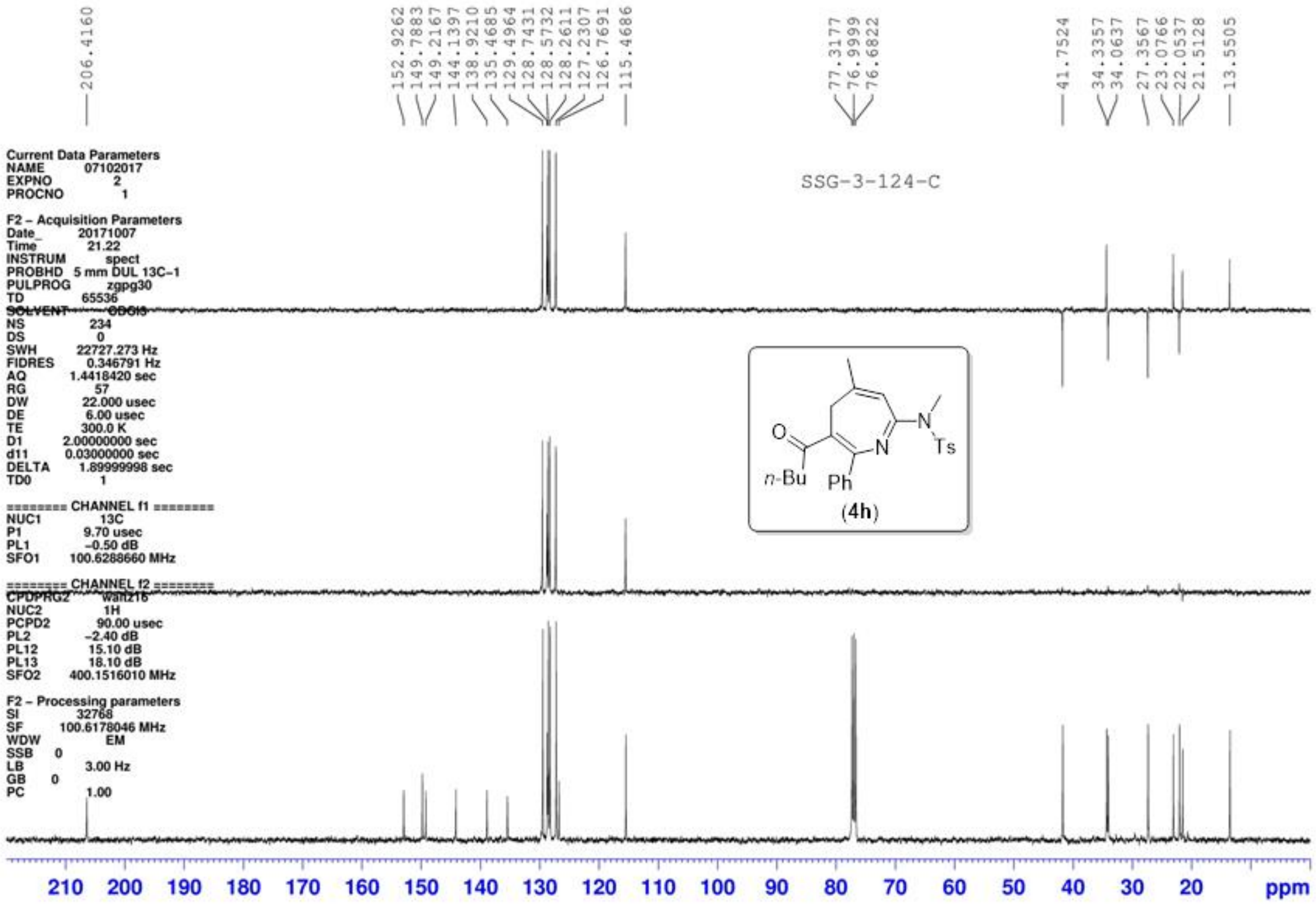

206.4160
152.9262
149.7883
149.2167
144.1397
138.9210
135.4685
129.4964
128.7431
128.5732
128.2611
127.2307
126.7691
115.4686
77.3177
76.9999
76.6822
41.7524
34.3357
34.0637
27.3567
23.0766
22.0537
21.5128
13.5505
Current Data Parameters
NAME 07102017
EXPNO 2
PROCNO 1
F2 - Acquisition Parameters
Date_ 20171007
Time 21.22
INSTRUM spect
PROBHD 5 mm DUL 13C-1
PULPROG zgpg30
TD 65536
SOLVENT CDCl3
NS 234
DS 0
SWH 22727.273 Hz
FIDRES 0.346791 Hz
AQ 1.4418420 sec
RG 57
DW 22.000 usec
DE 6.00 usec
TE 300.0 K
D1 2.00000000 sec
d11 0.03000000 sec
DELTA 1.89999998 sec
TD0 1
======== CHANNEL f1 ========
NUC1 13C
P1 9.70 usec
PL1 -0.50 dB
SFO1 100.6288660 MHz
======== CHANNEL f2 ========
CPDPRG2 waltz16
NUC2 1H
PCPD2 90.00 usec
PL2 -2.40 dB
PL12 15.10 dB
PL13 18.10 dB
SFO2 400.1516010 MHz
F2 - Processing parameters
SI 32768
SF 100.6178046 MHz
WDW EM
SSB 0
LB 3.00 Hz
GB 0
PC 1.00
SSG-3-124-C
O
N
N
Ts
n-Bu
Ph
(4h)
210 200 190 180 170 160 150 140 130 120 110 100 90 80 70 60 50 40 30 20 ppm

Current Data Parameters
NAME SSG-3-88A-H
EXPNO 1
PROCNO 1

F2 - Processing parameters
SI 32768
SF 400.4342286 MHz
WDW EM
SSB 0
LB 0.30 Hz
GB 0
PC 1.00

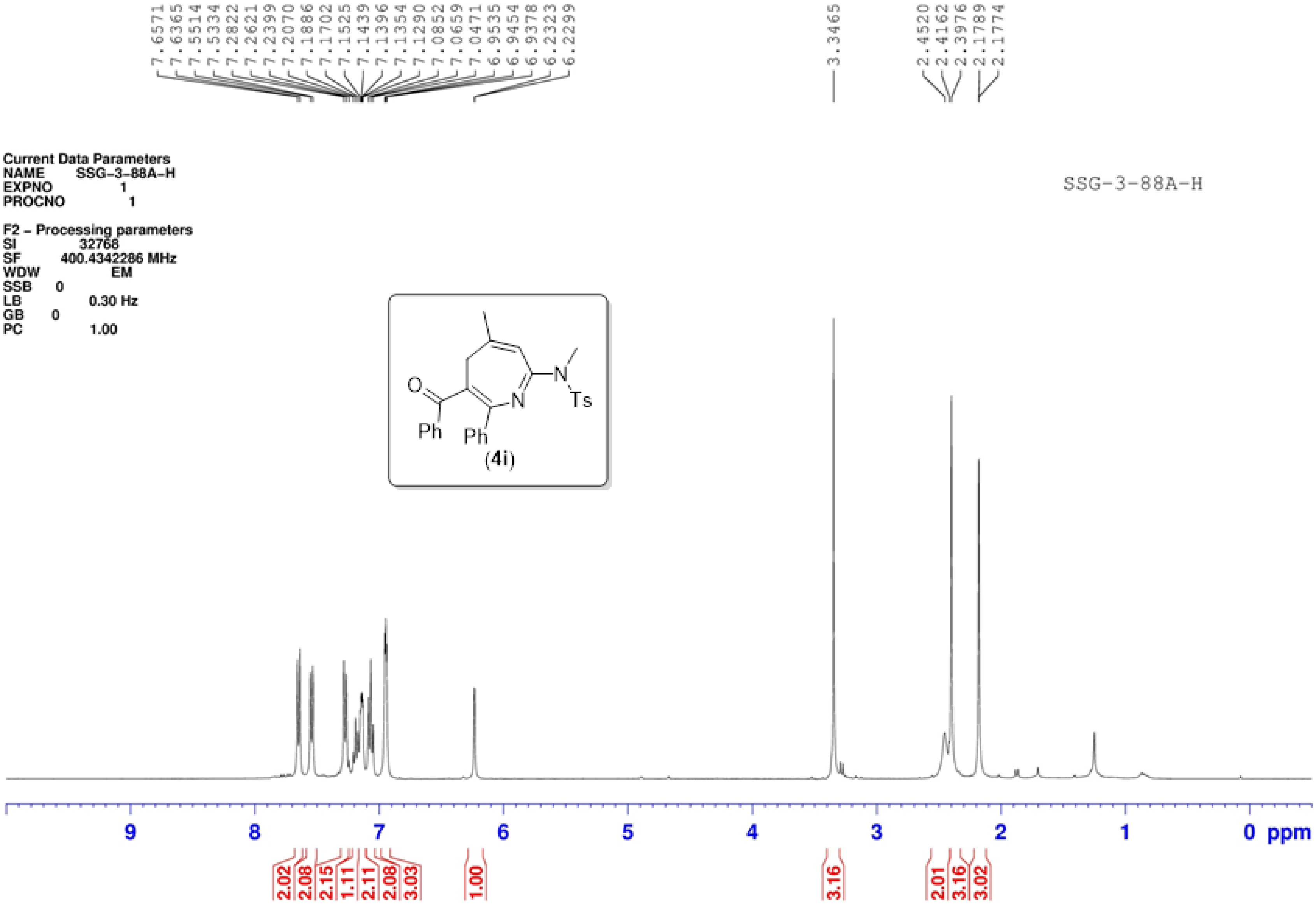

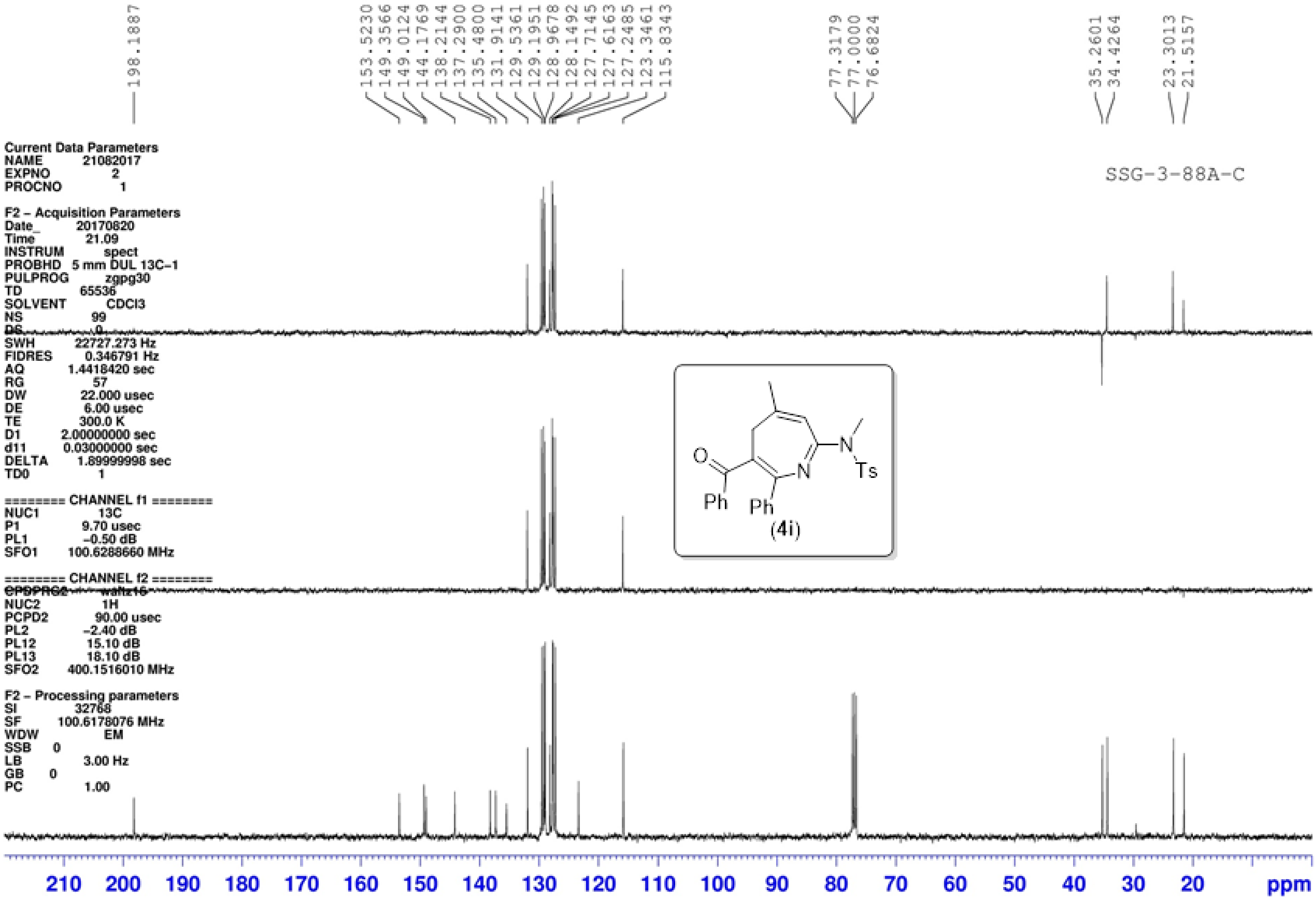
198.1887
153.5230
149.3566
149.0124
144.1769
138.2144
137.2900
135.4800
131.9141
129.5361
129.1951
128.9678
128.1492
127.7145
127.6163
127.2485
123.3461
115.8343
77.3179
77.0000
76.6824
35.2601
34.4264
23.3013
21.5157
SSG-3-88A-C
Current Data Parameters
NAME 21082017
EXPNO 2
PROCNO 1
F2 – Acquisition Parameters
Date_ 20170820
Time 21.09
INSTRUM spect
PROBHD 5 mm DUL 13C-1
PULPROG zgpg30
TD 65536
SOLVENT CDCl3
NS 99
DS 0
SWH 22727.273 Hz
FIDRES 0.346791 Hz
AQ 1.4418420 sec
RG 57
DW 22.000 usec
DE 6.00 usec
TE 300.0 K
D1 2.00000000 sec
d11 0.03000000 sec
DELTA 1.89999998 sec
TD0 1
======== CHANNEL f1 ========
NUC1 13C
P1 9.70 usec
PL1 -0.50 dB
SFO1 100.6288660 MHz
======== CHANNEL f2 ========
CPDPRG2 waltz16
NUC2 1H
PCPD2 90.00 usec
PL2 -2.40 dB
PL12 15.10 dB
PL13 18.10 dB
SFO2 400.1516010 MHz
F2 – Processing parameters
SI 32768
SF 100.6178076 MHz
WDW EM
SSB 0
LB 3.00 Hz
GB 0
PC 1.00
O
N
N
Ts
Ph
Ph
(4i)
210 200 190 180 170 160 150 140 130 120 110 100 90 80 70 60 50 40 30 20 ppm

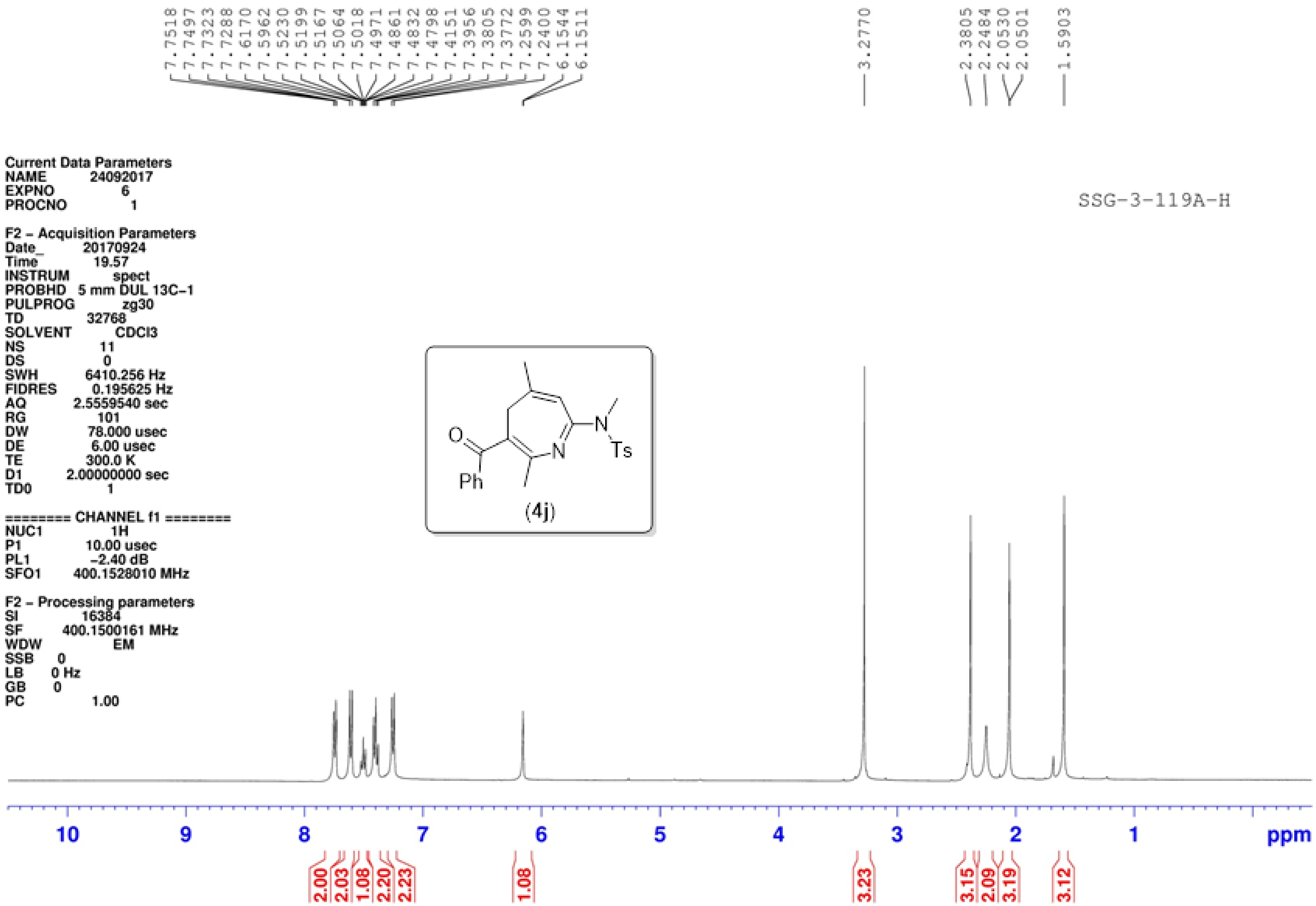
7.7518
7.7497
7.7323
7.7288
7.6170
7.5962
7.5230
7.5199
7.5167
7.5064
7.5018
7.4971
7.4861
7.4832
7.4798
7.4151
7.3956
7.3805
7.3772
7.2599
7.2400
6.1544
6.1511
3.2770
2.3805
2.2484
2.0530
2.0501
1.5903
Current Data Parameters
NAME 24092017
EXPNO 6
PROCNO 1
F2 - Acquisition Parameters
Date_ 20170924
Time 19.57
INSTRUM spect
PROBHD 5 mm DUL 13C-1
PULPROG zg30
TD 32768
SOLVENT CDCl3
NS 11
DS 0
SWH 6410.256 Hz
FIDRES 0.195625 Hz
AQ 2.5559540 sec
RG 101
DW 78.000 usec
DE 6.00 usec
TE 300.0 K
D1 2.00000000 sec
TD0 1
======== CHANNEL f1 ========
NUC1 1H
P1 10.00 usec
PL1 -2.40 dB
SFO1 400.1528010 MHz
F2 - Processing parameters
SI 16384
SF 400.1500161 MHz
WDW EM
SSB 0
LB 0 Hz
GB 0
PC 1.00
SSG-3-119A-H
O
Ph
N
N
Ts
(4j)
10
9
8
7
6
5
4
3
2
1
ppm
2.00
2.03
1.08
2.20
2.23
1.08
3.23
3.15
2.09
3.19
3.12

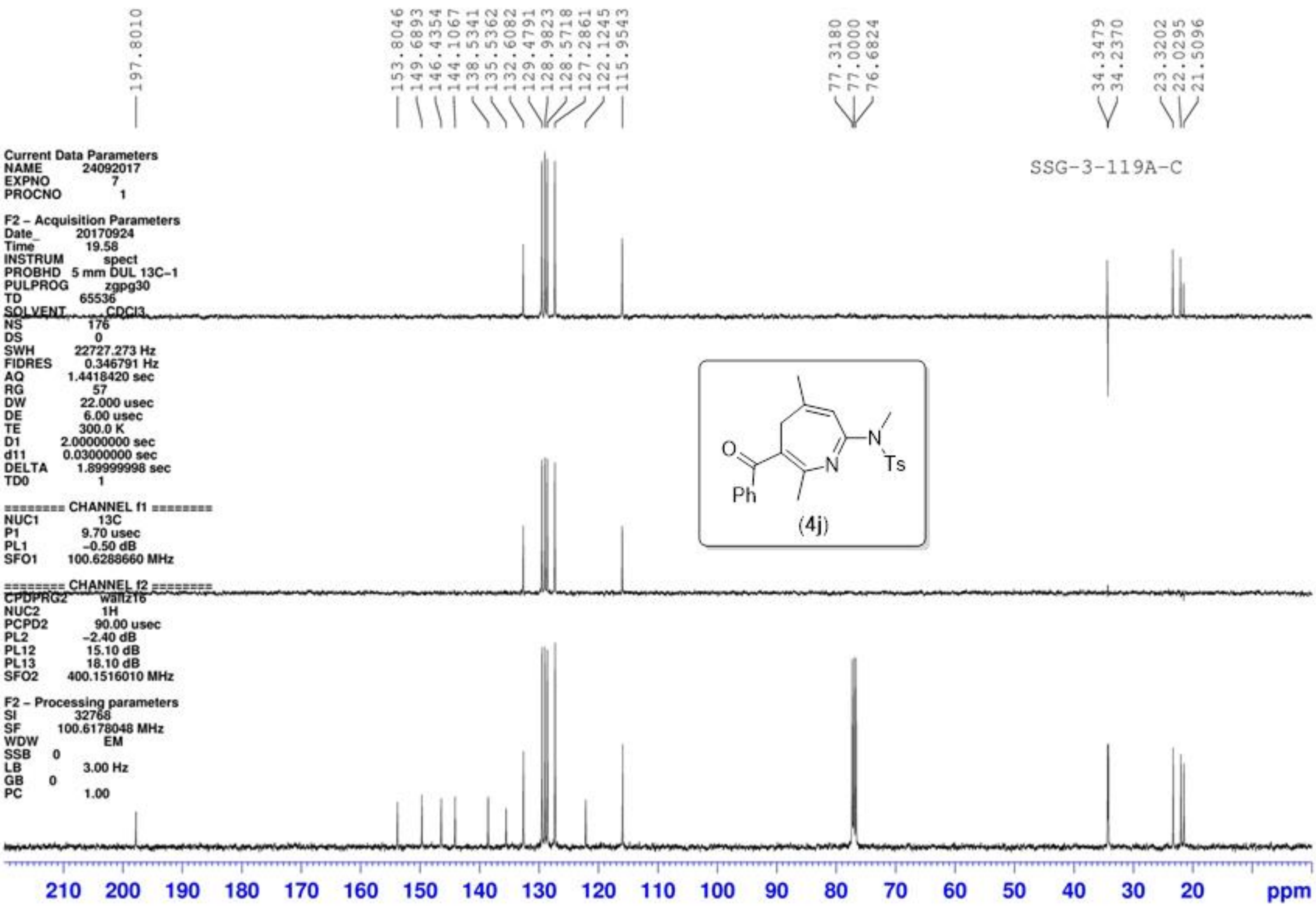

197.8010
153.8046
149.6893
146.4354
144.1067
138.5341
135.5362
132.6082
129.4791
128.9823
128.5718
127.2861
122.1245
115.9543
77.3180
77.0000
76.6824
34.3479
34.2370
23.3202
22.0295
21.5096
SSG-3-119A-C
Current Data Parameters
NAME 24092017
EXPNO 7
PROCNO 1
F2 - Acquisition Parameters
Date_ 20170924
Time 19.58
INSTRUM spect
PROBHD 5 mm DUL 13C-1
PULPROG zgpg30
TD 65536
SOLVENT CDCl3
NS 176
DS 0
SWH 22727.273 Hz
FIDRES 0.346791 Hz
AQ 1.4418420 sec
RG 57
DW 22.000 usec
DE 6.00 usec
TE 300.0 K
D1 2.00000000 sec
d11 0.03000000 sec
DELTA 1.89999998 sec
TD0 1
======== CHANNEL f1 ========
NUC1 13C
P1 9.70 usec
PL1 -0.50 dB
SFO1 100.6288660 MHz
======== CHANNEL f2 ========
CPDPRG2 waltz16
NUC2 1H
PCPD2 90.00 usec
PL2 -2.40 dB
PL12 15.10 dB
PL13 18.10 dB
SFO2 400.1516010 MHz
F2 - Processing parameters
SI 32768
SF 100.6178048 MHz
WDW EM
SSB 0
LB 3.00 Hz
GB 0
PC 1.00
O
N
N
Ts
Ph
(4j)
210 200 190 180 170 160 150 140 130 120 110 100 90 80 70 60 50 40 30 20 ppm

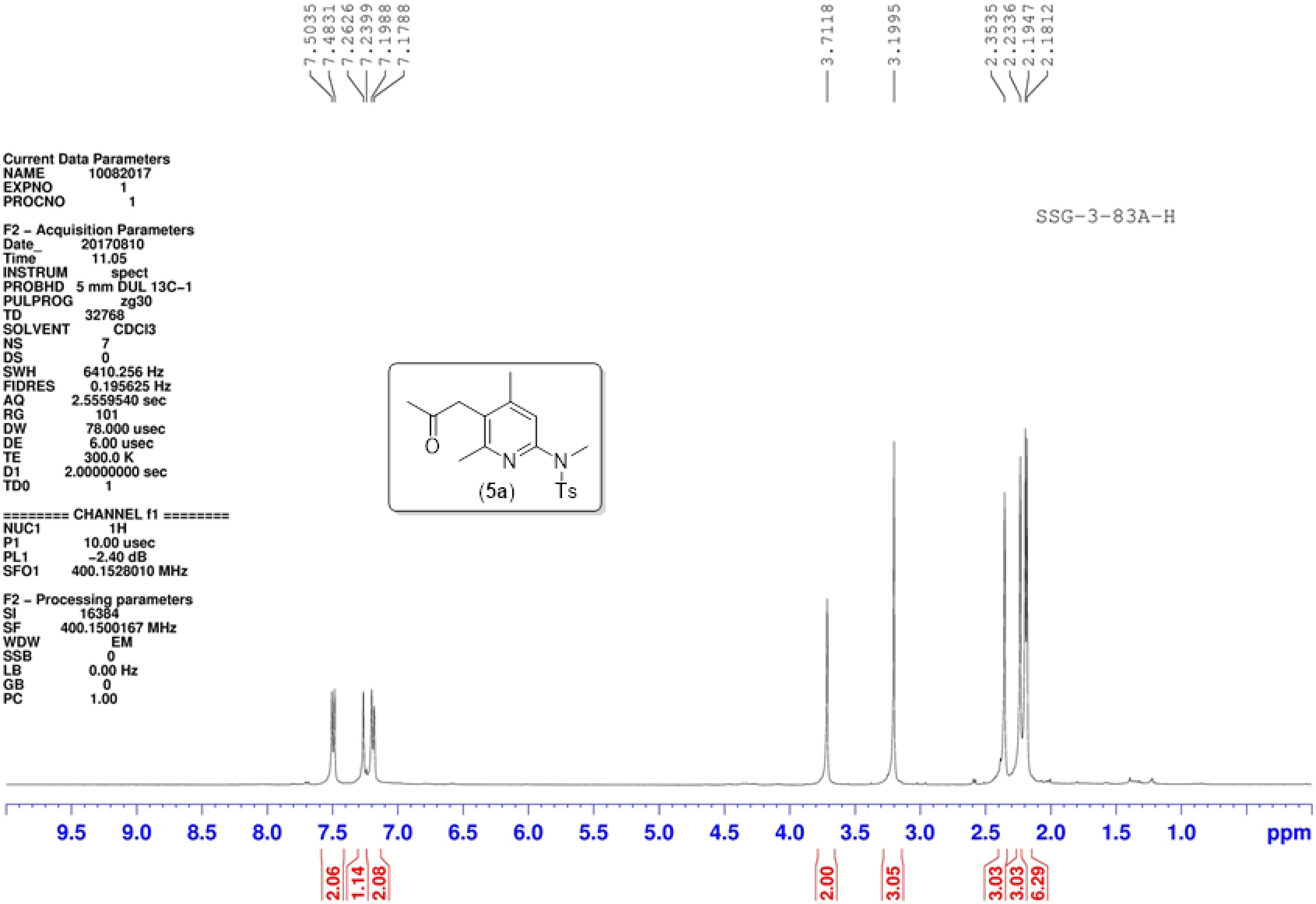
7.5035
7.4831
7.2626
7.2399
7.1988
7.1788
3.7118
3.1995
2.3535
2.2336
2.1947
2.1812
Current Data Parameters
NAME 10082017
EXPNO 1
PROCNO 1
F2 - Acquisition Parameters
Date_ 20170810
Time 11.05
INSTRUM spect
PROBHD 5 mm DUL 13C-1
PULPROG zg30
TD 32768
SOLVENT CDCl3
NS 7
DS 0
SWH 6410.256 Hz
FIDRES 0.195625 Hz
AQ 2.5559540 sec
RG 101
DW 78.000 usec
DE 6.00 usec
TE 300.0 K
D1 2.00000000 sec
TD0 1
======== CHANNEL f1 ========
NUC1 1H
P1 10.00 usec
PL1 -2.40 dB
SFO1 400.1528010 MHz
F2 - Processing parameters
SI 16384
SF 400.1500167 MHz
WDW EM
SSB 0
LB 0.00 Hz
GB 0
PC 1.00
SSG-3-83A-H
O
N
N
Ts
(5a)
9.5 9.0 8.5 8.0 7.5 7.0 6.5 6.0 5.5 5.0 4.5 4.0 3.5 3.0 2.5 2.0 1.5 1.0 ppm
2.06
1.14
2.08
2.00
3.05
3.03
3.03
6.29

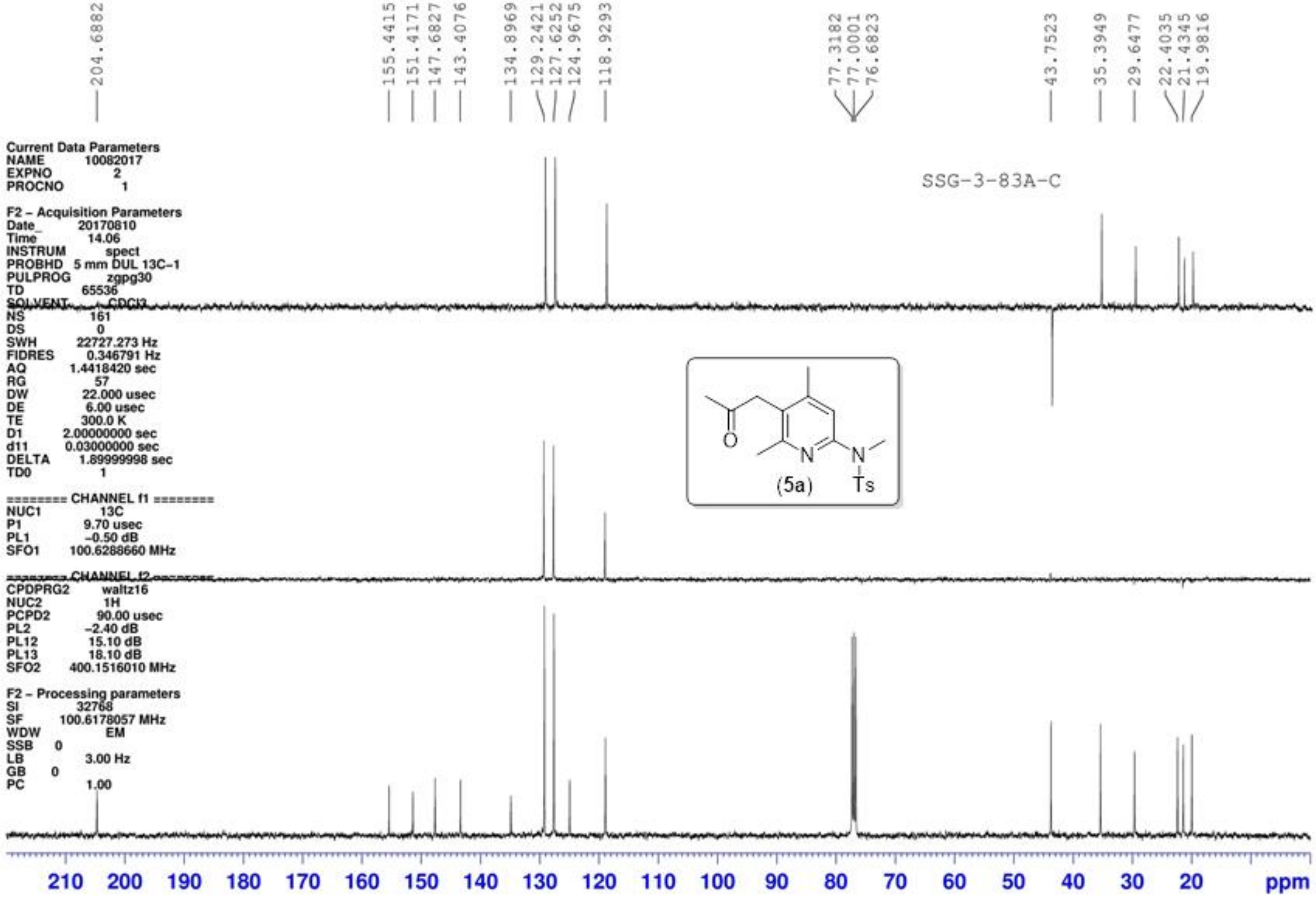
204.6882
155.4415
151.4171
147.6827
143.4076
134.8969
129.2421
127.6252
124.9675
118.9293
77.3182
77.0001
76.6823
43.7523
35.3949
29.6477
22.4035
21.4345
19.9816
SSG-3-83A-C
Current Data Parameters
NAME 10082017
EXPNO 2
PROCNO 1
F2 - Acquisition Parameters
Date_ 20170810
Time 14.06
INSTRUM spect
PROBHD 5 mm DUL 13C-1
PULPROG zgpg30
TD 65536
SOLVENT CDCl3
NS 161
DS 0
SWH 22727.273 Hz
FIDRES 0.346791 Hz
AQ 1.4418420 sec
RG 57
DW 22.000 usec
DE 6.00 usec
TE 300.0 K
D1 2.00000000 sec
d11 0.03000000 sec
DELTA 1.89999998 sec
TD0 1
======== CHANNEL f1 ========
NUC1 13C
P1 9.70 usec
PL1 -0.50 dB
SFO1 100.6288660 MHz
======== CHANNEL f2 ========
CPDPRG2 waltz16
NUC2 1H
PCPD2 90.00 usec
PL2 -2.40 dB
PL12 15.10 dB
PL13 18.10 dB
SFO2 400.1516010 MHz
F2 - Processing parameters
SI 32768
SF 100.6178057 MHz
WDW EM
SSB 0
LB 3.00 Hz
GB 0
PC 1.00
O
N
N
Ts
(5a)
210
200
190
180
170
160
150
140
130
120
110
100
90
80
70
60
50
40
30
20
ppm

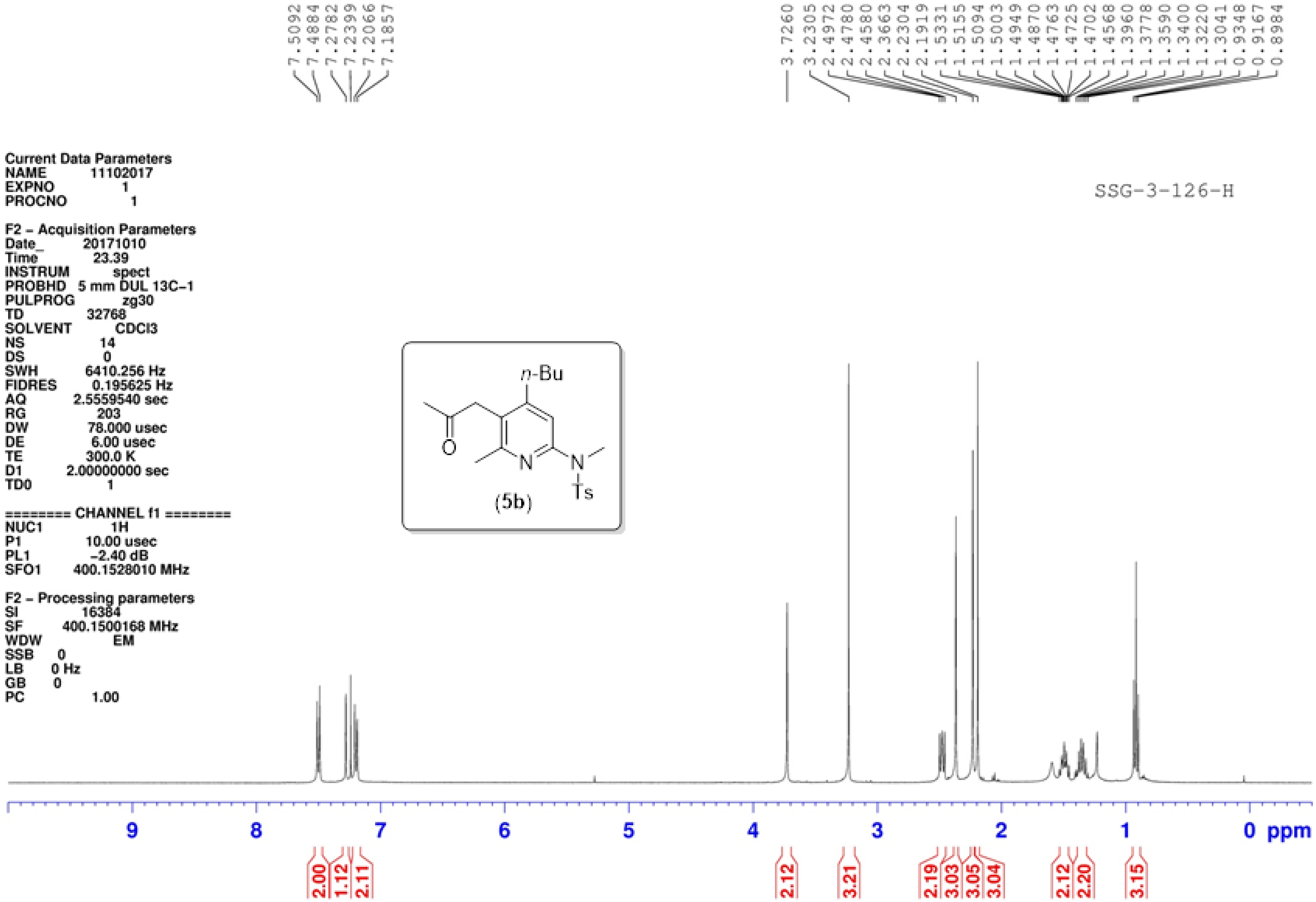
7.5092
7.4884
7.2782
7.2399
7.2066
7.1857
3.7260
3.2305
2.4972
2.4780
2.4580
2.3663
2.2304
2.1919
1.5331
1.5155
1.5094
1.5003
1.4949
1.4870
1.4763
1.4725
1.4702
1.4568
1.3960
1.3778
1.3590
1.3400
1.3220
1.3041
0.9348
0.9167
0.8984
Current Data Parameters
NAME 11102017
EXPNO 1
PROCNO 1
F2 - Acquisition Parameters
Date_ 20171010
Time 23.39
INSTRUM spect
PROBHD 5 mm DUL 13C-1
PULPROG zg30
TD 32768
SOLVENT CDCl3
NS 14
DS 0
SWH 6410.256 Hz
FIDRES 0.195625 Hz
AQ 2.5559540 sec
RG 203
DW 78.000 usec
DE 6.00 usec
TE 300.0 K
D1 2.00000000 sec
TD0 1
======== CHANNEL f1 ========
NUC1 1H
P1 10.00 usec
PL1 -2.40 dB
SFO1 400.1528010 MHz
F2 - Processing parameters
SI 16384
SF 400.1500168 MHz
WDW EM
SSB 0
LB 0 Hz
GB 0
PC 1.00
SSG-3-126-H
n-Bu
O
N
N
Ts
(5b)
9
8
7
6
5
4
3
2
1
0 ppm
2.00
1.12
2.11
2.12
3.21
2.19
3.03
3.05
3.04
2.12
2.20
3.15

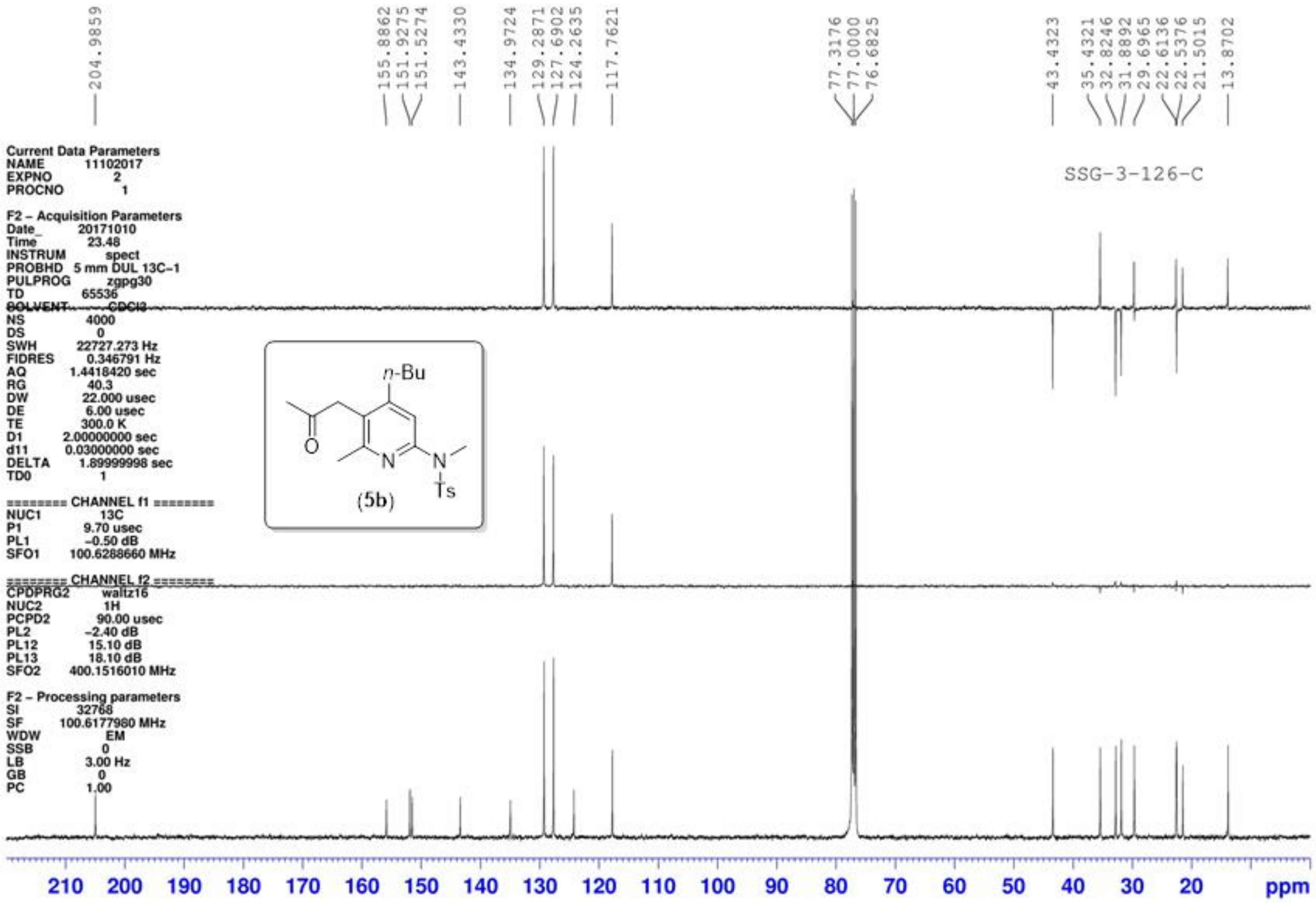
204.9859
155.8862
151.9275
151.5274
143.4330
134.9724
129.2871
127.6902
124.2635
117.7621
77.3176
77.0000
76.6825
43.4323
35.4321
32.8246
31.8892
29.6965
22.6136
22.5376
21.5015
13.8702
SSG-3-126-C
Current Data Parameters
NAME 11102017
EXPNO 2
PROCNO 1
F2 - Acquisition Parameters
Date_ 20171010
Time 23.48
INSTRUM spect
PROBHD 5 mm DUL 13C-1
PULPROG zgpg30
TD 65536
SOLVENT CDCl3
NS 4000
DS 0
SWH 22727.273 Hz
FIDRES 0.346791 Hz
AQ 1.4418420 sec
RG 40.3
DW 22.000 usec
DE 6.00 usec
TE 300.0 K
D1 2.00000000 sec
d11 0.03000000 sec
DELTA 1.89999998 sec
TD0 1
======== CHANNEL f1 ========
NUC1 13C
P1 9.70 usec
PL1 -0.50 dB
SFO1 100.6288660 MHz
======== CHANNEL f2 ========
CPDPRG2 waltz16
NUC2 1H
PCPD2 90.00 usec
PL2 -2.40 dB
PL12 15.10 dB
PL13 18.10 dB
SFO2 400.1516010 MHz
F2 - Processing parameters
SI 32768
SF 100.6177980 MHz
WDW EM
SSB 0
LB 3.00 Hz
GB 0
PC 1.00
n-Bu
O
N
N
Ts
(5b)
210
200
190
180
170
160
150
140
130
120
110
100
90
80
70
60
50
40
30
20
ppm

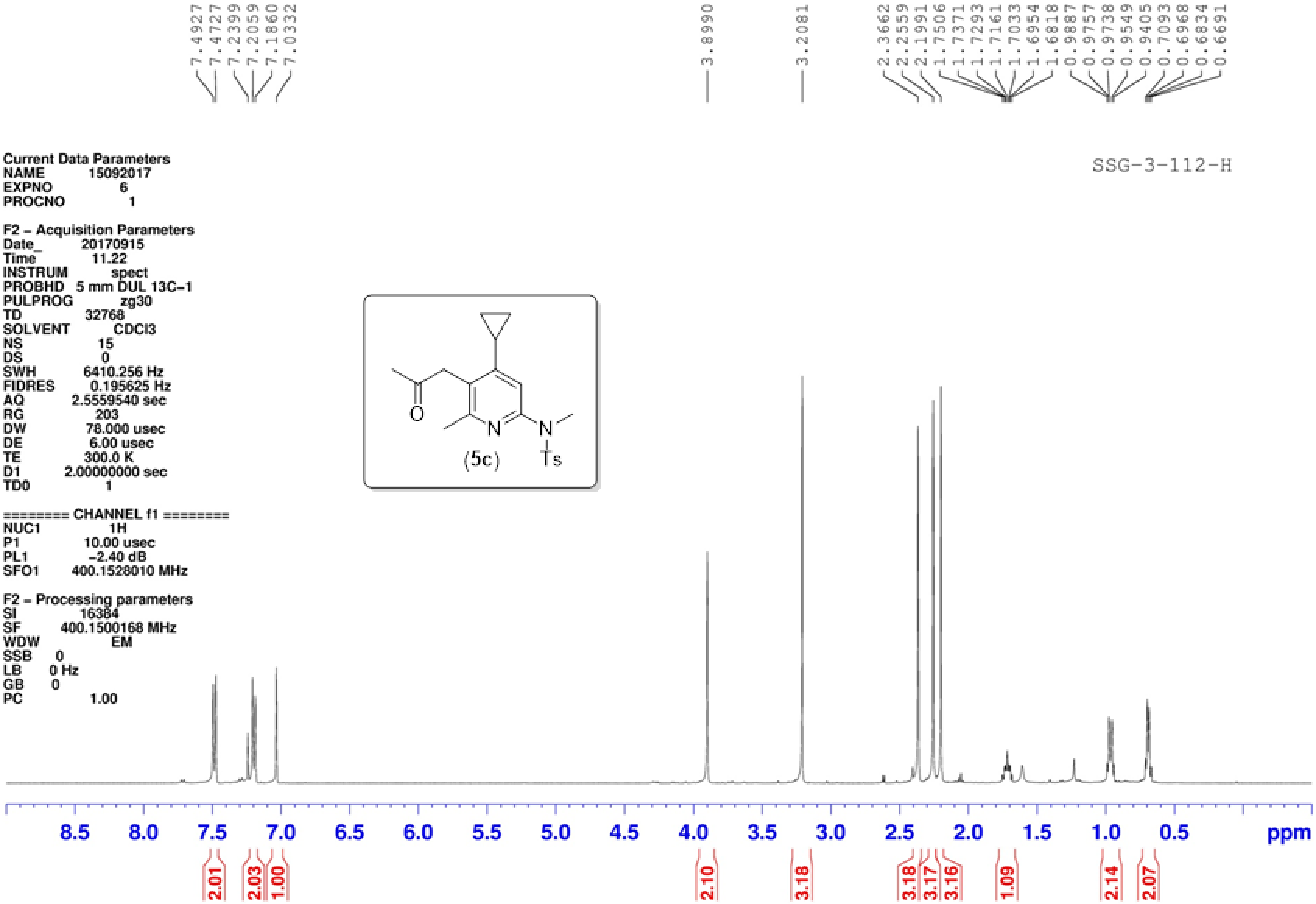
7.4927
7.4727
7.2399
7.2059
7.1860
7.0332
3.8990
3.2081
2.3662
2.2559
2.1991
1.7506
1.7371
1.7293
1.7161
1.7033
1.6954
1.6818
0.9887
0.9757
0.9738
0.9549
0.9405
0.7093
0.6968
0.6834
0.6691
SSG-3-112-H
Current Data Parameters
NAME 15092017
EXPNO 6
PROCNO 1
F2 - Acquisition Parameters
Date_ 20170915
Time 11.22
INSTRUM spect
PROBHD 5 mm DUL 13C-1
PULPROG zg30
TD 32768
SOLVENT CDCl3
NS 15
DS 0
SWH 6410.256 Hz
FIDRES 0.195625 Hz
AQ 2.5559540 sec
RG 203
DW 78.000 usec
DE 6.00 usec
TE 300.0 K
D1 2.00000000 sec
TD0 1
======== CHANNEL f1 ========
NUC1 1H
P1 10.00 usec
PL1 -2.40 dB
SFO1 400.1528010 MHz
F2 - Processing parameters
SI 16384
SF 400.1500168 MHz
WDW EM
SSB 0
LB 0 Hz
GB 0
PC 1.00
O
N
N
Ts
(5c)
8.5
8.0
7.5
7.0
6.5
6.0
5.5
5.0
4.5
4.0
3.5
3.0
2.5
2.0
1.5
1.0
0.5
ppm
2.01
2.03
1.00
2.10
3.18
3.18
3.17
3.16
1.09
2.14
2.07

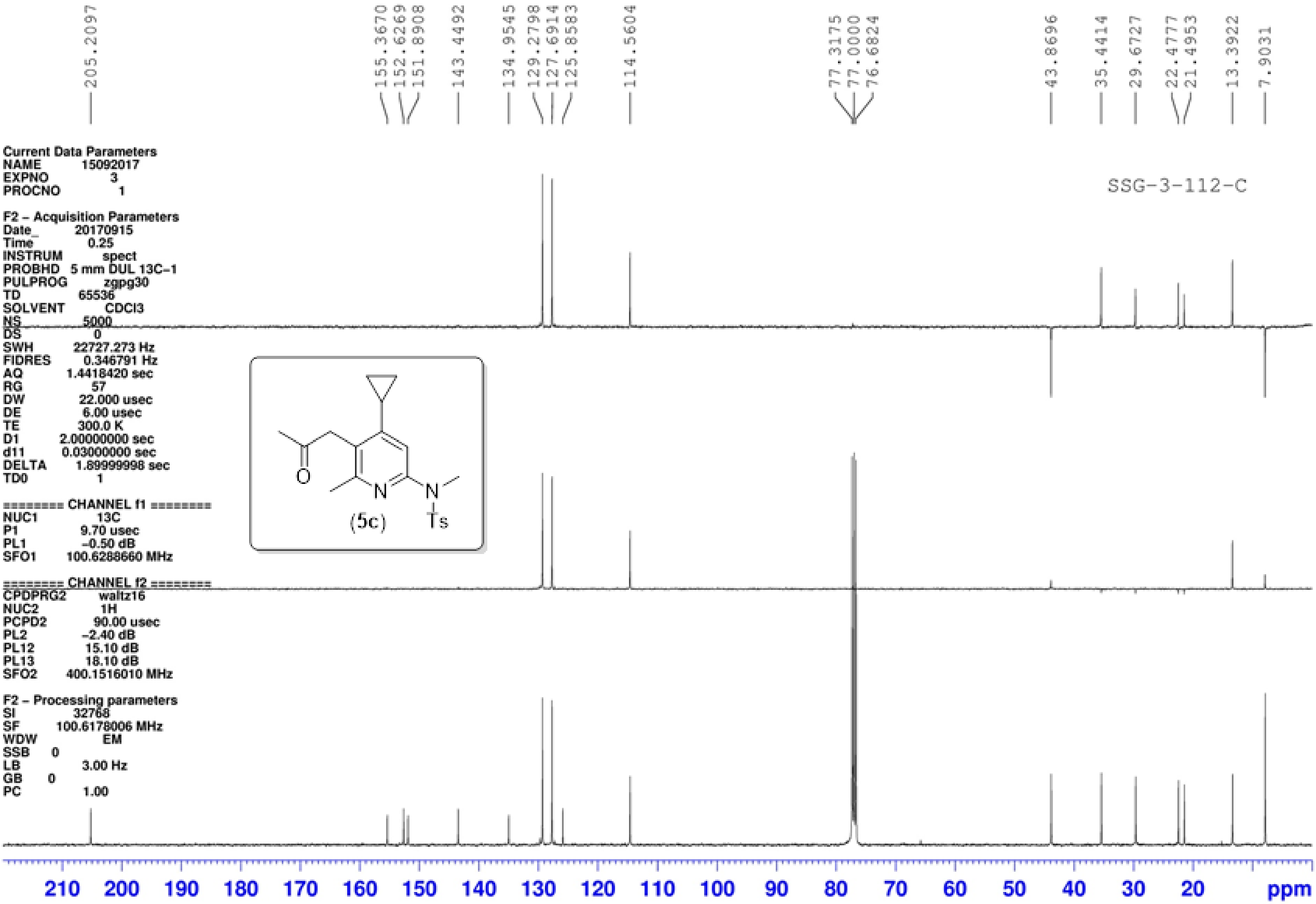
205.2097
155.3670
152.6269
151.8908
143.4492
134.9545
129.2798
127.6914
125.8583
114.5604
77.3175
77.0000
76.6824
43.8696
35.4414
29.6727
22.4777
21.4953
13.3922
7.9031
SSG-3-112-C
Current Data Parameters
NAME 15092017
EXPNO 3
PROCNO 1
F2 – Acquisition Parameters
Date_ 20170915
Time 0.25
INSTRUM spect
PROBHD 5 mm DUL 13C-1
PULPROG zgpg30
TD 65536
SOLVENT CDCl3
NS 5000
DS 0
SWH 22727.273 Hz
FIDRES 0.346791 Hz
AQ 1.4418420 sec
RG 57
DW 22.000 usec
DE 6.00 usec
TE 300.0 K
D1 2.00000000 sec
d11 0.03000000 sec
DELTA 1.89999998 sec
TD0 1
======== CHANNEL f1 ========
NUC1 13C
P1 9.70 usec
PL1 -0.50 dB
SFO1 100.6288660 MHz
======== CHANNEL f2 ========
CPDPRG2 waltz16
NUC2 1H
PCPD2 90.00 usec
PL2 -2.40 dB
PL12 15.10 dB
PL13 18.10 dB
SFO2 400.1516010 MHz
F2 – Processing parameters
SI 32768
SF 100.6178006 MHz
WDW EM
SSB 0
LB 3.00 Hz
GB 0
PC 1.00
O
N
N
Ts
(5c)
210 200 190 180 170 160 150 140 130 120 110 100 90 80 70 60 50 40 30 20 ppm

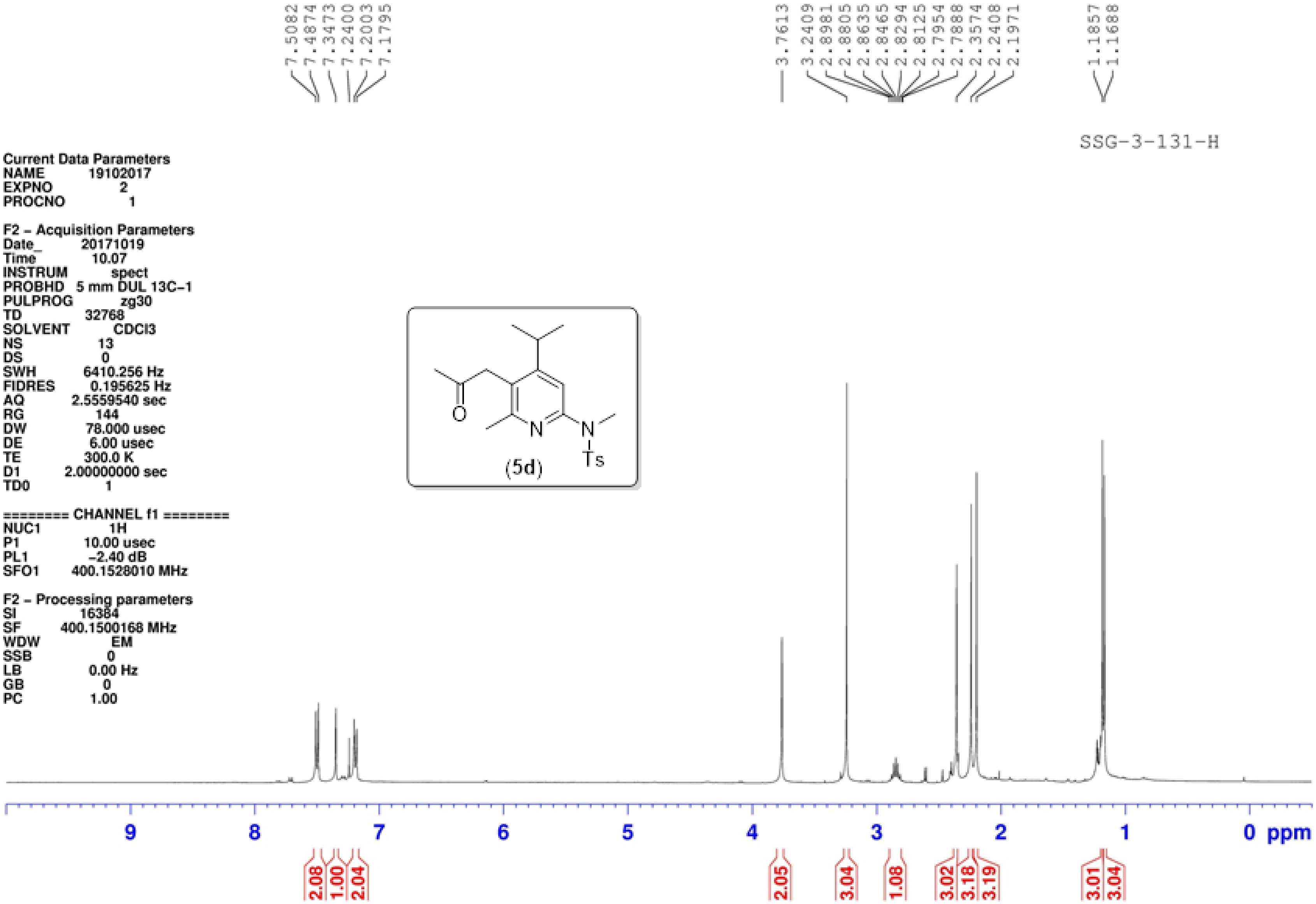
7.5082
7.4874
7.3473
7.2400
7.2003
7.1795
3.7613
3.2409
2.8981
2.8805
2.8635
2.8465
2.8294
2.8125
2.7954
2.7888
2.3574
2.2408
2.1971
1.1857
1.1688
SSG-3-131-H
Current Data Parameters
NAME 19102017
EXPNO 2
PROCNO 1
F2 - Acquisition Parameters
Date_ 20171019
Time 10.07
INSTRUM spect
PROBHD 5 mm DUL 13C-1
PULPROG zg30
TD 32768
SOLVENT CDCl3
NS 13
DS 0
SWH 6410.256 Hz
FIDRES 0.195625 Hz
AQ 2.5559540 sec
RG 144
DW 78.000 usec
DE 6.00 usec
TE 300.0 K
D1 2.00000000 sec
TD0 1
======== CHANNEL f1 ========
NUC1 1H
P1 10.00 usec
PL1 -2.40 dB
SFO1 400.1528010 MHz
F2 - Processing parameters
SI 16384
SF 400.1500168 MHz
WDW EM
SSB 0
LB 0.00 Hz
GB 0
PC 1.00
O
N
N
Ts
(5d)
9
8
7
6
5
4
3
2
1
0 ppm
2.08
1.00
2.04
2.05
3.04
1.08
3.02
3.18
3.19
3.01
3.04

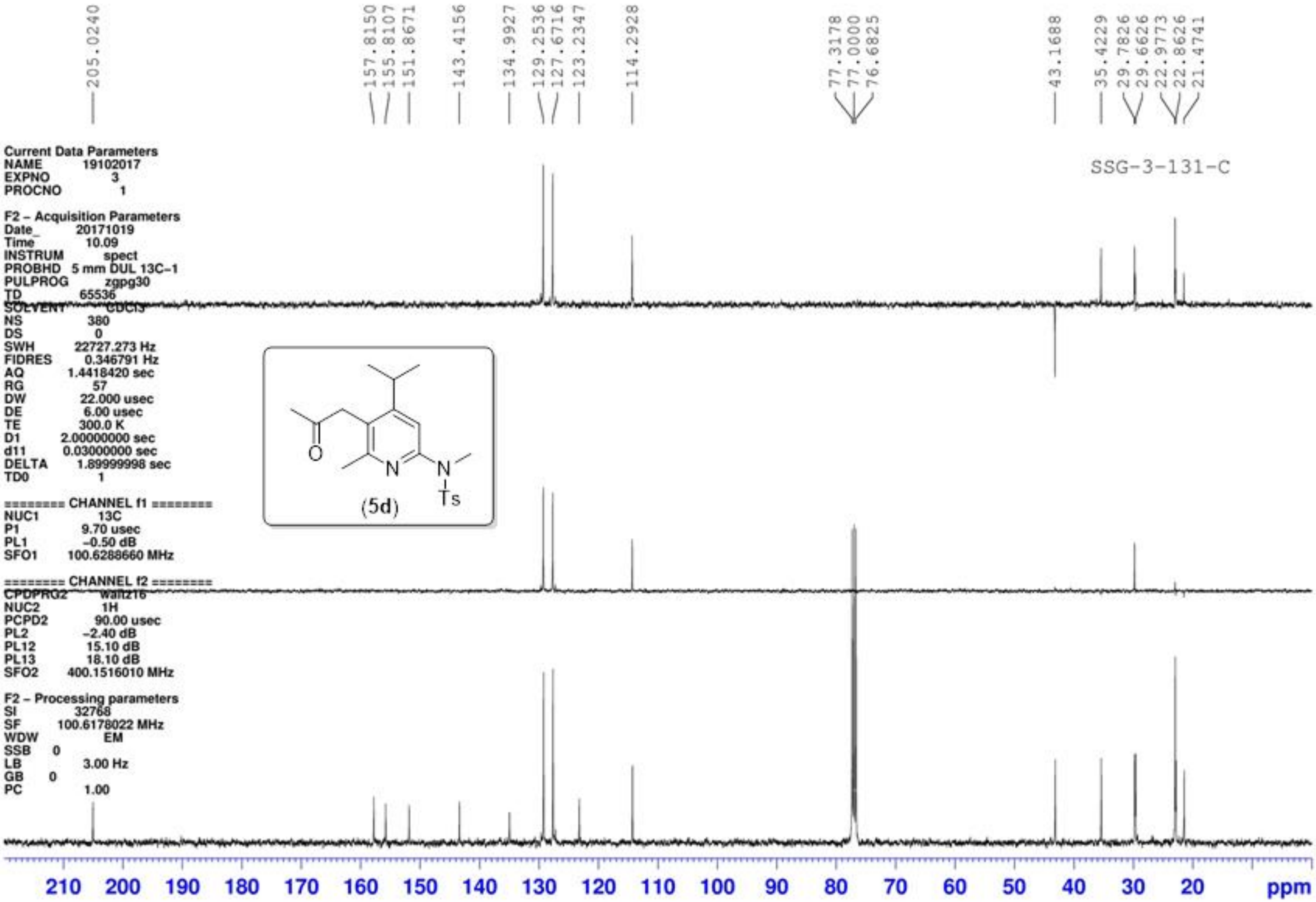
205.0240
157.8150
155.8107
151.8671
143.4156
134.9927
129.2536
127.6716
123.2347
114.2928
77.3178
77.0000
76.6825
43.1688
35.4229
29.7826
29.6626
22.9773
22.8626
21.4741
SSG-3-131-C
Current Data Parameters
NAME 19102017
EXPNO 3
PROCNO 1
F2 - Acquisition Parameters
Date_ 20171019
Time 10.09
INSTRUM spect
PROBHD 5 mm DUL 13C-1
PULPROG zgpg30
TD 65536
SOLVENT CDCl3
NS 380
DS 0
SWH 22727.273 Hz
FIDRES 0.346791 Hz
AQ 1.4418420 sec
RG 57
DW 22.000 usec
DE 6.00 usec
TE 300.0 K
D1 2.00000000 sec
d11 0.03000000 sec
DELTA 1.89999998 sec
TD0 1
======== CHANNEL f1 ========
NUC1 13C
P1 9.70 usec
PL1 -0.50 dB
SFO1 100.6288660 MHz
======== CHANNEL f2 ========
CPDPRG2 waltz16
NUC2 1H
PCPD2 90.00 usec
PL2 -2.40 dB
PL12 15.10 dB
PL13 18.10 dB
SFO2 400.1516010 MHz
F2 - Processing parameters
SI 32768
SF 100.6178022 MHz
WDW EM
SSB 0
LB 3.00 Hz
GB 0
PC 1.00
(5d)
210 200 190 180 170 160 150 140 130 120 110 100 90 80 70 60 50 40 30 20 ppm

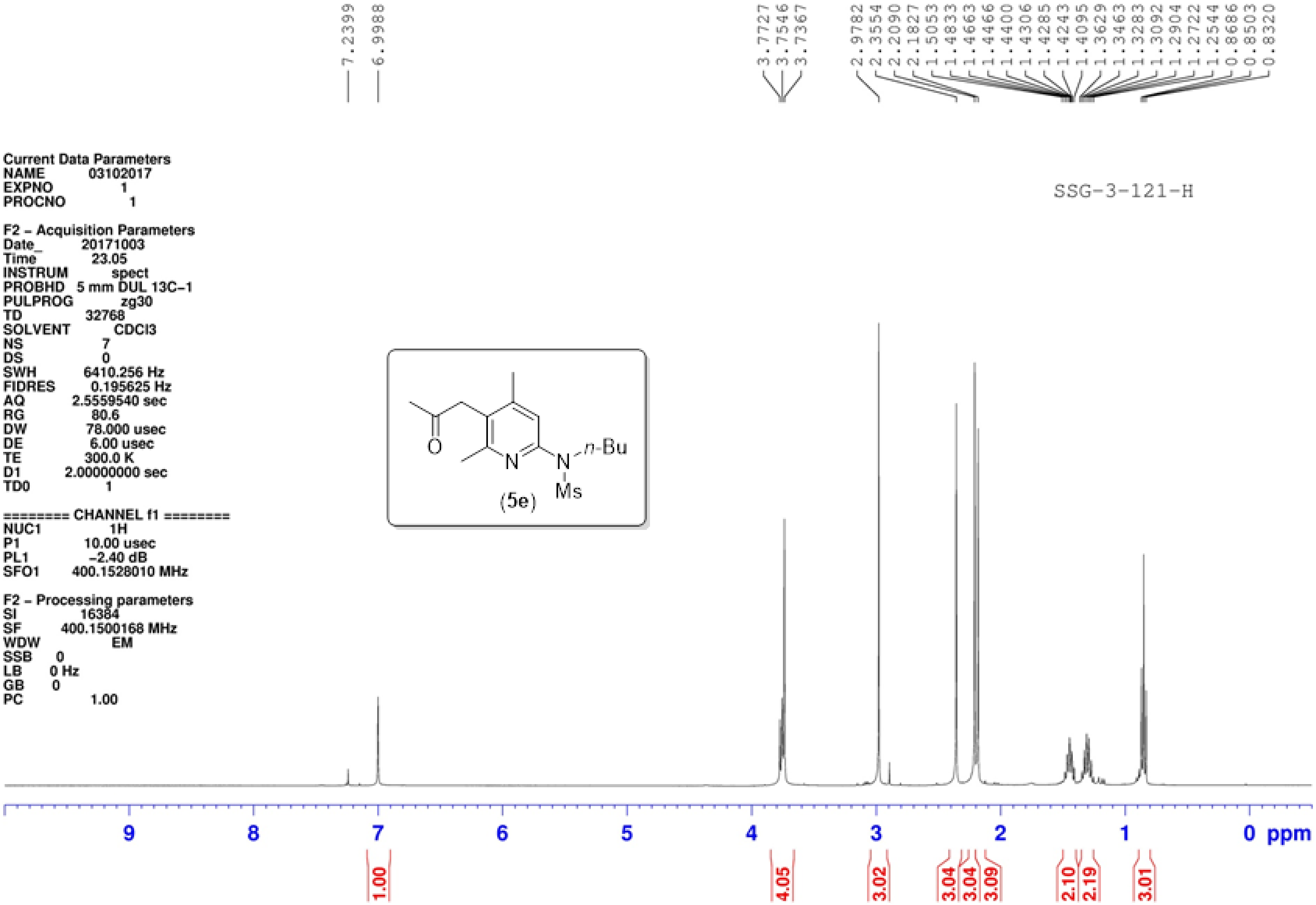
Current Data Parameters
NAME 03102017
EXPNO 1
PROCNO 1
F2 - Acquisition Parameters
Date_ 20171003
Time 23.05
INSTRUM spect
PROBHD 5 mm DUL 13C-1
PULPROG zg30
TD 32768
SOLVENT CDCl3
NS 7
DS 0
SWH 6410.256 Hz
FIDRES 0.195625 Hz
AQ 2.5559540 sec
RG 80.6
DW 78.000 usec
DE 6.00 usec
TE 300.0 K
D1 2.00000000 sec
TD0 1
======== CHANNEL f1 ========
NUC1 1H
P1 10.00 usec
PL1 -2.40 dB
SFO1 400.1528010 MHz
F2 - Processing parameters
SI 16384
SF 400.1500168 MHz
WDW EM
SSB 0
LB 0 Hz
GB 0
PC 1.00
SSG-3-121-H
7.2399
6.9988
3.7727
3.7546
3.7367
2.9782
2.3554
2.2090
2.1827
1.5053
1.4833
1.4663
1.4466
1.4400
1.4306
1.4285
1.4243
1.4095
1.3629
1.3463
1.3283
1.3092
1.2904
1.2722
1.2544
0.8686
0.8503
0.8320
O
N
N
n-Bu
Ms
(5e)
9
8
7
6
5
4
3
2
1
0 ppm
1.00
4.05
3.02
3.04
3.04
3.09
2.10
2.19
3.01

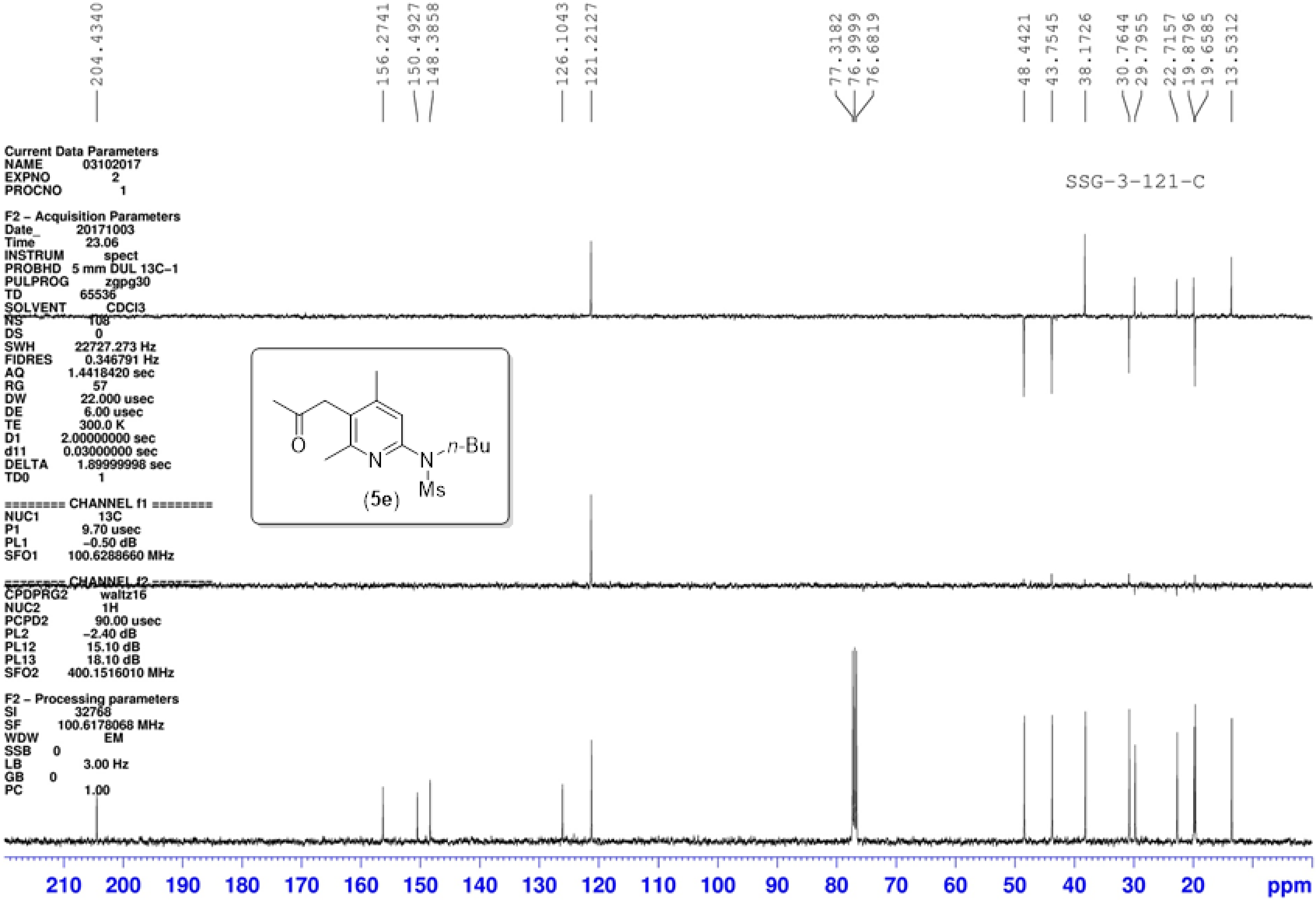
204.4340
156.2741
150.4927
148.3858
126.1043
121.2127
77.3182
76.9999
76.6819
48.4421
43.7545
38.1726
30.7644
29.7955
22.7157
19.8796
19.6585
13.5312
Current Data Parameters
NAME 03102017
EXPNO 2
PROCNO 1
F2 – Acquisition Parameters
Date_ 20171003
Time 23.06
INSTRUM spect
PROBHD 5 mm DUL 13C-1
PULPROG zgpg30
TD 65536
SOLVENT CDCl3
NS 108
DS 0
SWH 22727.273 Hz
FIDRES 0.346791 Hz
AQ 1.4418420 sec
RG 57
DW 22.000 usec
DE 6.00 usec
TE 300.0 K
D1 2.00000000 sec
d11 0.03000000 sec
DELTA 1.89999998 sec
TD0 1
======== CHANNEL f1 ========
NUC1 13C
P1 9.70 usec
PL1 -0.50 dB
SFO1 100.6288660 MHz
======== CHANNEL f2 ========
CPDPRG2 waltz16
NUC2 1H
PCPD2 90.00 usec
PL2 -2.40 dB
PL12 15.10 dB
PL13 18.10 dB
SFO2 400.1516010 MHz
F2 – Processing parameters
SI 32768
SF 100.6178068 MHz
WDW EM
SSB 0
LB 3.00 Hz
GB 0
PC 1.00
SSG-3-121-C
O
N
N
n-Bu
Ms
(5e)
210 200 190 180 170 160 150 140 130 120 110 100 90 80 70 60 50 40 30 20 ppm

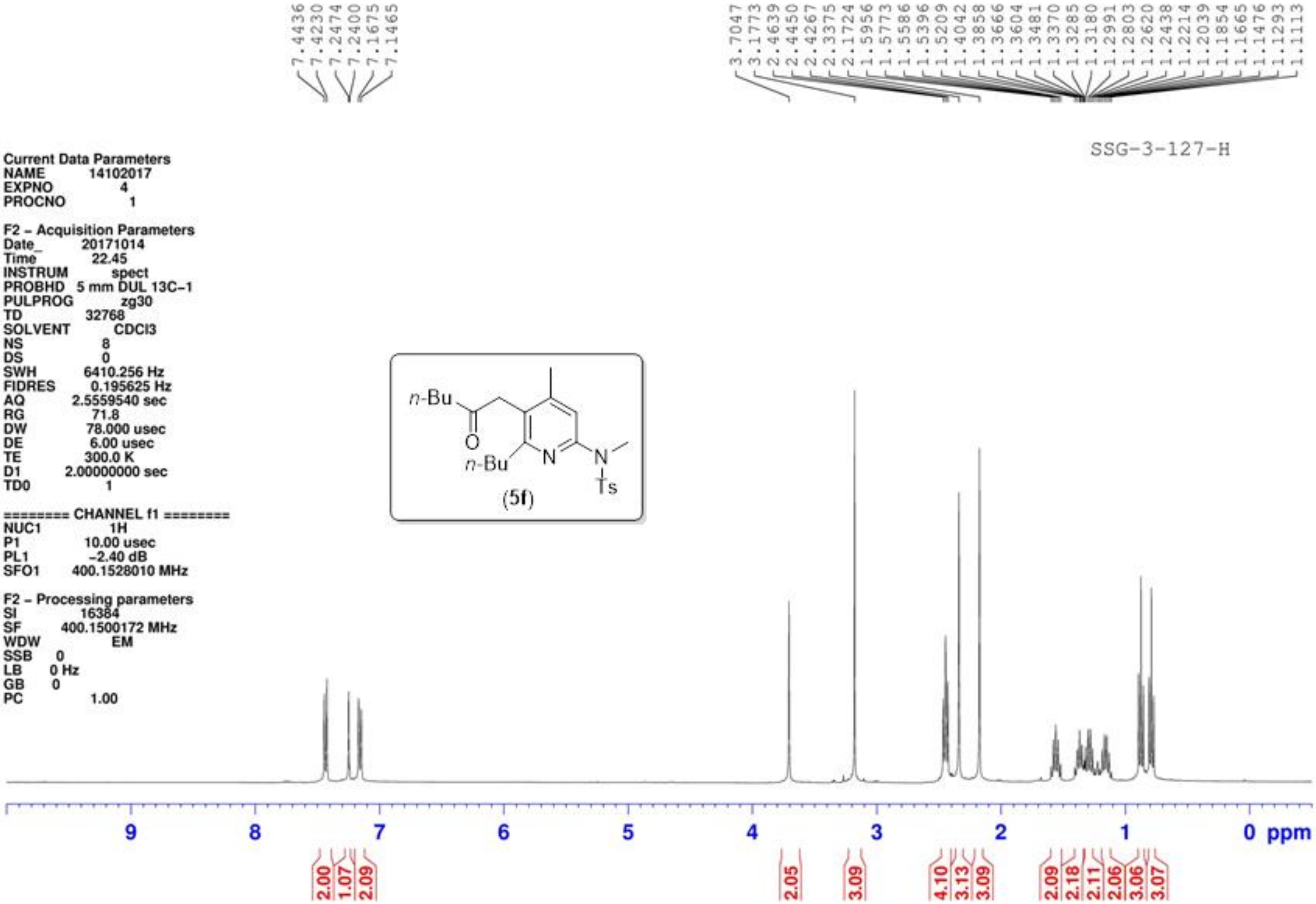

7.4436
7.4230
7.2474
7.2400
7.1675
7.1465
3.7047
3.1773
2.4639
2.4450
2.4267
2.3375
2.1724
1.5956
1.5773
1.5586
1.5396
1.5209
1.4042
1.3858
1.3666
1.3604
1.3481
1.3370
1.3285
1.3180
1.2991
1.2803
1.2620
1.2438
1.2214
1.2039
1.1854
1.1665
1.1476
1.1293
1.1113
SSG-3-127-H
Current Data Parameters
NAME 14102017
EXPNO 4
PROCNO 1
F2 - Acquisition Parameters
Date_ 20171014
Time 22.45
INSTRUM spect
PROBHD 5 mm DUL 13C-1
PULPROG zg30
TD 32768
SOLVENT CDCl3
NS 8
DS 0
SWH 6410.256 Hz
FIDRES 0.195625 Hz
AQ 2.5559540 sec
RG 71.8
DW 78.000 usec
DE 6.00 usec
TE 300.0 K
D1 2.00000000 sec
TD0 1
======== CHANNEL f1 ========
NUC1 1H
P1 10.00 usec
PL1 -2.40 dB
SFO1 400.1528010 MHz
F2 - Processing parameters
SI 16384
SF 400.1500172 MHz
WDW EM
SSB 0
LB 0 Hz
GB 0
PC 1.00
n-Bu
O
n-Bu
N
N
Ts
(5f)
9
8
7
6
5
4
3
2
1
0 ppm
2.00
1.07
2.09
2.05
3.09
4.10
3.13
3.09
2.09
2.18
2.11
2.06
3.06
3.07

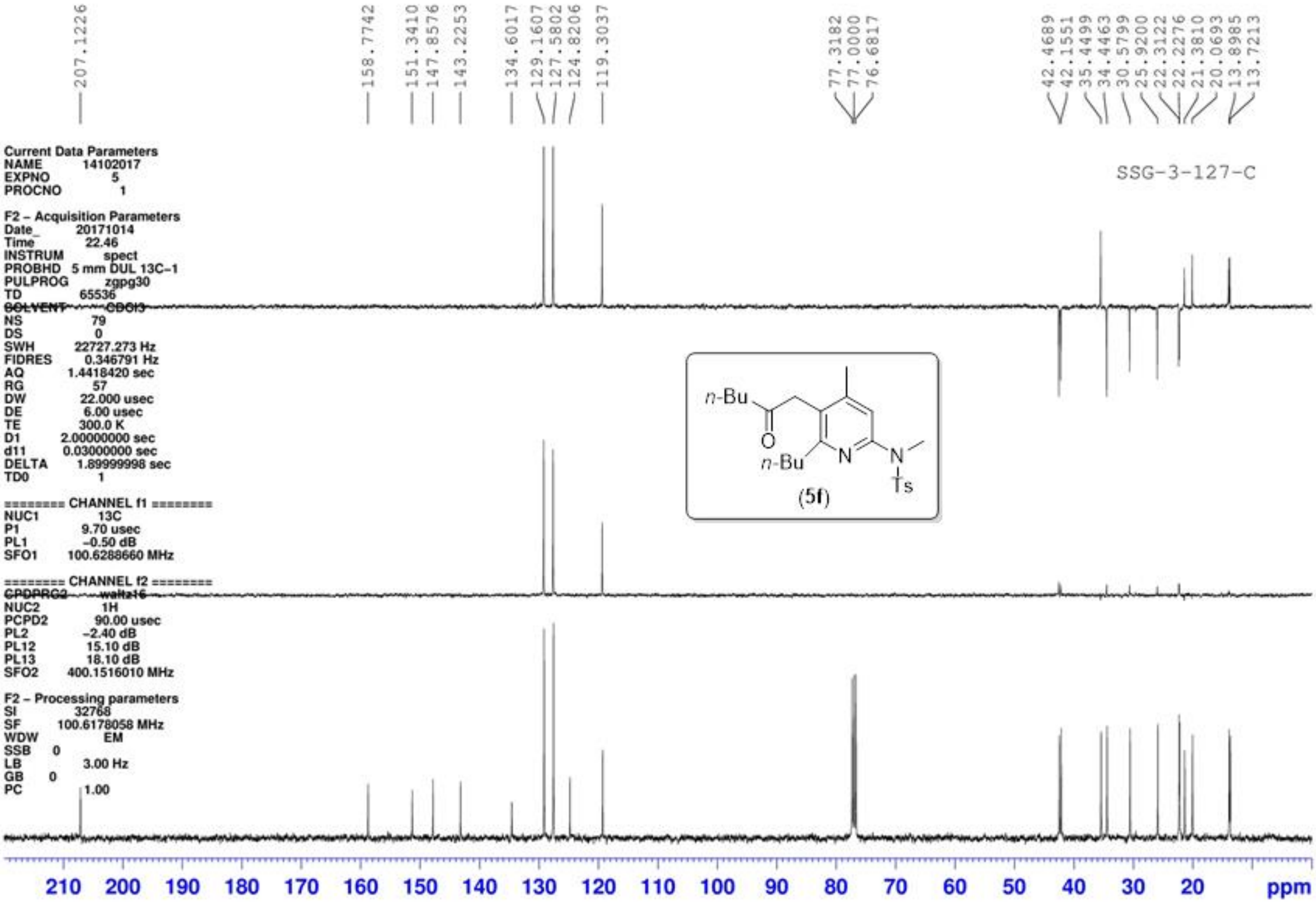

207.1226
158.7742
151.3410
147.8576
143.2253
134.6017
129.1607
127.5802
124.8206
119.3037
77.3182
77.0000
76.6817
42.4689
42.1551
35.4499
34.4463
30.5799
25.9200
22.3122
22.2276
21.3810
20.0693
13.8985
13.7213
SSG-3-127-C
Current Data Parameters
NAME 14102017
EXPNO 5
PROCNO 1
F2 - Acquisition Parameters
Date_ 20171014
Time 22.46
INSTRUM spect
PROBHD 5 mm DUL 13C-1
PULPROG zgpg30
TD 65536
SOLVENT CDCl3
NS 79
DS 0
SWH 22727.273 Hz
FIDRES 0.346791 Hz
AQ 1.4418420 sec
RG 57
DW 22.000 usec
DE 6.00 usec
TE 300.0 K
D1 2.00000000 sec
d11 0.03000000 sec
DELTA 1.89999998 sec
TD0 1
======== CHANNEL f1 ========
NUC1 13C
P1 9.70 usec
PL1 -0.50 dB
SFO1 100.6288660 MHz
======== CHANNEL f2 ========
CPDPRG2 waltz16
NUC2 1H
PCPD2 90.00 usec
PL2 -2.40 dB
PL12 15.10 dB
PL13 18.10 dB
SFO2 400.1516010 MHz
F2 - Processing parameters
SI 32768
SF 100.6178058 MHz
WDW EM
SSB 0
LB 3.00 Hz
GB 0
PC 1.00
n-Bu
O
n-Bu
N
N
Ts
(5f)
210 200 190 180 170 160 150 140 130 120 110 100 90 80 70 60 50 40 30 20 ppm

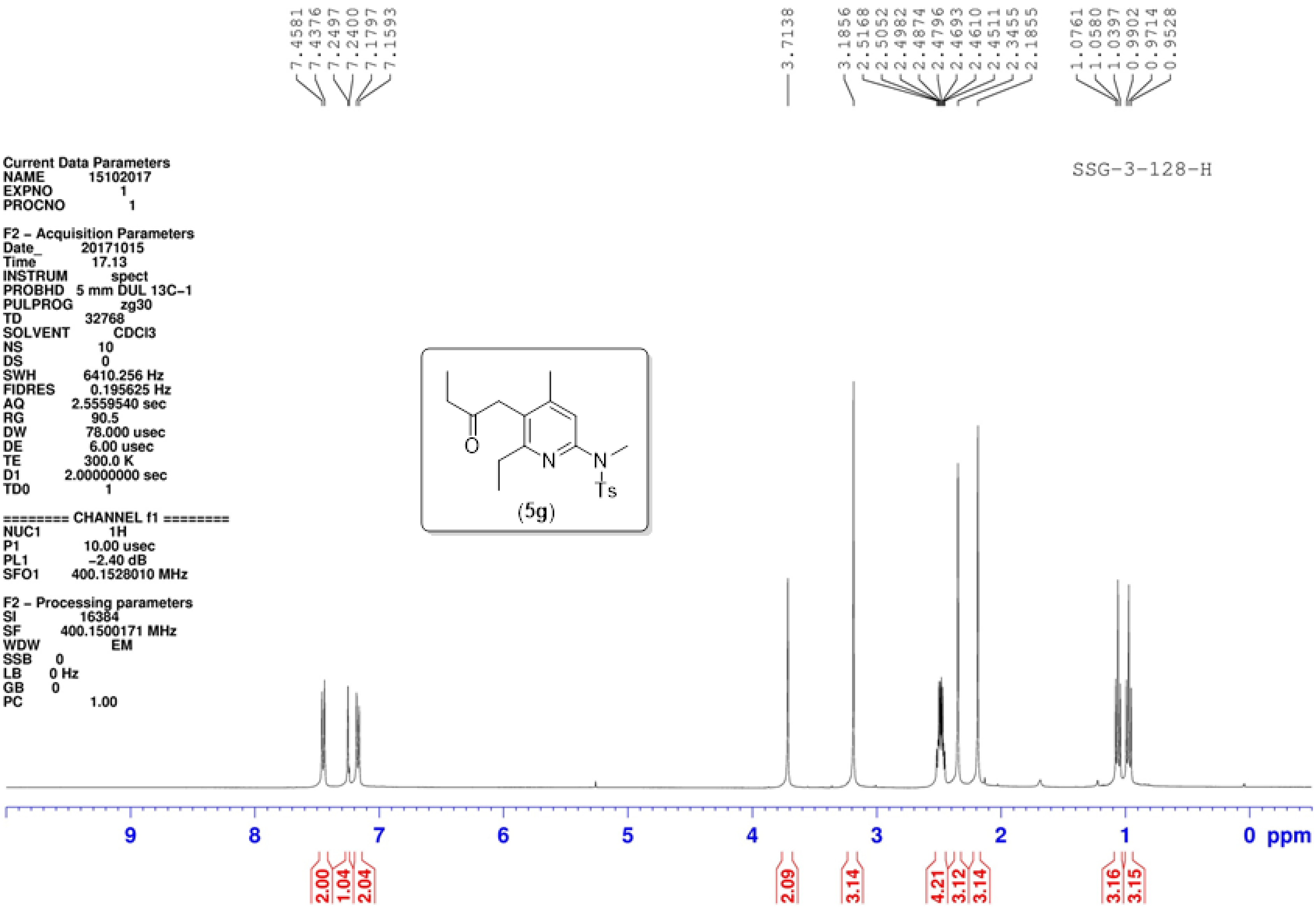
7.4581
7.4376
7.2497
7.2400
7.1797
7.1593
3.7138
3.1856
2.5168
2.5052
2.4982
2.4874
2.4796
2.4693
2.4610
2.4511
2.3455
2.1855
1.0761
1.0580
1.0397
0.9902
0.9714
0.9528
SSG-3-128-H
Current Data Parameters
NAME 15102017
EXPNO 1
PROCNO 1
F2 - Acquisition Parameters
Date_ 20171015
Time 17.13
INSTRUM spect
PROBHD 5 mm DUL 13C-1
PULPROG zg30
TD 32768
SOLVENT CDCl3
NS 10
DS 0
SWH 6410.256 Hz
FIDRES 0.195625 Hz
AQ 2.5559540 sec
RG 90.5
DW 78.000 usec
DE 6.00 usec
TE 300.0 K
D1 2.00000000 sec
TD0 1
======== CHANNEL f1 ========
NUC1 1H
P1 10.00 usec
PL1 -2.40 dB
SFO1 400.1528010 MHz
F2 - Processing parameters
SI 16384
SF 400.1500171 MHz
WDW EM
SSB 0
LB 0 Hz
GB 0
PC 1.00
O
N
N
Ts
(5g)
9
8
7
6
5
4
3
2
1
0 ppm
2.00
1.04
2.04
2.09
3.14
4.21
3.12
3.14
3.16
3.15

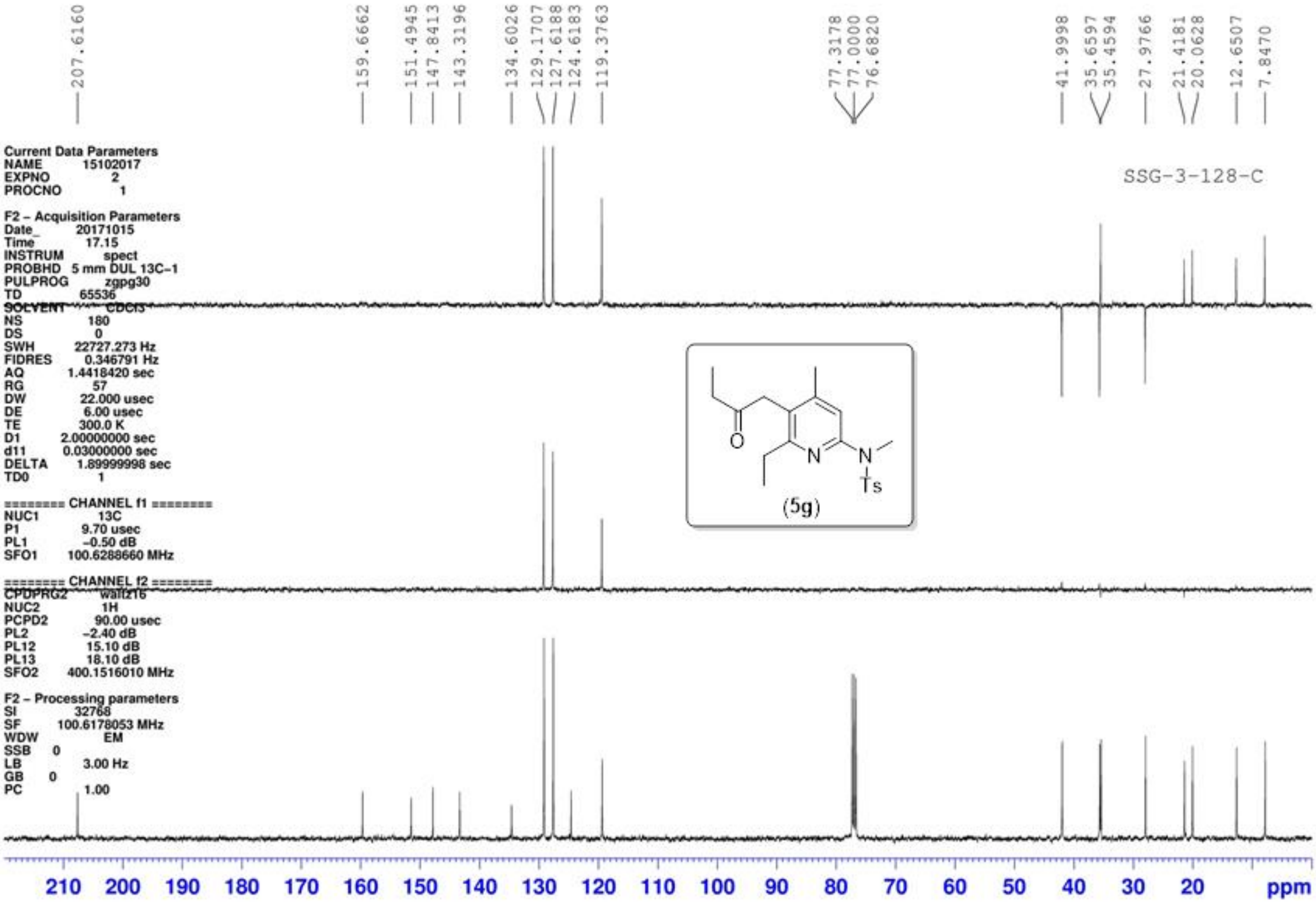

207.6160
159.6662
151.4945
147.8413
143.3196
134.6026
129.1707
127.6188
124.6183
119.3763
77.3178
77.0000
76.6820
41.9998
35.6597
35.4594
27.9766
21.4181
20.0628
12.6507
7.8470
SSG-3-128-C
Current Data Parameters
NAME 15102017
EXPNO 2
PROCNO 1
F2 - Acquisition Parameters
Date_ 20171015
Time 17.15
INSTRUM spect
PROBHD 5 mm DUL 13C-1
PULPROG zgpg30
TD 65536
SOLVENT CDCl3
NS 180
DS 0
SWH 22727.273 Hz
FIDRES 0.346791 Hz
AQ 1.4418420 sec
RG 57
DW 22.000 usec
DE 6.00 usec
TE 300.0 K
D1 2.00000000 sec
d11 0.03000000 sec
DELTA 1.89999998 sec
TD0 1
======== CHANNEL f1 ========
NUC1 13C
P1 9.70 usec
PL1 -0.50 dB
SFO1 100.6288660 MHz
======== CHANNEL f2 ========
CPDPRG2 waltz16
NUC2 1H
PCPD2 90.00 usec
PL2 -2.40 dB
PL12 15.10 dB
PL13 18.10 dB
SFO2 400.1516010 MHz
F2 - Processing parameters
SI 32768
SF 100.6178053 MHz
WDW EM
SSB 0
LB 3.00 Hz
GB 0
PC 1.00
O
N
N
Ts
(5g)
210 200 190 180 170 160 150 140 130 120 110 100 90 80 70 60 50 40 30 20 ppm

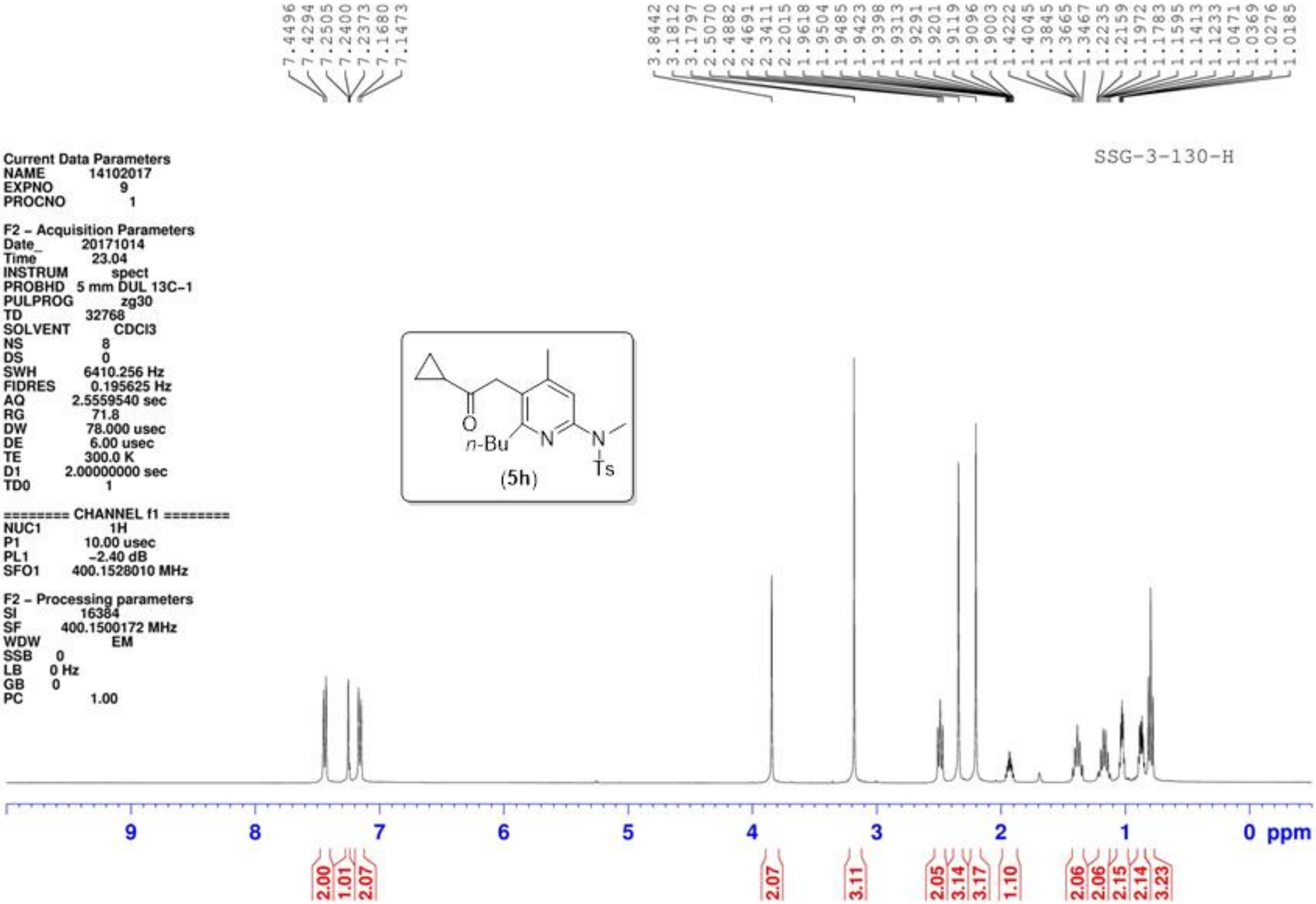

Current Data Parameters
NAME 14102017
EXPNO 9
PROCNO 1

F2 - Acquisition Parameters
Date_ 20171014
Time 23.04
INSTRUM spect
PROBHD 5 mm DUL 13C-1
PULPROG zg30
TD 32768
SOLVENT CDCl3
NS 8
DS 0
SWH 6410.256 Hz
FIDRES 0.195625 Hz
AQ 2.5559540 sec
RG 71.8
DW 78.000 usec
DE 6.00 usec
TE 300.0 K
D1 2.00000000 sec
TD0 1

======== CHANNEL f1 ========
NUC1 1H
P1 10.00 usec
PL1 -2.40 dB
SFO1 400.1528010 MHz

F2 - Processing parameters
SI 16384
SF 400.1500172 MHz
WDW EM
SSB 0
LB 0 Hz
GB 0
PC 1.00

(5h)

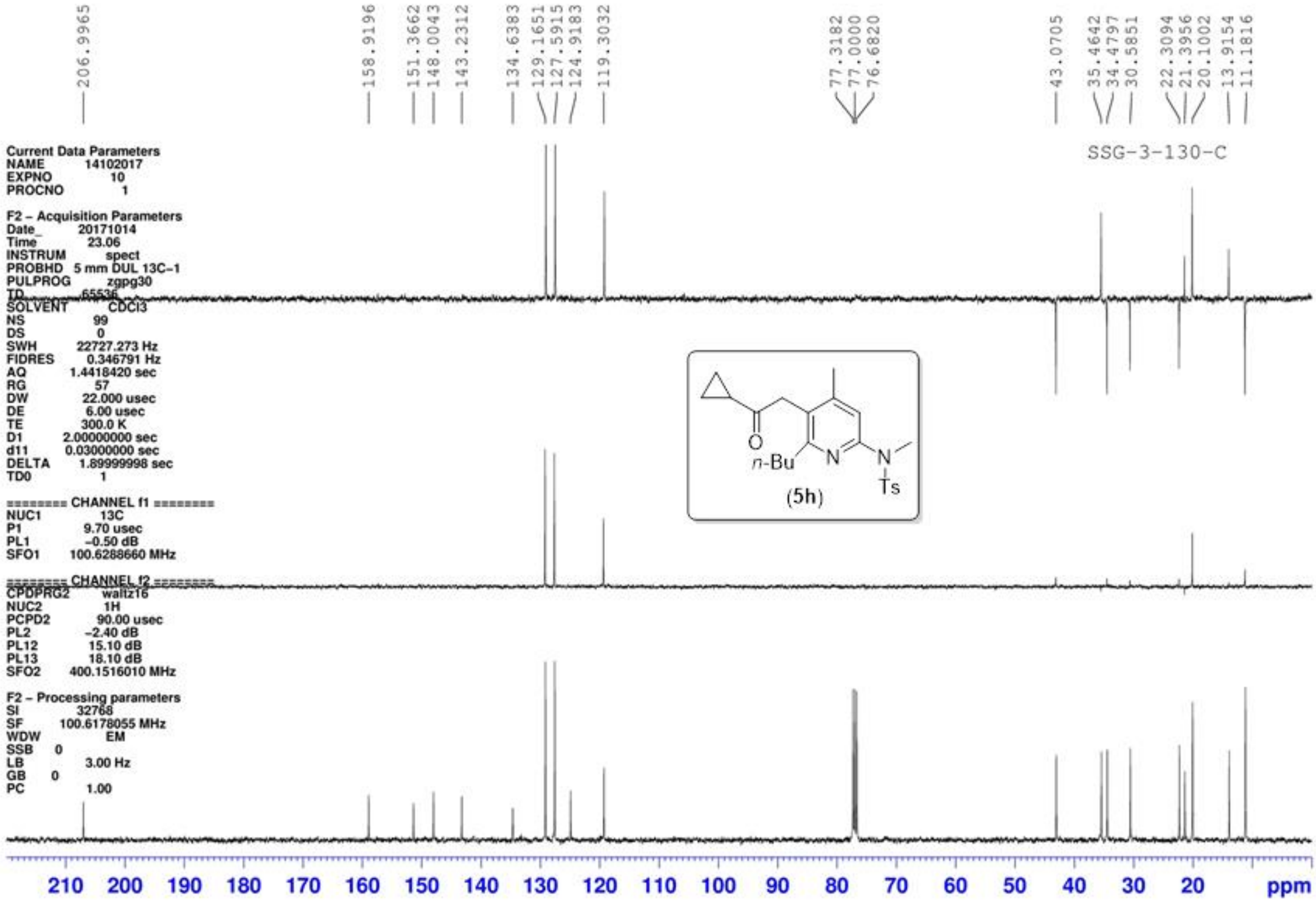
206.9965
158.9196
151.3662
148.0043
143.2312
134.6383
129.1651
127.5915
124.9183
119.3032
77.3182
77.0000
76.6820
43.0705
35.4642
34.4797
30.5851
22.3094
21.3956
20.1002
13.9154
11.1816
SSG-3-130-C
Current Data Parameters
NAME 14102017
EXPNO 10
PROCNO 1
F2 - Acquisition Parameters
Date_ 20171014
Time 23.06
INSTRUM spect
PROBHD 5 mm DUL 13C-1
PULPROG zgpg30
TD 65536
SOLVENT CDCl3
NS 99
DS 0
SWH 22727.273 Hz
FIDRES 0.346791 Hz
AQ 1.4418420 sec
RG 57
DW 22.000 usec
DE 6.00 usec
TE 300.0 K
D1 2.00000000 sec
d11 0.03000000 sec
DELTA 1.89999998 sec
TD0 1
======== CHANNEL f1 ========
NUC1 13C
P1 9.70 usec
PL1 -0.50 dB
SFO1 100.6288660 MHz
======== CHANNEL f2 ========
CPDPRG2 waltz16
NUC2 1H
PCPD2 90.00 usec
PL2 -2.40 dB
PL12 15.10 dB
PL13 18.10 dB
SFO2 400.1516010 MHz
F2 - Processing parameters
SI 32768
SF 100.6178055 MHz
WDW EM
SSB 0
LB 3.00 Hz
GB 0
PC 1.00
O
n-Bu
N
N
Ts
(5h)
210 200 190 180 170 160 150 140 130 120 110 100 90 80 70 60 50 40 30 20
ppm

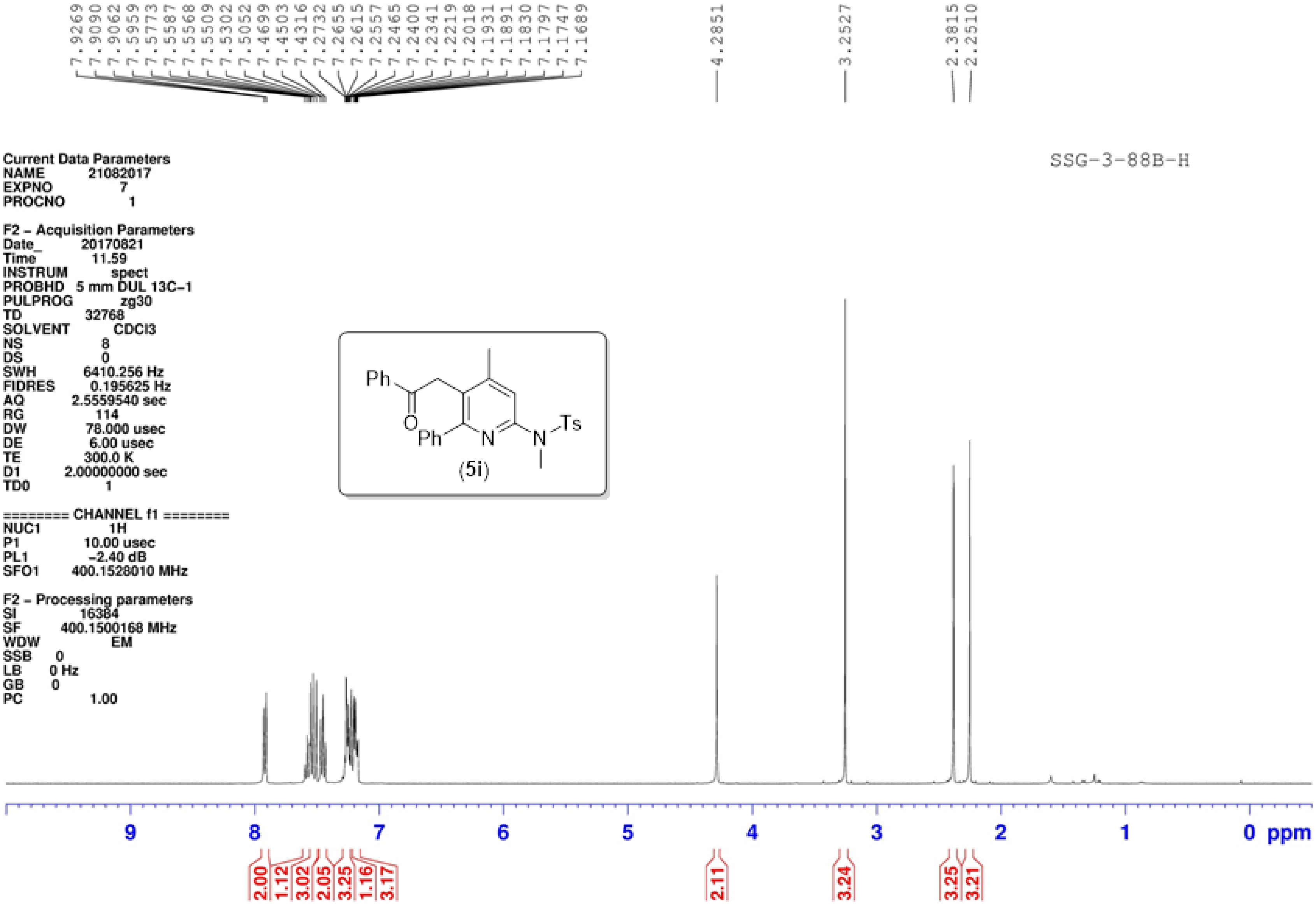
7.9269
7.9090
7.9062
7.5959
7.5773
7.5587
7.5568
7.5509
7.5302
7.5052
7.4699
7.4503
7.4316
7.2732
7.2655
7.2615
7.2557
7.2465
7.2400
7.2341
7.2219
7.2018
7.1931
7.1891
7.1830
7.1797
7.1747
7.1689
4.2851
3.2527
2.3815
2.2510
SSG-3-88B-H
Current Data Parameters
NAME 21082017
EXPNO 7
PROCNO 1
F2 - Acquisition Parameters
Date_ 20170821
Time 11.59
INSTRUM spect
PROBHD 5 mm DUL 13C-1
PULPROG zg30
TD 32768
SOLVENT CDCl3
NS 8
DS 0
SWH 6410.256 Hz
FIDRES 0.195625 Hz
AQ 2.5559540 sec
RG 114
DW 78.000 usec
DE 6.00 usec
TE 300.0 K
D1 2.00000000 sec
TD0 1
======== CHANNEL f1 ========
NUC1 1H
P1 10.00 usec
PL1 -2.40 dB
SFO1 400.1528010 MHz
F2 - Processing parameters
SI 16384
SF 400.1500168 MHz
WDW EM
SSB 0
LB 0 Hz
GB 0
PC 1.00
Ph
O
Ph
N
N
Ts
(5i)
9
8
7
6
5
4
3
2
1
0 ppm
2.00
1.12
3.02
2.05
3.25
1.16
3.17
2.11
3.24
3.25
3.21

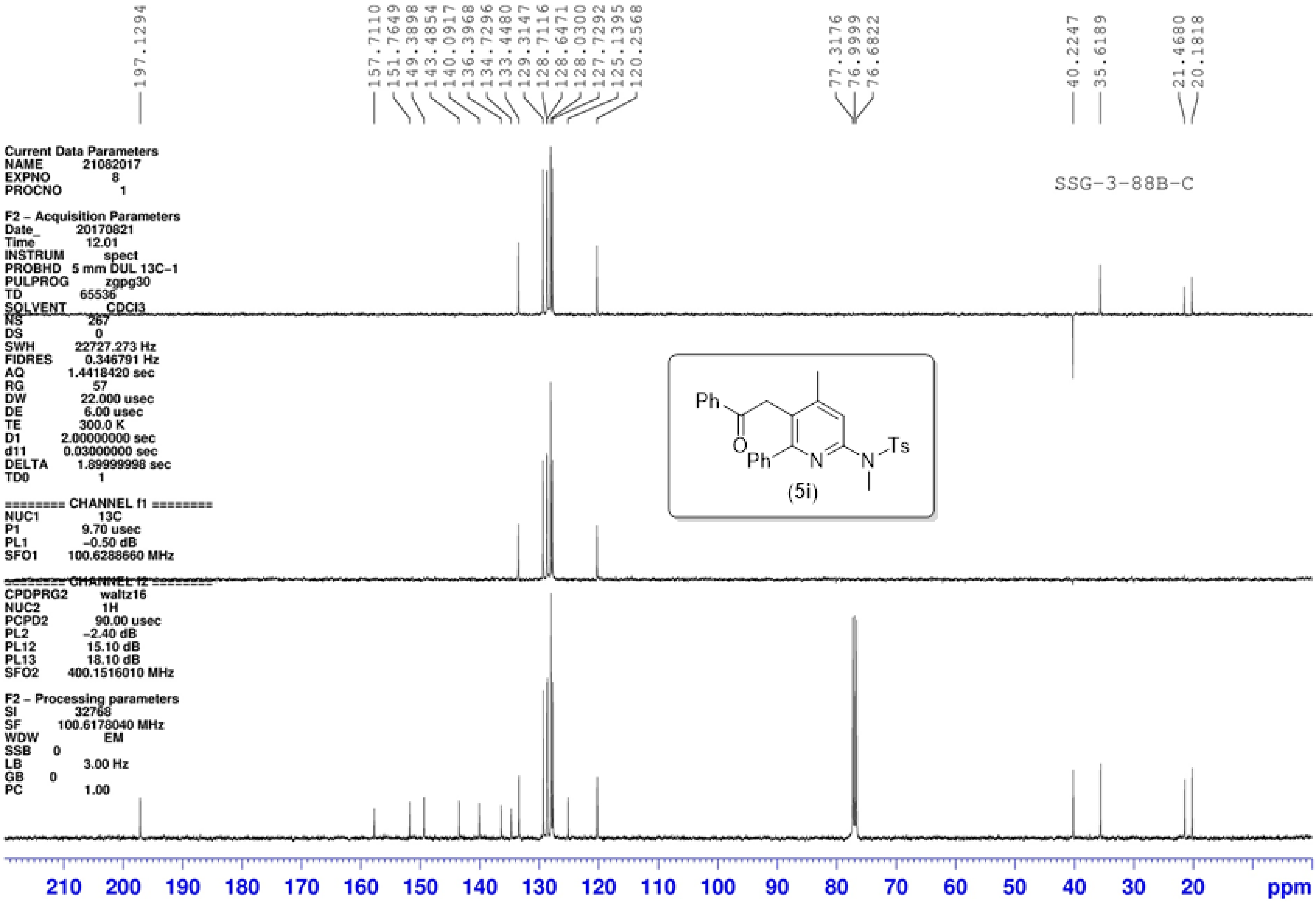
197.1294
157.7110
151.7649
149.3898
143.4854
140.0917
136.3968
134.7296
133.4480
129.3147
128.7116
128.6471
128.0300
127.7292
125.1395
120.2568
77.3176
76.9999
76.6822
40.2247
35.6189
21.4680
20.1818
SSG-3-88B-C
Current Data Parameters
NAME 21082017
EXPNO 8
PROCNO 1
F2 – Acquisition Parameters
Date_ 20170821
Time 12.01
INSTRUM spect
PROBHD 5 mm DUL 13C–1
PULPROG zgpg30
TD 65536
SOLVENT CDCl3
NS 267
DS 0
SWH 22727.273 Hz
FIDRES 0.346791 Hz
AQ 1.4418420 sec
RG 57
DW 22.000 usec
DE 6.00 usec
TE 300.0 K
D1 2.00000000 sec
d11 0.03000000 sec
DELTA 1.89999998 sec
TD0 1
======== CHANNEL f1 ========
NUC1 13C
P1 9.70 usec
PL1 –0.50 dB
SFO1 100.6288660 MHz
======== CHANNEL f2 ========
CPDPRG2 waltz16
NUC2 1H
PCPD2 90.00 usec
PL2 –2.40 dB
PL12 15.10 dB
PL13 18.10 dB
SFO2 400.1516010 MHz
F2 – Processing parameters
SI 32768
SF 100.6178040 MHz
WDW EM
SSB 0
LB 3.00 Hz
GB 0
PC 1.00
Ph
O
Ph
N
N
Ts
(5i)
210 200 190 180 170 160 150 140 130 120 110 100 90 80 70 60 50 40 30 20 ppm

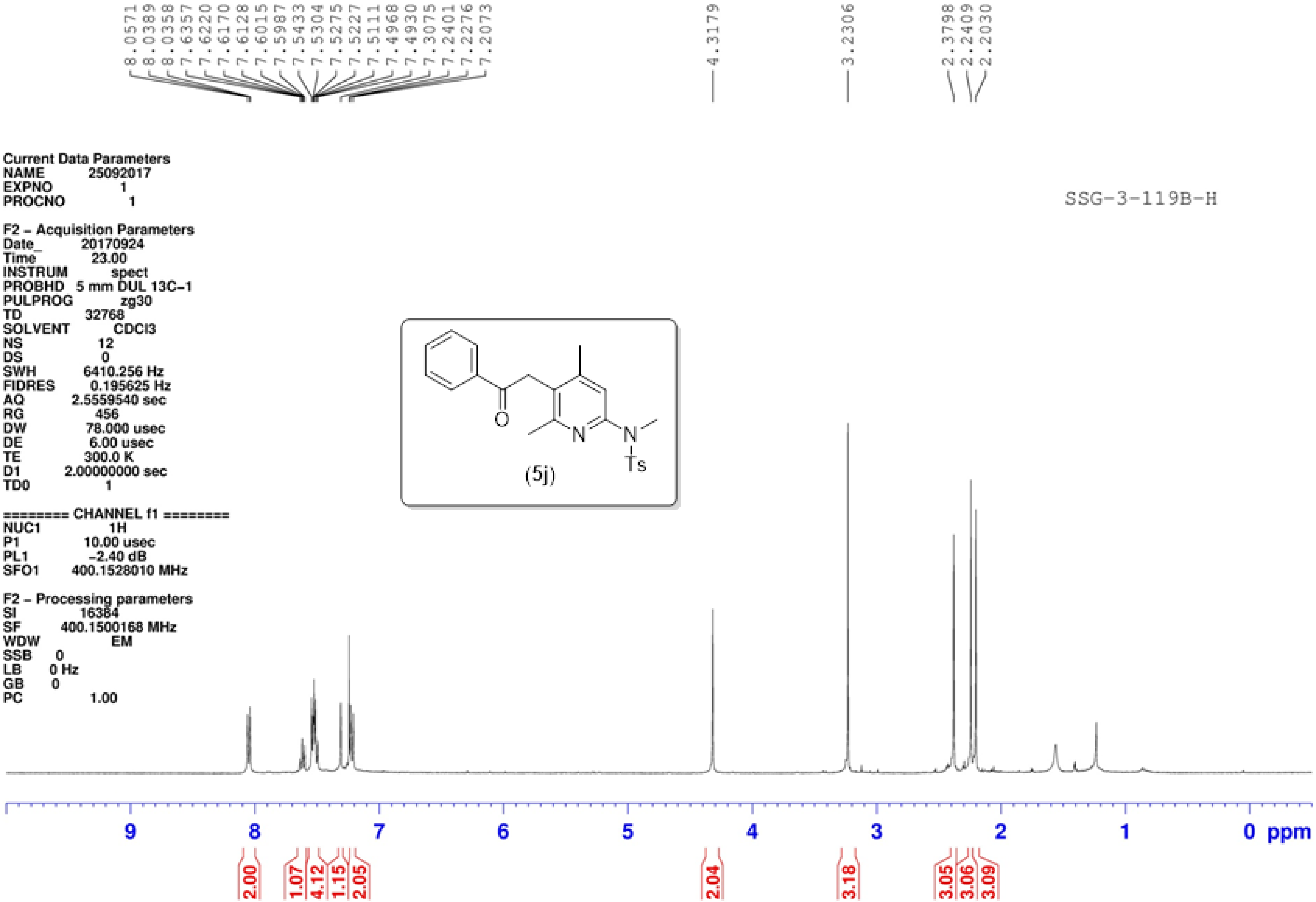
8.0571
8.0389
8.0358
7.6357
7.6220
7.6170
7.6128
7.6015
7.5987
7.5433
7.5304
7.5275
7.5227
7.5111
7.4968
7.4930
7.3075
7.2401
7.2276
7.2073
4.3179
3.2306
2.3798
2.2409
2.2030
Current Data Parameters
NAME 25092017
EXPNO 1
PROCNO 1
F2 - Acquisition Parameters
Date_ 20170924
Time 23.00
INSTRUM spect
PROBHD 5 mm DUL 13C-1
PULPROG zg30
TD 32768
SOLVENT CDCl3
NS 12
DS 0
SWH 6410.256 Hz
FIDRES 0.195625 Hz
AQ 2.5559540 sec
RG 456
DW 78.000 usec
DE 6.00 usec
TE 300.0 K
D1 2.00000000 sec
TD0 1
======== CHANNEL f1 ========
NUC1 1H
P1 10.00 usec
PL1 -2.40 dB
SFO1 400.1528010 MHz
F2 - Processing parameters
SI 16384
SF 400.1500168 MHz
WDW EM
SSB 0
LB 0 Hz
GB 0
PC 1.00
SSG-3-119B-H
O
N
N
Ts
(5j)
9
8
7
6
5
4
3
2
1
0 ppm
2.00
1.07
4.12
1.15
2.05
2.04
3.18
3.05
3.06
3.09

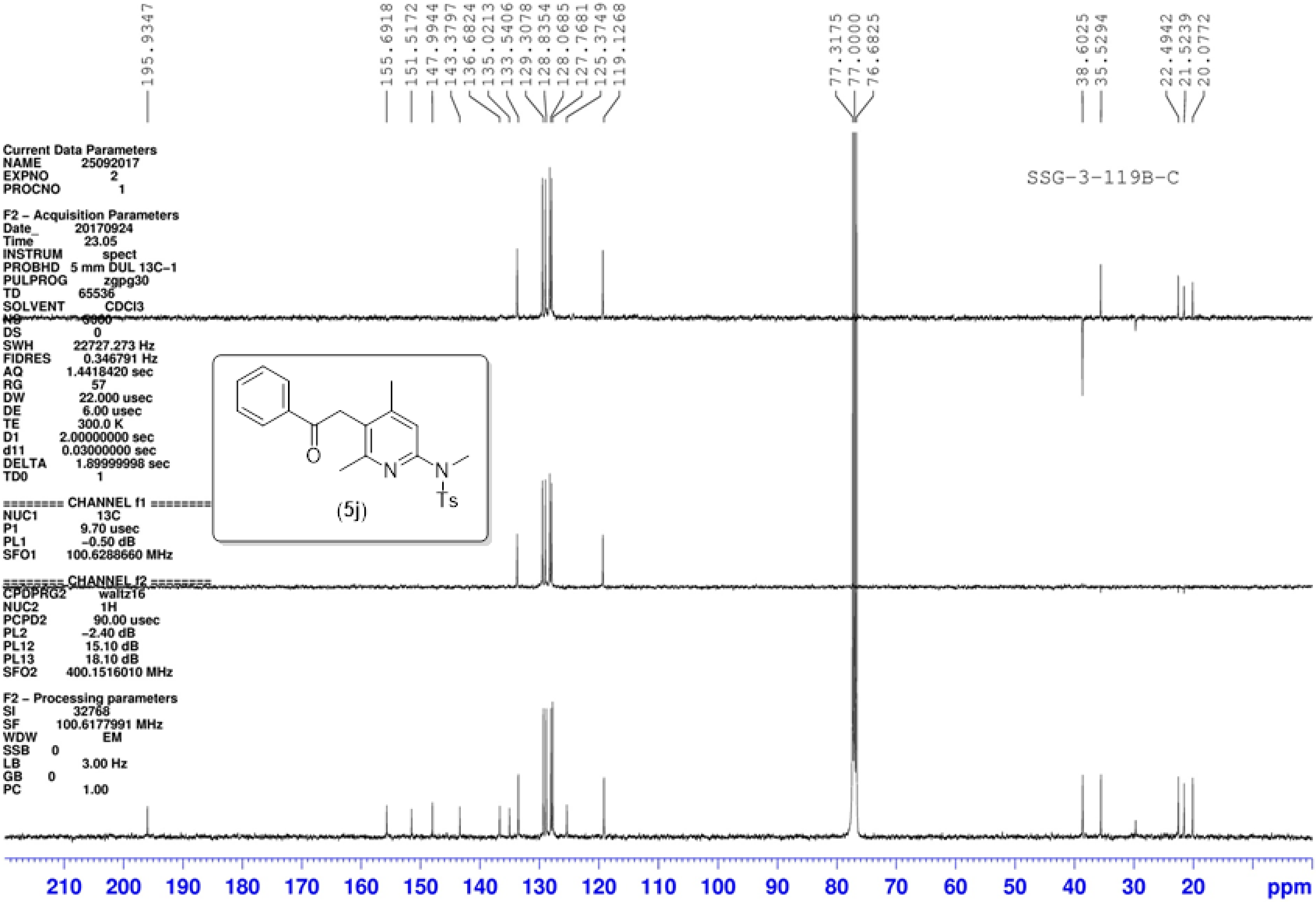
195.9347
155.6918
151.5172
147.9944
143.3797
136.6824
135.0213
133.5406
129.3078
128.8354
128.0685
127.7681
125.3749
119.1268
77.3175
77.0000
76.6825
38.6025
35.5294
22.4942
21.5239
20.0772
SSG-3-119B-C
Current Data Parameters
NAME 25092017
EXPNO 2
PROCNO 1
F2 - Acquisition Parameters
Date_ 20170924
Time 23.05
INSTRUM spect
PROBHD 5 mm DUL 13C-1
PULPROG zgpg30
TD 65536
SOLVENT CDCl3
DS 0
SWH 22727.273 Hz
FIDRES 0.346791 Hz
AQ 1.4418420 sec
RG 57
DW 22.000 usec
DE 6.00 usec
TE 300.0 K
D1 2.00000000 sec
d11 0.03000000 sec
DELTA 1.89999998 sec
TD0 1
======== CHANNEL f1 ========
NUC1 13C
P1 9.70 usec
PL1 -0.50 dB
SFO1 100.6288660 MHz
======== CHANNEL f2 ========
CPDPRG2 waltz16
NUC2 1H
PCPD2 90.00 usec
PL2 -2.40 dB
PL12 15.10 dB
PL13 18.10 dB
SFO2 400.1516010 MHz
F2 - Processing parameters
SI 32768
SF 100.6177991 MHz
WDW EM
SSB 0
LB 3.00 Hz
GB 0
PC 1.00
O
N
N
Ts
(5j)
210 200 190 180 170 160 150 140 130 120 110 100 90 80 70 60 50 40 30 20 ppm

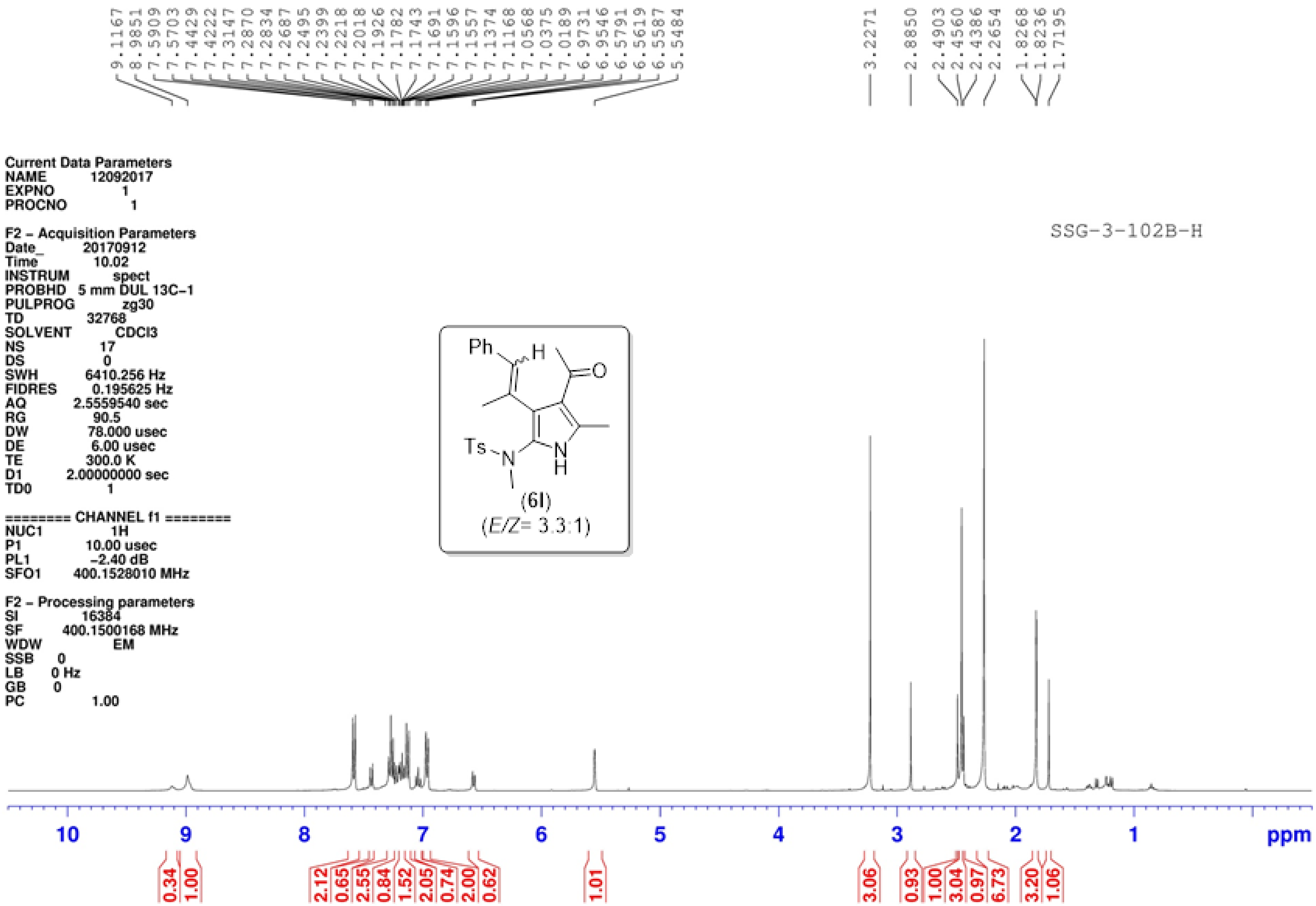
9.1167
8.9851
7.5909
7.5703
7.4429
7.4222
7.3147
7.2870
7.2834
7.2687
7.2495
7.2399
7.2218
7.2018
7.1926
7.1782
7.1743
7.1691
7.1596
7.1557
7.1374
7.1168
7.0568
7.0375
7.0189
6.9731
6.9546
6.5791
6.5619
6.5587
5.5484
3.2271
2.8850
2.4903
2.4560
2.4386
2.2654
1.8268
1.8236
1.7195
Current Data Parameters
NAME 12092017
EXPNO 1
PROCNO 1
F2 - Acquisition Parameters
Date_ 20170912
Time 10.02
INSTRUM spect
PROBHD 5 mm DUL 13C-1
PULPROG zg30
TD 32768
SOLVENT CDCl3
NS 17
DS 0
SWH 6410.256 Hz
FIDRES 0.195625 Hz
AQ 2.5559540 sec
RG 90.5
DW 78.000 usec
DE 6.00 usec
TE 300.0 K
D1 2.00000000 sec
TD0 1
======== CHANNEL f1 ========
NUC1 1H
P1 10.00 usec
PL1 -2.40 dB
SFO1 400.1528010 MHz
F2 - Processing parameters
SI 16384
SF 400.1500168 MHz
WDW EM
SSB 0
LB 0 Hz
GB 0
PC 1.00
SSG-3-102B-H
Ph
H
O
Ts
N
N
H
(6l)
(E/Z= 3.3:1)
10
9
8
7
6
5
4
3
2
1
ppm
0.34
1.00
2.12
0.65
2.55
0.84
1.52
2.05
0.74
2.00
0.62
1.01
3.06
0.93
1.00
3.04
0.97
6.73
3.20
1.06

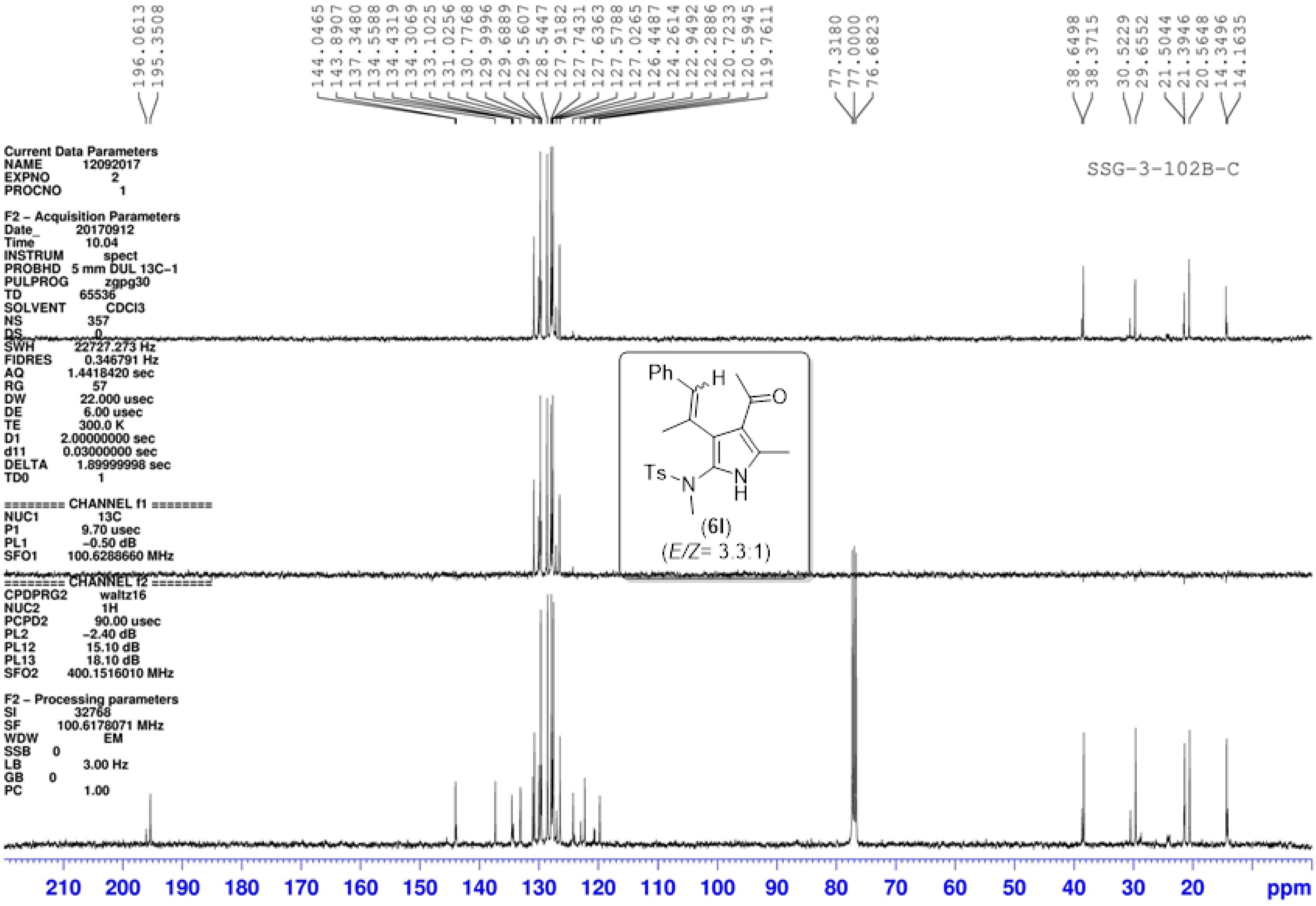
196.0613
195.3508
144.0465
143.8907
137.3480
134.5588
134.4319
134.3069
133.1025
131.0256
130.7768
129.9996
129.6889
129.5607
128.5447
127.9182
127.7431
127.6363
127.5788
127.0265
126.4487
124.2614
122.9492
122.2886
120.7233
120.5945
119.7611
77.3180
77.0000
76.6823
38.6498
38.3715
30.5229
29.6552
21.5044
21.3946
20.5648
14.3496
14.1635
SSG-3-102B-C
Current Data Parameters
NAME 12092017
EXPNO 2
PROCNO 1
F2 – Acquisition Parameters
Date_ 20170912
Time 10.04
INSTRUM spect
PROBHD 5 mm DUL 13C-1
PULPROG zgpg30
TD 65536
SOLVENT CDCl3
NS 357
DS 0
SWH 22727.273 Hz
FIDRES 0.346791 Hz
AQ 1.4418420 sec
RG 57
DW 22.000 usec
DE 6.00 usec
TE 300.0 K
D1 2.00000000 sec
d11 0.03000000 sec
DELTA 1.89999998 sec
TD0 1
======== CHANNEL f1 ========
NUC1 13C
P1 9.70 usec
PL1 -0.50 dB
SFO1 100.6288660 MHz
======== CHANNEL f2 ========
CPDPRG2 waltz16
NUC2 1H
PCPD2 90.00 usec
PL2 -2.40 dB
PL12 15.10 dB
PL13 18.10 dB
SFO2 400.1516010 MHz
F2 – Processing parameters
SI 32768
SF 100.6178071 MHz
WDW EM
SSB 0
LB 3.00 Hz
GB 0
PC 1.00
Ph
H
O
Ts
N
N
H
(6l)
(E/Z= 3.3:1)
210
200
190
180
170
160
150
140
130
120
110
100
90
80
70
60
50
40
30
20
ppm

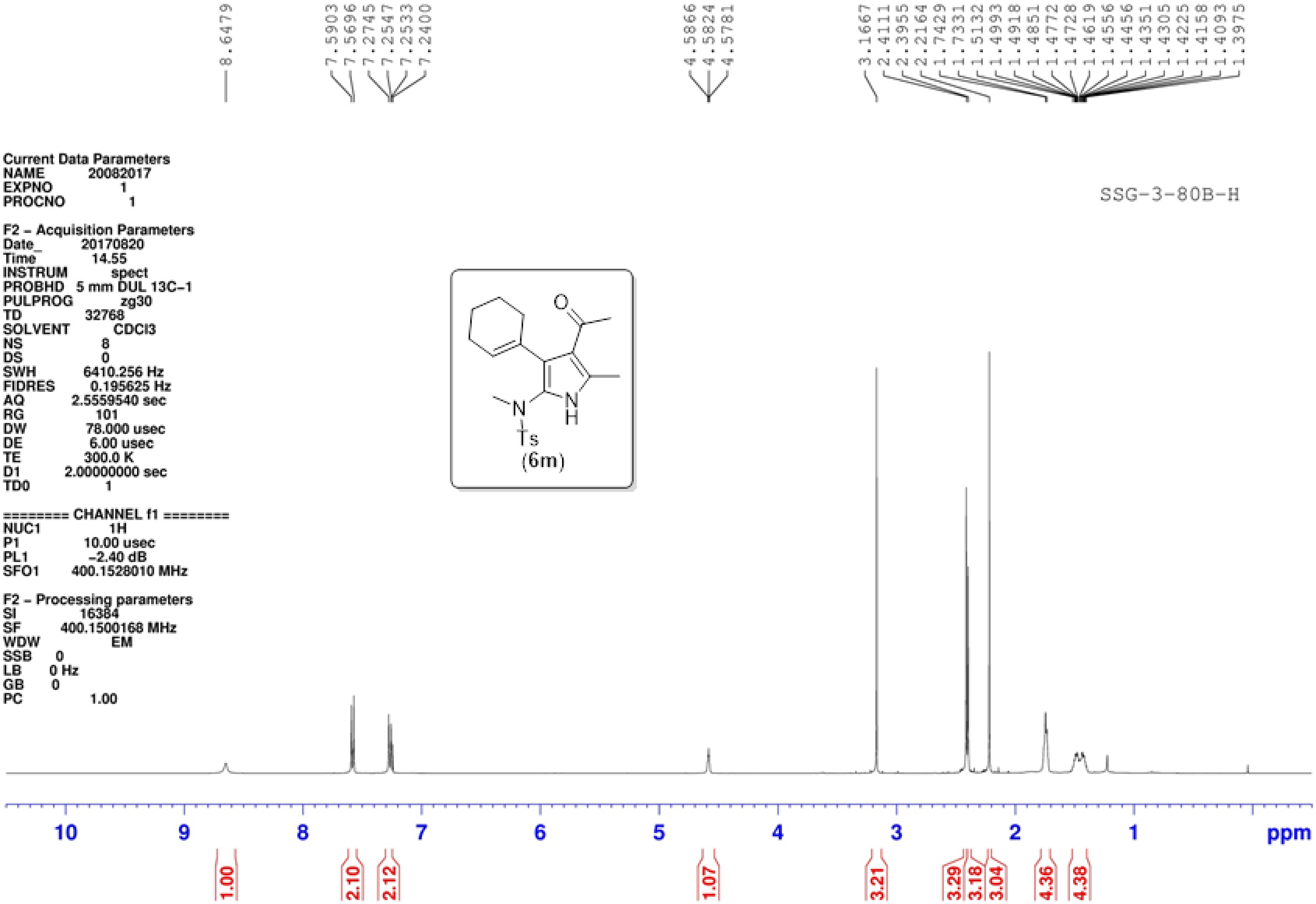
8.6479
7.5903
7.5696
7.2745
7.2547
7.2533
7.2400
4.5866
4.5824
4.5781
3.1667
2.4111
2.3955
2.2164
1.7429
1.7331
1.5132
1.4993
1.4918
1.4851
1.4772
1.4728
1.4619
1.4556
1.4456
1.4351
1.4305
1.4225
1.4158
1.4093
1.3975
Current Data Parameters
NAME 20082017
EXPNO 1
PROCNO 1
F2 - Acquisition Parameters
Date_ 20170820
Time 14.55
INSTRUM spect
PROBHD 5 mm DUL 13C-1
PULPROG zg30
TD 32768
SOLVENT CDCl3
NS 8
DS 0
SWH 6410.256 Hz
FIDRES 0.195625 Hz
AQ 2.5559540 sec
RG 101
DW 78.000 usec
DE 6.00 usec
TE 300.0 K
D1 2.00000000 sec
TD0 1
======== CHANNEL f1 ========
NUC1 1H
P1 10.00 usec
PL1 -2.40 dB
SFO1 400.1528010 MHz
F2 - Processing parameters
SI 16384
SF 400.1500168 MHz
WDW EM
SSB 0
LB 0 Hz
GB 0
PC 1.00
SSG-3-80B-H
O
N
H
N
Ts
(6m)
10
9
8
7
6
5
4
3
2
1
ppm
1.00
2.10
2.12
1.07
3.21
3.29
3.18
3.04
4.36
4.38

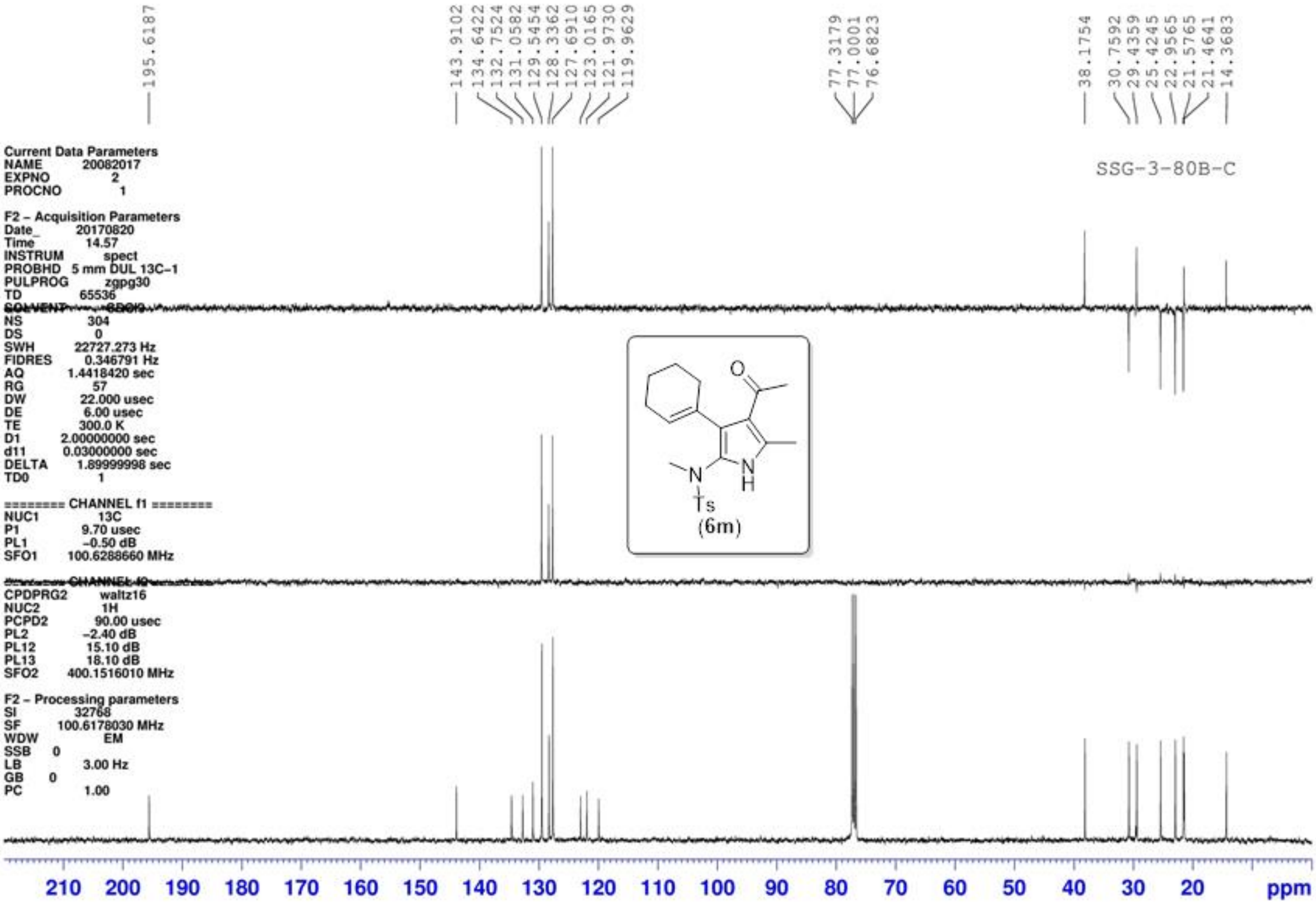
195.6187
143.9102
134.6422
132.7524
131.0582
129.5454
128.3362
127.6910
123.0165
121.9730
119.9629
77.3179
77.0001
76.6823
38.1754
30.7592
29.4359
25.4245
22.9565
21.5765
21.4641
14.3683
SSG-3-80B-C
Current Data Parameters
NAME 20082017
EXPNO 2
PROCNO 1
F2 - Acquisition Parameters
Date_ 20170820
Time 14.57
INSTRUM spect
PROBHD 5 mm DUL 13C-1
PULPROG zgpg30
TD 65536
NS 304
DS 0
SWH 22727.273 Hz
FIDRES 0.346791 Hz
AQ 1.4418420 sec
RG 57
DW 22.000 usec
DE 6.00 usec
TE 300.0 K
D1 2.00000000 sec
d11 0.03000000 sec
DELTA 1.89999998 sec
TD0 1
======== CHANNEL f1 ========
NUC1 13C
P1 9.70 usec
PL1 -0.50 dB
SFO1 100.6288660 MHz
CPDPRG2 waltz16
NUC2 1H
PCPD2 90.00 usec
PL2 -2.40 dB
PL12 15.10 dB
PL13 18.10 dB
SFO2 400.1516010 MHz
F2 - Processing parameters
SI 32768
SF 100.6178030 MHz
WDW EM
SSB 0
LB 3.00 Hz
GB 0
PC 1.00
O
N
H
N
Ts
(6m)
210 200 190 180 170 160 150 140 130 120 110 100 90 80 70 60 50 40 30 20 ppm

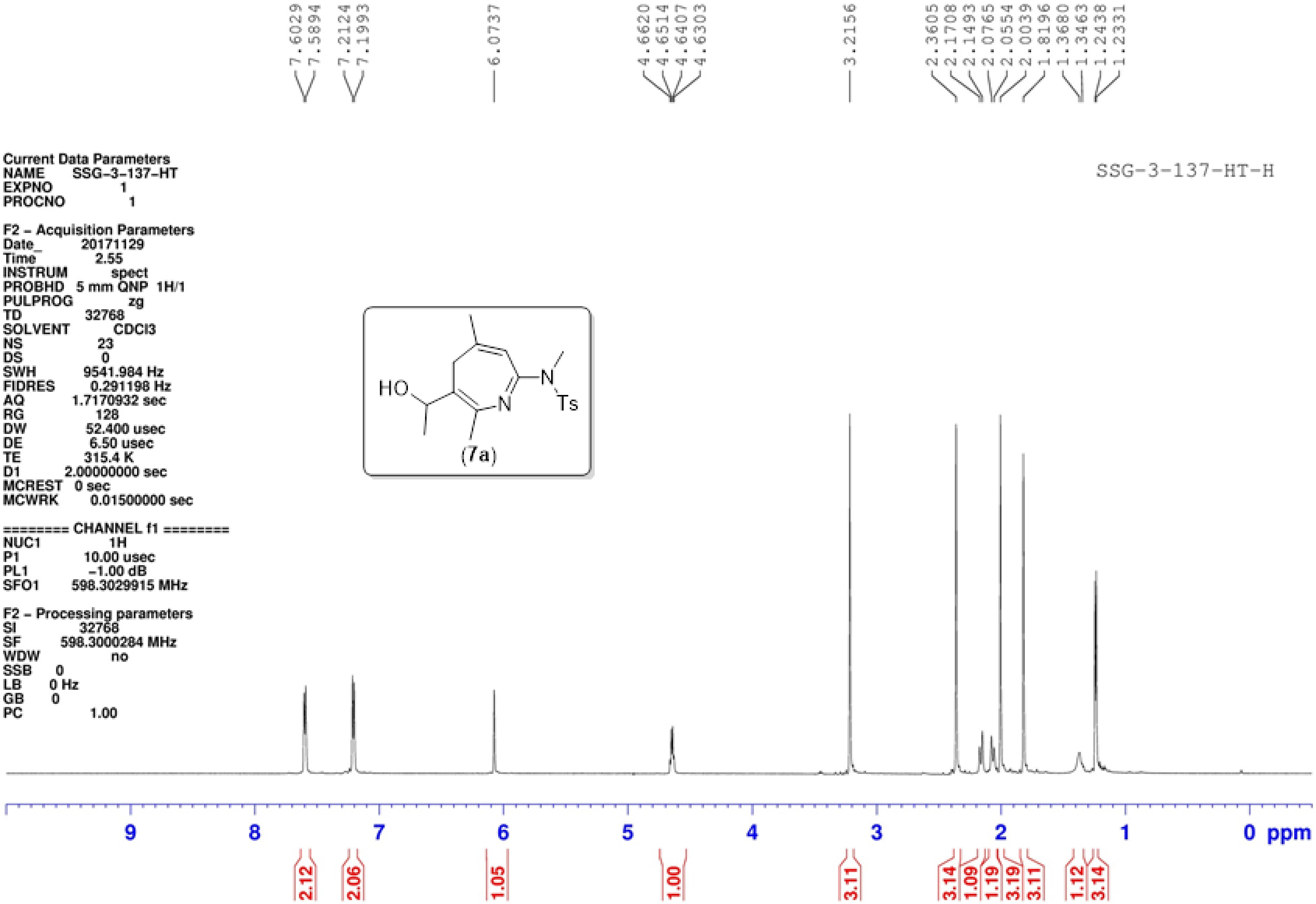
7.6029
7.5894
7.2124
7.1993
6.0737
4.6620
4.6514
4.6407
4.6303
3.2156
2.3605
2.1708
2.1493
2.0765
2.0554
2.0039
1.8196
1.3680
1.3463
1.2438
1.2331
SSG-3-137-HT-H
Current Data Parameters
NAME SSG-3-137-HT
EXPNO 1
PROCNO 1
F2 - Acquisition Parameters
Date_ 20171129
Time 2.55
INSTRUM spect
PROBHD 5 mm QNP 1H/1
PULPROG zg
TD 32768
SOLVENT CDCl3
NS 23
DS 0
SWH 9541.984 Hz
FIDRES 0.291198 Hz
AQ 1.7170932 sec
RG 128
DW 52.400 usec
DE 6.50 usec
TE 315.4 K
D1 2.00000000 sec
MCREST 0 sec
MCWRK 0.01500000 sec
======== CHANNEL f1 ========
NUC1 1H
P1 10.00 usec
PL1 -1.00 dB
SFO1 598.3029915 MHz
F2 - Processing parameters
SI 32768
SF 598.3000284 MHz
WDW no
SSB 0
LB 0 Hz
GB 0
PC 1.00
HO
N
N
Ts
(7a)
9
8
7
6
5
4
3
2
1
0 ppm
2.12
2.06
1.05
1.00
3.11
3.14
1.09
1.19
3.19
3.11
1.12
3.14

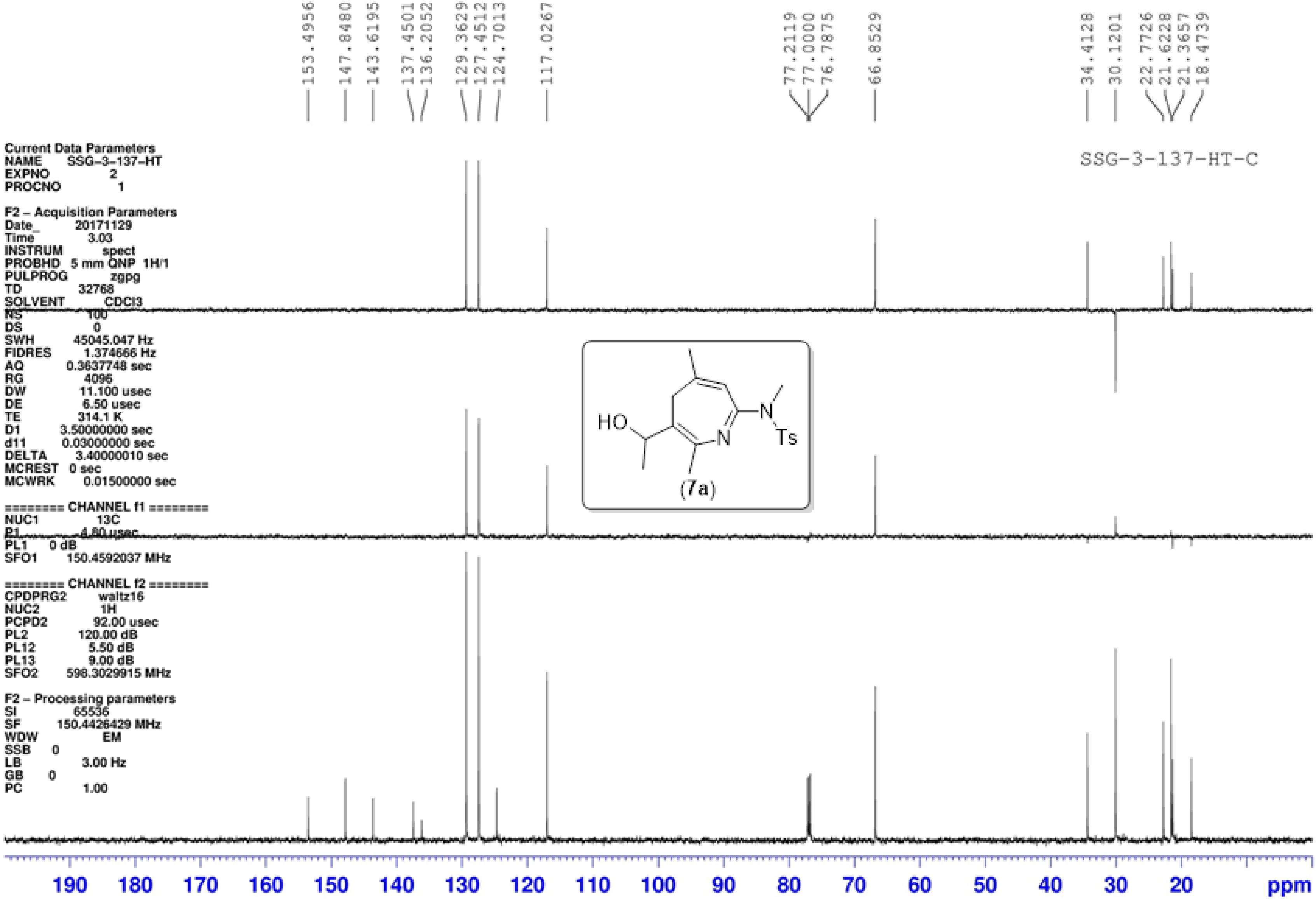
153.4956
147.8480
143.6195
137.4501
136.2052
129.3629
127.4512
124.7013
117.0267
77.2119
77.0000
76.7875
66.8529
34.4128
30.1201
22.7726
21.6228
21.3657
18.4739
SSG-3-137-HT-C
Current Data Parameters
NAME SSG-3-137-HT
EXPNO 2
PROCNO 1
F2 - Acquisition Parameters
Date_ 20171129
Time 3.03
INSTRUM spect
PROBHD 5 mm QNP 1H/1
PULPROG zgpg
TD 32768
SOLVENT CDCl3
NS 100
DS 0
SWH 45045.047 Hz
FIDRES 1.374666 Hz
AQ 0.3637748 sec
RG 4096
DW 11.100 usec
DE 6.50 usec
TE 314.1 K
D1 3.50000000 sec
d11 0.03000000 sec
DELTA 3.40000010 sec
MCREST 0 sec
MCWRK 0.01500000 sec
======== CHANNEL f1 ========
NUC1 13C
P1 4.80 usec
PL1 0 dB
SFO1 150.4592037 MHz
======== CHANNEL f2 ========
CPDPRG2 waltz16
NUC2 1H
PCPD2 92.00 usec
PL2 120.00 dB
PL12 5.50 dB
PL13 9.00 dB
SFO2 598.3029915 MHz
F2 - Processing parameters
SI 65536
SF 150.4426429 MHz
WDW EM
SSB 0
LB 3.00 Hz
GB 0
PC 1.00
HO
N
N
Ts
(7a)
190 180 170 160 150 140 130 120 110 100 90 80 70 60 50 40 30 20 ppm

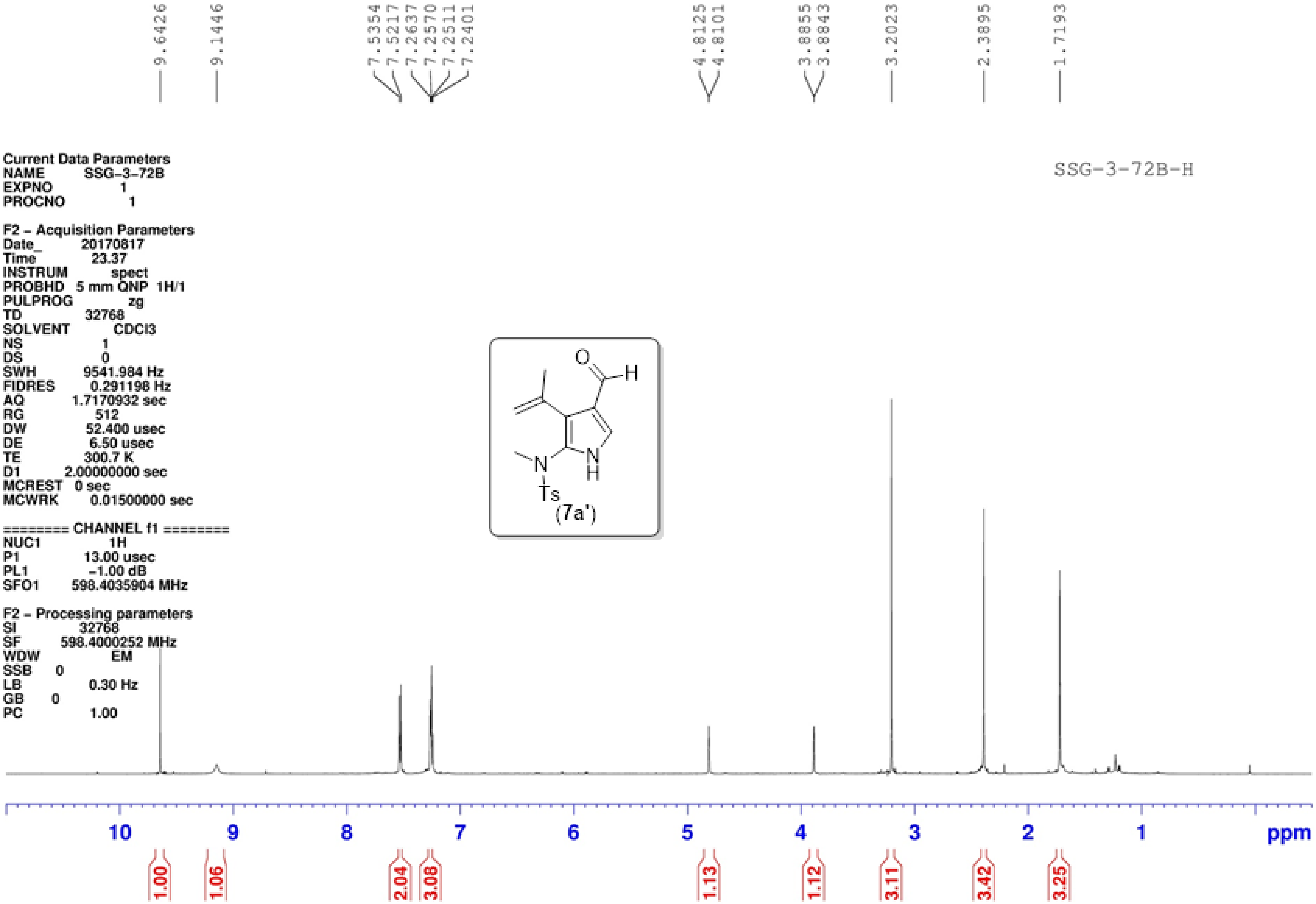
9.6426
9.1446
7.5354
7.5217
7.2637
7.2570
7.2511
7.2401
4.8125
4.8101
3.8855
3.8843
3.2023
2.3895
1.7193
Current Data Parameters
NAME SSG-3-72B
EXPNO 1
PROCNO 1
F2 - Acquisition Parameters
Date_ 20170817
Time 23.37
INSTRUM spect
PROBHD 5 mm QNP 1H/1
PULPROG zg
TD 32768
SOLVENT CDCl3
NS 1
DS 0
SWH 9541.984 Hz
FIDRES 0.291198 Hz
AQ 1.7170932 sec
RG 512
DW 52.400 usec
DE 6.50 usec
TE 300.7 K
D1 2.00000000 sec
MCREST 0 sec
MCWRK 0.01500000 sec
======== CHANNEL f1 ========
NUC1 1H
P1 13.00 usec
PL1 -1.00 dB
SFO1 598.4035904 MHz
F2 - Processing parameters
SI 32768
SF 598.4000252 MHz
WDW EM
SSB 0
LB 0.30 Hz
GB 0
PC 1.00
SSG-3-72B-H
O
H
N
N
H
Ts
(7a')
10
9
8
7
6
5
4
3
2
1
ppm
1.00
1.06
2.04
3.08
1.13
1.12
3.11
3.42
3.25

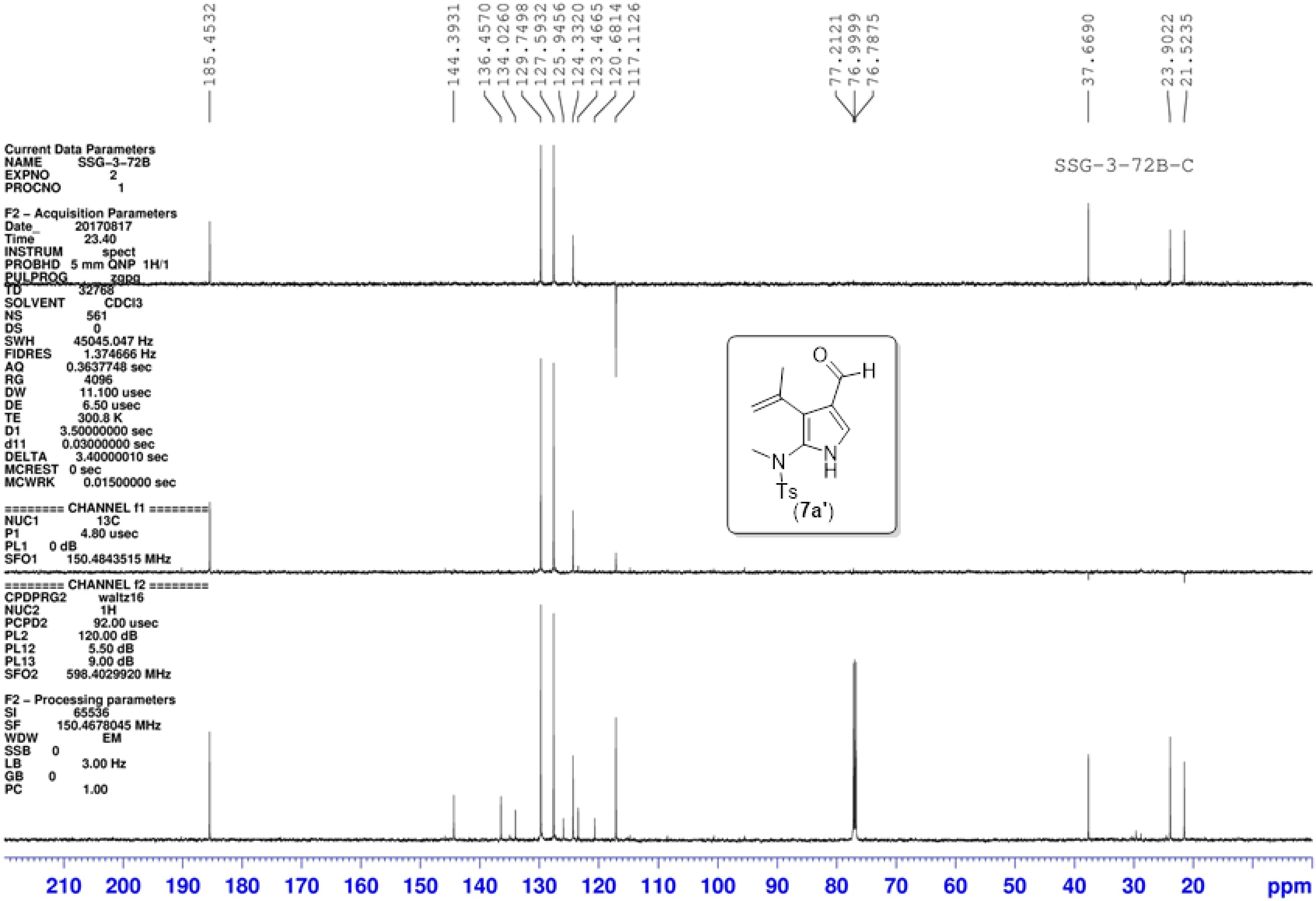
185.4532
144.3931
136.4570
134.0260
129.7498
127.5932
125.9456
124.3320
123.4665
120.6814
117.1126
77.2121
76.9999
76.7875
37.6690
23.9022
21.5235
Current Data Parameters
NAME SSG-3-72B
EXPNO 2
PROCNO 1
F2 - Acquisition Parameters
Date_ 20170817
Time 23.40
INSTRUM spect
PROBHD 5 mm QNP 1H/1
PULPROG zgpg
TD 32768
SOLVENT CDCl3
NS 561
DS 0
SWH 45045.047 Hz
FIDRES 1.374666 Hz
AQ 0.3637748 sec
RG 4096
DW 11.100 usec
DE 6.50 usec
TE 300.8 K
D1 3.50000000 sec
d11 0.03000000 sec
DELTA 3.40000010 sec
MCREST 0 sec
MCWRK 0.01500000 sec
======== CHANNEL f1 ========
NUC1 13C
P1 4.80 usec
PL1 0 dB
SFO1 150.4843515 MHz
======== CHANNEL f2 ========
CPDPRG2 waltz16
NUC2 1H
PCPD2 92.00 usec
PL2 120.00 dB
PL12 5.50 dB
PL13 9.00 dB
SFO2 598.4029920 MHz
F2 - Processing parameters
SI 65536
SF 150.4678045 MHz
WDW EM
SSB 0
LB 3.00 Hz
GB 0
PC 1.00
SSG-3-72B-C
O
H
N
NH
H
Ts
(7a')
210 200 190 180 170 160 150 140 130 120 110 100 90 80 70 60 50 40 30 20 ppm

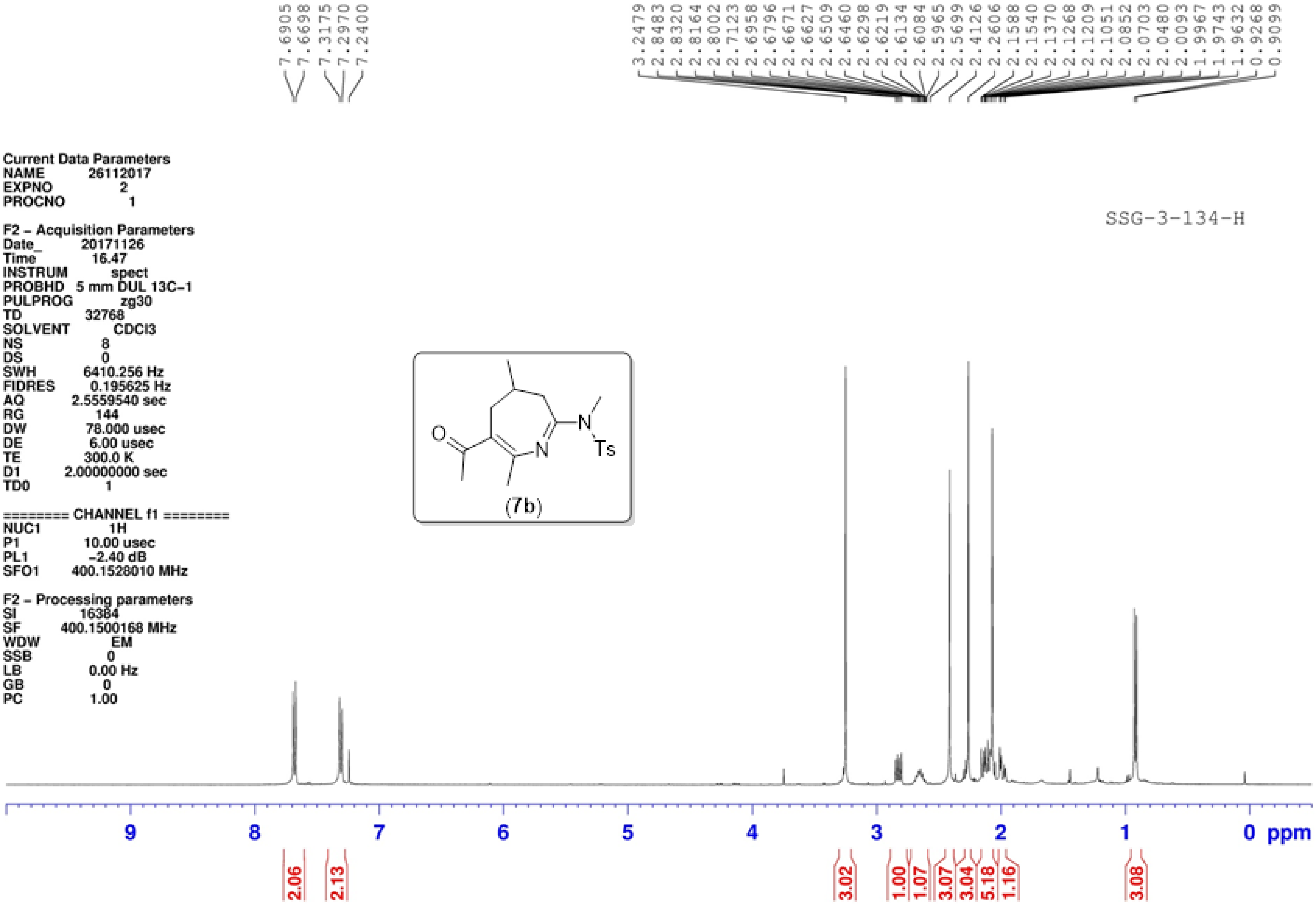
7.6905
7.6698
7.3175
7.2970
7.2400
3.2479
2.8483
2.8320
2.8164
2.8002
2.7123
2.6958
2.6796
2.6671
2.6627
2.6509
2.6460
2.6298
2.6219
2.6134
2.6084
2.5965
2.5699
2.4126
2.2606
2.1588
2.1540
2.1370
2.1268
2.1209
2.1051
2.0852
2.0703
2.0480
2.0093
1.9967
1.9743
1.9632
0.9268
0.9099
Current Data Parameters
NAME 26112017
EXPNO 2
PROCNO 1
F2 - Acquisition Parameters
Date_ 20171126
Time 16.47
INSTRUM spect
PROBHD 5 mm DUL 13C-1
PULPROG zg30
TD 32768
SOLVENT CDCl3
NS 8
DS 0
SWH 6410.256 Hz
FIDRES 0.195625 Hz
AQ 2.5559540 sec
RG 144
DW 78.000 usec
DE 6.00 usec
TE 300.0 K
D1 2.00000000 sec
TD0 1
======== CHANNEL f1 ========
NUC1 1H
P1 10.00 usec
PL1 -2.40 dB
SFO1 400.1528010 MHz
F2 - Processing parameters
SI 16384
SF 400.1500168 MHz
WDW EM
SSB 0
LB 0.00 Hz
GB 0
PC 1.00
SSG-3-134-H
O
N
N
Ts
(7b)
9
8
7
6
5
4
3
2
1
0 ppm
2.06
2.13
3.02
1.00
1.07
3.07
3.04
5.18
1.16
3.08

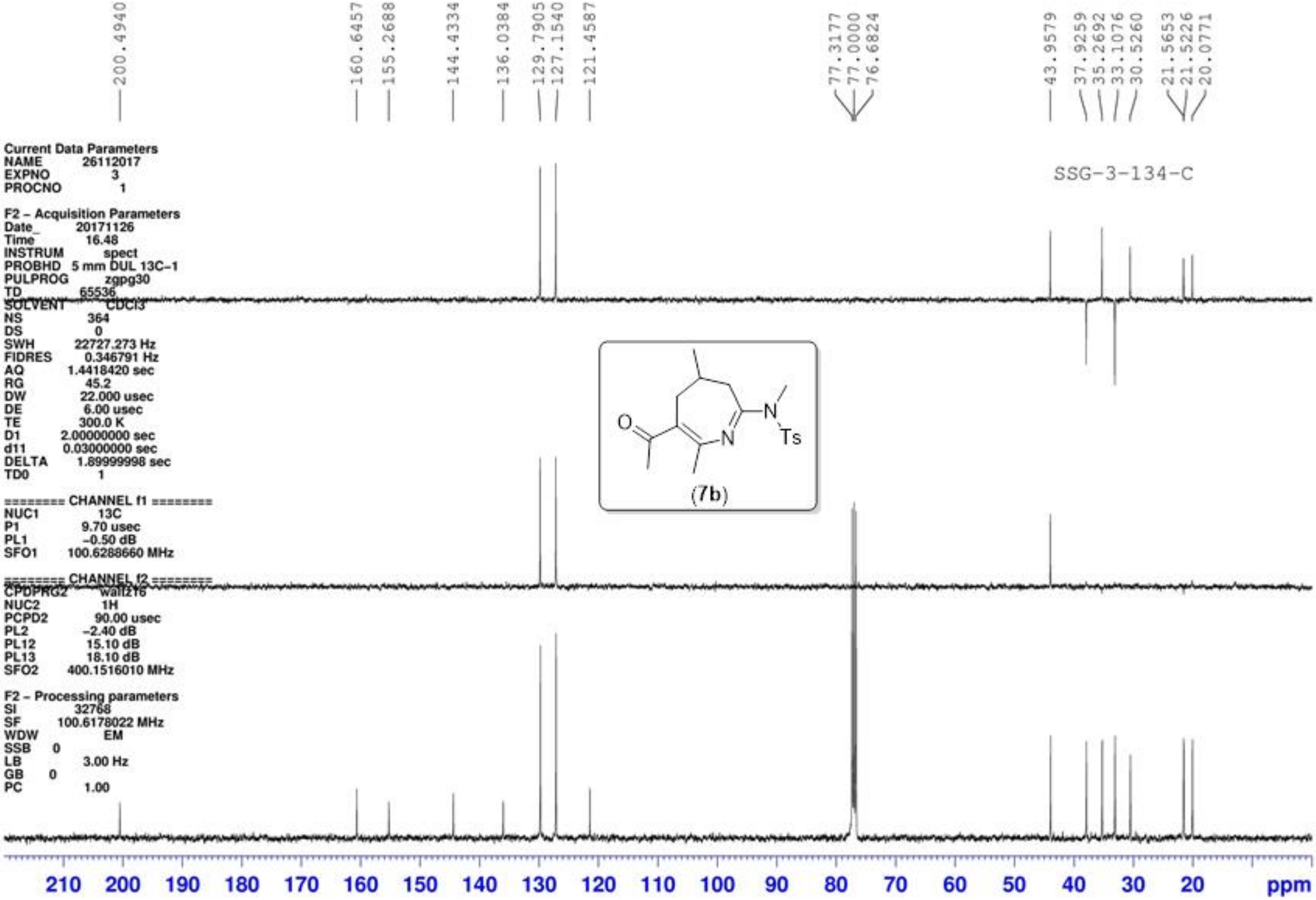
200.4940
160.6457
155.2688
144.4334
136.0384
129.7905
127.1540
121.4587
77.3177
77.0000
76.6824
43.9579
37.9259
35.2692
33.1076
30.5260
21.5653
21.5226
20.0771
SSG-3-134-C
Current Data Parameters
NAME 26112017
EXPNO 3
PROCNO 1
F2 - Acquisition Parameters
Date_ 20171126
Time 16.48
INSTRUM spect
PROBHD 5 mm DUL 13C-1
PULPROG zgpg30
TD 65536
SOLVENT CDCl3
NS 364
DS 0
SWH 22727.273 Hz
FIDRES 0.346791 Hz
AQ 1.4418420 sec
RG 45.2
DW 22.000 usec
DE 6.00 usec
TE 300.0 K
D1 2.00000000 sec
d11 0.03000000 sec
DELTA 1.89999998 sec
TD0 1
======== CHANNEL f1 ========
NUC1 13C
P1 9.70 usec
PL1 -0.50 dB
SFO1 100.6288660 MHz
======== CHANNEL f2 ========
CPDPRG2 waltz16
NUC2 1H
PCPD2 90.00 usec
PL2 -2.40 dB
PL12 15.10 dB
PL13 18.10 dB
SFO2 400.1516010 MHz
F2 - Processing parameters
SI 32768
SF 100.6178022 MHz
WDW EM
SSB 0
LB 3.00 Hz
GB 0
PC 1.00
O
N
N
Ts
(7b)
210
200
190
180
170
160
150
140
130
120
110
100
90
80
70
60
50
40
30
20
ppm

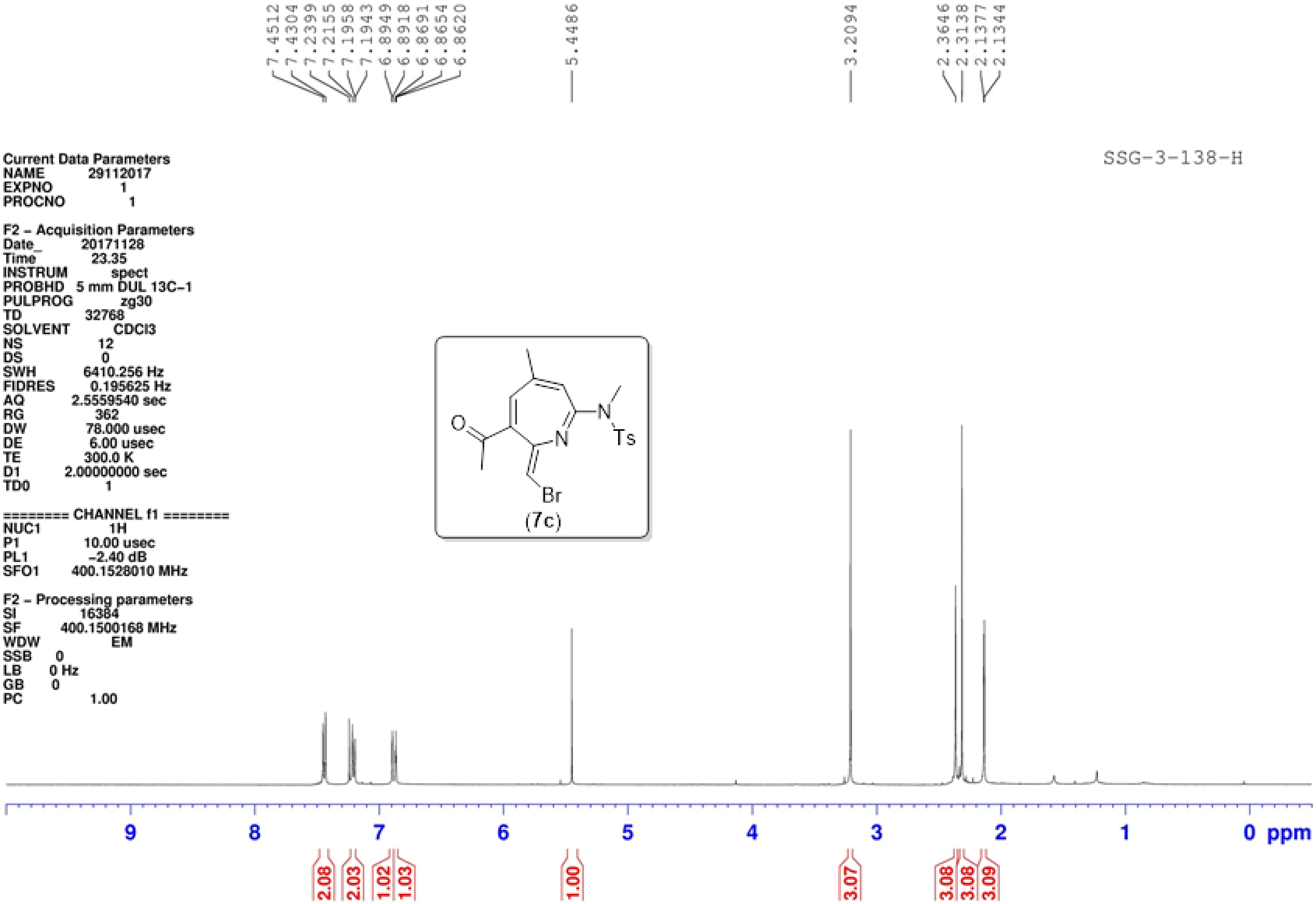

7.4512
7.4304
7.2399
7.2155
7.1958
7.1943
6.8949
6.8918
6.8691
6.8654
6.8620
5.4486
3.2094
2.3646
2.3138
2.1377
2.1344
SSG-3-138-H
Current Data Parameters
NAME 29112017
EXPNO 1
PROCNO 1
F2 - Acquisition Parameters
Date_ 20171128
Time 23.35
INSTRUM spect
PROBHD 5 mm DUL 13C-1
PULPROG zg30
TD 32768
SOLVENT CDCl3
NS 12
DS 0
SWH 6410.256 Hz
FIDRES 0.195625 Hz
AQ 2.5559540 sec
RG 362
DW 78.000 usec
DE 6.00 usec
TE 300.0 K
D1 2.00000000 sec
TD0 1
======== CHANNEL f1 ========
NUC1 1H
P1 10.00 usec
PL1 -2.40 dB
SFO1 400.1528010 MHz
F2 - Processing parameters
SI 16384
SF 400.1500168 MHz
WDW EM
SSB 0
LB 0 Hz
GB 0
PC 1.00
O
N
N
Ts
Br
(7c)
9
8
7
6
5
4
3
2
1
0 ppm
2.08
2.03
1.02
1.03
1.00
3.07
3.08
3.08
3.09

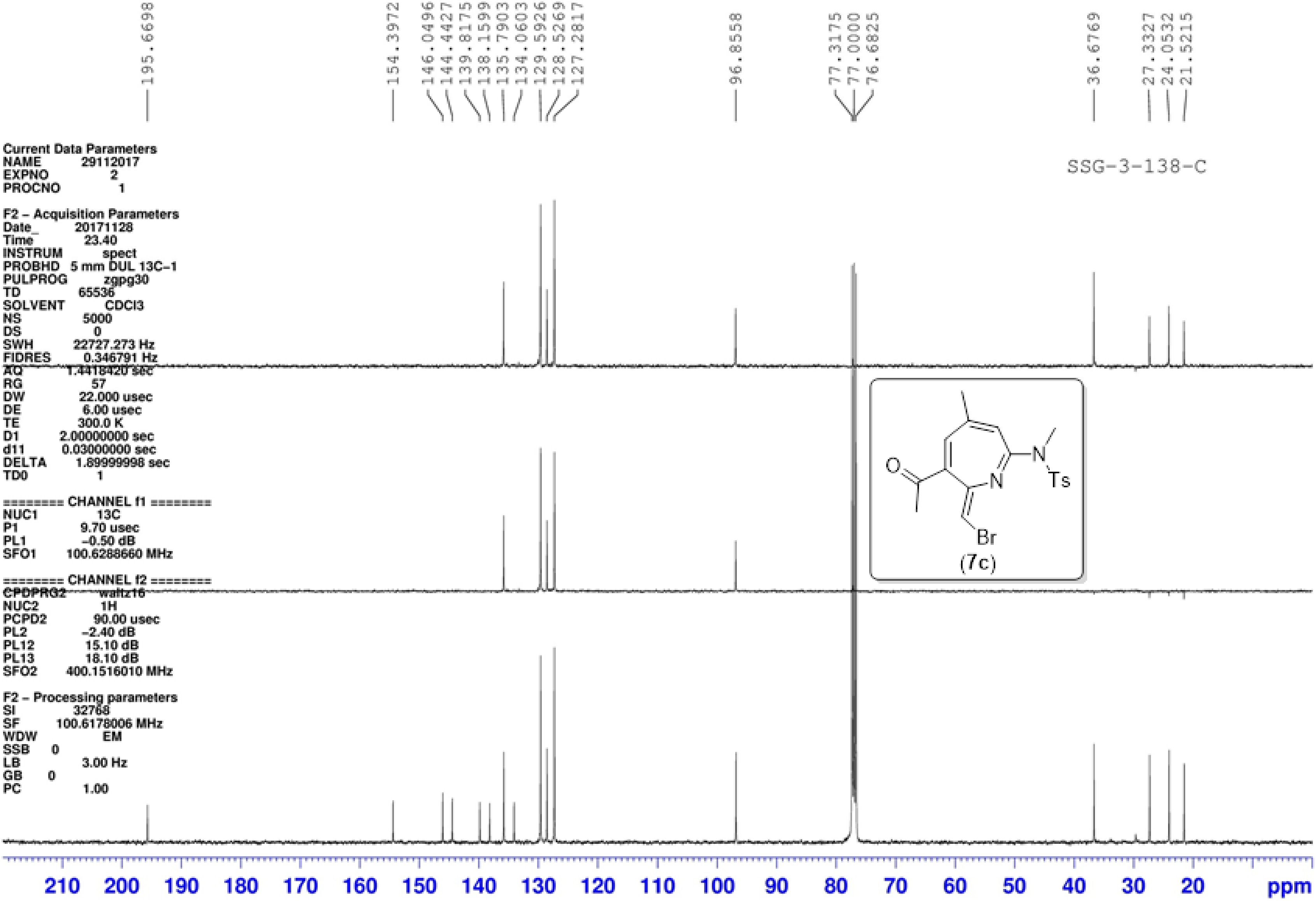
195.6698
154.3972
146.0496
144.4427
139.8175
138.1599
135.7903
134.0603
129.5926
128.5269
127.2817
96.8558
77.3175
77.0000
76.6825
36.6769
27.3327
24.0532
21.5215
SSG-3-138-C
Current Data Parameters
NAME 29112017
EXPNO 2
PROCNO 1
F2 – Acquisition Parameters
Date_ 20171128
Time 23.40
INSTRUM spect
PROBHD 5 mm DUL 13C-1
PULPROG zgpg30
TD 65536
SOLVENT CDCl3
NS 5000
DS 0
SWH 22727.273 Hz
FIDRES 0.346791 Hz
AQ 1.4418420 sec
RG 57
DW 22.000 usec
DE 6.00 usec
TE 300.0 K
D1 2.00000000 sec
d11 0.03000000 sec
DELTA 1.89999998 sec
TD0 1
======== CHANNEL f1 ========
NUC1 13C
P1 9.70 usec
PL1 -0.50 dB
SFO1 100.6288660 MHz
======== CHANNEL f2 ========
CPDPRG2 waltz16
NUC2 1H
PCPD2 90.00 usec
PL2 -2.40 dB
PL12 15.10 dB
PL13 18.10 dB
SFO2 400.1516010 MHz
F2 – Processing parameters
SI 32768
SF 100.6178006 MHz
WDW EM
SSB 0
LB 3.00 Hz
GB 0
PC 1.00
O
N
Ts
N
Br
(7c)
210 200 190 180 170 160 150 140 130 120 110 100 90 80 70 60 50 40 30 20 ppm

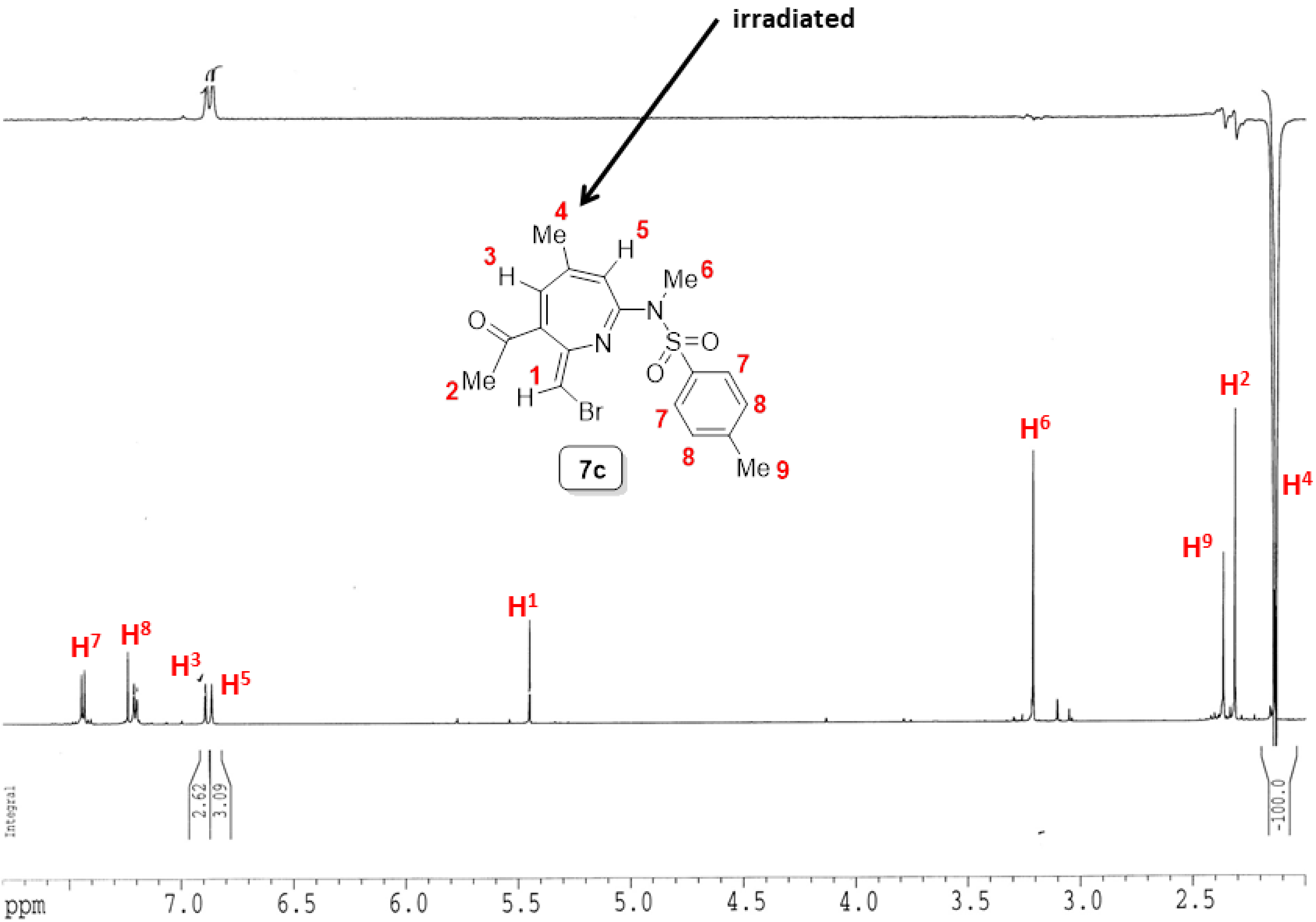
irradiated
7c
H7
H8
H3
H5
H1
H6
H9
H2
H4
Integral
2.62
3.09
-100.0
ppm
7.0
6.5
6.0
5.5
5.0
4.5
4.0
3.5
3.0
2.5

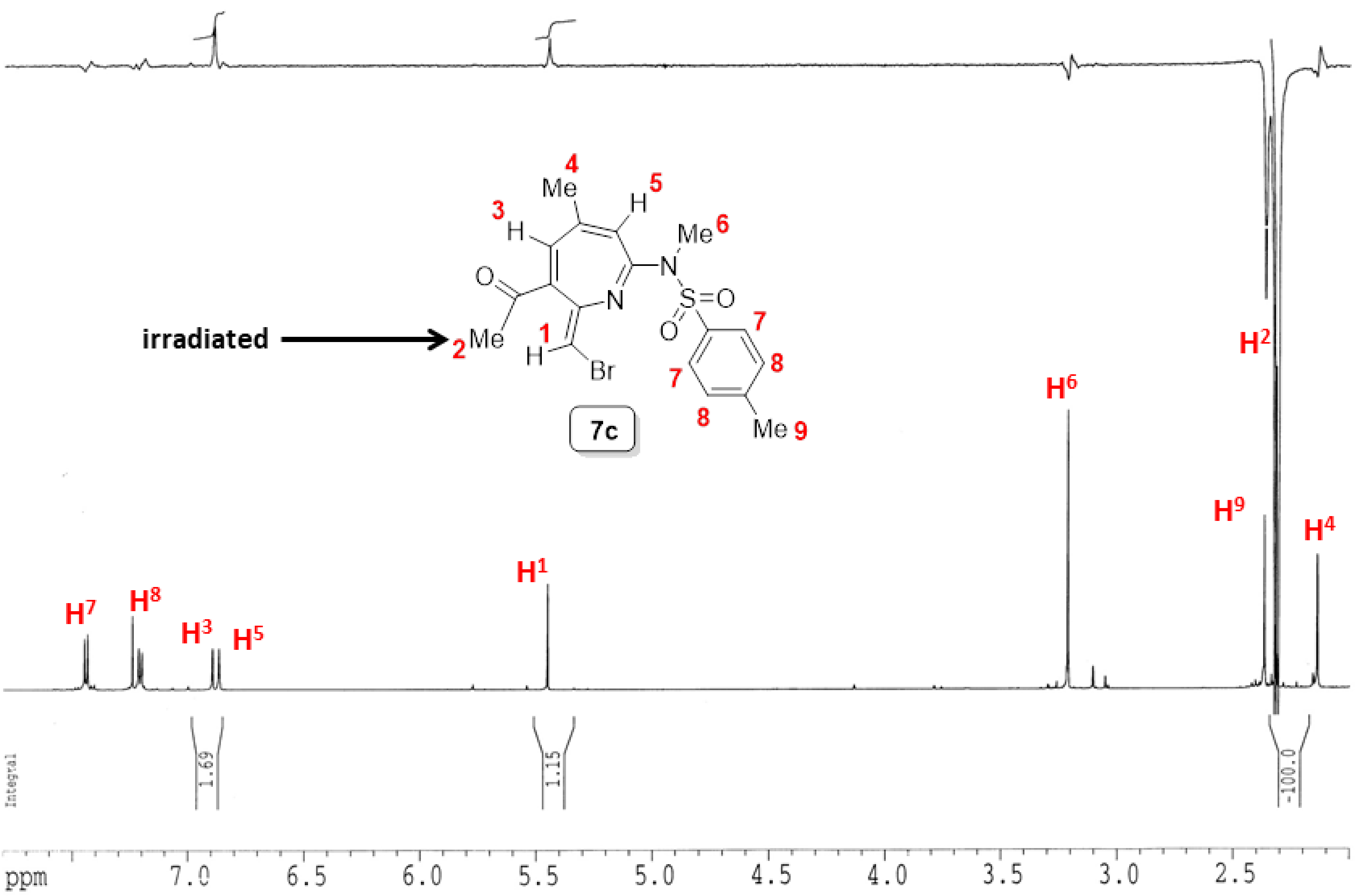

irradiated
2 Me
7c
H1
H2
H3
H4
H5
H6
H7
H8
H9
1.69
1.15
-100.0
Integral
ppm
7.0
6.5
6.0
5.5
5.0
4.5
4.0
3.5
3.0
2.5

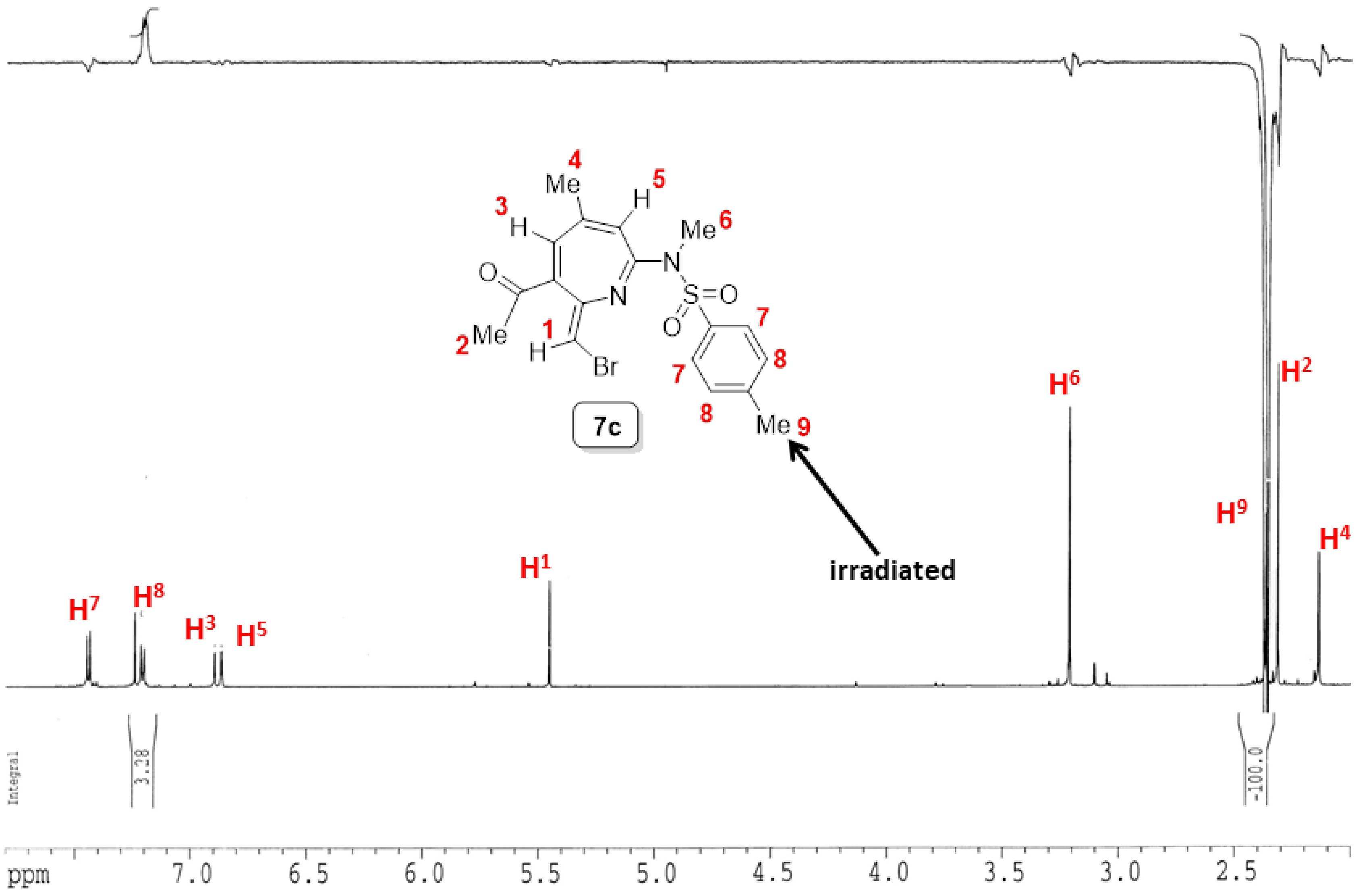
7c
irradiated
H7
H8
H3
H5
H1
H6
H9
H2
H4
Integral
3.28
-100.0
ppm
7.0
6.5
6.0
5.5
5.0
4.5
4.0
3.5
3.0
2.5

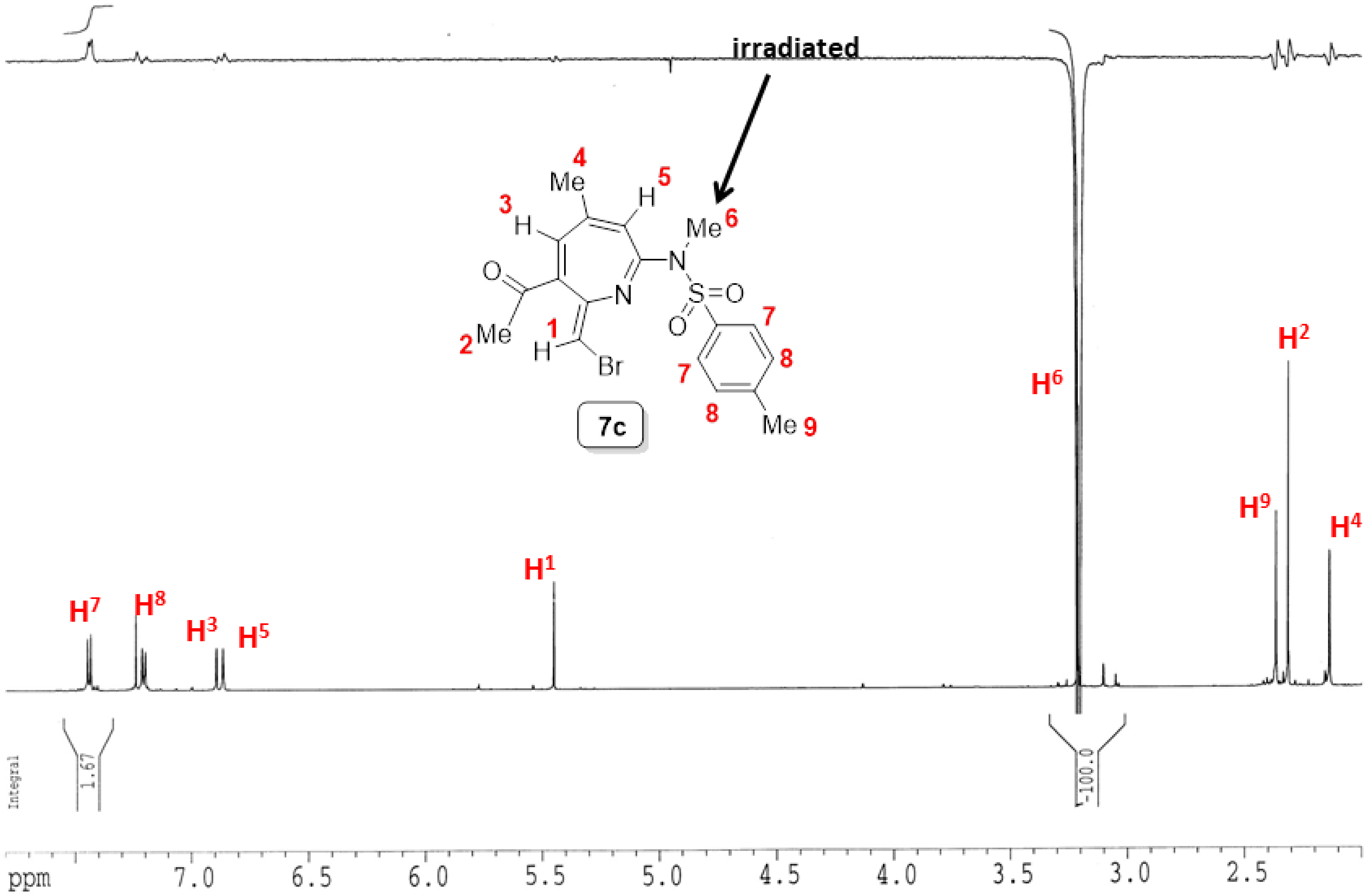
irradiated
7c
H1
H2
H3
H4
H5
H6
H7
H8
H9
ppm
7.0
6.5
6.0
5.5
5.0
4.5
4.0
3.5
3.0
2.5
Integral
1.67
-100.0

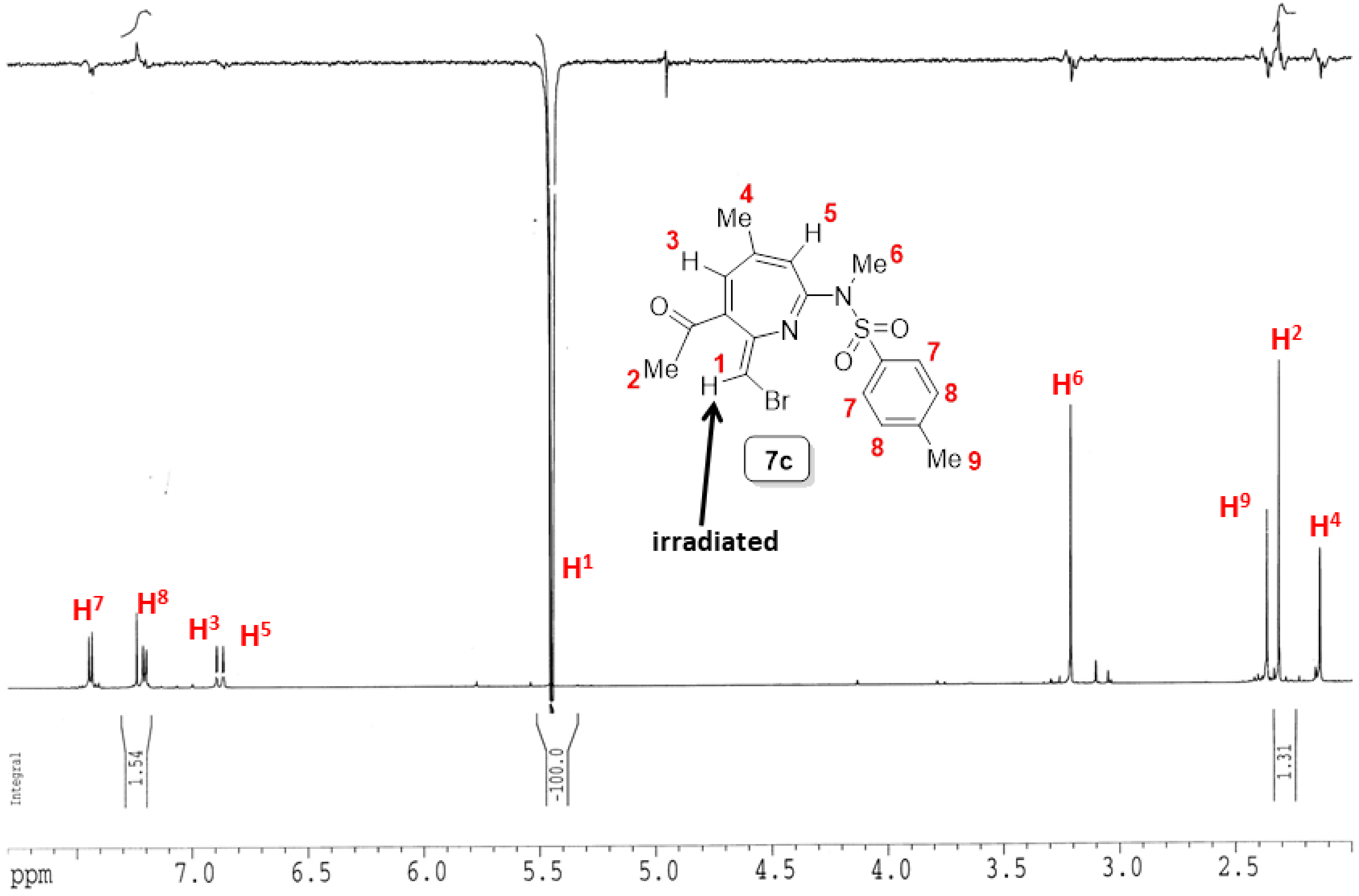
H7
H8
H3
H5
H1
irradiated
7c
H6
H9
H2
H4
Integral
1.54
-100.0
1.31
ppm
7.0
6.5
6.0
5.5
5.0
4.5
4.0
3.5
3.0
2.5

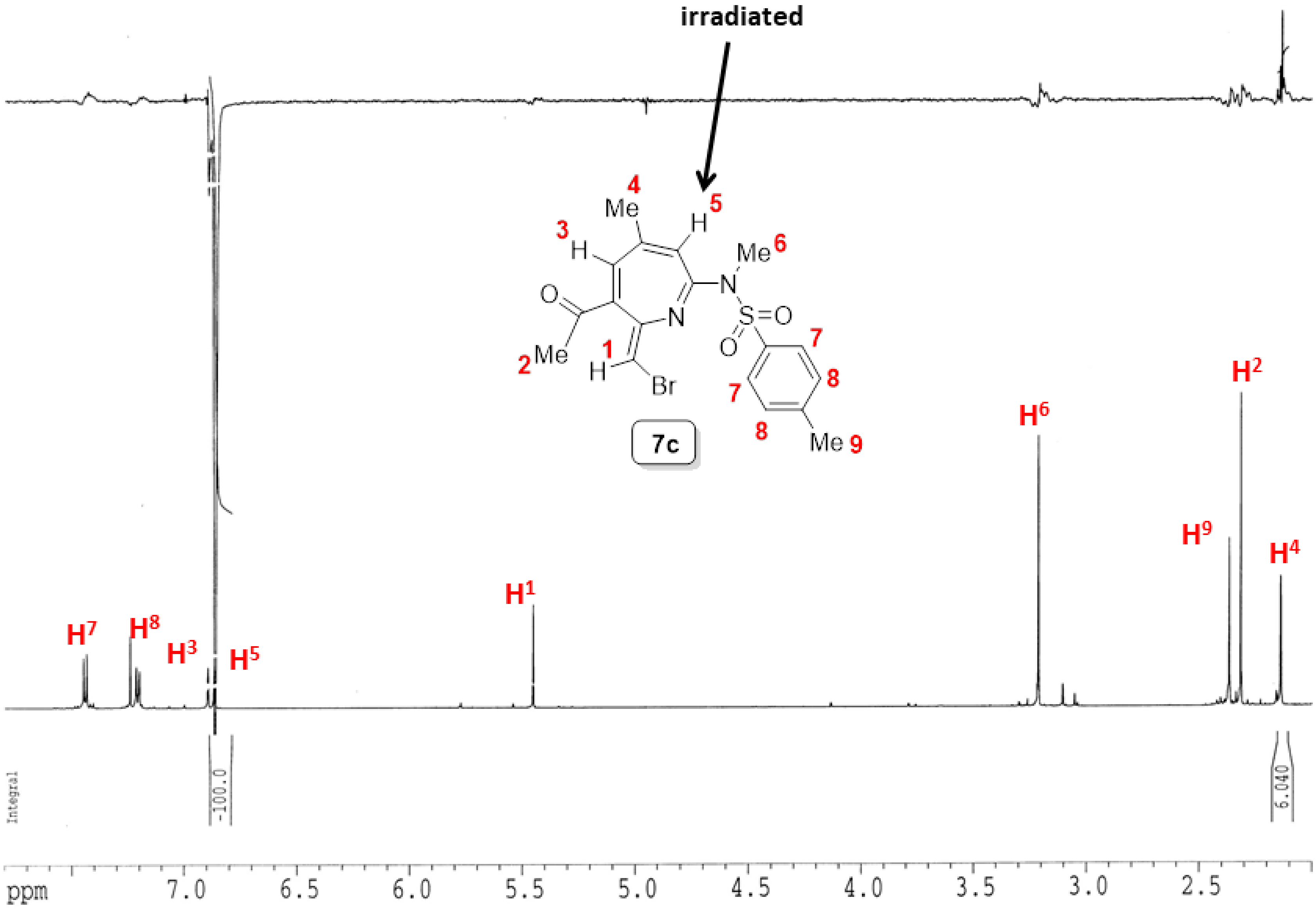

irradiated
7c
H1
H2
H3
H4
H5
H6
H7
H8
H9
Integral
-100.0
6.040
ppm
7.0
6.5
6.0
5.5
5.0
4.5
4.0
3.5
3.0
2.5

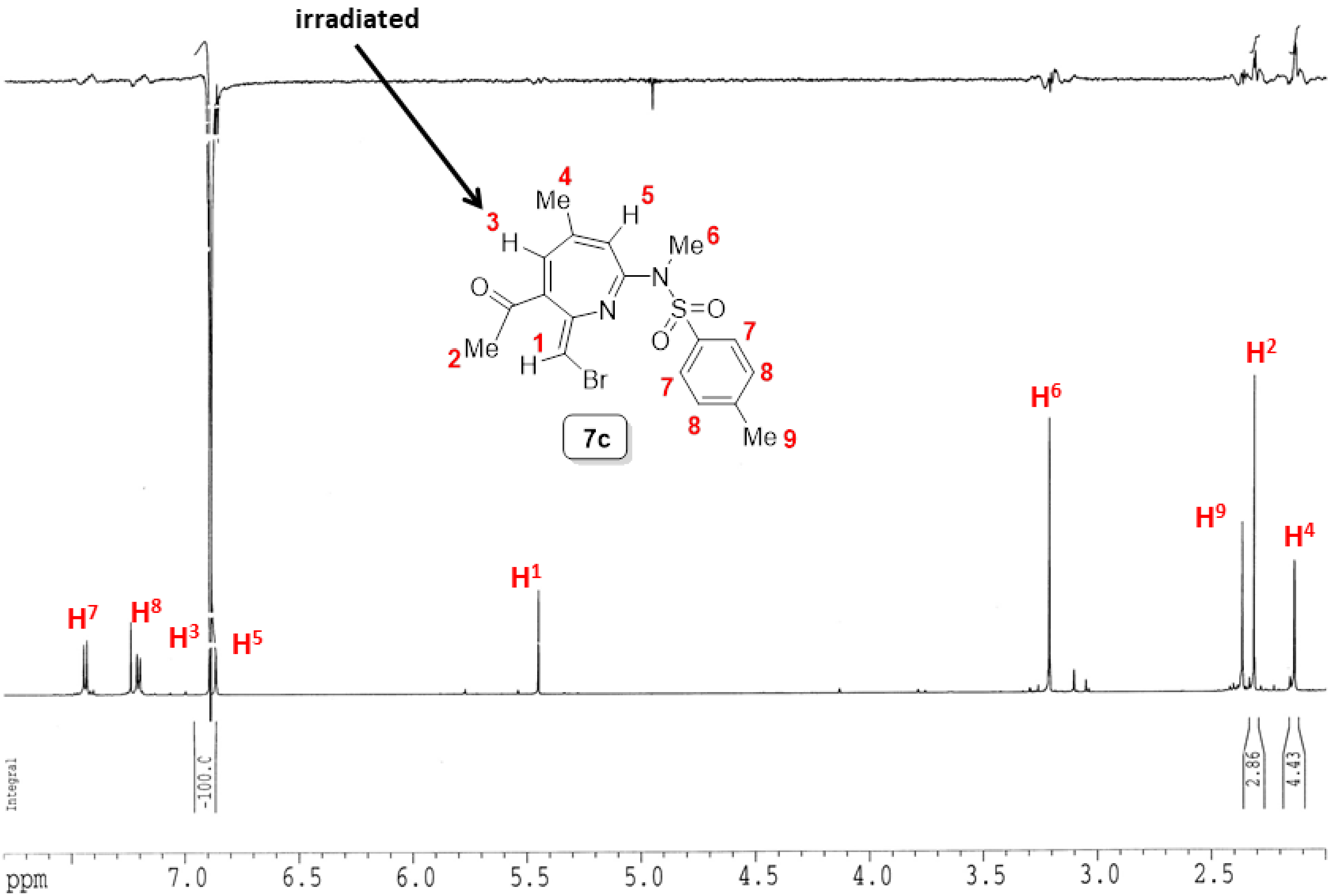
irradiated
7c
H7
H8
H3
H5
H1
H6
H9
H2
H4
Integral
-100.0
2.86
4.43
ppm
7.0
6.5
6.0
5.5
5.0
4.5
4.0
3.5
3.0
2.5

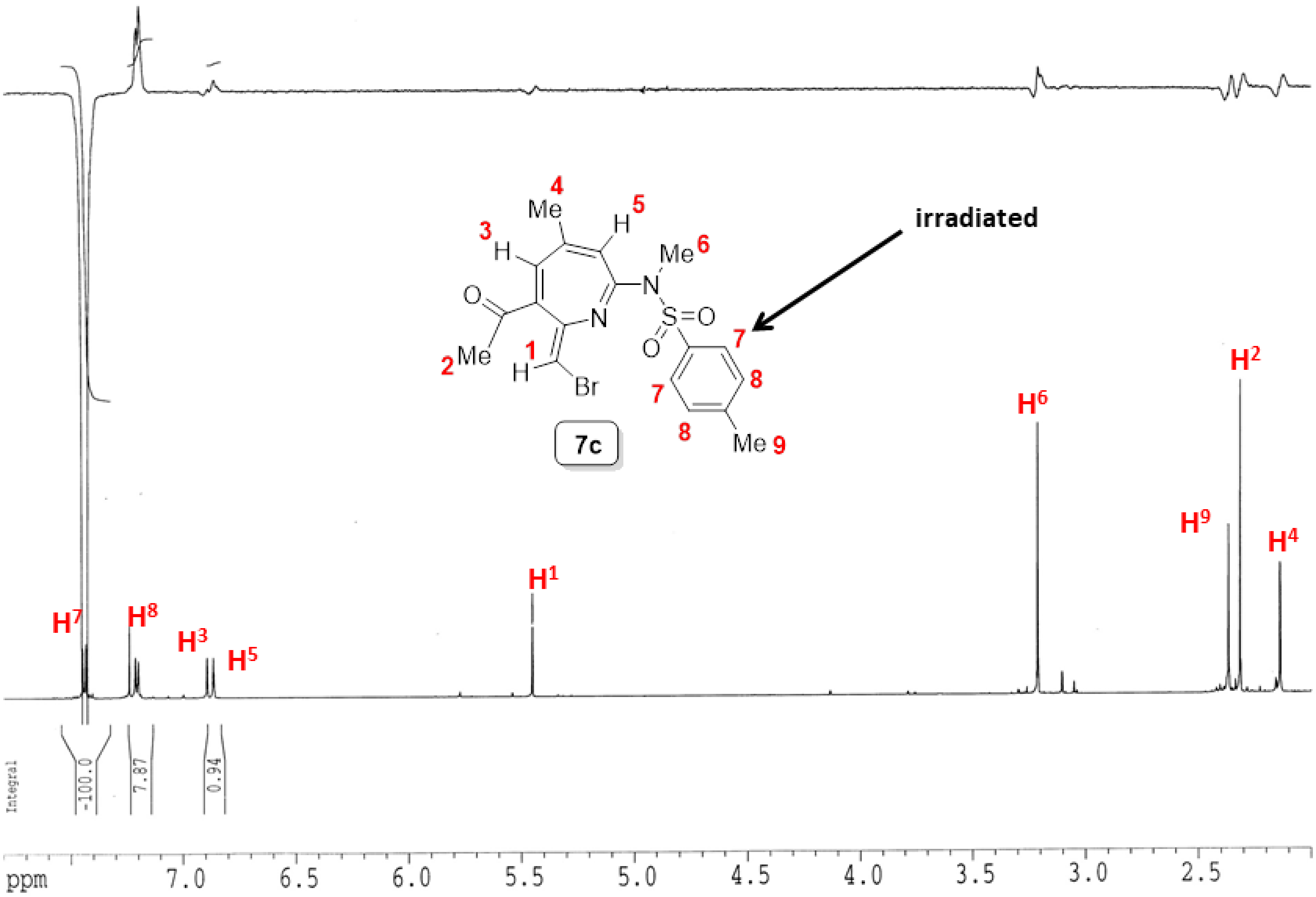
irradiated
7c
H7
H8
H3
H5
H1
H6
H9
H2
H4
Integral
-100.0
7.87
0.94
ppm
7.0
6.5
6.0
5.5
5.0
4.5
4.0
3.5
3.0
2.5
